# Catalytic Enantioselective Synthesis of Axially Chiral Diaryl Ethers Via Asymmetric Povarov Reaction Enabled Desymmetrization

**DOI:** 10.1002/advs.202403125

**Published:** 2024-07-16

**Authors:** Zidan Ye, Wansen Xie, Wei Liu, Changyu Zhou, Xiaoyu Yang

**Affiliations:** ^1^ School of Physical Science and Technology Shanghai 201210 China

**Keywords:** asymmetric catalysis, atropisomerism, desymmetrization, diaryl ethers, Povarov reaction

## Abstract

Axially chiral diaryl ethers represent a distinct class of atropisomers, characterized by a unique dual C─O axes system, which have been found in a variety of natural products, pharmaceuticals, and ligands. However, the catalytic enantioselective synthesis of these atropoisomers poses significant challenges, due to the difficulty in controlling both chiral C─O axes, and their more flexible conformations. Herein, an efficient protocol for catalytic enantioselective synthesis of axially chiral diaryl ethers is presented using organocatalyzed asymmetric Povarov reaction‐enabled desymmetrization, followed by aromatizations. This method yields a wide range of novel quinoline‐based diaryl ether atropoisomers in good yields and high enantioselectivities. Notably, various aromatization protocols are developed, resulting in a diverse set of polysubstituted quinoline‐containing diaryl ether atropisomers. Thermal racemization studies suggested excellent configurational stabilities for these novel diaryl ether atropisomers (with racemization barriers up to 38.1 kcal mol^−1^). Moreover, this research demonstrates for the first time that diaryl ether atropisomers lacking the bulky *t*‐Bu group can still maintain a stable configuration, challenging the prior knowledge in the field. The fruitful derivatizations of the functional group‐rich chiral products further underscore the value of this method.

## Graphical abstract

An efficient and stereoselective protocol is developed for the catalytic enantioselective synthesis of axially chiral diaryl ethers, a unique class of atropisomers characterized by a dual C─O axes system. This method leverages organocatalyzed asymmetric Povarov reaction‐enabled desymmetrization, followed by various aromatization protocols, which resulted in a diverse array of novel polysubstituted quinoline‐containing diaryl ether atropisomers with high enantioselectivities.

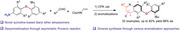

## 1 Introduction

**Figure 1:**
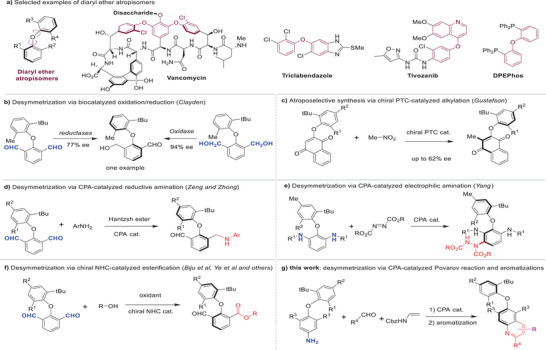
Catalytic enantioselective synthesis of axially chiral diaryl ethers atropisomers. a) Selected examples of diaryl ether atropisomers. b) Desymmetrization via biocatalyzed oxidation/reduction (Clayden). c) Atroposelective synthesis via chiral PTC‐catalyzed alkylation (Gustafson). d) Desymmetrization via CPA‐catalyzed reductive amination (Zeng and Zhong). e) Desymmetrization via CPA‐catalyzed electrophilic amination (Yang). f) Desymmetrization via chiral NHC‐catalyzed esterification (Biju et al, Ye et al and others). g) this work: desymmetrization via CPA‐catalyzed Povarov reaction and aromatizations.

The diaryl ether motif is a prevalent structure unit in organic molecules, frequently found in various natural products, pharmaceuticals, and organic functional molecules. Interestingly, increasing steric hindrance around the diaryl ether linkage can induce atropisomerism in these structures (**Figure** **1**a).^[^ ^1^ ^]^ Unlike other types of axially chiral biaryls,^[^ ^2^ ^]^ amides,^[^ ^3^ ^]^ styrenes,^[^ ^4^ ^]^ anilines,^[^ ^5^ ^]^ and others,^[^ ^6^ ^]^ which possess one single chiral axis, diaryl ether atropisomers feature a unique chiral dual C─O axes system.^[^ ^7^ ^]^ Axially chiral diaryl ether atropisomerism has been identified across a broad spectrum of organic molecules, notably within the well‐known vancomycin antibiotics, which contain two configurationally stable cyclic diaryl ether atropisomerism units.^[^ ^8^ ^]^ Moreover, the unstable atropisomeric diaryl ethers are commonly found in pharmaceuticals,^[^ ^9^ ^]^ such as triclabendazole and tivozanib, as well as in the ligand DPEPhos.^[^ ^10^ ^]^

In contrast to the extensively studied asymmetric synthesis of biaryl atropisomers,^[^ ^11^ ^]^ the methods for accessing axially chiral diaryl ethers are rather limited, particularly through catalytic enantioselective methods.^[^ ^12^ ^]^ This limitation is likely a result of the challenges involved in simultaneously controlling both chiral C─O axes and their more flexible conformations. In 2010, Turner, Clayden and co‐workers reported the first catalytic asymmetric synthesis of diaryl ether atropisomers through biocatalyzed desymmetrization using both an enantioselective oxidase and reductases; however, only one example was presented in this work (Figure 1b).^[^ ^13^ ^]^ In 2018, Gustafson and co‐workers reported the organocatalyzed atroposelective synthesis of diaryl ethers through a C(sp^2^)─H alkylation protocol, albeit with only moderate enantioselectivities (Figure 1c).^[^ ^14^ ^]^ Recently, several highly efficient and stereoselective enantioselective desymmetrization methods^[^ ^15^ ^]^ have been developed for constructing diaryl ether atropisomers. In 2023, Zhong, Zeng and co‐workers disclosed the asymmetric synthesis of axially chiral diaryl ethers via desymmetrization using chiral phosphoric acid (CPA) catalyzed reductive amination (Figure 1d).^[^ ^16^ ^]^ Subsequently, our group presented the asymmetric synthesis of diaryl ether atropisomers through the enantioselective desymmetrization of the achiral 2,6‐di‐amino‐substituted diaryl ether substrate, employing CPA‐catalyzed asymmetric electrophilic aminations^[^ ^17^ ^]^ with azodicarboxylates (Figure 1e).^[^ ^18^ ^]^ Recently, the Bijiu group, the Ye group and others have independently reported the asymmetric synthesis of these novel atropisomers via desymmetrization using chiral N‐heterocyclic carbene (NHC) catalyzed esterifications (Figure 1f).^[^ ^19^ ^]^ Noteworthily, during the submission of this manuscript, several elegant enantioselective desymmetrization methods have been disclosed for the asymmetric synthesis of axially chiral diaryl ethers from prochiral diaryl ethers.^[^ ^20^ ^]^

Nonetheless, it is worth noting that most of these methods utilized Clayden's prochiral dialdehydes as the substrate (synthetic building blocks), limiting the structural diversity of accessed diaryl ether atropisomers. Moreover, these methods usually involved a sequential desymmetrization and kinetic resolution process, resulting in diminished yields due to the formation of undesired achiral difuntionalized byproducts. Consequently, it is evident that there is a significant demand for the development of novel methods, and new platform molecules for the asymmetric synthesis of structurally diverse diaryl ether atropisomers. Herein, we introduce the use of a new prochiral diaryl ether substrate for the catalytic enantioselective synthesis of axially chiral diaryl ethers through a desymmetrization strategy, utilizing the sequential CPA‐catalyzed Povarov reaction and aromatization protocols.^[^ ^21^ ^]^ Notably, various aromatization methods have been developed to enable diverse access to novel polysubstituted quinoline‐based diaryl ether atropisomers (Figure 1g).

## 2 Results and Discussion

Our study commenced with the selection of 4‐amino‐substituted prochiral diaryl ether **1a** as the model substrate, which was readily prepared in two steps through the S_N_Ar reaction and reduction (**Table** **1**). We envisioned that the CPA‐catalyzed Povarov reaction of the prochiral diaryl ether **1a** could enable desymmetrization by selectively activating one side of the 4‐amino group, resulting in the formation of diaryl ether atropisomer bearing stereocenters, which upon oxidative aromatization would afford the quinoline‐based diaryl ether atropisomer. Encouragingly, the asymmetric Povarov reaction between **1a**, benzaldehyde (**2a**) and enamide **3a**, catalyzed by CPA **A1** (10 mol%) in THF at 20 °C, yielded the tetrahydroquinoline (THQ) **4a’**, which was followed by aromatization with DDQ (in toluene), ultimately affording the axially chiral diaryl ether **4a** in 45% yield with 88% ee (entry 1). Subsequently, a series of BINOL‐derived CPA catalysts were screened for the Povarov reaction (entries 2–7), and we were delighted that the 3,3′‐(2,4,6‐(Me)_3_C_6_H_2_)‐substituted CPA **A6** resulted in **4a** in 57% yield with 88% ee after the aromatization step (entry 6). Switching the chiral scaffold of CPA **A6** to the H8‐BINOL type yielded the product with the same ee value, albeit with a slightly diminished yield (entry 8). Various solvents were also examined for the Povarov reaction (entries 9–11); however, none of them provided superior results compared to THF. Finally, reducing the reaction temperature for the Povarov reaction was attempted (entries 12–14), and we found that −20 °C was the optimal temperature, resulting in the formation of diaryl ether **4a** in 80% yield with 95% ee (entry 13). It was worth noting that the one‐pot sequential Povarov reaction and aromatization were also viable, which produced the product with almost identical enantioselectivity, albeit in a reduced yield (entry 15).

**Table 1:** Optimizations of reaction conditions.^a)^

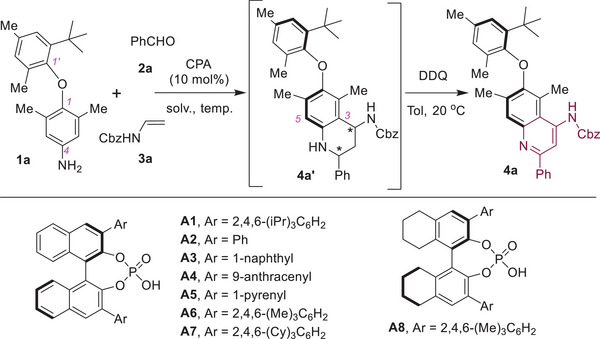					
Entry	Cat.	Solv.	Temp.(°C)	Yield (%)^b)^	Ee (%)^c)^
1	**A1**	THF	20	45	84
2	**A2**	THF	20	30	73
3	**A3**	THF	20	50	78
4	**A4**	THF	20	44	87
5	**A5**	THF	20	50	86
6	**A6**	THF	20	57	88
7	**A7**	THF	20	44	66
8	**A8**	THF	20	54	88
9	**A6**	DCM	20	34	85
10	**A6**	Tol	20	45	82
11	**A6**	1,4‐dioxane	20	41	85
12	**A6**	THF	0	70	91
13	**A6**	THF	−20	80	95
14	**A6**	THF	−40	69	95
15^d)^	**A6**	THF	−20	62	93

^a)^ Reactions were conducted with **1a** (0.1 mmol), **2a** (0.15 mmol), **3a** (0.2 mmol), and CPA (0.01 mmol, 10 mol%) in solvent (1 mL) at designated temperature for 12 h. After being purified by flash column chromatography, a solution of DDQ (0.21 mmol) in toluene (1 mL) was added at 20 °C, which was allowed to stir at room temperature for another 2 h;

^b)^ Isolated yield;

^c)^ Ee values were determined by chiral HPLC analysis;

^d)^ The two steps were performed in one pot, without purifying the **4a’** intermediate.

**Figure 2:**
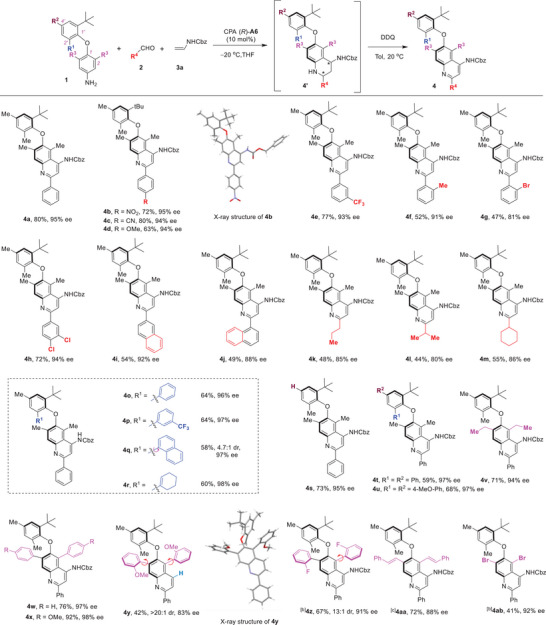
Scope for enantioselective synthesis of axially chiral diaryl ethers. a) Reactions were conducted with **1** (0.1 mmol), **2** (0.15 mmol), **3a** (0.2 mmol) and CPA (*R*)‐A6 (0.01 mmol, 10 mol%) in THF (1 mL) at −20 °C for 12 h. After being purified by flash column chromatography, a solution of DDQ (0.21 mmol) in toluene (1 mL) was added at 20 °C, which was allowed to stir at room temperature for another 2 h. b) Reactions were performed with enamide **3a** (0.3 mmol) for 36 h. c) Reactions were performed on 0.05 mmol scale with enamide **3a** (3.0 equiv.) for 36 h.

With the optimal conditions established, we set out to explore the scope of this protocol for the asymmetric synthesis of quinoline‐based diaryl ether atropisomers (**Figure** **2**). Initially, a range of *para*‐ (**4b**–**4d**), *meta*‐ (**4e**) and *ortho*‐substituted (**4f** and **4** **g**) benzaldehydes were investigated using this method, which afforded axially chiral diaryl ethers in good yields with high to excellent enantioselectivities, regardless of the substitution positions and electronic nature of the substituents. The configurations of these diaryl ethers were assigned as (*R*) by analogy to **4b**, whose structure was unambiguously determined by X‐ray crystallography analysis. In addition, the disubstituted benzaldehyde (**4** **h**) and both 2‐naphthyl (**4i**) and 1‐naphthaldehyde (**4j**) were well tolerated. Gratifyingly, various aliphatic aldehydes were also amenable to this protocol, producing chiral alkyl‐modified quinoline‐based diaryl ethers, albeit with slightly diminished yields, and enantioselectivities (**4k**–**4m**). After examination of the aldehyde variants, we turned our attention to the C2’‐substitutions of the diaryl ether substrate. Notably, switching the C2’‐methyl group to aryl groups (**4o**–**4q**) and alkenyl group (**4r**) proved to be compatible with this method, resulting in diaryl ether atropisomers with excellent enantioselectivities. Interestingly, the 1‐naphthyl‐substituted product **4q** was obtained as a mixture of two equilibrium diastereomers, likely due to the significant steric bulkiness of the aryl ether moiety, resulting in a relatively high rotation barrier of this disubstituted biaryl C─C bond. Modifications of the C4’‐position (**4s**) and both the C2’‐ and C4’‐positions (**4t** and **4u**) were also studied, which provided the chiral diaryl ethers in good yields with excellent ee values. Finally, the scope of the C2‐ and C6‐substituents of the diaryl ether substrate were investigated. Modifications of these substitutions to an ethyl group (**4v**), various aryl groups (**4w‐**‐**4z**), and an alkenyl group (**4aa**) were readily feasible under the standard conditions, yielding axially chiral diaryl ethers with high to excellent enantioselectivities. Notably, when the *ortho*‐substituted phenyl groups (**4y** and **4z**) were introduced, chiral products bearing three contiguous axes were obtained with both high diastereoselectivities and enantioselectivities, whose structures were assigned as (*R_ether_, R_c‐c_, R_c‐c_*)‐configuration by analyzing the X‐ray structure of **4y**. Remarkably, the 2,6‐di‐bromo‐substitued diaryl ether was also compatible with this method, yielding the chiral diaryl ether **4ab** with significant potential for further functionalizations.

**Figure 3:**
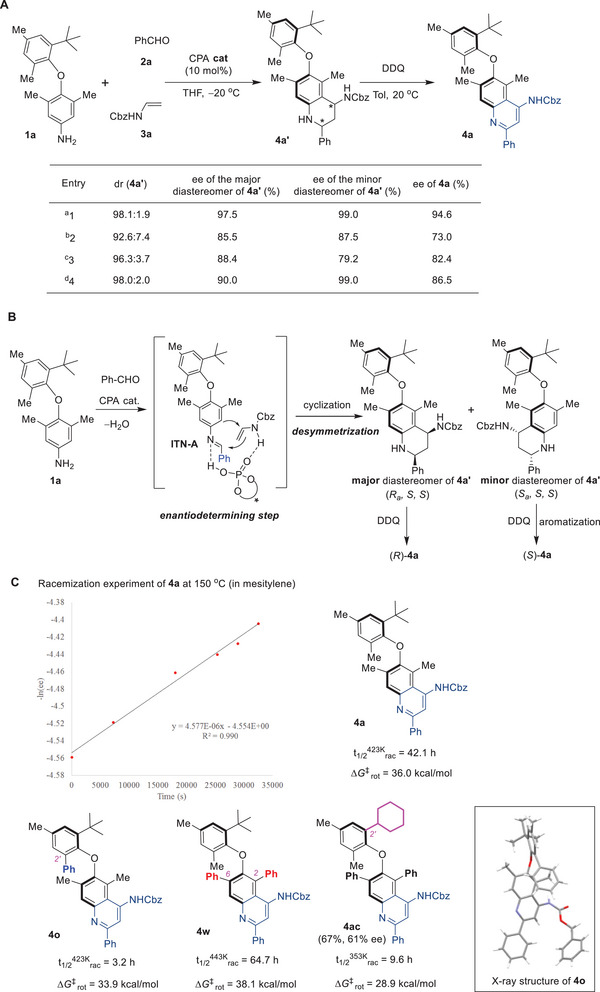
Detailed stepwise studies and investigations of the configurational stabilities of products. A) Investigations of the origin of stereoselectivity. B) Proposed reaction mechanism. C) Studies of the configurational stabilities. a) The conditions of entry 13 in Table 1 were used. b) The conditions of entry 2 in Table 1 were used. c) The conditions of entry 10 in Table 1 were used. d) The conditions of entry 4 in Table 1 were used.

To elucidate the origin of atroposelectivity for this protocol, a thorough investigation of the two steps was conducted (**Figure** **3**A). Detailed analysis of the Povarov reaction under the standard conditions (entry 1) indicated the formation of THQ **4a’** as a diastereomeric mixture with 98.1:1.9 dr and 97.5% ee (for the major diastereomer) and 99.0% ee (for the minor diastereomer), which proved inseparable by column chromatography. Subsequent oxidative aromatization of this diastereomeric mixture with DDQ produced the axially chiral diaryl ether **4a** with a reduced 94.6% ee. Moreover, we systematically examined several other conditions from the optimization Table, which resulted in the production of THQs with varying dr values and ee values. Analysis based on the ee values of the major and minor diastereomers, along with their dr values, strongly suggested that the two diastereomers exhibited opposite configurations at the chiral axis, which led to a decrease of ee value after the aromatization step. Based on these results and previous studies,^[^ ^21b^ ^]^ a plausible reaction mechanism is proposed (Figure 3B). The acid‐catalyzed condensation of arylamine **1a** and benzaldehyde afforded the imine intermediate **INT‐A**. By dual hydrogen bonding activation of the imine intermediate and the enamide through the CPA catalyst, an asymmetric [4+2] cycloaddition between these components yielded a pair of THQ diastereomers, with (*R_a_, S, S*)−**4a’** as the major diastereomer, and the (*S_a_, S, S*)−**4a’** was the minor one. Notably, this step represents the enantiodetermining step, achieving the desymmetrization of the prochiral diaryl ether axis. Subsequent oxidative aromatization with DDQ resulted in both diastereomers converging into the same axially chiral diaryl ether product **4a**, albeit with an opposite enantiomeric bias.

The configurational stability of atropisomers plays a critical role in the further applications of these chiral scaffolds. Consequently, thermal racemization experiments were conducted on the selected diaryl ether atropisomers (Figure 3C). The chiral diaryl ether **4a** exhibited excellent stability at 100 °C (in toluene), without an observed decrease in enantiopurity after 24 h. Upon raising the temperature to 150 °C (in mesitylene), a gradual decline in enantiopurity was detected, and the racemization half‐life was determined to be 42.1 h at 150 °C, corresponding to a racemization barrier of 36.0 kcal mol^−1^. Remarkably, this value is significantly higher than the previously reported range (typically between 28 and 32 kcal), highlighting the exceptional configurational stability of these diaryl ether atropisomers. Furthermore, other types of diaryl ether atropisomers, featuring aryl groups at the C2’‐ or C2, C6‐positions, were also investigated. Interestingly, substituting the C2’‐Me group with a phenyl group (**4o**) led to significant decrease in configurational stability, with a measured racemization barrier of 33.9 kcal mol^−1^. Conversely, the replacement of the C2, C6‐dimethyl groups with diphenyl groups (**4w**) resulted in dramatically increased configurational stability, with a racemization half‐life of 64.7 h at 170 °C (in diphenyl ether) and a racemization barrier of 38.1 kcal mol^−1^. This inconsistency may arise from the aryl‐aryl interaction observed between the C‐2′ phenyl group and the quinoline moiety in the X‐ray structure of **4o**, which led to a distortion of this molecular and consequently a relatively low racemization barrier (for details see Figure S1, Supporting Information). Additionally, DFT calculations were employed to study the racemization of these diaryl ether atropisomers, revealing racemization barriers of 35.5 kcal mol^−1^ (**4a**), 32.6 kcal mol^−1^ (**4o**), and 39.7 kcal mol^−1^ (**4w**), consistent with experimental results (for details see Supporting Information). With these discoveries, we further modified the *tert*‐butyl group into a cyclohexanyl group, and found that the corresponding chiral diaryl ether **2ac** could be obtained with 67% yield with 61% ee under the standard conditions. The racemization experiment was also conducted for **2ac**, which revealed a racemization half‐life of 9.6 h at 80 °C (in mesitylene) and a racemization barrier of 28.9 kcal mol^−1^, demonstrating good configuration stability of this diaryl ether atropisomer. Remarkably, this result challenges the previously held consensus that a bulky group, as large as a *tert‐*butyl group, was essential for the configurational stability of the diaryl ether atropisomers.^[^ ^1b^ ^]^

**Figure 4:**
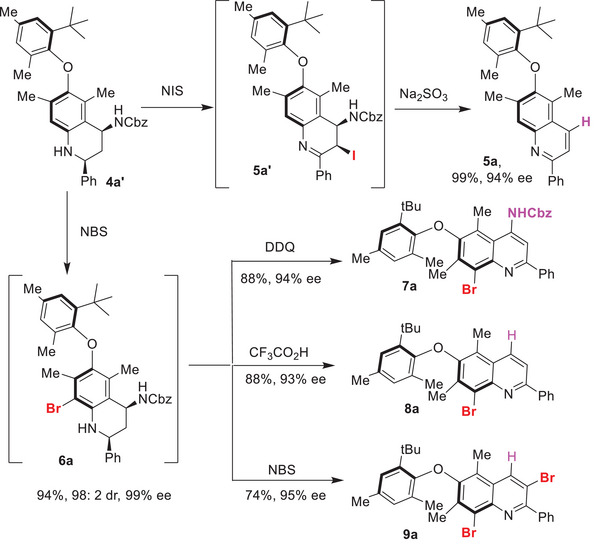
Accessing diverse axially chiral diaryl ethers through various aromatization approaches.

Given that the axial chirality of the diaryl ethers was established through the initial Povarov reaction step via enantioselective desymmetrization, we envisioned that employing various aromatization methods would provide access to a diverse array of diaryl ether atropisomer structures (**Figure** **4**). Notably, treatment of the THQ derivative **4a’** with N‐iodosuccinimide (NIS) readily afforded the 4‐unsubstituted quinoline‐based chiral diaryl ether **5a** in 99% yield with 94% ee, which was produced through the reductive elimination of the C3‐iodo‐substituted intermediate **5a’**. In contrast, when **4a’** was treated with N‐bromosuccinimde (NBS), the *ortho*‐brominated **6a** was obtained, which upon subsequent oxidative aromatization with DDQ afforded the C4‐amido‐C8‐bromo‐substituted quinoline‐based diaryl ether **7a** in 88% yield with 94% ee. Furthermore, treatment of **6a** with TFA also led to the eliminative aromatization, resulting in the formation of C8‐bromo‐substituted quinoline‐based diaryl ether **8a**. Intriguingly, subsequent treatment of **6a** with NBS generated the C3, C8‐dibromo‐substituted quinolinic diaryl ether **9a**, likely following sequential C‐3 bromination and elimination of the C‐4 amido group.

**Figure 5:**
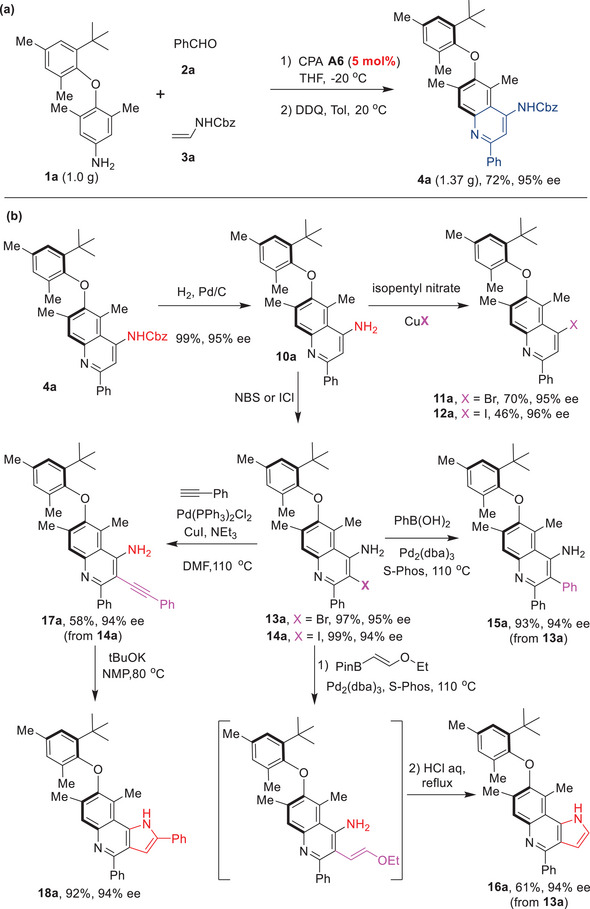
Large‐scale reaction and derivatizations of the chiral products. a) Large‐scale asymmetric synthesis. b) Derivatization of the chiral products.

To showcase the practicability of this approach, a gram‐scale reaction was performed using **1a**, **2a,** and **3a** with a reduced amount of CPA **A6** (5 mol%), which readily afforded the axially chiral diaryl ether **4a** (1.36 g) in 72% yield with 95% ee (**Figure** **5**a). The derivatizations of the standard diaryl ether atropisomer product **4a** were also investigated to demonstrate the method's versatility (Figure 5b). Removal of the *N*‐Cbz group was facilely achieved under catalytic hydrogenation conditions, yielding the 4‐amino‐quinoline‐containing diaryl ether **10a** in 99% yield with 95% ee. Subsequent Sandmayer reactions of **10a** with isopentyl nitrite and CuBr or CuI afforded the C4‐brominated (**11a**) or iodinated (**12a**) quinoline‐based diaryl ethers, further enhancing structural diversity in conjunction with the methods depicted in Figure 4. Furthermore, treatment of **10a** with electrophilic halogenation reagents, such as NBS and ICl, generated the C‐3 brominated or iodinated quinoline‐containing diaryl ether **13a** and **14a** in high yields. The Suzuki coupling of **13a** with phenyl boronic acid afforded **15a** in 93% yield in 94% ee, while the sequential Suzuki coupling with ethoxyvinyl boronic ester and hydrolysis/cyclization resulted in the pyrroloquinoline‐containing diaryl ether **16a** in 61% yield with 94% ee. Moreover, the Sonogashira coupling of **14a** with phenylacetylene readily afforded **17a**, which upon treatment with KOtBu, gave access to another pyrroloquinoline‐based diaryl ether atropisomer **18a** in 92% yield with 94% ee. It was noteworthy that the enantiopurities of these atropisomers were well retained under these harsh conditions, thus further emphasizing the exceptional configurational stability exhibited by these diaryl ether atropisomers.

## 3 Conclusion

In conclusion, we have developed a versatile method for the catalytic enantioselective synthesis of axially chiral diaryl ethers through CPA‐catalyzed asymmetric Povarov reaction‐enabled desymmetrization and subsequent aromatizations. Notably, in combination with various aromatization approaches, a diverse array of novel polysubstituted quinoline‐based diaryl ether atropisomers were generated with good yields and high enantioselectivities. Extensive racemization experiments were performed on these novel quinolone‐based diaryl ether atropisomers, unveiling their exceptional configurational stabilities (with racemization barriers up to 38.1 kcal mol^−1^). Remarkably, one diaryl ether product lacking a tert‐butyl group demonstrated substantial configurational stability, which challenges the previous assertion that a bulky group as large as *tert*‐butyl group is indispensable for ensuring the configurational stability of diaryl ether atropisomers. Furthermore, the potential for large‐scale asymmetric synthesis and diverse derivatizations of these diaryl ether atropisomers underscore the value of this method.

## 4 Experimental Section

### General Procedure for Asymmetric Synthesis of Product **4**

To a stirred solution of **1** (0.1 mmol, 1.0 equiv.), CPA (*R*)‐**A6** (10 mol%) and activated 4Å molecular sieves (ca. 50 mg) in THF (0.5 mL) were added the corresponding aldehyde **2** (0.15 mmol, 1.5 equiv.) at room temperature and stirred at −20 °C for 30 min. Following that, a solution of enamide **3a** (0.2 mmol, 2.0 equiv.) in THF (0.5 mL) was added dropwise into the reaction mixture via syringe and the mixture was allowed to stir at the same temperature for another 12 h. After completion of the Povarov reaction as monitored by TLC analysis, the product **4′** was afforded by flash column chromatography (petroleum ether/EtOAc = 9:1). To a solution of the above product **4′** in toluene (1 mL) was added 1,2‐dichloro‐4,5‐dicyanobenzoquinone (DDQ, 0.21 mmol, 2.1 equiv.) in portions at rt and the reaction mixture was allowed to stir for 2 h. The reaction was then diluted with EtOAc, and washed with saturated Na_2_SO_3_ solution two times and saturated aqueous NaHCO_3_ solution two times. The organic layer was dried over Na_2_SO_4_ and concentrated under vacuum to give a residue, which was purified by flash column chromatography (petroleum ether/EtOAc = 12:1) to give the product **4**.

The X‐ray crystallographic coordinates for structures reported in this study have been deposited at the Cambridge Crystallographic Data Centre (CCDC), under deposition numbers CCDC 2330129 (for **4b**), 2330130 (for **4y**) and 2353648 (for **4o**). These data can be obtained free of charge from The Cambridge Crystallographic Data Centre via www.ccdc.cam.ac.uk/data_request/cif.

## Conflict of Interest

The authors declare no conflict of interest.

## Author Contributions

Z.Y., W.L. and C.Z. performed the experiments. W.X. performed computational studies on the racemization barriers of these atropisomers. X.Y. directed the project and wrote the paper with the feedback from other authors.

## Supporting information

Supporting Information

## Data Availability

The data that support the findings of this study are available in the supplementary material of this article.
