# Supplementary material for: Catalytic Enantioselective Synthesis of Axially Chiral Diaryl Ethers Via Asymmetric Povarov Reaction Enabled Desymmetrization
Source: Adv Sci (Weinh). 2024 Jul 16;11(35):2403125. doi: 10.1002/advs.202403125 (PMC11425261; doi:10.1002/advs.202403125)

Table of Contents

General Information 2

Synthesis of substrates........ 3

Method A for the synthesis of substrate **1a**, **1o-1q**, **1s-1u** 3

Method B for the synthesis of substrate **1v** 4

Method C for synthesis of substrate **1w-1y**........ 5

Method D for synthesis of substrate **1aa**........ 6

Method E for synthesis of the key intermediate **1ab**........ 7

Method F for synthesis of substrate **1ac**........ 8

Enantioselective synthesis of axially chiral diaryl ethers........ 20

Determination of $t_{1/2}^{rac}$ and racemization barrier 46

DFT calculation of the racemization process 53

Accessing divers axially chiral diaryl ethers through various aromatization approaches 75

Large-scale asymmetric reaction........ 81

Derivatizations of the chiral products........ 82

Reference........ 91

X-Ray structures........ 92

HPLC traces 98

NMR spectra........ 142

General Information

Unless otherwise noted, all commercial reagents were used without further purification. Dichloromethane, toluene, ether, THF were purified by passage through an activated alumina column under argon. Thin-layer chromatography (TLC) analysis of reaction mixtures was performed using Huanghai silica gel HSGF254 TLC plates, and visualized under UV or by staining with ceric ammonium molybdate or potassium permanganate. Flash column chromatography was carried out on Huanghai Silica Gel HHGJ-300, 300-400 mesh. Nuclear magnetic resonance (NMR) spectra were recorded using Bruker Avance III HD spectrometer (FT, 500 MHz for ^{1}H, 126 MHz for ^{13}C, 471 MHz for ^{19}F, or 400 MHz for ^{1}H, 101 MHz for ^{13}C, 376 MHz for ^{19}F). ^{1}H and ^{13}C chemical shifts are reported in ppm downfield of tetramethylsilane and referenced to residual solvent peak ($CDCl_3$, δH = 7.26 and δC = 77.16; CD_3OD, δH= 3.31 and δC = 49.00; $(CD_3)_2O$, δH= 2.05 and δC = 29.84; $(CD_3)_2SO$, δH= 2.50 and δC = 39.52). Multiplicities are reported using the following abbreviations: s = singlet, d = doublet, t = triplet, q = quartet, m = multiplet, br = broad resonance. FT-IR spectra were recorded on ThermoFisher Scientific Nicolet iS7 Spectrometer, and absorption frequencies are reported in reciprocal centimeters (cm^{-1}). Mass spectral data were obtained from the Agilent Technologies 6230 TOF LC/MS spectrometer in electrospray ionization (ESI^{+}) mode. X-Ray structure analyses were performed using a Bruker D8 Venture X-ray single crystal diffractometer and D8 Venture metalJet X-Ray single crystal diffractometer. Optical rotations were measured with an Autopol V Plus/VI digital polarimeter. Enantiomeric excesses were determined on an Agilent 1260 Chiral HPLC using IA, IB, IB N-5, IC, ID columns. Racemic products were synthesized by carrying out the reactions using *rac*-**A6** as catalyst.

Synthesis of substrates

Method A for the synthesis of substrate 1a, 1o-1q, 1s-1u

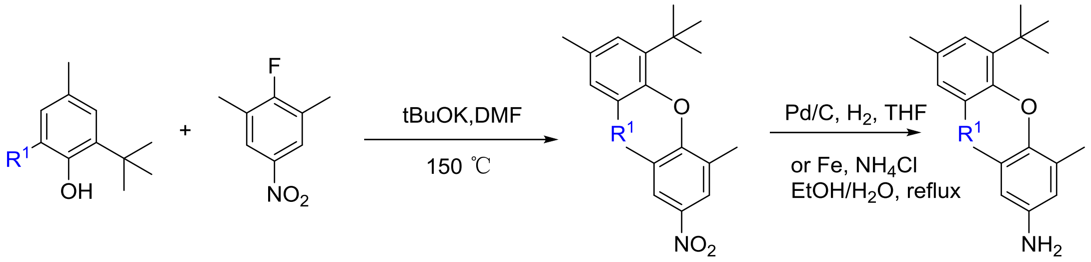

Compound **S1** were prepared according to the previous literature[1]

To a solution of phenol **S1** (1.1 equiv.) in DMF (1.0 M) was added tBuOK (1.5 equiv.) at rt. After stirring for 30 min, a solution of **S2** (1.0 equiv.) in DMF (1.0 M) was added. After stirring at 150 ^{o}C for 12h, the reaction mixture was poured into H_2O and then extracted with EtOAc for three times. The combined organic layers were washed with brine, dried over Na_2SO_4, filtered and concentrated to give a residue, which was purified by column chromatography (petroleum ether/EtOAc = 100:1) to give a residue **S3**.

To a solution of **S3** in THF (1.0 M) was added Pd/C (10 wt %) at rt, and the flask was evacuated and purged with H_2 fort 3 times. After stirring at rt under H_2 atmosphere (1 atm) for 12 hours, the mixture was filtered through Celite and concentrated under vacuum to give a residue, which was purified by column chromatography (petroleum ether/EtOAc) to give the product **1a, 1o-1q, 1s-1u**.

To a round-bottomed flask charged with the crude product **S3**, Fe (4.0 equiv.), NH_4Cl (2.0 equiv.) was added EtOH/H_2O (2:1, 0.5 M). The reaction mixture was stirred in oil bath at 90 ^{o}C and refluxed overnight. After completion of the reaction as monitored by TLC analysis, the reaction mixture was diluted with EtOAc and filtered through Celite. The filtrate was extracted with EtOAc for 3 times, and the combined organic layer was washed with brine, dried over $NaSO_4$ and concentrated under vacuum to

give a residue, which was purified by column chromatography (petroleum ether/EtOAc) to afford the product **1r**.

Method B for the synthesis of substrate 1v

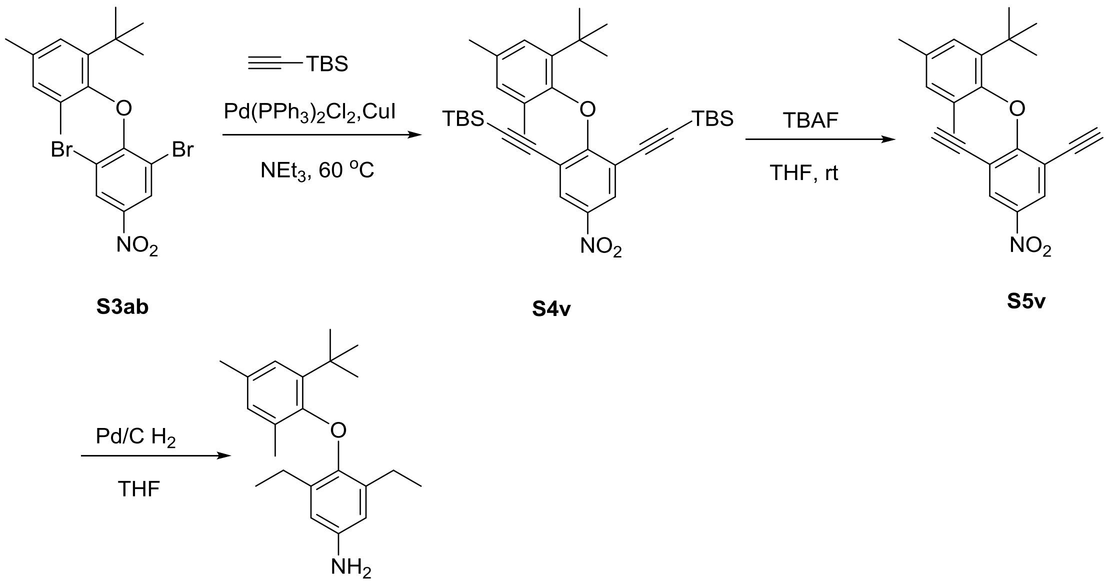

1v

For the synthesis of key intermediate **S3ab**, see method **E**

To a flask containing **S3ab** (611 mg, 1.3 mmol, 1.0 equiv.), tert-butyl(ethynyl)dimethylsilane (0.8 mL, 4.3 mmol, 3.3 equiv.), CuI (25 mg, 0.13 mmol, 10% mol) and $Pd(PPh_3)_2Cl_2$ (94 mg, 0.13 mmol, 10% mol) was added dry NEt_3 (10 mL) under N_2 atmosphere at rt, and the mixture was then warmed to 60 ^oC. After completion of the reaction as monitored by TLC analysis, the reaction was filtered through celite and the filter cake was washed by EtOAc. The filtrate was collected and concentrated under vacuum to give a residue, which was purified by column chromatography (petroleum ether/DCM = 15:1) to afford the crude product **S4v** as a yellow solid for the next deprotection reaction.

To a solution of **S4v** mentioned above (288 mg, 0.5 mmol, 1.0 equiv.) in THF (0.5 mL) was added TBAF (1 M in THF, 0.75 mL, 1.5 equiv.) at rt. After stirring for 10 min, then the reaction mixture was extracted with EtOAc for 3 times. The combined organic layer was dried over Na_2SO_4, filtered and concentrated in vacuo to give a

residue, which was purified by column chromatography (petroleum ether/DCM = 10:1) to afford the crude product **S5v** as yellow foam for the next catalytic hydrogenation.

To a solution of **S5v** in THF (5 mL) was added Pd/C (20 mg, 10 wt %) at rt, and the flask was then evacuated and purged with H_2 fort 3 times. After stirring at rt under H_2 atmosphere (1 atm) for 12 hours, the mixture was filtered through Celite and concentrated under vacuum to give a residue, which was purified by column chromatography (petroleum ether/EtOAc 8:1) to give the product **1v** as a white solid (48.0 mg, 30% yield for two steps).

Method C for synthesis of substrate 1w-1y

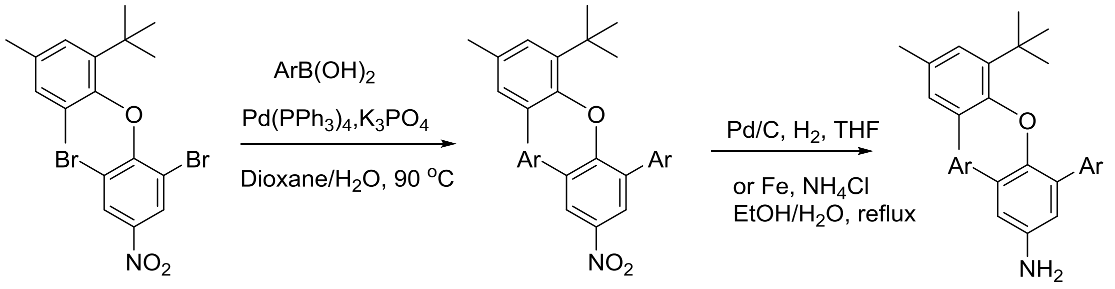

S3ab **S4** **1w-1z**

To a flask containing **S3ab** (1.0 equiv.), $ArB(OH)_2$ (3.0 equiv.), K_3PO_4 (5.0 equiv.) and $Pd(PPh_3)_4$ (20 mol%) was added dioxane/H_2O (5:1, 1.0 M) under N_2 atmosphere at rt. After stirring at 90 °C for 12 hours, the mixture was then allowed to cool to rt and diluted with EtOAc. The reaction mixture was extracted with EtOAc for 3 times. The combined organic layer was washed with brine, dried over $NaSO_4$ and concentrated under vacuum to give a residue, which was purified by column chromatography (petroleum ether/EtOAc = 100: 1) to afford the crude product **S4** for the next catalytic hydrogenation.

The crude product **S4** was treated with palladium on carbon (10% wt, 0.1 equiv.) in THF (10 mL, 1.0 M) and stirred overnight under hydrogen atmosphere at room

temperature. After completion of the reaction as monitored by TLC analysis, the mixture was filtered through celite, and the filtrate was concentrated under vacuum to give a residue, which was purified by column chromatography (petroleum ether/EtOAc) to afford the product **1w-1y**.

To a round-bottomed flask charged with the crude product **S4**, Fe (4.0 equiv.) and NH_4Cl (2.0 equiv.) was added EtOH/H_2O (2:1, 0.5 M) at rt. The reaction mixture was warmed to 90 ^oC and then refluxed overnight. After completion of the reaction as monitored by TLC analysis, the reaction mixture was diluted with EtOAc and filtered through celite. The filtrate was extracted with EtOAc for 3 times, and the combined organic layer was washed with brine, dried over $NaSO_4$ and concentrated under vacuum to give a residue, which was purified by column chromatography (petroleum ether/EtOAc) to afford the product **1z**.

Method D for synthesis of substrate 1aa

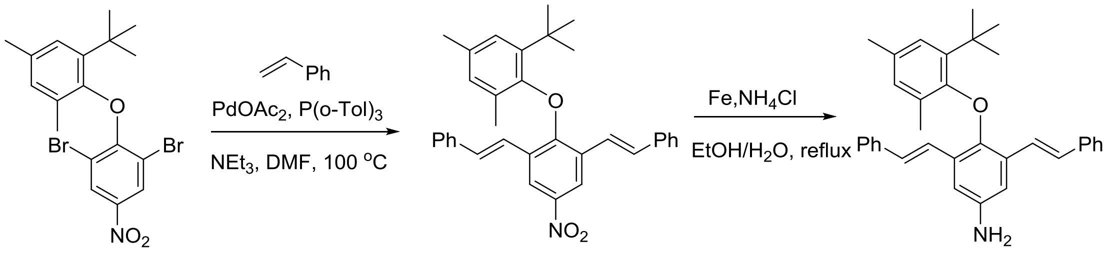

S3ab **S4aa** **1aa**

To a flask containing **S3ab** (228.6 mg, 0.5 mmol, 1.0 equiv.), styrene (0.2 mL, 1.5 mmol, 3.0 equiv.), $PdOAc_2$ (23 mg, 0.1 mmol, 10 mol%), P(o-Tol) (46 mg, 0.15 mmol, 30 mol%) and NEt_3 (0.35 mL, 2.5 mmol, 5.0 equiv.) was added DMF (1 mL) under N_2 atmosphere at rt. After stirring at 100 ^oC for 20 hours, the mixture was then allowed to cool to rt and filtered through Celite. The filtrate was concentrated under vacuum to give a residue, which was purified by column chromatography (petroleum ether/EtOAc = 50:1) to afford the crude product **S4aa** as a yellow solid.

To a round-bottomed flask charged with the crude product **S4aa**, Fe (4.0 equiv.) and NH_4Cl (2.0 equiv.) was added EtOH/H_2O (2:1, 0.5 M). The reaction mixture was warmed to 90 °C and then refluxed overnight. After completion of the reaction as monitored by TLC analysis, the reaction mixture was diluted with EtOAc and filtered through celite. The filtrate was extracted with EtOAc for 3 times, and the combined organic layer was washed with brine, dried over $NaSO_4$ and concentrated under vacuum to give a residue, which was purified by column chromatography (petroleum ether/EtOAc = 6: 1) to afford the product **1aa** as yellow foam (81 mg, 34% yield for two steps).

Method E for synthesis of the key intermediate 1ab

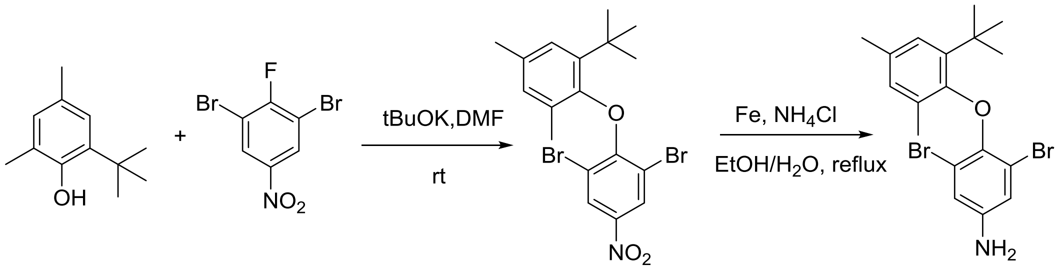

S1 **S2ab** **S3ab** **1ab**

To a solution of **S1** (1.57 g, 8.8 mmol, 1.1 equiv.) in DMF (1.0 M) was added tBuOK (1.35g, 12.0 mmol, 1.5 equiv.) at rt. After stirring for 30 min, a solution of **S2ab** (2.39 g, 8.0 mmol, 1.0 equiv.) in DMF (1.0 M) was added. After stirring at rt for 24 h, the reaction mixture was poured into H_2O and then extracted with EtOAc for three times. The combined organic layers were washed with brine, dried over Na_2SO_4, filtered and concentrated to give a residue, which was purified by column chromatography (petroleum ether/EtOAc 100:1) to give the product **S3ab** as a yellow solid (2.2 g, 57% yield).

To a round-bottomed flask charged with **S3ab**, Fe (4.0 equiv.) and NH_4Cl (2.0 equiv.) was added EtOH/H_2O (2: 1, 0.5 M). The reaction mixture was warmed to 90 °C and then refluxed overnight. After completion of the reaction as monitored by TLC

analysis, the reaction mixture was diluted with EtOAc and filtered through celite. The filtrate was extracted with EtOAc for 3 times, and the combined organic layer was washed with brine, dried over $NaSO_4$ and concentrated under vacuum to give a residue, which was purified by column chromatography (petroleum ether/EtOAc) to afford the product **1ab** as a yellow solid (740 mg, 55% yield).

Method F for synthesis of substrate 1ac

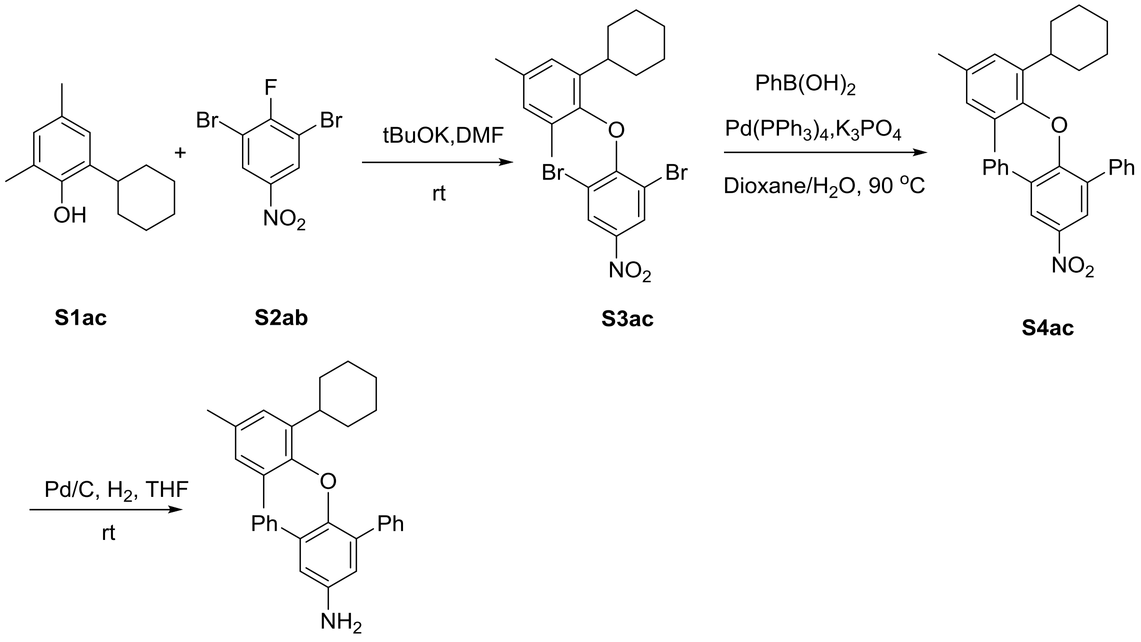

1ac

To a solution of **S1ac** (449.5 g, 2.2 mmol, 1.1 equiv.) in DMF (1.0 M) was added tBuOK (336.6 mg, 3.0 mmol, 1.5 equiv.) at rt. After stirring for 30 min, a solution of **S2ab** (598 mg, 2.0 mmol, 1.0 equiv.) in DMF (1.0 M) was added. After stirring at rt for 24 h, the reaction mixture was poured into H_2O and then extracted with EtOAc for three times. The combined organic layers were washed with brine, dried over Na_2SO_4, filtered and concentrated to give a residue, which was purified by column chromatography (petroleum ether/EtOAc 100:1) to give the crude product **S3ac** as a yellow solid.

To a flask containing crude product of **S3ac** (1.0 equiv.), $PhB(OH)_2$ (3.0 equiv.), K_3PO_4 (5.0 equiv.) and $Pd(PPh_3)_4$ (20 mol%) was added Toluene (1.0 M) under N_2 atmosphere at rt. After stirring at 120 ^oC for 12 hours, the mixture was then allowed to cool to rt and diluted with EtOAc. The reaction mixture was extracted with EtOAc for 3 times. The combined organic layer was washed with brine, dried over $NaSO_4$ and concentrated under vacuum to give a residue, which was purified by column chromatography (petroleum ether/EtOAc = 100: 1) to afford the crude product **S4ac** for the next catalytic hydrogenation.

The crude product **S4ac** was treated with palladium on carbon (10% wt, 0.1 equiv.) in THF (10 mL, 1.0 M) and stirred overnight under hydrogen atmosphere at room temperature. After completion of the reaction as monitored by TLC analysis, the mixture was filtered through celite, and the filtrate was concentrated under vacuum to give a residue, which was purified by column chromatography (petroleum ether/EtOAc = 6: 1) to afford the product **1ac**. (255 mg, 62% yield for three steps)

4-(2-(tert-butyl)-4,6-dimethylphenoxy)-3,5-dimethylaniline (**1a**)

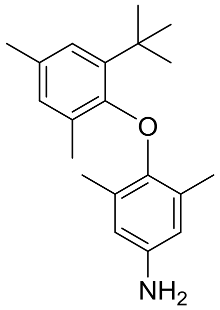

1a was prepared with 30.0 mmol scale according to the method **A** as a brown solid. (5.32 g, 60% yield for two steps)

^{1}H NMR (500 MHz, Chloroform-*d*) δ 7.03 (d, *J* = 2.7 Hz, 1H), 6.72 (d, *J* = 2.8 Hz, 1H), 6.35 (s, 2H), 3.36 (s, 2H), 2.29 (d, *J* = 2.7 Hz, 3H), 1.99 (d, *J* = 2.7 Hz, 6H), 1.73 (d, *J* = 2.8 Hz, 3H), 1.46 (d, *J* = 2.3 Hz, 9H). ^{13}C NMR (126 MHz, Chloroform-*d*) δ

152.7, 146.6, 140.8, 139.0, 131.0, 130.6, 128.6, 127.2, 125.9, 116.2, 35.4, 30.6, 21.0, 18.1, 18.0. m/z HRMS (ESI) found $[M+H]^+$ 298.2158, $C_{20}H_{28}NO^+$ requires 298.2166.

4-((3-(tert-butyl)-5-methyl-[1,1'-biphenyl]-2-yl)oxy)-3,5-dimethylaniline (**1o**)

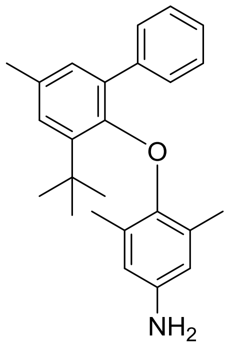

1o was prepared with 0.91 mmol scale according to the method **A** as white foam. (124.2 mg, 38% yield for two steps)

^{1}H NMR (500 MHz, Chloroform-*d*) δ 7.21 (d, *J* = 2.3 Hz, 1H), 7.16 – 7.10 (m, 2H), 7.10 – 7.00 (m, 3H), 6.82 (d, *J* = 2.4 Hz, 1H), 5.98 (s, 2H), 3.13 (s, 2H), 2.37 (s, 2H), 1.88 (s, 6H), 1.54 (s, 9H). ^{13}C NMR (126 MHz, Chloroform-*d*) δ 151.9, 146.4, 140.3, 139.7, 139.4, 132.5, 130.8, 130.4, 128.2, 128.0, 127.3, 127.1, 126.3, 116.0, 35.6, 30.6, 21.1, 18.0. m/z. HRMS (ESI) found $[M+H]^+$ 360.2316, $C_{25}H_{30}NO^+$ requires 360.2322.

4-((3-(tert-butyl)-5-methyl-3'-(trifluoromethyl)-[1,1'-biphenyl]-2-yl)oxy)-3,5-dimethylaniline (**1p**)

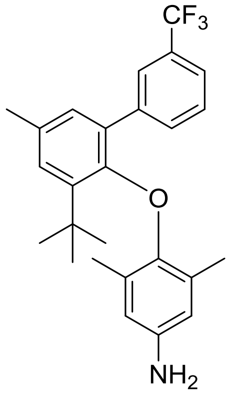

1p was prepared with 0.91 mmol scale according to the method **A** as yellow oil. (204.7 mg, 53% yield for two steps)

^{1}H NMR (500 MHz, Chloroform-*d*) δ 7.30 – 7.25 (m, 2H), 7.23 – 7.18 (m, 2H), 7.14 (t, *J* = 7.7 Hz, 1H), 6.72 (d, *J* = 2.3 Hz, 1H), 3.10 (s, 2H), 2.32 (s, 3H), 1.83 (s, 6H), 1.50 (d, *J* = 1.9 Hz, 9H). ^{13}C NMR (126 MHz, Chloroform-*d*) δ 152.1, 146.2, 141.1, 140.3, 139.7, 131.4, 130.8, 130.5, 130.3, 129.2 (q, *J* = 32.0 Hz), 128.3, 128.1, 127.6, 125.0 (q, *J* = 3.8 Hz), 124.3 (q, *J* = 272.5 Hz), 123.1 (q, *J* = 4.1 Hz), 121.1, 115.9, 35.6, 30.5, 21.0, 17.8. ^{19}F NMR (471 MHz, Chloroform-*d*) δ -62.6. m/z. HRMS (ESI) found $[M+H]^+$ 428.2192, $C_{26}H_{29}F_3NO^+$ requires 428.2196.

4-(2-(tert-butyl)-4-methyl-6-(naphthalen-1-yl)phenoxy)-3,5-dimethylaniline (**1q**)

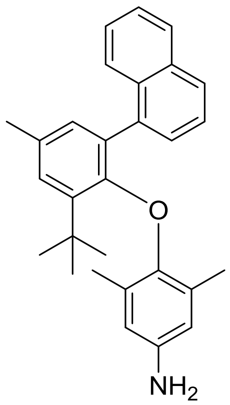

1q was prepared with 0.91 mmol scale according to the method **A** as a white solid. (203.4 mg, 55% yield for two steps)

^1H NMR (500 MHz, Chloroform-*d*) δ 7.80 (d, *J* = 8.1 Hz, 1H), 7.62 (d, *J* = 8.3 Hz, 2H), 7.45 (t, *J* = 7.4 Hz, 1H), 7.38 (dd, *J* = 7.5, 5.1 Hz, 2H), 7.21 (t, *J* = 7.6 Hz, 1H), 7.09 (d, *J* = 6.9 Hz, 1H), 6.93 (d, *J* = 2.3 Hz, 1H), 5.97 (d, *J* = 2.9 Hz, 1H), 5.61 (d, *J* = 2.9 Hz, 1H), 3.13 (s, 2H), 2.45 (s, 3H), 1.89 (s, 3H), 1.64 (s, 9H), 1.29 (s, 3H). ^{13}C NMR (126 MHz, Chloroform-*d*) δ 153.7, 146.8, 140.3, 139.2, 137.2, 133.0, 131.9, 130.9, 130.7, 130.1, 129.6, 127.9, 127.6, 127.5, 126.8, 126.7, 125.8, 125.3, 125.2, 124.8, 115.8, 115.7, 35.6, 30.6, 21.0, 18.4, 16.4. m/z. HRMS (ESI) found $[M+H]^+$ 410.2472, $C_{29}H_{32}NO^+$ requires 410.2479.

4-((3-(tert-butyl)-5-methyl-2',3',4',5'-tetrahydro-[1,1'-biphenyl]-2-yl)oxy)-3,5-dimethylaniline (**1r**)

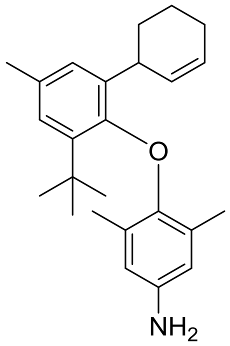

1r was prepared with 1.0 mmol scale according to the method **A** as white foam. (166.0 mg, 46% yield for two steps)

^1H NMR (500 MHz, Chloroform-*d*) δ 7.06 (d, *J* = 2.3 Hz, 1H), 6.69 (d, *J* = 2.3 Hz, 1H), 6.31 (s, 2H), 5.48 – 5.42 (m, 1H), 3.31 (s, 1H), 2.31 (s, 3H), 1.93 (s, 6H), 1.89 – 1.85 (m, 2H), 1.82 – 1.78 (m, 2H), 1.48 (s, 9H), 1.32 – 1.25 (m, 4H). ^{13}C NMR (126 MHz, Chloroform-*d*) δ 151.9, 146.9, 140.7, 139.2, 135.7, 134.5, 130.3, 128.6, 128.5, 126.3, 125.1, 116.3, 35.5, 30.6, 29.2, 25.4, 22.6, 21.8, 21.0, 18.1. m/z. HRMS (ESI) found $[M+H]^+$ 364.2627, $C_{25}H_{34}NO^+$ requires 364.2635.

4-(2-(tert-butyl)-6-methylphenoxy)-3,5-dimethylaniline (**1s**)

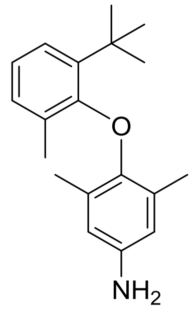

1s was prepared with 2.8 mmol scale according to the method **A** as white foa m. (397.5 mg, 50% yield for two steps)

^{1}H NMR (500 MHz, Chloroform-*d*) δ 7.25 – 7.19 (m, 1H), 6.94 – 6.87 (m, 2H), 6.36 (s, 2H), 3.20 (s, 2H), 1.98 (s, 6H), 1.76 (s, 2H), 1.47 (s, 9H). ^{13}C NMR (126 MHz, Chloroform-*d*) δ 154.9, 146.5, 141.0, 139.4, 130.2, 128.6, 127.5, 125.1, 122.0, 116.2, 35.5, 30.6, 18.2, 18.0. m/z HRMS (ESI) found $[M+H]^+$ 284.2009, $C_{19}H_{26}NO^+$ requires 284.2009.

4-((5'-(tert-butyl)-[1,1':3',1''-terphenyl]-4'-yl)oxy)-3,5-dimethylaniline (**1t**)

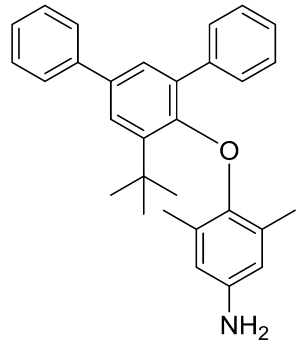

1t was prepared with 2.1 mmol scale according to the method **A** as white foa m. (461.9 mg, 78% yield for two steps)

^{1}H NMR (500 MHz, Chloroform-*d*) δ 7.7 – 7.6 (m, 3H), 7.4 (t, *J* = 7.6 Hz, 2H), 7.3 (t, *J* = 7.4 Hz, 1H), 7.2 (d, *J* = 2.3 Hz, 1H), 7.2 – 7.1 (m, 2H), 7.1 – 7.0 (m, 3H), 6.0 (s, 2H), 3.1 (s, 2H), 1.9 (s, 6H), 1.6 (s, 9H). ^{13}C NMR (126 MHz, Chloroform-*d*) δ 153.8,

146.2, 141.2, 140.7, 140.3, 139.3, 134.4, 132.9, 128.8, 128.7, 128.3, 128.2, 127.2, 127.0, 126.9, 126.5, 125.4, 116.0, 35.9, 30.6, 18.1. HRMS (ESI) found $[M+H]^+$ 422.2486, $C_{30}H_{32}NO^+$ requires 422.2479.

4-((5'-(tert-butyl)-4,4''-dimethoxy-[1,1':3',1''-terphenyl]-4'-yl)oxy)-3,5-dimethylaniline (**1u**)

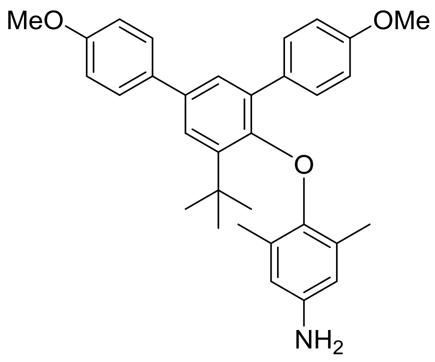

1u was prepared with 0.8 mmol scale according to the method **A** as white foa m. (267.7 mg, 69% yield for two steps)

^{1}H NMR (500 MHz, Chloroform-*d*) δ 7.58 (d, *J* = 2.5 Hz, 1H), 7.57 – 7.53 (m, 2H), 7.18 (d, *J* = 2.4 Hz, 1H), 7.11 – 7.04 (m, 2H), 7.01 – 6.94 (m, 2H), 6.67 – 6.59 (m, 2H), 6.00 (s, 2H), 3.85 (s, 3H), 3.74 (s, 3H), 2.86 (s, 2H), 1.90 (s, 6H), 1.57 (s, 9H). ^{13}C NMR (126 MHz, Chloroform-*d*) δ 158.9, 158.4, 153.4, 146.2, 140.6, 140.2, 134.0, 133.8, 132.6, 132.0, 129.3, 128.3, 128.0, 124.7, 116.0, 114.2, 112.8, 55.5, 35.8, 30.6, 18.1. m/z. HRMS (ESI) found $[M+H]^+$ 482.2699, $C_{32}H_{36}NO_3^+$ requires 482.2690.

4-(2-(tert-butyl)-4,6-dimethylphenoxy)-3,5-diethylaniline (**1v**)

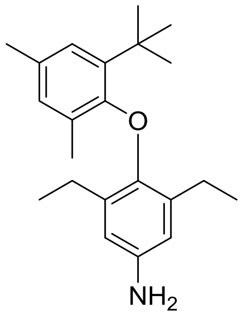

1v was prepared with 0.5 mmol scale according to the method **B** as a gray so lid (48.0 mg, 30% yield for two steps).

^{1}H NMR (500 MHz, Chloroform-*d*) δ 7.04 (d, *J* = 2.4 Hz, 1H), 6.69 (d, *J* = 2.8 Hz, 1H), 6.45 (s, 2H), 3.39 (s, 2H), 2.53 (dq, *J* = 15.0, 7.5 Hz, 2H), 2.33 (dq, *J* = 15.0, 7.5 Hz, 2H), 2.29 (s, 3H), 1.71 (s, 3H), 1.47 (s, 9H), 1.11 (t, *J* = 7.5 Hz, 6H). ^{13}C NMR (126 MHz, Chloroform-*d*) δ 153.3, 145.8, 141.5, 138.5, 135.2, 131.4, 130.5, 126.0, 125.8, 113.9, 35.4, 30.5, 23.2, 20.9, 18.7, 14.2. m/z. HRMS (ESI) found $[M+H]^+$ 326.2475, $C_{22}H_{32}NO^+$ requires 326.2479.

2'-(2-(tert-butyl)-4,6-dimethylphenoxy)-[1,1':3',1''-terphenyl]-5'-amine (**1w**)

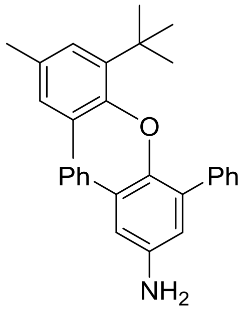

1w was prepared with 1.0 mmol scale according to the method **C** as a white solid (256.4 mg, 61% yield for two steps).

^{1}H NMR (500 MHz, Chloroform-*d*) δ 7.26 – 7.21 (m, 4H), 7.19 – 7.11 (m, 6H), 6.61 (s, 3H), 6.37 (d, *J* = 2.3 Hz, 1H), 3.56 (s, 2H), 2.08 (s, 3H), 1.83 (s, 3H), 1.08 (s, 9H). ^{13}C NMR (126 MHz, Chloroform-*d*) δ 153.4, 144.7, 140.6, 139.2, 138.7, 134.7, 130.8, 130.7, 129.8, 127.3, 126.6, 126.4, 125.4, 118.4, 35.0, 30.7, 20.7, 19.2. m/z. HRMS

(ESI) found $[M+H]^+$ 422.2482, $C_{30}H_{32}NO^+$ requires 422.2479.

2'-(2-(tert-butyl)-4,6-dimethylphenoxy)-4,4''-dimethoxy-[1,1':3',1''-terphenyl]-5'-amine (**1x**)

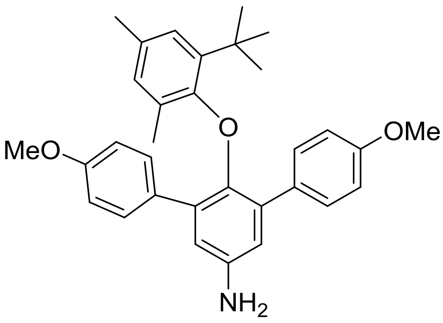

1x was prepared with 0.5 mmol scale according to the method **C** as a white solid (210 mg, 87% yield for two steps).

^{1}H NMR (500 MHz, Chloroform-*d*) δ 7.20 – 7.15 (m, 4H), 6.75 – 6.70 (m, 4H), 6.68 (s, 1H), 6.58 (s, 2H), 6.43 (s, 1H), 3.81 – 3.75 (m, 6H), 3.42 (s, 2H), 2.14 (s, 3H), 1.83 (s, 3H), 1.13 (s, 9H). ^{13}C NMR (126 MHz, Chloroform-*d*) δ158.5, 153.5, 144.8, 140.9, 138.6, 134.4, 131.8, 130.8, 130.8, 130.6, 126.2, 125.5, 118.0, 112.8, 55.3, 35.0, 30.7, 20.7, 19.2. m/z. HRMS (ESI) found $[M+H]^+$ 482.2683, $C_{32}H_{36}NO_3^+$ requires 482.2690.

2'-(2-(tert-butyl)-4,6-dimethylphenoxy)-2,2''-dimethoxy-[1,1':3',1''-terphenyl]-5'-amine (**1y**)

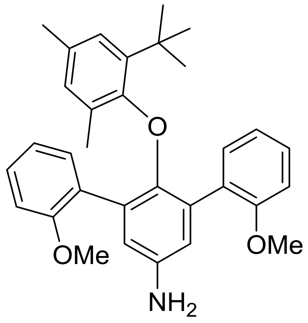

1y was prepared with 0.5 mmol scale according to the method **C** as a white s olid (234.7 mg, 98% yield for two steps).

^{1}H NMR (500 MHz, Chloroform-*d*) δ 7.14 (t, *J* = 7.8 Hz, 2H), 7.07 (d, *J* = 7.4 Hz, 2H), 6.73 (q, *J* = 7.0 Hz, 4H), 6.55 (d, *J* = 1.4 Hz, 2H), 6.48 (s, 1H), 6.41 (s, 1H), 3.73 (s, 6H), 3.48 (s, 2H), 2.08 (s, 3H), 1.87 (s, 3H), 1.10 (d, *J* = 1.6 Hz, 9H). ^{13}C NMR (126 MHz, Chloroform-*d*) δ 156.7, 153.3, 146.4, 140.2, 138.6, 131.7, 130.6, 130.3, 130.0, 128.2, 127.7, 124.6, 119.3, 119.0, 109.6, 54.9, 34.9, 30.4, 20.7, 18.9. m/z HRMS (ESI) found $[M+H]^+$ 482.2684, $C_{32}H_{36}NO_3^+$ requires 482.2690.

2'-(2-(tert-butyl)-4,6-dimethylphenoxy)-2,2''-difluoro-[1,1':3',1''-terphenyl]-5'-amine (**1z**)

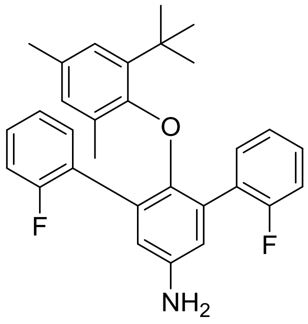

1z was prepared with 0.5 mmol scale according to the method **C** as a white s olid (224.7 mg, 98% yield for two steps).

^{1}H NMR (500 MHz, Chloroform-*d*) δ 7.18 – 7.06 (m, 4H), 6.96 – 6.86 (m, 4H), 6.62 (s, 2H), 6.52 (d, *J* = 2.3 Hz, 1H), 6.40 (d, *J* = 2.8 Hz, 1H), 3.57 (s, 2H), 2.05 (s, 3H), 1.83 (s, 3H), 1.10 (s, 9H). ^{13}C NMR (126 MHz, Chloroform-*d*) δ 159.8 (d, *J* = 246.4 Hz), 152.7, 145.9, 140.2, 139.1, 132.2 (d, *J* = 2.8 Hz), 131.4, 130.3, 128.8 (d, *J* = 7.9 Hz), 127.9, 127.7, 126.7 (d, *J* = 15.8 Hz), 125.1, 123.0 (d, *J* = 3.6 Hz), 119.3, 115.1 (d, *J* = 22.5 Hz), 35.0, 30.6, 20.7, 18.5. ^{19}F NMR (471 MHz, Chloroform-*d*) δ -112.1. m/z HRMS (ESI) found $[M+H]^+$ 458.2286, $C_{30}H_{30}F_2NO^+$ requires 458.2290.

4-(2-(tert-butyl)-4,6-dimethylphenoxy)-3,5-di((E)-styryl)aniline (**1aa**)

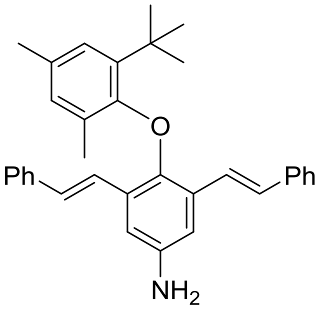

1aa was prepared with 0.5 mmol scale according to the method **D** as yellow f oam (81.0 mg, 32% yield for two steps).

^{1}H NMR (500 MHz, Chloroform-*d*) δ 7.31 – 7.26 (m, 4H), 7.24 – 7.19 (m, 7H), 7.17 (s, 2H), 6.93 (s, 2H), 6.87 (d, *J* = 16.2 Hz, 2H), 6.72 (d, *J* = 2.1 Hz, 1H), 3.56 (s, 2H), 2.28 (s, 3H), 1.81 (s, 3H), 1.51 (s, 9H). ^{13}C NMR (126 MHz, Chloroform-*d*) δ 153.3, 145.5, 141.1, 139.5, 137.8, 132.3, 131.2, 129.2, 129.1, 128.6, 127.7, 127.5, 126.6, 125.9, 124.4, 113.3, 35.4, 30.6, 21.0, 18.2. m/z HRMS (ESI) found $[M+H]^+$ 474.2790, $C_{34}H_{36}NO^+$ requires 474.2792.

3,5-dibromo-4-(2-(tert-butyl)-4,6-dimethylphenoxy)aniline (**1ab**)

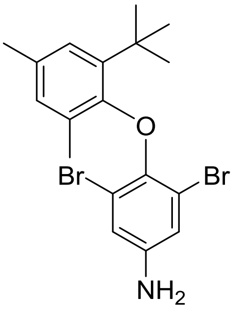

1ab was prepared with 3.13 mmol scale according to the method **E** as a yello w solid (740 mg, 55% yield).

^{1}H NMR (400 MHz, Chloroform-*d*) δ 7.1 (d, *J* = 2.2 Hz, 1H), 6.9 (s, 2H), 6.7 (d, *J* = 2.3 Hz, 1H), 3.6 (s, 2H), 2.3 (s, 3H), 1.8 (s, 3H), 1.5 (s, 9H). ^{13}C NMR (101 MHz,

Chloroform-*d*) δ 151.1, 143.0, 142.7, 139.4, 132.0, 130.4, 126.9, 126.1, 119.6, 114.7, 35.3, 30.7, 21.1, 18.3. m/z HRMS (ESI) found $[M+H]^+$ 428.0048, $C_{18}H_{22}Br_2NO^+$ requires 428.0043.

2'-(2-cyclohexyl-4,6-dimethylphenoxy)-[1,1':3',1''-terphenyl]-5'-amine (**1ac**)

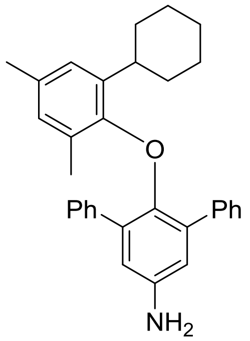

^{1}H NMR (500 MHz, Chloroform-*d*) δ 7.4 – 7.3 (m, 4H), 7.2 – 7.1 (m, 6H), 6.6 (s, 2H), 6.4 (d, *J* = 2.4 Hz, 1H), 6.3 (d, *J* = 2.3 Hz, 1H), 3.5 (s, 2H), 2.7 (tt, *J* = 11.9, 3.0 Hz, 1H), 2.1 (s, 3H), 1.8 (dt, *J* = 13.1, 3.2 Hz, 2H), 1.8 (d, *J* = 13.0 Hz, 1H), 1.7 (s, 3H), 1.7 – 1.6 (m, 2H), 1.5 – 1.4 (m, 2H), 1.2 (tt, *J* = 13.0, 3.6 Hz, 1H), 1.2 – 1.1 (m, 2H). ^{13}C NMR (126 MHz, Chloroform-*d*) δ 150.9, 145.4, 141.1, 138.9, 137.5, 134.6, 131.1, 129.6, 129.3, 127.5, 126.8, 126.1, 124.1, 117.4, 37.8, 33.4, 27.3, 26.6, 20.6, 18.2. m/z. HRMS (ESI) found $[M+H]^+$ 448.2637, $C_{32}H_{34}NO^+$ requires 448.2635.

Enantioselective synthesis of axially chiral diaryl ethers

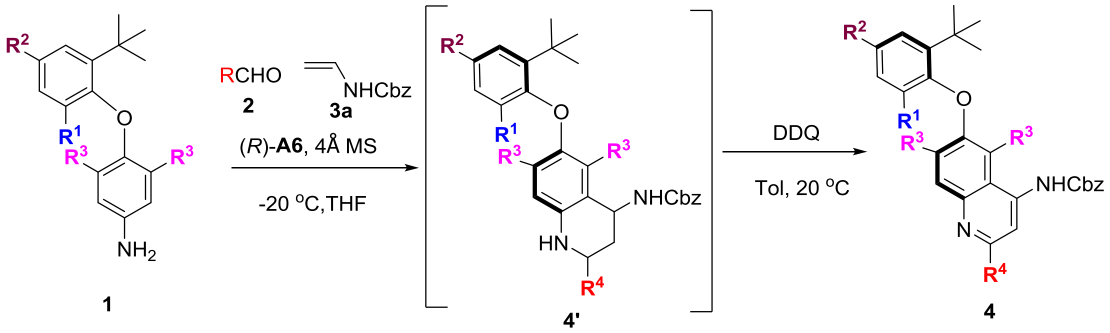

General Procedure for asymmetric synthesis of product **4**:

To a stirred solution of **1** (0.1 mmol, 1.0 equiv.), CPA (*R*)-**A6** (10 mol%) and activated 4Å molecular sieves (ca. 50mg) in THF (0.5 mL) was added the corresponding aldehyde **2** (0.15 mmol, 1.5 equiv.) at room temperature and stirred at -20 °C for 30 min. Following that, a solution of enamide **3a** (0.2 mmol, 2.0 equiv.) in THF (0.5 mL) was added dropwise into the reaction mixture via syringe and the mixture was allowed to stir at the same temperature for another 12 h. After completion of the Povarov reaction as monitored by TLC analysis, the product **4'** was afforded by flash column chromatography (petroleum ether/EtOAc = 9:1).

To a solution of the above product **4'** in toluene (1 mL) was added 1,2-dichloro-4,5-dicyanobenzoquinone (DDQ, 0.21 mmol, 2.1 equiv.) in portions at rt and the reaction mixture was allowed to stir for 2 h. The reaction was then diluted with EtOAc, and wash with saturated aqueous Na_2SO_3 for two times and saturated aqueous $NaHCO_3$ solution for two times. The organic layer was dried over Na_2SO_4 and concentrated under vacuum to give a residue, which was purified by flash column chromatography (petroleum ether/EtOAc = 12:1) to give the product **4**.

General procedure for the one-pot asymmetric synthesis of product **4a**:

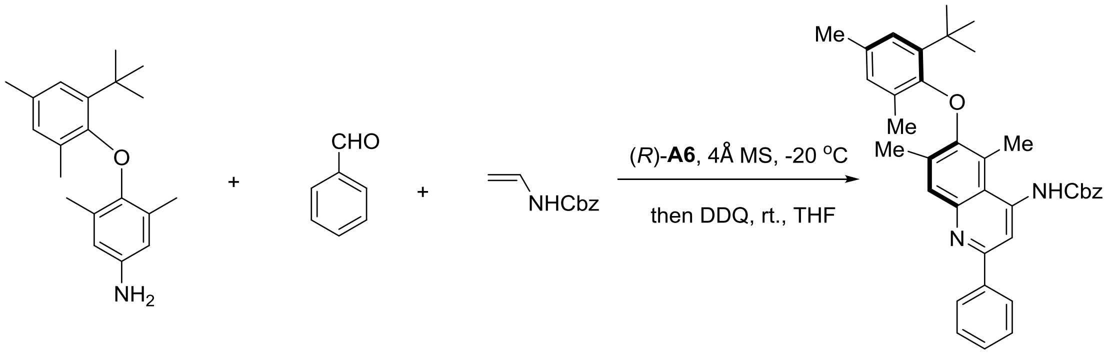

1a 2a 3 4a

To a stirred solution of **1a** (0.1 mmol, 1.0 equiv.), CPA (*R*)-**A6** (0.1 equiv.) and activated 4Å molecular sieves (ca. 50mg) in THF (0.5 mL) was added **2a** (0.15 mmol, 1.5 equiv.) at room temperature and stirred at -20 °C for 30 min. Following that, a solution of enamide **3** (0.2 mmol, 2.0 equiv.) in THF (0.5 mL) was added dropwise into the reaction mixture via syringe and the mixture was allowed to stir at the same temperature for another 12 h. After the completion of the Povarov reaction as monitored by TLC analysis, the reaction mixture was allowed to warm to rt. Then 1,2-dichloro-4,5-dicyanobenzoquinone (DDQ, 0.21 mmol, 2.1 equiv.) was added in portions at rt and the reaction mixture was allowed to stir for 2 h. The mixture was then diluted with EtOAc, and wash with saturated aqueous Na_2SO_3 for two times and saturated aqueous $NaHCO_3$ solution for two times. The organic layer was dried over Na_2SO_4 and concentrated under vacuum to give a residue, which was purified by flash column chromatography (petroleum ether/EtOAc = 12:1) to give the product **4a** as white foam (34.8 mg, 62.4% yield, 93% ee).

benzyl-((*2S,4S*)-6-(2-(tert-butyl)-4,6-dimethylphenoxy)-5,7-dimethyl-2-phenyl-1,2,3,4-tetrahydroquinolin-4-yl)carbamate (**4a'**)

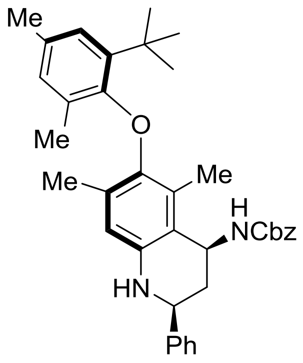

The intermediate **4a'** could be afforded on 0.1 mmol scale by flash column chromatography (petroleum ether : EtOAc = 9:1) as white foam. (56.2 mg, 99% yield).

^{1}H NMR (500 MHz, Chloroform-*d*) δ 7.46 – 7.26 (m, 7H), 7.20 (dd, *J* = 12.2, 7.1 Hz, 3H), 7.02 (d, *J* = 2.4 Hz, 1H), 6.69 (d, *J* = 2.4 Hz, 1H), 6.29 (s, 1H), 5.14 – 4.86 (m, 3H), 4.62 (t, *J* = 5.0 Hz, 1H), 4.19 (d, *J* = 8.4 Hz, 1H), 4.13 (s, 1H), 2.60 – 2.43 (m, 2H), 2.27 (s, 3H), 2.07 (d, *J* = 3.2 Hz, 3H), 1.87 (s, 3H), 1.68 (d, *J* = 4.9 Hz, 3H), 1.45 (d, *J* = 3.5 Hz, 9H). ^{13}C NMR (126 MHz, Chloroform-*d*) δ 155.1, 152.6, 146.2, 144.6, 140.2, 138.6, 136.8, 131.0, 130.7, 129.3, 128.9, 128.5, 128.0, 127.8, 127.1, 126.2, 126.0, 125.8, 117.3, 114.7, 66.3, 52.6, 45.1, 36.8, 35.4, 30.6, 21.0, 18.5, 18.2, 13.0. m/z HRMS (ESI) found $[M+H]^+$ 526.3265, $C_{37}H_{43}N_2O_3^+$ requires 563.3269. $[\alpha]_D^{23}$ = +31.1 (c 1.0, $CHCl_3$). HPLC: Chiralpak IB N-5 column, 80:20 hexane/isopropanol, 1 mL/min; t_R= 8.27 min (major); 17.27 min (minor); 98% ee; 98:2 dr.

benzyl (6-(2-(tert-butyl)-4,6-dimethylphenoxy)-5,7-dimethyl-2-phenylquinolin-4-yl)carbamate (**4a**)

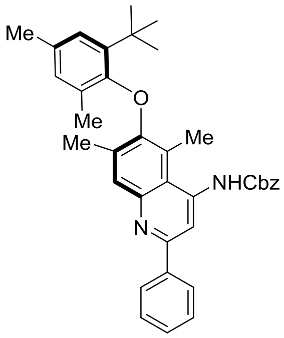

The reaction was performed on 0.1 mmol scale and **4a** was afforded as white foam (44.6 mg, 80% yield).

^{1}H NMR (400 MHz, Chloroform-*d*) δ 8.47 (s, 1H), 8.15 (d, J = 7.1 Hz, 2H), 7.83 (s, 1H), 7.70 (s, 1H), 7.57 – 7.33 (m, 8H), 7.09 (d, J = 2.3 Hz, 1H), 6.74 (d, J = 2.2 Hz, 1H), 5.29 (s, 2H), 2.73 (s, 3H), 2.31 (s, 3H), 2.18 (s, 3H), 1.64 (s, 3H), 1.50 (s, 9H). ^{13}C NMR (101 MHz, Chloroform-*d*) δ 156.0, 153.1, 152.8, 152.3, 147.0, 142.5, 139.4, 138.7, 135.8, 133.0, 131.9, 131.3, 130.9, 129.3, 128.8, 128.7, 128.6, 127.5, 126.4, 126.4, 126.4, 119.6, 118.6, 110.0, 67.7, 35.5, 30.7, 21.0, 19.1, 18.5, 16.5. m/z HRMS (ESI) found $[M+H]^+$ 559.2969, $C_{37}H_{39}N_2O_3^+$ requires 559.2956. $[\alpha]_D^{22}$ = +80.1 (c 1.0, $CHCl_3$). HPLC: Chiralpak ID column, 90:10 hexane/isopropanol, 1 mL/min; t_R= 6.43 min (minor); 6.96 min (major); 95% ee.

benzyl (6-(2-(tert-butyl)-4,6-dimethylphenoxy)-5,7-dimethyl-2-(4-nitrophenyl)quinolin-4-yl)carbamate (**4b**)

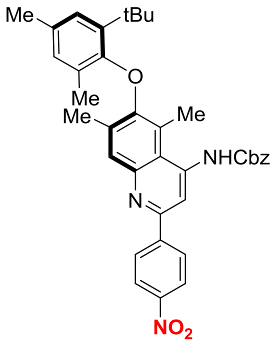

The reaction was performed on 0.1 mmol scale and **4b** was afforded as yellow foam (43.3 mg, 72% yield).

^{1}H NMR (500 MHz, Chloroform-*d*) δ 8.6 (s, 1H), 8.3 (s, 4H), 7.8 (s, 1H), 7.8 (s, 1H), 7.5 – 7.3 (m, 5H), 7.1 (d, *J* = 2.3 Hz, 1H), 6.7 (d, *J* = 2.2 Hz, 1H), 5.3 (d, *J* = 1.4 Hz, 2H), 2.7 (s, 3H), 2.3 (s, 3H), 2.2 (s, 3H), 1.6 (s, 3H), 1.5 (s, 9H). ^{13}C NMR (126 MHz, Chloroform-*d*) δ 153.5, 153.1, 153.0, 152.2, 148.3, 147.0, 145.4, 143.0, 138.8, 135.6, 133.6, 132.2, 131.6, 130.9, 128.9, 128.8, 128.6, 128.1, 126.5, 126.4, 124.0, 119.8, 118.5, 109.3, 67.9, 35.5, 30.7, 21.0, 19.2, 18.4, 16.5. m/z HRMS (ESI) found $[M+H]^+$ 604.2818, $C_{37}H_{38}N_3O_5^+$ requires 604.2806. $[\alpha]_D^{22}$ = +74.5 (c 0.5, $CHCl_3$). HPLC: Chiralpak IA column, 90:10 hexane/isopropanol, 1 mL/min; t_R= 11.85 min (minor); 17.42 min (major); 95% ee.

benzyl (6-(2-(tert-butyl)-4,6-dimethylphenoxy)-2-(4-cyanophenyl)-5,7-dimethylquinolin-4-yl)carbamate (**4c**)

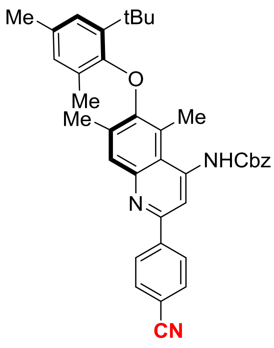

The reaction was performed on 0.1 mmol scale and **4c** was afforded as white foam (46.7 mg, 80% yield).

^{1}H NMR (500 MHz, Chloroform-*d*) δ 8.5 (s, 1H), 8.3 (d, *J* = 8.3 Hz, 2H), 7.8 – 7.7 (m, 4H), 7.5 – 7.3 (m, 5H), 7.1 (d, *J* = 2.2 Hz, 1H), 6.7 (d, *J* = 2.2 Hz, 1H), 5.3 (d, *J* = 1.5 Hz, 2H), 2.7 (s, 3H), 2.3 (s, 3H), 2.2 (s, 3H), 1.6 (s, 3H), 1.5 (s, 9H). ^{13}C NMR (126 MHz, Chloroform-*d*) δ 153.5, 153.3, 153.0, 152.1, 147.0, 143.6, 142.9, 138.7, 135.6, 133.5, 132.6, 132.2, 131.5, 130.8, 128.9, 128.8, 128.6, 127.9, 126.5, 126.4, 119.8,

119.1, 118.6, 112.5, 109.2, 67.9, 35.5, 30.7, 21.0, 19.2, 18.4, 16.5. m/z HRMS (ESI) found $[M+H]^+$ 584.2908, $C_{38}H_{38}N_3O_3^+$ requires 584.2908. $[\alpha]_D^{22}$ = +49.9 (c 1.0, $CHCl_3$). HPLC: Chiralpak IA column, 90:10 hexane/isopropanol, 1 mL/min; t_R= 11.12 min (minor); 15.86 min (major); 94% ee.

benzyl (6-(2-(tert-butyl)-4,6-dimethylphenoxy)-2-(4-methoxyphenyl)-5,7-dimethylquinolin-4-yl)carbamate (**4d**)

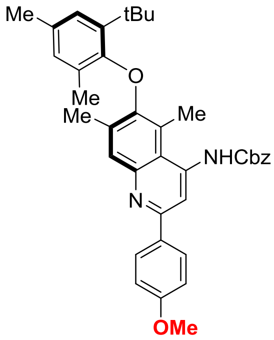

The reaction was performed on 0.1 mmol scale and **4d** was afforded as white foam (37.1 mg, 63% yield).

^{1}H NMR (500 MHz, Chloroform-*d*) δ 8.4 (s, 1H), 8.2 – 8.1 (m, 2H), 7.8 (s, 1H), 7.7 (s, 1H), 7.5 – 7.3 (m, 5H), 7.1 (d, *J* = 2.3 Hz, 1H), 7.1 – 7.0 (m, 2H), 6.8 – 6.7 (m, 1H), 5.3 (d, *J* = 2.2 Hz, 2H), 3.9 (s, 3H), 2.7 (s, 3H), 2.3 (s, 3H), 2.2 (s, 3H), 1.6 (s, 3H), 1.5 (s, 9H). ^{13}C NMR (126 MHz, Chloroform-*d*) δ 160.8, 155.6, 153.1, 152.5, 152.3, 147.1, 142.3, 138.7, 135.8, 132.9, 132.0, 131.8, 131.1, 130.9, 128.8, 128.7, 128.6, 128.5, 126.4, 126.3, 119.3, 118.7, 114.2, 109.7, 67.7, 55.5, 35.5, 30.7, 21.0, 19.1, 18.4, 16.4. m/z HRMS (ESI) found $[M+H]^+$ 683.3274, $C_{38}H_{41}N_2O_4^+$ requires 589.3061. $[\alpha]_D^{22}$ = +69.9 (c 1.0, $CHCl_3$). HPLC: Chiralpak IA column, 80:20 hexane/isopropanol, 1 mL/min; t_R= 6.32 min (minor); 9.95 min (major); 94% ee.

benzyl (6-(2-(tert-butyl)-4,6-dimethylphenoxy)-5,7-dimethyl-2-(3-(trifluoromethyl)p

henyl)quinolin-4-yl)carbamate (**4e**)

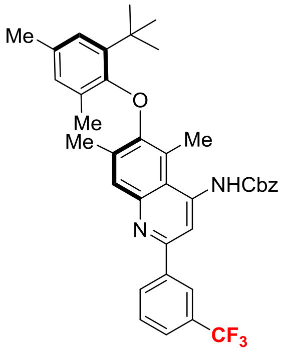

The reaction was performed on 0.1 mmol scale and **4e** was afforded as yellow syrup (48.4 mg, 77% yield).

^{1}H NMR (500 MHz, Chloroform-*d*) δ 8.5 (d, *J* = 3.7 Hz, 2H), 8.3 (d, *J* = 7.8 Hz, 1H), 7.9 (s, 1H), 7.8 (s, 1H), 7.7 (d, *J* = 7.8 Hz, 1H), 7.6 (t, *J* = 7.8 Hz, 1H), 7.5 – 7.3 (m, 5H), 7.1 (d, *J* = 2.3 Hz, 1H), 6.8 (d, *J* = 2.2 Hz, 1H), 5.3 (d, *J* = 1.7 Hz, 2H), 2.8 (s, 3H), 2.3 (s, 3H), 2.2 (s, 3H), 1.7 (s, 3H), 1.5 (s, 9H). ^{13}C NMR (126 MHz, Chloroform-*d*) δ 154.2, 153.1, 153.0, 152.2, 147.0, 142.8, 140.2, 138.7, 135.7, 133.3, 132.1, 131.4, 131.3 (q, *J* = 32.3 Hz), 130.9, 130.6, 129.2, 128.9, 128.7, 128.6, 126.5, 126.4, 125.8 (q, *J* = 3.1 Hz), 124.4 (q, *J* = 272.6 Hz), 124.3 (d, *J* = 4.0 Hz), 119.7, 118.6, 109.4, 67.8, 35.5, 30.7, 21.0, 19.1, 18.4, 16.5. ^{19}F NMR (471 MHz, Chloroform-*d*) δ -62.4. m/z HRMS (ESI) found $[M+H]^{+}$ 627.2828, $C_{38}H_{38}F_3N_2O_3^{+}$ requires 627.2830. $[\alpha]_D^{22}$ = +57.7 (c 1.0, $CHCl_3$). HPLC: Chiralpak IB-N column, 90:10 hexane/isopropanol, 1 mL/min; t_R= 6.15 min (major); 6.71 min (minor); 93% ee.

benzyl (6-(2-(tert-butyl)-4,6-dimethylphenoxy)-5,7-dimethyl-2-(o-tolyl)quinolin-4-yl) carbamate (**4f**)

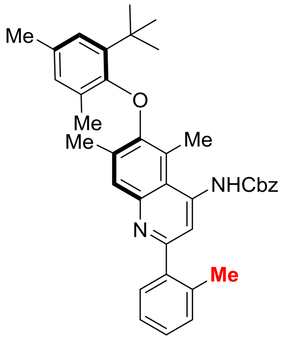

The reaction was performed on 0.1 mmol scale and **4f** was afforded as white foam (29.7 mg, 52% yield).

^{1}H NMR (500 MHz, Chloroform-*d*) δ 8.1 (s, 1H), 7.8 (s, 1H), 7.7 (s, 1H), 7.5 (dd, *J* = 7.5, 2.0 Hz, 1H), 7.5 – 7.3 (m, 5H), 7.3 – 7.3 (m, 3H), 7.1 (d, *J* = 2.3 Hz, 1H), 6.8 (d, *J* = 2.2 Hz, 1H), 5.3 (d, *J* = 2.9 Hz, 2H), 2.7 (s, 3H), 2.5 (s, 3H), 2.3 (s, 3H), 2.2 (s, 3H), 1.7 (s, 3H), 1.5 (s, 9H). ^{13}C NMR (126 MHz, Chloroform-*d*) δ 158.8, 153.0, 152.8, 152.3, 146.7, 141.7, 140.5, 138.7, 136.2, 135.8, 132.9, 132.0, 131.2, 130.9, 130.9, 129.8, 128.8, 128.7, 128.6, 128.5, 126.4, 126.0, 119.2, 118.5, 113.7, 67.7, 35.5, 30.7, 21.0, 20.6, 19.2, 18.5, 16.5. m/z HRMS (ESI) found $[M+H]^+$ 573.3111, $C_{38}H_{41}N_2O_3^+$ requires 573.3112. $[\alpha]_D^{22}$ = +72.6 (c 1.0, $CHCl_3$). HPLC: Chiralpak IC column, 90:10 hexane/isopropanol, 1 mL/min; t_R= 5.97 min (minor); 6.42 min (major); 91% ee.

benzyl (2-(2-bromophenyl)-6-(2-(tert-butyl)-4,6-dimethylphenoxy)-5,7-dimethylquinolin-4-yl)carbamate (**4g**)

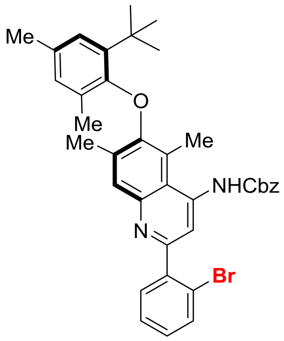

The reaction was performed on 0.1 mmol scale and **4g** was afforded as white foam (29.8 mg, 47% yield).

^{1}H NMR (400 MHz, Chloroform-*d*) δ 8.2 (s, 1H), 7.8 (s, 1H), 7.7 (dt, *J* = 7.5, 1.4 Hz, 2H), 7.6 (dt, *J* = 7.7, 1.6 Hz, 1H), 7.5 – 7.3 (m, 6H), 7.3 – 7.2 (m, 1H), 7.1 (d, *J* = 2.4 Hz, 1H), 6.8 (d, *J* = 2.3 Hz, 1H), 5.3 (d, *J* = 2.1 Hz, 2H), 2.7 (s, 3H), 2.3 (s, 3H), 2.2 (s, 3H), 1.7 (s, 3H), 1.5 (s, 9H). ^{13}C NMR (101 MHz, Chloroform-*d*) δ 157.4, 153.1, 152.9, 152.2, 146.6, 141.5, 138.7, 135.7, 133.4, 133.1, 132.1, 131.5, 131.3, 130.8, 130.0, 128.8, 128.7, 128.6, 127.6, 126.5, 126.5, 122.1, 119.6, 118.6, 113.7, 67.8, 35.5, 30.7, 21.0, 19.2, 18.5, 16.5. m/z HRMS (ESI) found $[M+H]^+$ 637.2057, $C_{37}H_{38}BrN_2O_3^+$ requires 637.2061. $[\alpha]_D^{22}$ = +81.2 (c 0.5, $CHCl_3$). HPLC: Chiralpak IA column, 90:10 hexane/isopropanol, 1 mL/min; t_R= 6.04 min (major); 6.67 min (minor); 81% ee.

benzyl (6-(2-(tert-butyl)-4,6-dimethylphenoxy)-2-(3,4-dichlorophenyl)-5,7-dimethylquinolin-4-yl)carbamate (**4h**)

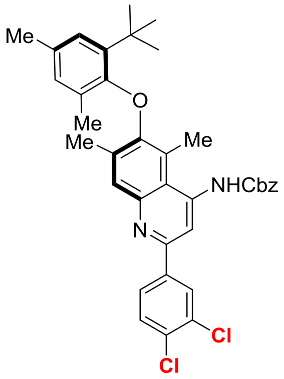

The reaction was performed on 0.1 mmol scale and **4h** was afforded as brown foam (44.9 mg, 72% yield).

^{1}H NMR (500 MHz, Chloroform-*d*) δ 8.4 (s, 1H), 8.3 (d, *J* = 2.0 Hz, 1H), 8.0 (dd, *J* = 8.4, 2.1 Hz, 1H), 7.8 (s, 1H), 7.7 (s, 1H), 7.5 (d, *J* = 8.4 Hz, 1H), 7.5 – 7.3 (m, 5H), 7.1 (d, *J* = 2.3 Hz, 1H), 6.7 (d, *J* = 2.6 Hz, 1H), 5.3 (d, *J* = 1.8 Hz, 2H), 2.7 (s, 3H),

2.3 (s, 3H), 2.2 (s, 3H), 1.6 (s, 3H), 1.5 (s, 9H). ^{13}C NMR (126 MHz, Chloroform-*d*) δ 153.2, 153.1, 153.0, 152.2, 146.9, 142.8, 139.3, 138.7, 135.7, 133.4, 133.1, 132.1, 131.4, 130.9, 130.7, 129.2, 128.9, 128.8, 128.6, 126.5, 126.5, 126.4, 119.6, 118.6, 109.0, 67.8, 35.5, 30.7, 21.0, 19.1, 18.4, 16.5. m/z HRMS (ESI) found $[M+H]^+$ 627.2178, $C_{37}H_{37}Cl_2N_2O_3^+$ requires 627.2176. $[\alpha]_D^{22}$ = +65.6 (c 1.0, $CHCl_3$). HPLC: Chiralpak IA column, 90:10 hexane/isopropanol, 1 mL/min; t_R= 5.88 min (minor); 6.80 min (major); 94% ee.

benzyl (6-(2-(tert-butyl)-4,6-dimethylphenoxy)-5,7-dimethyl-2-(naphthalen-2-yl)quinolin-4-yl)carbamate (**4i**)

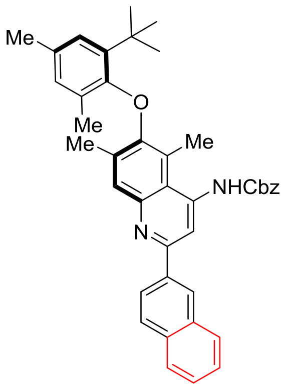

The reaction was performed on 0.1 mmol scale and **4i** was afforded as brown foam (32.8 mg, 54% yield).

^{1}H NMR (500 MHz, Chloroform-*d*) δ 8.6 (d, *J* = 3.9 Hz, 2H), 8.4 (dd, *J* = 8.6, 1.8 Hz, 1H), 8.0 – 8.0 (m, 1H), 8.0 (d, *J* = 8.6 Hz, 1H), 7.9 (t, *J* = 5.0 Hz, 2H), 7.7 (s, 1H), 7.6 – 7.5 (m, 2H), 7.5 (d, *J* = 7.0 Hz, 2H), 7.4 – 7.4 (m, 3H), 7.1 (d, *J* = 2.3 Hz, 1H), 6.8 (d, *J* = 2.2 Hz, 1H), 5.3 (d, *J* = 2.6 Hz, 2H), 2.8 (s, 3H), 2.3 (s, 3H), 2.2 (s, 3H), 1.7 (s, 3H), 1.5 (s, 9H). ^{13}C NMR (126 MHz, Chloroform-*d*) δ 155.8, 153.1, 152.8, 152.3, 147.1, 142.5, 138.7, 136.7, 135.8, 134.0, 133.6, 133.1, 131.9, 131.3, 130.9, 129.0, 128.8, 128.7, 128.5, 128.5, 127.8, 126.9, 126.7, 126.5, 126.4, 126.3, 125.0, 119.7, 118.7, 110.1, 67.7, 35.5, 30.7, 21.0, 19.1, 18.5, 16.5. m/z HRMS (ESI) found $[M+H]^+$

609.3123, $C_{41}H_{41}N_2O_3^+$ requires 609.3112. $[\alpha]_D^{22}$ = +35.8 (c 1.0, $CHCl_3$). HPLC: Chiralpak IA column, 90:10 hexane/ isopropanol, 1 mL/min; t_R= 7.73 min (minor); 10.07 min (major); 92% ee.

benzyl (6-(2-(tert-butyl)-4,6-dimethylphenoxy)-5,7-dimethyl-2-(naphthalen-1-yl)quinolin-4-yl)carbamate (**4j**)

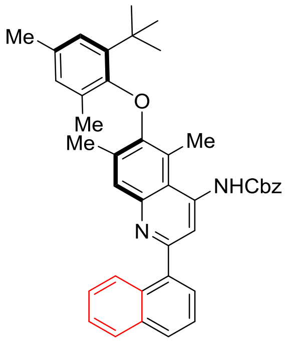

The reaction was performed on 0.1 mmol scale and **4j** was afforded as brown foam (29.5 mg, 49% yield).

^{1}H NMR (500 MHz, Chloroform-*d*) δ 8.3 (d, *J* = 10.1 Hz, 2H), 8.0 – 7.9 (m, 2H), 7.9 (s, 1H), 7.8 – 7.7 (m, 2H), 7.6 – 7.5 (m, 1H), 7.5 – 7.5 (m, 2H), 7.4 – 7.3 (m, 5H), 7.1 (d, *J* = 2.3 Hz, 1H), 6.8 (d, *J* = 2.3 Hz, 1H), 5.2 (d, *J* = 2.6 Hz, 2H), 2.8 (s, 3H), 2.3 (s, 3H), 2.2 (s, 3H), 1.7 (s, 3H), 1.5 (s, 9H). ^{13}C NMR (126 MHz, Chloroform-*d*) δ 158.0, 153.0, 153.0, 152.3, 146.8, 142.0, 138.8, 138.5, 135.8, 134.1, 133.1, 132.0, 131.4, 131.3, 130.9, 129.1, 128.8, 128.7, 128.7, 128.6, 128.4, 127.8, 126.6, 126.5, 126.0, 125.5, 119.6, 118.6, 114.6, 67.7, 35.5, 30.7, 21.0, 19.2, 18.6, 16.5. m/z HRMS (ESI) found [M+H]$^+$ 609.3116, $C_{41}H_{41}N_2O_3^+$ requires 609.3116. $[\alpha]_D^{22}$ = +62.5 (c 0.5, $CHCl_3$). HPLC: Chiralpak IB-N column, 80:20 hexane/isopropanol, 1 mL/min; t_R= 8.53 min (major); 9.76 min (minor); 88% ee.

benzyl (6-(2-(tert-butyl)-4,6-dimethylphenoxy)-5,7-dimethyl-2-propylquinolin-4-yl)c

arbamate (**4k**)

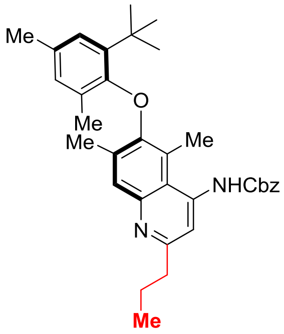

The reaction was performed on 0.1 mmol scale and **4k** was afforded as yellow foam (25.3 mg, 48% yield).

^{1}H NMR (400 MHz, Chloroform-*d*) δ 7.9 (s, 1H), 7.7 (s, 1H), 7.7 (s, 1H), 7.5 – 7.3 (m, 5H), 7.1 (d, *J* = 2.3 Hz, 1H), 6.7 (d, *J* = 2.2 Hz, 1H), 5.3 (s, 2H), 2.9 – 2.8 (m, 2H), 2.7 (s, 3H), 2.3 (s, 3H), 1.9 – 1.7 (m, 2H), 1.6 (s, 3H), 1.5 (s, 9H), 1.0 (t, *J* = 7.3 Hz, 3H). ^{13}C NMR (101 MHz, Chloroform-*d*) δ 161.5, 152.9, 152.4, 152.2, 138.7, 135.8, 132.9, 131.9, 130.9, 130.1, 128.8, 128.7, 128.6, 126.4, 126.3, 119.0, 118.6, 112.0, 67.7, 40.8, 35.5, 30.6, 23.3, 21.0, 19.1, 18.5, 16.5, 14.2. m/z HRMS (ESI) found $[M+H]^+$ 525.3118, $C_{34}H_{41}N_2O_3^+$ requires 525.3112. $[\alpha]_D^{22}$ = +62.5 (c 0.5, $CHCl_3$). HPLC: Chiralpak IB-N column, 90:10 hexane/isopropanol, 1 mL/min; t_R= 5.64 min (major); 6.35 min (minor); 85% ee.

benzyl (6-(2-(tert-butyl)-4,6-dimethylphenoxy)-2-isopropyl-5,7-dimethylquinolin-4-yl)carbamate (**4l**)

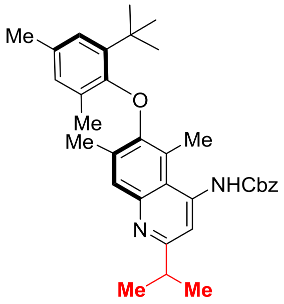

The reaction was performed on 0.1 mmol scale and **4l** was afforded as yellow syrup (23.2 mg, 44% yield).

^{1}H NMR (500 MHz, Chloroform-*d*) δ 7.9 (s, 1H), 7.7 (s, 1H), 7.6 (s, 1H), 7.5 – 7.3 (m, 5H), 7.1 (s, 1H), 6.7 (s, 1H), 5.3 (s, 2H), 3.2 (p, *J* = 7.1 Hz, 1H), 2.7 (s, 3H), 2.3 (s, 3H), 2.1 (s, 3H), 1.6 (s, 3H), 1.5 (s, 9H), 1.4 (d, *J* = 1.6 Hz, 3H), 1.4 (d, *J* = 1.6 Hz, 3H). ^{13}C NMR (126 MHz, Chloroform-*d*) δ 166.4, 153.0, 152.3, 152.3, 146.3, 142.2, 138.7, 135.8, 132.6, 131.8, 130.9, 130.5, 128.8, 128.7, 128.5, 126.4, 126.4, 119.3, 118.6, 110.2, 67.6, 37.0, 35.5, 30.6, 22.5, 21.0, 19.0, 18.5, 16.4. m/z HRMS (ESI) found $[M+H]^+$ 525.3116, $C_{34}H_{41}N_2O_3^+$ requires 525.3112. $[\alpha]_D^{22}$ = +77.1 (c 1.0, $CHCl_3$). HPLC: Chiralpak IB-N column, 90:10 hexane/isopropanol, 1 mL/min; t_R= 4.74 min (major); 5.10 min (minor); 80% ee.

benzyl (6-(2-(tert-butyl)-4,6-dimethylphenoxy)-2-cyclohexyl-5,7-dimethylquinolin-4-yl)carbamate (**4m**)

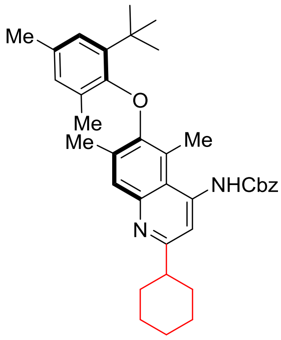

The reaction was performed on 0.1 mmol scale and **4m** was afforded as brown foam (30.8 mg, 55% yield).

^{1}H NMR (500 MHz, Chloroform-*d*) δ 7.9 (s, 1H), 7.7 (s, 1H), 7.6 (s, 1H), 7.5 – 7.3 (m, 5H), 7.1 (d, *J* = 2.3 Hz, 1H), 6.7 (d, *J* = 2.2 Hz, 1H), 5.3 (d, *J* = 1.4 Hz, 2H), 2.8 (tt, *J* = 12.2, 3.2 Hz, 1H), 2.7 (s, 3H), 2.3 (s, 3H), 2.1 (s, 3H), 2.0 (d, *J* = 12.6 Hz, 2H), 1.9 (dt, *J* = 12.9, 3.3 Hz, 2H), 1.8 (d, *J* = 12.7 Hz, 1H), 1.7 – 1.6 (m, 5H), 1.5 (s, 9H), 1.5 – 1.4 (m, 2H), 1.3 (tt, *J* = 12.8, 3.4 Hz, 1H). ^{13}C NMR (126 MHz, Chloroform-*d*) δ

165.6, 153.1, 152.3, 146.5, 142.1, 138.7, 135.8, 132.6, 131.8, 130.9, 130.5, 128.8, 128.7, 128.5, 126.4, 126.4, 119.3, 118.6, 110.6, 67.6, 47.3, 35.5, 32.8, 32.8, 30.6, 26.7, 26.2, 21.0, 19.1, 18.5, 16.4. m/z HRMS (ESI) found $[M+H]^+$ 565.3426, $C_{37}H_{45}N_2O_3^+$ requires 565.3426. $[\alpha]_D^{22}$ = +69.4 (c 1.0, $CHCl_3$). HPLC: Chiralpak IA column, 97:3 hexane/isopropanol, 1 mL/min; t_R= 7.12 min (minor); 7.68 min (major); 86% ee.

benzyl (6-((3-(tert-butyl)-5-methyl-[1,1'-biphenyl]-2-yl)oxy)-5,7-dimethyl-2-phenylquinolin-4-yl)carbamate (**4o**)

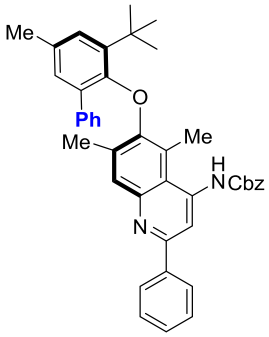

The reaction was performed on 0.1 mmol scale and **4o** was afforded as brown foam (39.7 mg, 64% yield).

^{1}H NMR (500 MHz, Chloroform-*d*) δ 8.35 (s, 1H), 8.11 (d, *J* = 7.2 Hz, 2H), 7.55 – 7.36 (m, 9H), 7.33 (s, 1H), 7.27 (d, *J* = 2.4 Hz, 1H), 7.03 – 6.98 (m, 2H), 6.86 – 6.77 (m, 3H), 6.72 – 6.67 (m, 1H), 5.35 – 5.26 (m, 2H), 2.46 (s, 3H), 2.37 (s, 3H), 2.18 (s, 3H), 1.57 (s, 9H). ^{13}C NMR (126 MHz, Chloroform-*d*) δ 155.4, 152.9, 152.4, 151.3, 146.7, 141.8, 139.4, 139.2, 139.1, 136.0, 132.6, 131.6, 131.5, 130.9, 130.6, 129.2, 128.9, 128.8, 128.7, 128.5, 128.0, 127.9, 127.4, 127.2, 126.8, 119.1, 118.1, 108.9, 67.6, 35.7, 30.7, 21.1, 19.0, 17.2. m/z HRMS (ESI) found $[M+H]^+$ 621.3100, $C_{42}H_{41}N_2O_3^+$ requires 621.3112. $[\alpha]_D^{22}$ = +177.6 (c 0.5, $CHCl_3$). HPLC: Chiralpak IC column, 90:10 hexane/isopropanol, 1 mL/min; t_R= 5.78 min (minor); 6.99 min (major); 96% ee.

benzyl (6-((3-(tert-butyl)-5-methyl-3'-(trifluoromethyl)-[1,1'-biphenyl]-2-yl)oxy)-5,7-dimethyl-2-phenylquinolin-4-yl)carbamate (**4p**)

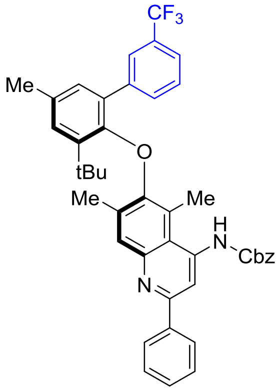

The reaction was performed on 0.1 mmol scale and **4p** was afforded as white foam (44.2 mg, 64% yield).

^{1}H NMR (500 MHz, Chloroform-*d*) δ 8.36 (s, 1H), 8.10 (d, *J* = 7.1 Hz, 2H), 7.54 – 7.35 (m, 10H), 7.31 (d, *J* = 2.5 Hz, 2H), 7.14 (d, *J* = 7.6 Hz, 1H), 6.98 (d, *J* = 7.9 Hz, 1H), 6.91 (t, *J* = 7.7 Hz, 1H), 6.78 (d, *J* = 2.2 Hz, 1H), 5.30 (s, 2H), 2.52 (s, 3H), 2.38 (s, 3H), 2.15 (s, 3H), 1.58 (s, 9H). ^{13}C NMR (126 MHz, Chloroform-*d*) δ 155.8, 153.0, 152.2, 151.4, 146.8, 141.9, 139.9, 139.5, 139.4, 136.0, 132.3, 131.9, 131.3, 131.3, 130.5, 129.9, 129.2, 129.2 (q, *J* = 31.9 Hz), 128.8, 128.8, 128.7, 128.6, 128.4, 128.0, 127.4, 125.1, 124.8 (q, *J* = 3.1 Hz), 124.0 (q, *J* = 272.6 Hz), 123.5 (q, *J* = 3.2 Hz), 119.0, 118.6, 109.5, 67.6, 35.8, 30.7, 21.0, 18.9, 16.7. ^{19}F NMR (471 MHz, Chloroform-*d*) δ -62.5. m/z HRMS (ESI) found $[M+H]^+$ 689.2979, $C_{43}H_{40}F_3N_2O_3^+$ requires 689.2986. $[\alpha]_D^{22}$ = +141.3 (c 1.0, $CHCl_3$). HPLC: Chiralpak IC column, 90:10 hexane/isopropanol, 1 mL/min; t_R= 5.29 min (minor); 6.06 min (major); 97% ee.

benzyl (6-(2-(tert-butyl)-4-methyl-6-(naphthalen-1-yl)phenoxy)-5,7-dimethyl-2-phenylquinolin-4-yl)carbamate (**4q**)

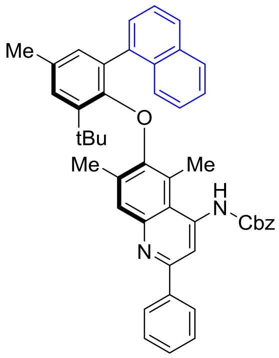

The reaction was performed on 0.1 mmol scale and **4q** was afforded as white foam (38.8 mg, 58% yield; dr = 4.7: 1).

^{1}H NMR (500 MHz, Chloroform-*d*) δ 8.31 (s, 0.2H, *minor diastereoisomer*), 8.27 (s, 0.8H, *major diastereoisomer*), 8.14 – 8.09 (m, 1.6H, *major diastereoisomer*), 8.07 – 8.04 (m, 0.4H, *minor diastereoisomer*), 7.62 (d, *J* = 8.4 Hz, 1H), 7.55 – 7.37 (m, 10H), 7.35 (d, *J* = 2.3 Hz, 1H), 7.34 – 7.28 (m, 1H), 7.21 – 7.12 (m, 2H), 7.06 (dd, *J* = 7.0, 1.2 Hz, 0.2H, *minor diastereoisomer*), 6.97 (s, 0.2H, *minor diastereoisomer*), 6.94 – 6.82 (m, 2H), 6.74 (dd, *J* = 8.2, 7.0 Hz, 0.8H, *major diastereoisomer*), 6.60 (s, 0.8H, *major diastereoisomer*), 5.41 – 5.19 (m, 2H), 2.44 (s, 0.5H, *minor diastereoisomer*), 2.40 (s, 3H), 2.13 (s, 2.5H, *major diastereoisomer*), 1.77 (s, 0.5H, *minor diastereoisomer*), 1.67 (s, 2.5H, *major diastereoisomer*), 1.60 (s, 9H). ^{13}C NMR (126 MHz, Chloroform-*d*) δ 155.5, 153.2, 152.8, 152.7, 146.4, 141.7, 139.5, 139.0, 137.0, 136.7, 136.0, 132.8, 132.0, 131.6, 131.5, 131.0, 130.5, 129.2, 129.0, 128.9, 128.8, 128.7, 128.6, 128.5, 128.3, 128.0, 127.9, 127.4, 126.8, 126.5, 126.4, 126.1, 125.8, 125.7, 125.1, 123.9, 120.2, 118.8, 118.4, 108.3, 67.7, 67.5, 35.9, 35.8, 30.8, 30.8, 21.1, 19.1, 18.3, 17.5, 15.7. m/z HRMS (ESI) found $[M+H]^+$ 671.3265, $C_{46}H_{43}N_2O_3^+$ requires 671.3269. $[\alpha]_D^{22}$ = +29.7 (c 1.0, $CHCl_3$). HPLC: Chiralpak IC column, 90:10 hexane/isopropanol, 1 mL/min; for the major diastereoisomer: t_R= 6.25 min (minor); 9.00 min (major); 97% ee; for the minor diastereoisomer: t_R= 7.98 min (major); >99% ee.

benzyl (6-((3-(tert-butyl)-5-methyl-2',3',4',5'-tetrahydro-[1,1'-biphenyl]-2-yl)oxy)-5,7-dimethyl-2-phenylquinolin-4-yl)carbamate (**4r**)

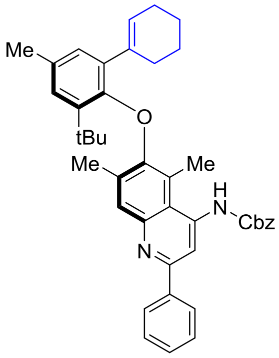

The reaction was performed on 0.1 mmol scale and **4r** was afforded as white foam (37.5 mg, 60% yield).

^{1}H NMR (500 MHz, Chloroform-*d*) δ 8.50 (s, 1H), 8.20 – 8.15 (m, 2H), 7.80 (s, 1H), 7.66 (s, 1H), 7.50 (t, *J* = 7.5 Hz, 2H), 7.47 – 7.33 (m, 6H), 7.13 (d, *J* = 2.3 Hz, 1H), 6.71 (d, *J* = 2.3 Hz, 1H), 5.48 – 5.43 (m, 1H), 5.28 (s, 2H), 2.57 (s, 3H), 2.32 (s, 3H), 2.20 (s, 3H), 1.79 (td, *J* = 19.2, 13.4 Hz, 2H), 1.64 – 1.53 (m, 2H), 1.51 (s, 9H), 0.96 – 0.83 (m, 4H). ^{13}C NMR (126 MHz, Chloroform-*d*) δ 155.7, 153.0, 151.4, 147.0, 142.2, 139.4, 138.9, 135.8, 135.7, 133.7, 133.3, 131.4, 131.2, 129.3, 128.8, 128.8, 128.7, 128.5, 127.5, 127.0, 126.1, 119.5, 117.9, 109.3, 67.7, 35.7, 30.7, 29.2, 25.3, 22.3, 21.4, 21.1, 19.1, 17.2. m/z HRMS (ESI) found $[M+H]^+$ 625.3425, $C_{42}H_{45}N_2O_3^+$ requires 625.3425. $[\alpha]_D^{22}$ = +161.4 (c 0.5, $CHCl_3$). HPLC: Chiralpak IC column, 90:10 hexane/isopropanol, 1 mL/min; t_R= 4.92 min (minor); 5.62 min (major); 98% ee.

benzyl (6-(2-(tert-butyl)-6-methylphenoxy)-5,7-dimethyl-2-phenylquinolin-4-yl)carbamate (**4s**)

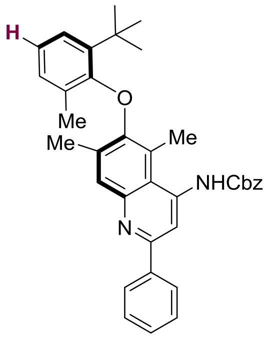

The reaction was performed on 0.1 mmol scale and **4s** was afforded as white foam (39.8 mg, 73% yield).

^{1}H NMR (500 MHz, Chloroform-*d*) δ 8.47 (s, 1H), 8.16 (d, *J* = 7.0 Hz, 2H), 7.85 (s, 1H), 7.69 (s, 1H), 7.50 (t, *J* = 7.5 Hz, 2H), 7.47 – 7.35 (m, 6H), 7.31 (dd, *J* = 7.9, 1.8 Hz, 1H), 6.98 (t, *J* = 7.6 Hz, 1H), 6.96 – 6.90 (m, 1H), 5.29 (d, *J* = 2.3 Hz, 2H), 2.74 (s, 3H), 2.19 (s, 3H), 1.69 (s, 3H), 1.52 (s, 9H). ^{13}C NMR (126 MHz, Chloroform-*d*) δ 156.1, 154.4, 153.1, 152.6, 147.1, 142.5, 139.4, 139.0, 135.8, 132.9, 131.4, 130.6, 129.3, 128.8, 128.7, 128.6, 127.4, 126.7, 125.7, 122.9, 119.6, 118.8, 110.2, 67.7, 35.6, 30.6, 19.0, 18.6, 16.4. m/z HRMS (ESI) found $[M+H]^+$ 545.2805, $C_{33}H_{37}N_2O_3^+$ requires 545.2799. $[\alpha]_D^{22}$ = +74.7 (c 1.0, $CHCl_3$). HPLC: Chiralpak IA column, 90:10 hexane/isopropanol, 1 mL/min; t_R= 5.95 min (minor); 7.63 min (major); 95% ee.

benzyl (6-((5'-(tert-butyl)-[1,1':3',1''-terphenyl]-4'-yl)oxy)-5,7-dimethyl-2-phenylquinolin-4-yl)carbamate (**4t**)

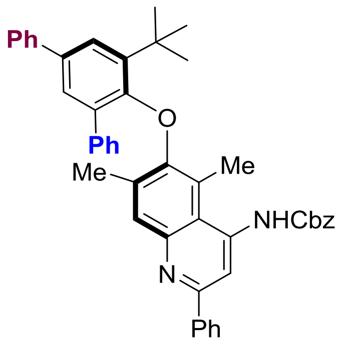

The reaction was performed on 0.1 mmol scale and **4t** was afforded as white foam (40.5 mg, 59% yield).

^{1}H NMR (500 MHz, Chloroform-*d*) δ 8.37 (s, 1H), 8.14 (d, *J* = 7.2 Hz, 2H), 7.74 (d, *J* = 2.4 Hz, 1H), 7.68 – 7.63 (m, 2H), 7.55 (s, 1H), 7.52 – 7.31 (m, 12H), 7.29 (d, *J* = 2.3 Hz, 1H), 7.08 (d, *J* = 7.2 Hz, 2H), 6.85 (t, *J* = 7.5 Hz, 2H), 6.73 (t, *J* = 7.5 Hz, 1H), 5.37 – 5.27 (m, 2H), 2.51 (s, 3H), 2.24 (s, 3H), 1.65 (s, 9H). ^{13}C NMR (126 MHz, Chloroform-*d*) δ 155.6, 153.1, 152.9, 152.3, 146.8, 141.9, 140.8, 139.8, 139.4, 139.0, 135.9, 135.1, 132.5, 132.0, 130.9, 129.2, 128.9, 128.8, 128.8, 128.7, 128.5, 128.1, 127.4, 127.2, 127.2, 127.0, 127.0, 126.0, 119.1, 118.4, 109.1, 67.6, 36.0, 30.7, 19.0, 17.3. m/z HRMS (ESI) found $[M+H]^+$ 683.3274, $C_{47}H_{43}N_2O_3^+$ requires 683.3269. $[\alpha]_D^{22}$ = +202.9 (c 1.0, $CHCl_3$). HPLC: Chiralpak IC column, 80:20 hexane/isopropanol, 1 mL/min; t_R= 4.82 min (minor); 5.34 min (major); 97% ee.

benzyl (6-((5'-(tert-butyl)-4,4''-dimethoxy-[1,1':3',1''-terphenyl]-4'-yl)oxy)-5,7-dimethyl-2-phenylquinolin-4-yl)carbamate (**4u**)

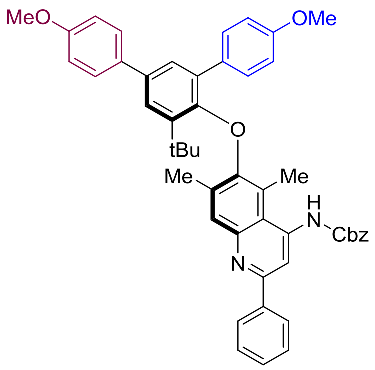

The reaction was performed on 0.1 mmol scale and **4u** was afforded as pink foam (50.9 mg, 69% yield).

^{1}H NMR (500 MHz, Chloroform-*d*) δ 8.39 (s, 1H), 8.12 (d, *J* = 7.0 Hz, 1H), 7.66 (d, *J* = 2.4 Hz, 1H), 7.60 – 7.54 (m, 3H), 7.52 – 7.46 (m, 4H), 7.46 – 7.35 (m, 5H), 7.21 (d, *J* = 2.4 Hz, 1H), 7.02 – 6.97 (m, 2H), 6.96 – 6.91 (m, 2H), 6.34 (d, *J* = 8.3 Hz, 2H), 5.37 – 5.26 (m, 2H), 3.86 (s, 3H), 3.29 (s, 3H), 2.53 (s, 3H), 2.21 (s, 3H), 1.64 (s, 9H).

^{13}C NMR (126 MHz, Chloroform-*d*) δ 159.1, 158.5, 155.6, 152.9, 152.8, 152.4, 146.9, 141.9, 139.7, 139.5, 135.9, 134.6, 133.4, 132.6, 131.5, 131.4, 130.9, 129.3, 129.2, 128.8, 128.8, 128.7, 128.5, 128.5, 128.0, 127.4, 125.4, 119.1, 118.7, 114.3, 112.7, 108.9, 67.6, 55.5, 55.2, 36.0, 30.8, 19.0, 17.2. m/z HRMS (ESI) found $[M+H]^+$ 743.3486, $C_{49}H_{47}N_2O_5^+$ requires 743.3480. $[\alpha]_D^{22}$ = +161.7 (c 0.5, $CHCl_3$). HPLC: Chiralpak IC column, 90:10 hexane/isopropanol, 1 mL/min; t_R= 9.37 min (minor); 15.31min (major); 97% ee.

benzyl (6-(2-(tert-butyl)-4,6-dimethylphenoxy)-5,7-diethyl-2-phenylquinolin-4-yl)carbamate (**4v**)

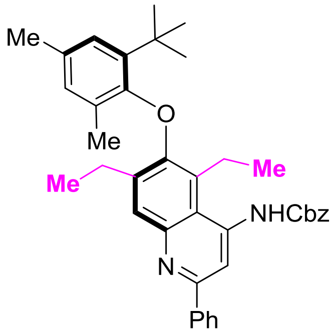

The reaction was performed on 0.1 mmol scale and **4v** was afforded as white foam (41.5 mg, 71% yield).

^{1}H NMR (500 MHz, Chloroform-*d*) δ8.49 (s, 1H), 8.20 – 8.14 (m, 2H), 7.94 (s, 1H), 7.79 (s, 1H), 7.51 (td, *J* = 7.3, 1.3 Hz, 2H), 7.47 – 7.34 (m, 6H), 7.11 (d, *J* = 2.3 Hz, 1H), 6.70 (d, *J* = 2.2 Hz, 1H), 5.32 (s, 2H), 3.36 (dq, *J* = 15.2, 7.6 Hz, 1H), 3.19 (dq, *J* = 15.0, 7.4 Hz, 1H), 2.61 (dq, *J* = 15.2, 7.4 Hz, 1H), 2.36 (dq, *J* = 16.4, 7.0 Hz, 1H), 2.30 (s, 3H), 1.59 (s, 3H), 1.52 (s, 9H), 1.42 (t, *J* = 7.5 Hz, 3H), 1.22 (t, *J* = 7.4 Hz, 3H). ^{13}C NMR (126 MHz, Chloroform-*d*) δ 156.1, 153.3, 152.8, 152.5, 147.7, 141.9, 139.4, 139.0, 138.5, 136.0, 131.6, 129.3, 129.2, 128.8, 128.8, 128.6, 128.3, 127.5, 126.6, 125.9, 125.6, 118.7, 110.6, 67.6, 35.6, 30.7, 23.3, 21.9, 21.0, 18.9, 15.6, 13.5. m/z HRMS (ESI) found $[M+H]^+$ 587.3270, $C_{39}H_{43}N_2O_3^+$ requires 587.3269. $[\alpha]_D^{22}$ =

+105.7 (c 1.0, $CHCl_3$). HPLC: Chiralpak IB column, 90:10 hexane/isopropanol, 1 mL/min; t_R= 6.07 min (major); 6.44 min (minor); 94% ee.

benzyl (6-(2-(tert-butyl)-4,6-dimethylphenoxy)-2,5,7-triphenylquinolin-4-yl)carbamate (**4w**)

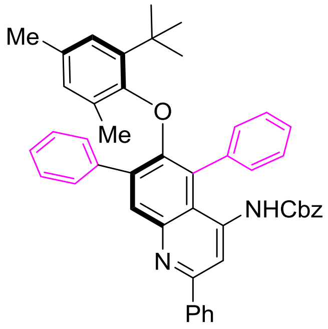

The reaction was performed on 0.1 mmol scale and **4w** was afforded as white foam (52.0 mg, 76% yield).

^{1}H NMR (500 MHz, Chloroform-*d*) δ 8.68 (s, 1H), 8.19 (d, *J* = 7.2 Hz, 2H), 8.13 (s, 1H), 7.54 – 7.47 (m, 3H), 7.46 – 7.41 (m, 2H), 7.41 – 7.33 (m, 3H), 7.33 – 7.30 (m, 2H), 7.30 – 7.27 (m, 1H), 7.23 (d, *J* = 6.7 Hz, 4H), 7.18 – 7.09 (m, 3H), 7.02 (s, 1H), 6.61 (d, *J* = 2.3 Hz, 1H), 6.36 (d, *J* = 2.2 Hz, 1H), 5.01 (d, *J* = 2.5 Hz, 2H), 2.08 (s, 3H), 1.82 (s, 3H), 1.03 (s, 9H). ^{13}C NMR (126 MHz, Chloroform-*d*) δ 156.8, 152.7, 152.5, 150.6, 146.1, 142.4, 139.4, 138.5, 137.7, 137.0, 136.9, 135.9, 134.4, 131.7, 131.5, 130.6, 129.8, 129.6, 129.4, 129.1, 128.9, 128.8, 128.6, 128.5, 128.3, 127.8, 127.5, 127.3, 127.0, 126.2, 125.7, 124.9, 117.8, 108.3, 66.8, 35.1, 30.7, 20.7, 19.3. m/z HRMS (ESI) found $[M+H]^+$ 683.3268, $C_{47}H_{43}N_2O_3^+$ requires 683.3269. $[\alpha]_D^{22}$ = +44.5 (c 1.0, $CHCl_3$). HPLC: Chiralpak IA column, 95:05 hexane/isopropanol, 1 mL/min; t_R= 5.25 min (major); 5.71 min (minor); 97% ee.

benzyl (6-(2-(tert-butyl)-4,6-dimethylphenoxy)-5,7-bis(4-methoxyphenyl)-2-phenylquinolin-4-yl)carbamate (**4x**)

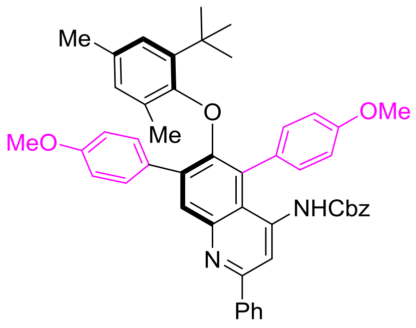

The reaction was performed on 0.1 mmol scale and **4x** was afforded as white foam (68.3 mg, 92% yield).

^{1}H NMR (500 MHz, Chloroform-*d*) δ 8.75 (s, 1H), 8.22 (d, *J* = 7.6 Hz, 2H), 8.16 (s, 1H), 7.52 (t, *J* = 7.5 Hz, 2H), 7.45 (t, *J* = 7.3 Hz, 1H), 7.40 (t, *J* = 7.3 Hz, 2H), 7.38 – 7.34 (m, 2H), 7.32 (d, *J* = 8.5 Hz, 2H), 7.24 (d, *J* = 7.8 Hz, 3H), 7.10 (dd, *J* = 8.5, 2.3 Hz, 1H), 6.97 (dd, *J* = 8.4, 2.7 Hz, 1H), 6.77 – 6.71 (m, 3H), 6.69 (d, *J* = 2.2 Hz, 1H), 6.43 (d, *J* = 2.2 Hz, 1H), 5.04 (s, 2H), 3.78 (s, 3H), 3.66 (s, 3H), 2.14 (s, 3H), 1.81 (s, 3H), 1.09 (s, 9H). ^{13}C NMR (126 MHz, Chloroform-*d*) δ 159.8, 158.9, 156.7, 152.7, 151.2, 146.3, 142.6, 139.5, 138.5, 136.8, 135.8, 133.9, 132.7, 131.5, 131.0, 130.9, 130.7, 130.2, 129.3, 128.8, 128.6, 128.4, 128.2, 127.6, 127.5, 126.0, 125.8, 124.7, 117.8, 114.5, 114.4, 112.9, 107.8, 66.8, 55.4, 55.3, 35.1, 30.7, 20.7, 19.3. m/z HRMS (ESI) found $[M+H]^+$ 743.3477, $C_{49}H_{47}N_2O_5^+$ requires 743.3480. $[\alpha]_D^{22}$ = +35.9 (c 1.0, $CHCl_3$). HPLC: Chiralpak IA column, 90:10 hexane/isopropanol, 1 mL/min; t_R= 6.30 min (minor); 6.94 min (major); 98% ee.

(*R*)-6-(2-(tert-butyl)-4,6-dimethylphenoxy)-5,7-bis(2-methoxyphenyl)-2-phenylquinoline (**4y**)

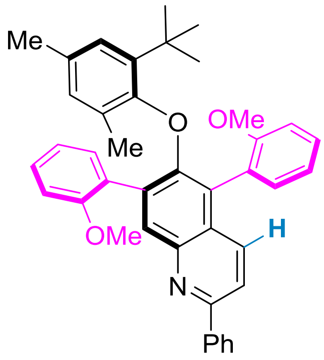

The reaction was performed on 0.1 mmol scale and **4y** was afforded as a yellow solid (25.1 mg, 42% yield).

^{1}H NMR (500 MHz, Chloroform-*d*) δ 8.17 – 8.10 (m, 3H), 7.66 (d, *J* = 8.8 Hz, 1H), 7.50 (t, *J* = 7.7 Hz, 3H), 7.43 (t, *J* = 7.3 Hz, 1H), 7.37 (dd, *J* = 7.5, 1.7 Hz, 1H), 7.34 – 7.28 (m, 1H), 7.14 (q, *J* = 8.0 Hz, 2H), 6.97 (t, *J* = 7.4 Hz, 1H), 6.90 (d, *J* = 8.3 Hz, 1H), 6.78 (t, *J* = 7.4 Hz, 1H), 6.57 (d, *J* = 8.3 Hz, 1H), 6.49 (s, 1H), 6.38 (s, 1H), 3.80 (s, 3H), 3.54 (s, 3H), 2.08 (s, 3H), 1.83 (s, 3H), 1.17 (s, 9H). ^{13}C NMR (126 MHz, Chloroform-*d*) δ 157.2, 157.1, 155.4, 151.6, 144.4, 140.0, 138.7, 135.0, 134.1, 132.1, 131.3, 129.2, 129.0, 128.9, 128.8, 128.8, 128.2, 127.5, 124.7, 123.3, 121.6, 120.0, 119.5, 118.8, 109.9, 109.1, 55.1, 54.3, 35.1, 30.9, 20.8, 18.5. m/z HRMS (ESI) found $[M+H]^+$ 594.2998, $C_{41}H_{40}NO_3^+$ requires 594.3003. $[\alpha]_D^{22}$ = -100.1 (c 0.5, $CHCl_3$). HPLC: Chiralpak IC column, 90:10 hexane/isopropanol, 1 mL/min; t_R= 5.35 min (minor); 5.99 min (major); 83% ee, > 20:1 dr.

benzyl (*R*)-(6-(2-(tert-butyl)-4,6-dimethylphenoxy)-5,7-bis(2-fluorophenyl)-2-phenyl quinolin-4-yl)carbamate (**4z**)

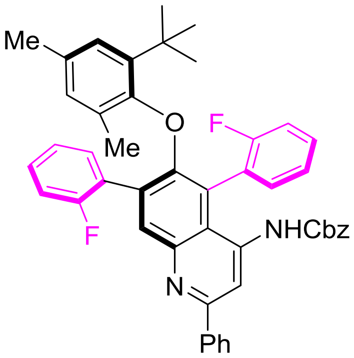

The reaction was performed on 0.1 mmol scale with 3.0 equiv. enamide for 36 h and **4z** was afforded as white foams (48.1 mg, 67% yield).

^{1}H NMR (500 MHz, Chloroform-*d*) δ 8.69 (s, 0.1H, *minor diastereoisomer*), 8.67 (s, 0.9H, *major diastereoisomer*), 8.19 (d, *J* = 7.6 Hz, 2H), 8.11 (s, 1H), 7.56 (td, *J* = 7.3, 1.9 Hz, 1H), 7.49 (t, *J* = 7.5 Hz, 2H), 7.46 – 7.33 (m, 4H), 7.16 – 7.02 (m, 3H), 6.96 (t, *J* = 8.8 Hz, 1H), 6.83 (dt, *J* = 12.7, 8.3 Hz, 2H), 6.55 (s, 0.1H, *minor diastereoisomer*), 6.53 (d, *J* = 2.2 Hz, 0.9H, *major diastereoisomer*), 6.46 (s, 0.1H, *minor diastereoisomer*), 6.39 (d, *J* = 2.3 Hz, 0.9H, *major diastereoisomer*), 5.14 – 4.92 (m, 2H), 2.08 (s, 0.2H, *minor diastereoisomer*), 2.05 (s, 2.8H, *major diastereoisomer*), 1.83 (s, 3H), 1.27 (s, 0.6H, *minor diastereoisomer*), 1.08 (s, 8.4H, *major diastereoisomer*). ^{13}C NMR (126 MHz, Chloroform-*d*) δ 160.0 (d, *J* = 248.3 Hz), 159.2 (d, *J* = 246.5 Hz), 156.7, 152.7, 151.5, 151.3, 145.4, 142.0, 139.3, 138.9, 136.3, 135.8, 133.1, 132.4, 131.8, 131.1 (d, *J* = 8.2 Hz), 130.2, 129.5, 129.4, 129.0 (d, *J* = 8.0 Hz), 128.8, 128.5, 128.4, 128.3, 128.0, 127.5, 125.5 (d, *J* = 16.0 Hz), 125.3, 124.8 (d, *J* = 3.7 Hz), 124.6, 122.8 (d, *J* = 3.1 Hz), 118.7, 117.9, 116.4 (d, *J* = 21.8 Hz), 115.0 (d, *J* = 22.3 Hz).109.2, 67.0, 35.1, 30.8, 30.5 (*minor diastereoisomer*), 29.8 (*minor diastereoisomer*), 22.8 (*minor diastereoisomer*), 20.7, 18.9 (*minor diastereoisomer*), 18.1. ^{19}F NMR (471 MHz, Chloroform-*d*) δ -109.8 (*major diastereoisomer*), -109.0 (*minor diastereoisomer*), -110.4 (*major diastereoisomer*), -110.6 (*minor diastereoisomer*). m/z HRMS (ESI) found $[M+H]^+$ 719.3064, $C_{47}H_{41}F_2N_2O_3^+$ requires 719.3080. $[\alpha]_D^{22}$ = +35.8 (c 0.5, $CHCl_3$). HPLC: Chiralpak IA column, 95:5 hexane/isopropanol, 1 mL/min; for the *major diastereoisomer*: t_R= 5.72 min (minor);

9.57 min (major); 91% ee; for the *minor diastereoisomer*: t_R= 6.19 min (major); 6.55 min (minor); 16% ee, 13:1 dr.

benzyl (6-(2-(tert-butyl)-4,6-dimethylphenoxy)-2-phenyl-5,7-di((E)-styryl)quinolin-4-yl)carbamate (**4aa**)

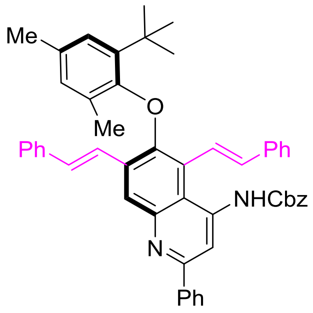

The reaction was performed on 0.05 mmol scale with 3.0 equiv. enamide **3a** for 36 hours and **4aa** was afforded as yellow foam (26.3 mg, 72% yield).

^{1}H NMR (500 MHz, Chloroform-*d*) δ 8.70 (s, 1H), 8.59 (s, 1H), 8.21 (s, 1H), 8.05 (dt, *J* = 7.7, 1.9 Hz, 1H), 7.36 (t, *J* = 7.5 Hz, 2H), 7.32 – 7.29 (m, 1H), 7.27 (d, *J* = 16.7 Hz, 1H), 7.18 – 7.04 (m, 11H), 7.02 – 6.98 (m, 2H), 6.97 – 6.89 (m, 3H), 6.83 (d, *J* = 8.8 Hz, 2H), 6.56 (d, *J* = 2.3 Hz, 1H), 6.55 (d, *J* = 16.6 Hz, 1H), 4.94 – 4.85 (m, 2H), 2.07 (s, 3H), 1.62 (s, 3H), 1.27 (s, 9H). ^{13}C NMR (126 MHz, Chloroform-*d*) δ 156.8, 152.8, 152.3, 151.0, 146.5, 142.3, 139.6, 138.5, 137.4, 136.1, 135.6, 133.3, 132.1, 131.9, 130.7, 129.4, 128.9, 128.8, 128.7, 128.6, 128.2, 128.1, 127.9, 127.9, 127.6, 126.9, 126.3, 123.9, 123.6, 120.2, 117.5, 107.9, 67.2, 35.4, 30.6, 21.0, 18.2. m/z HRMS (ESI) found $[M+H]^+$ 735.3567, $C_{51}H_{47}N_2O_3^+$ requires 735.3582. $[\alpha]_D^{22}$ = +7.6 (c 1.0, $CHCl_3$). HPLC: Chiralpak ID column, 95:05 hexane/isopropanol, 1 mL/min; t_R= 8.67 min (minor); 9.86 min (major); 88% ee.

benzyl (5,7-dibromo-6-(2-(tert-butyl)-4,6-dimethylphenoxy)-2-phenylquinolin-4-yl)carbamate (**4ab**)

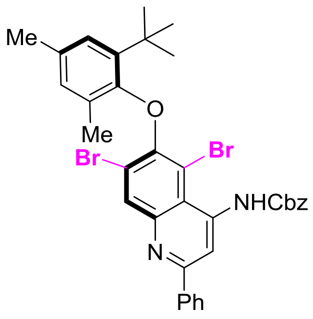

The reaction was performed on 0.1 mmol scale with 3.0 equiv. enamide for 36 h and **4a** was afforded as a yellow solid (28.1 mg, 41% yield).

^{1}H NMR (500 MHz, Chloroform-*d*) δ 9.9 (s, 1H), 8.9 (s, 1H), 8.4 (s, 1H), 8.2 (m, 2H), 7.6 – 7.4 (m, 5H), 7.4 – 7.3 (m, 3H), 7.1 (d, *J* = 2.3 Hz, 1H), 6.8 (d, *J* = 2.3 Hz, 1H), 5.3 (s, 2H), 2.3 (s, 3H), 1.7 (s, 3H), 1.5 (s, 9H). ^{13}C NMR (126 MHz, Chloroform-*d*) δ 157.6, 152.9, 150.7, 148.6, 146.7, 142.2, 139.3, 138.5, 136.7, 135.8, 133.0, 130.6, 130.0, 129.0, 128.8, 128.7, 128.5, 127.6, 126.7, 126.6, 117.5, 115.7, 109.3, 107.5, 67.7, 35.5, 30.9, 21.1, 18.3. m/z HRMS (ESI) found $[M+H]^+$ 689.0827, $C_{35}H_{33}Br_2N_2O_3^+$ requires 689.0832. $[\alpha]_D^{22}$ = +82.9 (c 1.0, $CHCl_3$). HPLC: Chiralpak IB-N column, 90:10 hexane/isopropanol, 1 mL/min; t_R= 5.60 min (major); 6.10 min (minor); 92% ee.

benzyl (6-(2-cyclohexyl-4,6-dimethylphenoxy)-2,5,7-triphenylquinolin-4-yl)carbamate (**4ac**)

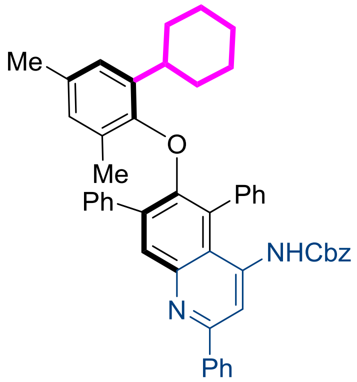

The reaction was performed on 0.1 mmol scale and **4ac** was afforded as white foams (47.7 mg, 67% yield).

While the reaction was performed on 0.05 mmol scale at room temperature, with the removal of the minor diastereomer of **4ac'** by column chromatography, the product **4ac** was afforded with 48% yield and 70% ee.

^{1}H NMR (500 MHz, Chloroform-*d*) δ 8.6 (s, 1H), 8.2 – 8.2 (m, 3H), 7.6 – 7.4 (m, 6H), 7.4 – 7.3 (m, 4H), 7.3 – 7.3 (m, 1H), 7.3 – 7.2 (m, 5H), 7.1 – 7.0 (m, 2H), 7.0 (s, 1H), 6.4 – 6.3 (m, 2H), 5.1 – 4.9 (m, 2H), 2.5 (tt, *J* = 12.0, 2.9 Hz, 1H), 2.1 (s, 3H), 1.8 – 1.7 (m, 3H), 1.7 (s, 3H), 1.5 – 1.3 (m, 1H), 1.3 – 1.2 (m, 3H), 1.2 – 1.1 (m, 1H), 1.0 (qt, *J* = 12.6, 3.3 Hz, 2H). ^{13}C NMR (126 MHz, Chloroform-*d*) δ 156.8, 152.7, 151.3, 150.8, 146.5, 142.5, 139.5, 137.7, 137.6, 137.5, 136.1, 135.9, 133.2, 132.1, 130.4, 130.3, 129.6, 129.5, 129.4, 128.9, 128.6, 128.5, 128.5, 128.3, 127.9, 127.8, 127.5, 126.1, 124.8, 124.2, 117.5, 108.3, 66.8, 38.1, 33.7, 32.7, 27.3, 27.3, 26.5, 20.7, 18.3. m/z HRMS (ESI) found $[M+H]^+$ 709.3426, $C_{49}H_{45}N_2O_3^+$ requires 709.3425. $[\alpha]_D^{23}$ = −9.6 (c 1.0, $CHCl_3$). HPLC: Chiralpak IB-N column, 95:5 hexane/ isopropanol, 1 mL/min; t_R= 7.09 min (major); 7.66 min (minor); 61% ee.

Determination of $t_{1/2}^{rac}$ and racemization barrier

The configurational stability of the atropisomers was determined by measuring the energy barrier to rotation and the racemic half-life. Reactions were conducted at 10 mg/mL concentration. Enantiomeric excess values were determined by chiral HPLC analysis.

benzyl (6-(2-(tert-butyl)-4,6-dimethylphenoxy)-5,7-dimethyl-2-phenylquinolin-4-yl)carbamate (**4a**)

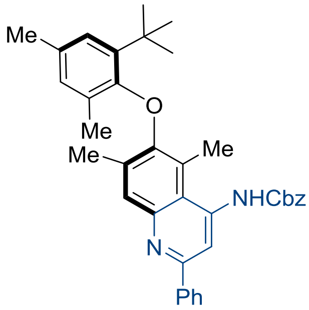

Racemization of **4a** in mesitylene at 150 °C

Time (s)	ee (%)	ln(ee)
0	95.5	-4.5591
7200	91.7	-4.5185
18000	86.6	-4.4613
25320	84.8	-4.4403
28920	83.7	-4.4272
32520	81.8	-4.4043

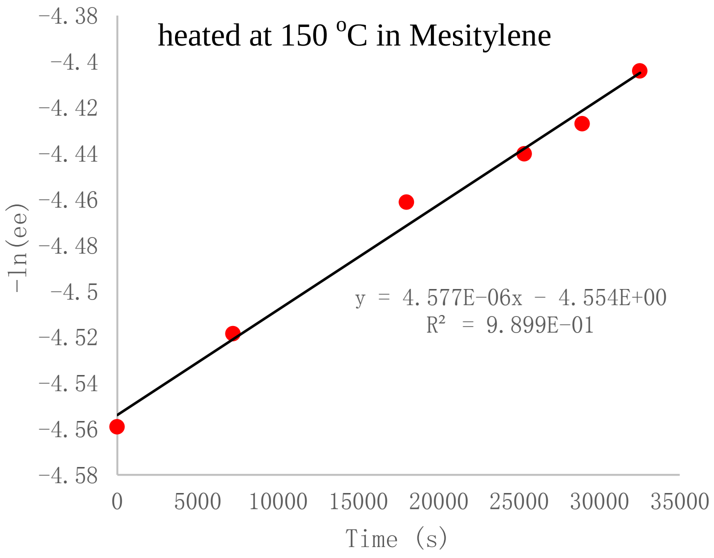

$-\ln(ee) = 2k_{ent}t + C$

Therefore, $k_{ent} = 2.29\times10^{-6}\ s^{-1}$

$t_{1/2}{}^{150}{}_{rac} = \ln(2)/2k_{ent} = 42.1$ h

Employing the Eyring equation:

$$\Delta G = -RT\ln\left(\frac{k_{ent} \times h}{k_B \times T}\right) = -8.314 \times 423.15 \ln\left(\frac{2.29 \times 10^{-6} \times 6.626 \times 10^{-34}}{1.381 \times 10^{-23} \times 423.15}\right)$$

=150.6 kJ/mol (36.0 kcal/mol)

benzyl (6-((3-(tert-butyl)-5-methyl-[1,1'-biphenyl]-2-yl)oxy)-5,7-dimethyl-2-phenylquinolin-4-yl)carbamate (**4t**)

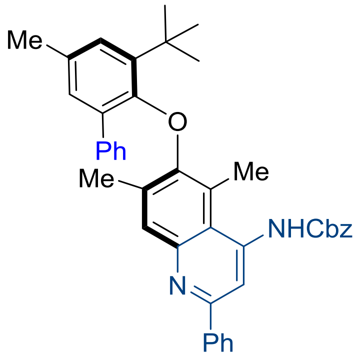

Racemization of **4t** in mesitylene at 150 °C

Time (s)	ee (%)	ln(ee)
0	96.1	-4.5654
3600	78.6	-4.3644
7200	63.8	-4.1558
9000	58.1	-4.0622
12660	45.6	-3.8199
15000	39.1	-3.6661
18000	32.9	-3.4935

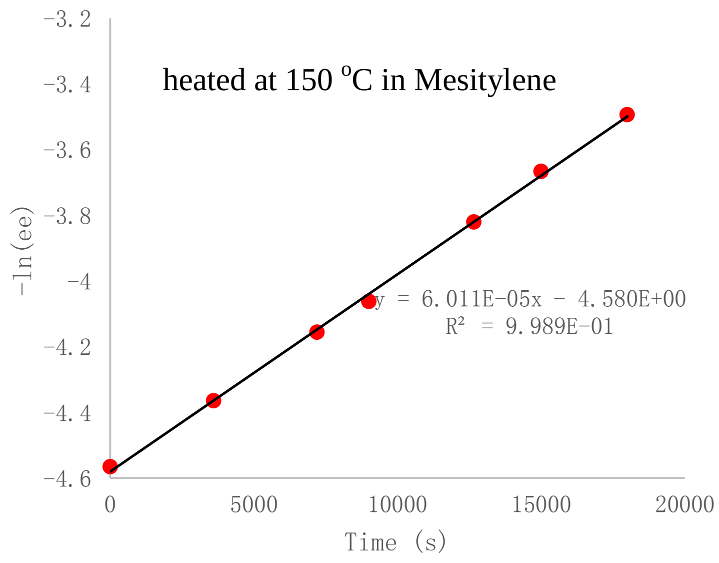

$-\ln(ee) = 2k_{ent}t + C$

Therefore, $k_{ent} = 3.01 \times 10^{-5}\ s^{-1}$

$t_{1/2}{}^{150}{}_{rac} = \ln(2)/2k_{ent} = 3.2$ h

Employing the Eyring equation:

$$\Delta G = -RT\ln\left(\frac{k_{ent} \times h}{k_B \times T}\right) = -8.314 \times 423.15 \ln\left(\frac{3.01 \times 10^{-5} \times 6.626 \times 10^{-34}}{1.381 \times 10^{-23} \times 423.15}\right)$$

=141.5 kJ/mol (33.9 kcal/mol)

benzyl (6-(2-(tert-butyl)-4,6-dimethylphenoxy)-2,5,7-triphenylquinolin-4-yl)carbamate (**4w**)

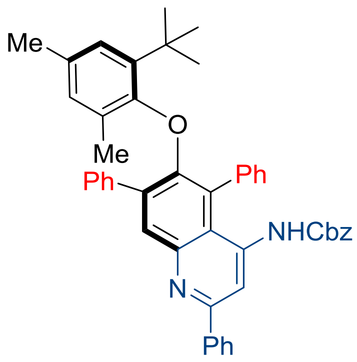

Racemization of **4w** in diphenyl ether at 170 ^{o}C

Time (s)	ee (%)	ln(ee)
0	96.6	-4.5706
2400	95.5	-4.5591
6000	94.7	-4.5507
9600	92.9	-4.5315
18000	90.3	-4.5031
46200	83.8	-4.4284
86400	74.2	-4.3068
172800	57.5	-4.0518

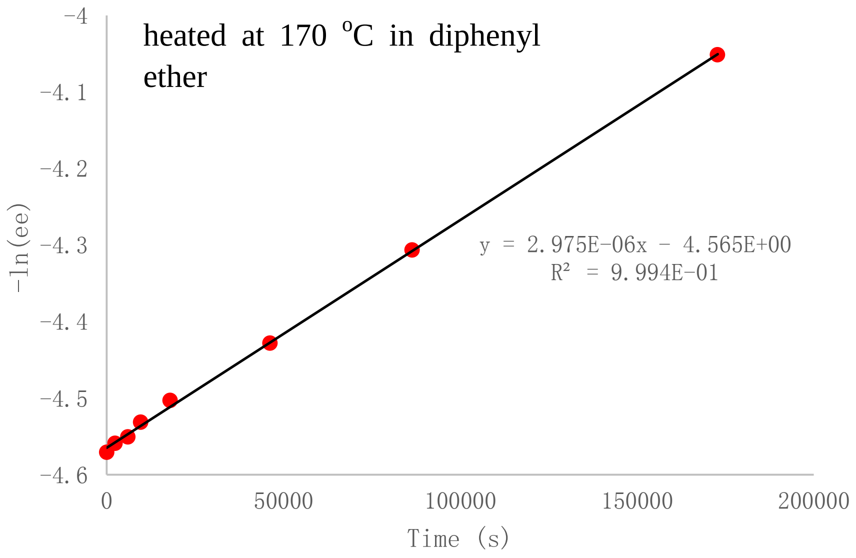

-ln(ee) = 2k_{ent}t + C

Therefore, k_{ent} = 1.49 ×10^{-6} s^{-1}

$t_{1/2}{}^{170}{}_{rac}$ = ln(2)/2k_{ent} = 64.7 h

Employing the Eyring equation:

$$\Delta G = -RT\ln\left(\frac{k_{ent}\times h}{k_B\times T}\right) = -8.314\times 443.15\ln\left(\frac{1.49\times 10^{-6}\times 6.626\times 10^{-34}}{1.381\times 10^{-23}\times 443.15}\right)$$

=159.4 kJ/mol (38.1 kcal/mol)

benzyl (6-(2-cyclohexyl-4,6-dimethylphenoxy)-2,5,7-triphenylquinolin-4-yl)carbamate (**4ac**)

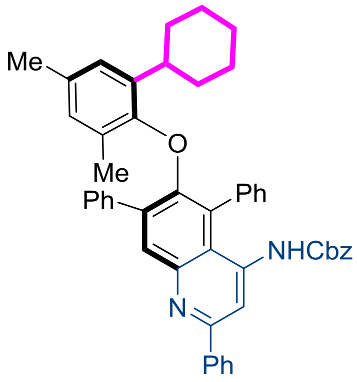

Racemization of **4ac** in Mesitylene at 80 ^{o}C

Time (s)	ee (%)	ln(ee)
0	69.5	-4.2408
1800	66.5	-4.1978
2700	65.1	-4.1756
3600	64.4	-4.1645
4500	62.6	-4.1369
7200	59.2	-4.0814
10800	56.0	-4.0246

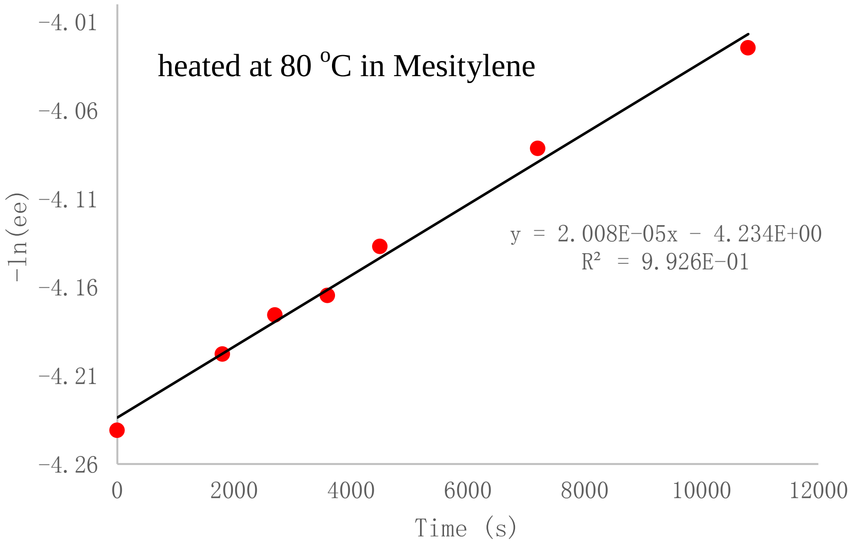

$-\ln(ee) = 2k_{ent}t + C$

Therefore, $k_{ent} = 1.00 \times 10^{-5}\ s^{-1}$

$t_{1/2}{}^{80}{}_{rac} = \ln(2)/2k_{ent} = 9.6$ h

Employing the Eyring equation:

$$\Delta G = -RT \ln\left(\frac{k_{ent} \times h}{k_B \times T}\right) = -8.314 \times 353.15 \ln\left(\frac{1.00 \times 10^{-5} \times 6.626 \times 10^{-34}}{1.381 \times 10^{-23} \times 353.15}\right)$$

=120.8 kJ/mol (28.9 kcal/mol)

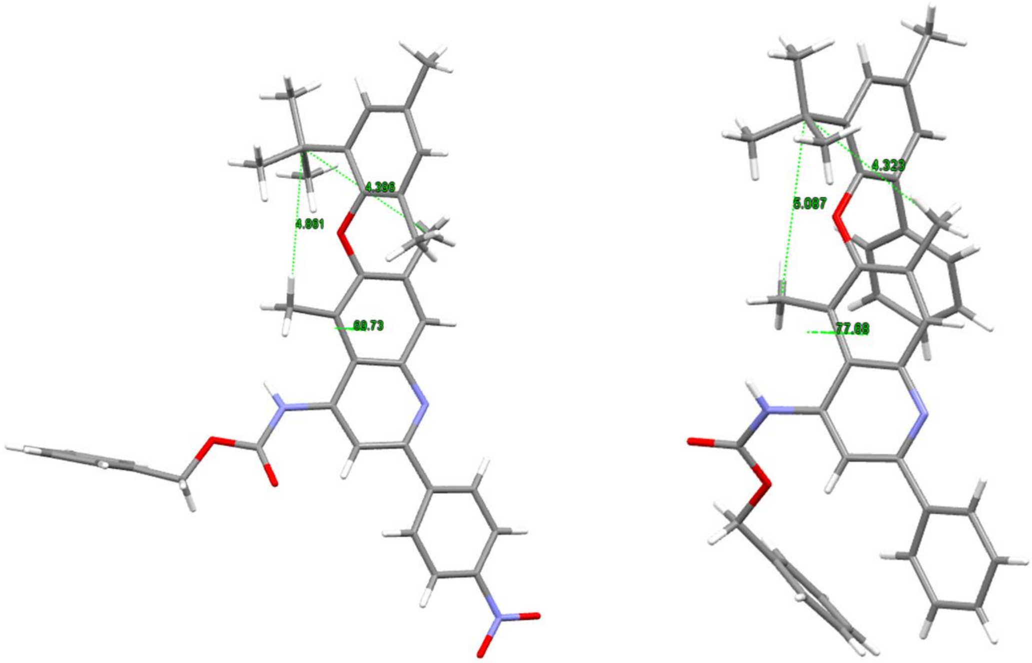

X-ray structure of **4b** X-ray structure of **4o**

Figure S1. Comparison of the dihedral angle between the diaryl rings of the diaryl ether and distance between the quaternary carbon of the t-Bu group and the carbon of the two methyl groups in the X-ray structure of **4b** and **4o**.

DFT calculation of the racemization process

Computational Method:

All the calculations were performed at the B3LYP/Def2-TZVPP//B3LYP/Def2-SVP level with included the D3(BJ) Grimme correction[1] for the dispersion. The structure optimization and frequency analysis were conducted of the B3LYP[3] functional and Def2-SVP basis[2], while the single point energy was calculated with the B3LYP[3] functional and Def2-TZVPP basis[2], under the SMD toluene model[4]. The calculations were performed with Gaussian 09, Revision E.01[5]. Molecule visualization was performed with CYLview20[6].

(1) Grimme, S.; Antony, J.; Ehrlich, S.; Krieg, H. A Consistent and Accurate Ab

Initio Parametrization of Density Functional Dispersion Correction (DFT-D) for the 94 Elements H-Pu. *J. Chem. Phys.* (**2010**), 132, 154104.

(2) A. Schaefer, H. Horn, and R. Ahlrichs, Fully optimized contracted Gaussian-basis sets for atoms Li to Kr, *J. Chem. Phys*., 97 (**1992**) 2571-2577.

(3) Stephens, Philip J. et al. Ab Initio Calculation of Vibrational Absorption and Circular Dichroism Spectra Using Density Functional Force Fields. *The Journal of Physical Chemistry* 98 (**1994**): 11623-11627.

(4) A. V. Marenich, C. J. Cramer, and D. G. Truhlar, Universal solvation model based on solute electron density and a continuum model of the solvent defined by the bulk dielectric constant and atomic surface tensions, *J. Phys. Chem. B*, 113 (**2009**) 6378-96.

(5) Gaussian 09, Revision E.01, M. J. Frisch, G. W. Trucks, H. B. Schlegel, G. E. Scuseria, M. A. Robb, J. R. Cheeseman, G. Scalmani, V. Barone, B. Mennucci, G. A. Petersson, H. Nakatsuji, M. Caricato, X. Li, H. P. Hratchian, A. F. Izmaylov, J. Bloino, G. Zheng, J. L. Sonnenberg, M. Hada, M. Ehara, K. Toyota, R. Fukuda, J. Hasegawa, M. Ishida, T. Nakajima, Y. Honda, O. Kitao, H. Nakai, T. Vreven, J. A. Montgomery, Jr., J. E. Peralta, F. Ogliaro, M. Bearpark, J. J. Heyd, E. Brothers, K. N. Kudin, V. N. Staroverov, T. Keith, R. Kobayashi, J. Normand, K. Raghavachari, A. Rendell, J. C. Burant, S. S. Iyengar, J. Tomasi, M. Cossi, N. Rega, J. M. Millam, M. Klene, J. E. Knox, J. B. Cross, V. Bakken, C. Adamo, J. Jaramillo, R. Gomperts, R. E. Stratmann, O. Yazyev, A. J. Austin, R. Cammi, C. Pomelli, J. W. Ochterski, R. L. Martin, K. Morokuma, V. G. Zakrzewski, G. A. Voth, P. Salvador, J. J. Dannenberg, S. Dapprich, A. D. Daniels, O. Farkas, J. B. Foresman, J. V. Ortiz, J. Cioslowski, and D. J. Fox, Gaussian, Inc., Wallingford CT, 2013.

(6) CYLview20; Legault, C. Y., Université de Sherbrooke, 2020 (http://www.cylview.org)

Hence, the corresponding absolute energy and some thermal corrections (in Hartree) were shown in table, and all the molecules were treated as ideal gas at the thermodynamic standard condition (298 K and 100 kPa). The final data was given in the form of kcal mol^{-1} (1 Hatree = 2625.5 kJ mol^{-1} = 627.5 kcal mol^{-1}). While the temperature compensation was performed below to match experimental situations.

$$\mathbf{H_{calc} = H_{corr} + SPE}$$

$$\mathbf{G_{calc} = H_{calc} - T\ S_{calc}}$$

Table S1. Thermodynamic Data

**Sub.**	**ZPE / Hartree**	**H_{corr} / Hartree**	**H / Hatree**	**S / kcal mol^{-1} K^{-1}**
(*R*)-**4a**	-1768.937518	0.706661	-1768.230857	240.868
**TS-4a**	-1768.882967	0.705478	-1768.177489	235.973
(*R*)-**4o**	-1960.770946	0.762613	-1960.008333	256.703
**TS-4o**	-1960.723197	0.761736	-1959.961461	249.223
(*R*)-**4w**	-2152.599548	0.818894	-2151.780654	278.204
**TS-4w**	-2152.545794	0.817257	-2151.728537	262.331

**Sub.**	**H / kcal mol^{-1}**	**Temp. / K**	**G / kcal mol^{-1}**	**$\Delta G^{\neq}$ / kcal mol^{-1}**
(*R*)-**4a**	-1109564.9	423	-1109666.7	
**TS-4a**	-1109531.4	423	-1109631.2	35.5
(*R*)-**4o**	-1229905.2	423	-1230013.8	
**TS-4o**	-1229875.8	423	-1229981.2	32.6
(*R*)-**4w**	-1350242.4	443	-1350365.6	
**TS-4w**	-1350209.7	443	-1350325.9	39.7

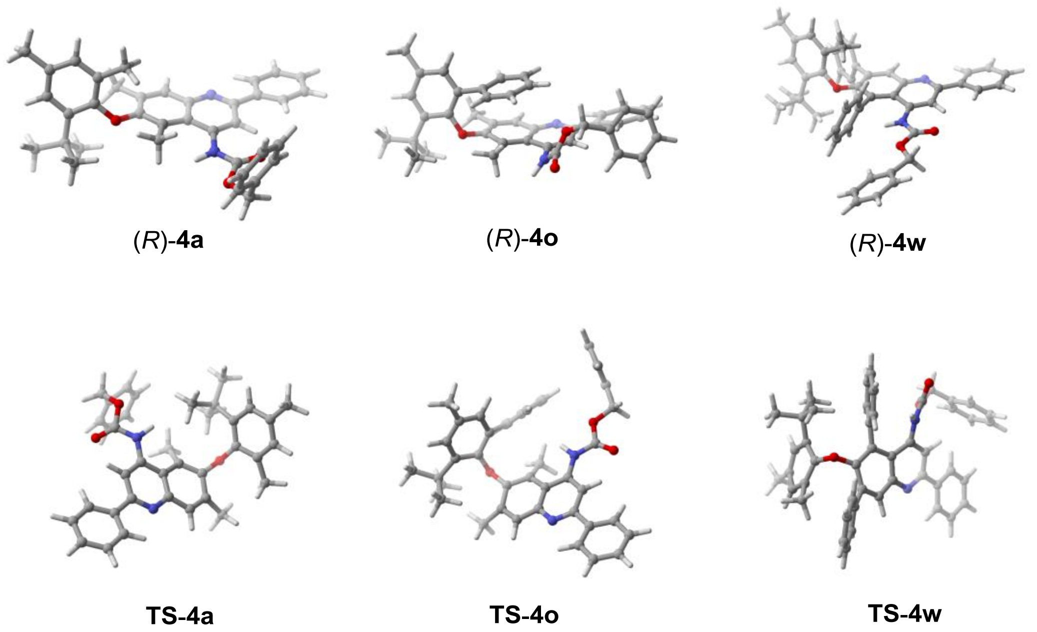

Fig S2. Structure of (*R*)-**4a**, (*R*)-**4o**, (*R*)-**4w** and their transition states of racemization. All structures were given as Cartesian coordinates (in Å).

(*R*)-**4a**

C	6.522915	-0.590803	-0.502801
C	6.512776	-0.361744	-1.886602
C	5.278717	-0.183273	-2.511226
C	4.074435	-0.188594	-1.792397
C	4.141158	-0.345853	-0.396929
C	5.359488	-0.604780	0.275772
C	5.409563	-0.940782	1.782554
O	2.991168	-0.345268	0.369258
C	4.868692	0.220562	2.642880
C	6.847969	-1.220116	2.252037
C	4.581715	-2.217653	2.049440
C	1.962807	0.560094	0.250394
C	2.215815	1.957919	0.106499
C	1.118436	2.786654	0.018296

C	-0.214578	2.315880	0.120867
C	-0.449337	0.917189	0.352623
C	0.676345	0.025870	0.354213
C	0.557844	-1.482513	0.388812
C	2.767544	-0.045548	-2.531315
C	7.803515	-0.339988	-2.665008
C	3.601317	2.543434	0.033706
N	-1.197074	3.241000	-0.004429
C	-2.458606	2.866257	0.106648
C	-2.812122	1.530784	0.421080
C	-1.832748	0.562351	0.570496
C	-3.503338	3.913130	-0.071932
N	-2.165445	-0.732399	0.974891
C	-3.121873	5.265679	-0.138395
C	-4.074539	6.269719	-0.301490
C	-5.431157	5.943897	-0.406615
C	-5.822523	4.603760	-0.350292
C	-4.868934	3.597387	-0.184216
C	-4.278241	-6.055010	-1.118327
H	7.486448	-0.767611	-0.026371
H	5.237226	-0.047615	-3.595642
H	4.948080	-0.039825	3.710991
H	3.815799	0.441333	2.432266
H	5.457974	1.137424	2.482625
H	7.503498	-0.344780	2.124592
H	6.837848	-1.470254	3.324108
H	7.301464	-2.070264	1.719977
H	4.645772	-2.493574	3.114889
H	4.968139	-3.063400	1.458307

H	3.523401	-2.076556	1.797188
H	1.246687	3.861751	-0.119629
H	0.406924	-1.877866	1.410432
H	1.485491	-1.941954	0.031241
H	-0.257810	-1.846725	-0.250575
H	2.332074	0.961673	-2.433462
H	2.010747	-0.754963	-2.167078
H	2.919554	-0.236297	-3.603542
H	7.624974	-0.197980	-3.741198
H	8.366662	-1.279731	-2.537407
H	8.463131	0.476192	-2.324225
H	3.552591	3.632807	0.171998
H	4.083324	2.347346	-0.936305
H	4.263436	2.128247	0.803251
H	-3.845865	1.251775	0.588220
C	-5.031726	-5.462716	-2.136006
C	-5.602830	-4.202323	-1.935050
C	-5.421858	-3.535445	-0.721382
C	-4.667195	-4.122971	0.305858
C	-4.097214	-5.386820	0.094823
C	-4.434460	-3.393658	1.603576
O	-3.252897	-2.565221	1.540170
C	-3.407625	-1.309309	1.070880
O	-4.473729	-0.802968	0.794138
H	-1.408702	-1.347954	1.237009
H	-2.061049	5.504624	-0.057562
H	-3.757887	7.314983	-0.346201
H	-6.178851	6.730729	-0.534282
H	-6.878502	4.336670	-0.439278

H	-5.199795	2.558183	-0.158552
H	-3.832230	-7.041211	-1.269467
H	-5.175780	-5.984620	-3.085374
H	-6.193442	-3.736252	-2.727654
H	-5.855355	-2.545869	-0.564875
H	-3.508601	-5.851763	0.890657
H	-5.294123	-2.764681	1.869795
H	-4.232840	-4.098557	2.420539

(*R*)-**4o**

C	-6.542270	0.069884	0.923243
C	-6.079986	0.266769	2.233396
C	-4.706804	0.184235	2.458059
C	-3.800631	-0.041314	1.410249
C	-4.299589	-0.125944	0.094800
C	-5.692068	-0.138420	-0.169003
C	-6.259360	-0.430748	-1.576413
O	-3.454641	-0.231481	-0.993276
C	-5.767675	-1.819720	-2.040600
C	-7.797494	-0.466001	-1.569594
C	-5.833946	0.639097	-2.603475
C	-2.228654	0.405907	-1.012258
C	-2.175612	1.822749	-0.845656
C	-0.932426	2.409634	-0.802689
C	0.263675	1.654679	-0.895938
C	0.187013	0.241277	-1.156688
C	-1.103574	-0.381153	-1.240424
C	-1.305296	-1.853446	-1.503406
C	-2.358123	-0.245348	1.748758

C	-7.051692	0.526772	3.355851
C	-3.415091	2.656363	-0.670927
N	1.423476	2.321277	-0.683841
C	2.572657	1.668231	-0.708011
C	2.617685	0.284625	-1.005017
C	1.454453	-0.427787	-1.222505
C	3.815278	2.438565	-0.430245
N	1.559005	-1.808592	-1.494009
C	3.763901	3.843779	-0.386804
C	4.909516	4.592129	-0.122346
C	6.131722	3.951505	0.109570
C	6.193771	2.556076	0.076248
C	5.047923	1.805010	-0.191310
C	6.915287	-1.764184	-0.913956
H	-7.619405	0.084229	0.762356
H	-4.312289	0.268266	3.473750
H	-6.197327	-2.063173	-3.026071
H	-4.674619	-1.854503	-2.129733
H	-6.082532	-2.602446	-1.332088
H	-8.158819	-0.704553	-2.581724
H	-8.234043	0.504103	-1.285725
H	-8.193588	-1.235076	-0.889036
H	-6.177881	1.639228	-2.295651
H	-4.747127	0.669199	-2.742650
H	-6.292269	0.417554	-3.581176
H	-0.827339	3.485016	-0.648623
H	-1.012180	-2.121734	-2.533133
H	-0.729134	-2.481428	-0.814100
H	-2.362600	-2.115639	-1.398591

C	-1.541793	0.821035	2.155707
C	-0.188214	0.616771	2.430965
C	0.364647	-0.662578	2.327204
H	-7.836184	-0.246968	3.397832
H	-6.545311	0.547400	4.332224
H	-7.564465	1.495217	3.224710
H	-4.201088	2.375508	-1.382381
H	-3.844860	2.538797	0.336594
H	-3.178904	3.720222	-0.814443
H	3.568824	-0.235900	-1.076140
C	7.583747	-1.025990	0.069561
C	7.047555	-0.938621	1.356347
C	5.843103	-1.582156	1.657266
C	5.172633	-2.331156	0.682636
C	5.720829	-2.417950	-0.607870
C	3.848985	-2.985589	0.983773
O	2.788316	-2.258654	0.336741
C	2.321357	-2.736459	-0.833436
O	2.510884	-3.860268	-1.250109
H	1.048436	-2.190214	-2.280599
H	2.804053	4.329780	-0.564728
H	4.850275	5.683283	-0.097054
H	7.030321	4.537711	0.317799
H	7.138530	2.041238	0.264039
H	5.125670	0.717802	-0.191347
H	7.331637	-1.835723	-1.921663
H	8.521510	-0.518971	-0.170201
H	7.560857	-0.358592	2.126912
H	5.416430	-1.498775	2.660318

H	5.205068	-3.004110	-1.372665
H	3.821295	-4.028845	0.641115
H	3.626266	-2.951134	2.058179
C	-0.450186	-1.739638	1.969619
C	-1.801813	-1.532587	1.685493
H	-1.964437	1.825700	2.216514
H	0.443011	1.465787	2.703386
H	1.429607	-0.818057	2.509232
H	-0.025905	-2.743829	1.894091
H	-2.433964	-2.371757	1.387536

(*R*)-**4w**

C	-5.600749	-1.971071	0.580781
C	-5.812623	-1.505583	1.880445
C	-4.724645	-0.949918	2.560597
C	-3.477665	-0.805303	1.950194
C	-3.337102	-1.207594	0.605335
C	-4.372693	-1.858045	-0.093812
C	-4.245854	-2.553600	-1.471210
O	-2.105628	-1.045089	-0.007378
C	-3.004686	-2.172847	-2.298800
C	-4.179204	-4.074551	-1.203669
C	-5.482179	-2.250263	-2.344075
C	-1.469118	0.161037	-0.094029
C	-2.168144	1.390773	-0.293191
C	-1.429445	2.555662	-0.285436
C	-0.016907	2.571303	-0.160365
C	0.688903	1.323978	-0.073785
C	-0.074902	0.113367	-0.001301

C	0.531025	-1.233306	0.246632
C	-2.319423	-0.240491	2.729971
C	-7.173200	-1.588899	2.522374
C	0.615558	-2.185205	-0.782763
N	0.582616	3.786313	-0.152943
C	1.899696	3.857544	-0.080266
C	2.708586	2.693156	-0.066809
C	2.131332	1.434053	-0.082226
C	2.511532	5.215390	-0.044282
N	2.912598	0.283279	-0.137473
C	1.704274	6.342814	-0.282961
C	2.244293	7.627244	-0.255811
C	3.604117	7.814426	0.015045
C	4.415580	6.703652	0.259979
C	3.876074	5.416153	0.230096
C	5.317176	-5.148805	-0.890545
C	6.473435	-5.764020	-0.404020
C	7.447382	-4.992440	0.237493
C	7.262015	-3.617132	0.391686
C	6.106613	-2.993316	-0.100433
C	5.134404	-3.770888	-0.742925
C	5.960249	-1.500187	0.041266
O	4.580888	-1.151648	-0.072775
C	4.272083	0.159806	0.001465
O	5.085173	1.042314	0.165660
H	-6.434850	-2.456293	0.073274
H	-4.843297	-0.623020	3.597626
H	-3.076793	-2.662509	-3.283498
H	-2.935993	-1.090491	-2.467444

H	-2.074877	-2.503430	-1.823813
H	-4.103072	-4.628597	-2.154215
H	-5.074534	-4.432287	-0.672439
H	-3.300064	-4.327544	-0.590099
H	-6.418622	-2.622142	-1.904801
H	-5.591712	-1.168785	-2.507041
H	-5.372133	-2.737518	-3.325902
H	-1.924109	3.520229	-0.408417
C	1.182293	-3.440548	-0.547336
C	1.672805	-3.762996	0.720992
C	1.594482	-2.823194	1.752501
H	-2.547709	-0.243376	3.805490
H	-1.407739	-0.833302	2.572091
H	-2.079931	0.796455	2.444220
H	-7.753680	-2.441698	2.137949
H	-7.101498	-1.688603	3.616543
H	-7.760719	-0.676408	2.315928
C	1.025730	-1.569366	1.518624
C	-3.637550	1.484706	-0.558941
C	-4.110586	1.345220	-1.871825
H	3.790292	2.764021	-0.075768
H	2.423971	-0.599842	-0.234967
H	0.645812	6.182566	-0.489828
H	1.600852	8.489694	-0.447994
H	4.028316	8.821382	0.037063
H	5.477692	6.838159	0.479339
H	4.529458	4.567866	0.438481
H	4.548461	-5.744628	-1.389535
H	6.614799	-6.841338	-0.520324

H	8.352665	-5.464957	0.626736
H	8.022644	-3.020024	0.903013
H	4.227474	-3.295185	-1.116332
H	6.351681	-1.155336	1.011868
H	6.532416	-0.970338	-0.740278
C	-5.470738	1.488585	-2.157749
C	-6.376788	1.767931	-1.132048
C	-5.911309	1.925457	0.176564
C	-4.550521	1.797583	0.458624
H	0.239195	-1.930613	-1.775227
H	1.243557	-4.168000	-1.360447
H	2.126265	-4.739841	0.902525
H	1.983655	-3.065163	2.744238
H	0.980310	-0.830988	2.322056
H	-3.403323	1.133819	-2.676394
H	-5.822167	1.382746	-3.186943
H	-7.442067	1.870859	-1.352540
H	-6.611083	2.152013	0.984596
H	-4.191140	1.929056	1.479766

TS-4a

C	-4.549345	-1.508672	-1.685635
C	-4.480503	-3.628516	-0.632000
C	-3.338021	-1.734496	0.397122
C	-3.841222	-0.892033	-0.639533
C	-3.688357	0.640843	-0.752210
C	-4.821235	1.289868	-1.584470
C	-3.799203	1.315318	0.631342
C	-2.379688	0.915570	-1.528807

C	-1.128275	-1.437063	1.354529
C	1.588614	-1.980805	0.795686
C	1.000786	-0.727493	0.426967
C	-0.323815	-0.396268	0.868511
C	-0.678073	1.048971	1.181688
H	-4.899759	-0.889471	-2.508783
H	-4.774760	-4.680279	-0.576565
H	-4.728030	2.384801	-1.506506
H	-4.775241	1.045960	-2.655188
H	-5.816577	1.010123	-1.206979
H	-3.498008	2.373071	0.574741
H	-3.213360	0.819369	1.403657
H	-4.851234	1.286461	0.958100
H	-1.543019	0.326429	-1.136478
H	-2.510529	0.604776	-2.576706
H	-2.116477	1.985791	-1.526284
H	0.240878	1.562205	1.497783
H	-1.127177	1.654225	0.391701
H	-1.367774	1.070382	2.032853
C	0.831402	-2.840233	1.626423
H	1.353428	-3.682144	2.086677
C	-0.483715	-2.569908	1.957517
C	-3.762433	-3.085728	0.440717
C	-4.845688	-2.869221	-1.738385
C	-3.561595	-3.995249	1.619996
H	-4.345057	-4.766629	1.621536
H	-2.597355	-4.518587	1.580575
H	-3.623054	-3.452126	2.568163
C	-1.060802	-3.270703	3.166954

H	-2.010295	-2.830295	3.483306
H	-1.197917	-4.351471	3.026537
H	-0.344992	-3.144185	3.994673
C	3.641629	-1.443043	-0.131290
C	-5.552538	-3.475133	-2.921529
H	-6.221921	-2.750331	-3.410147
H	-4.827206	-3.812854	-3.682781
H	-6.149798	-4.353090	-2.630996
O	-2.511736	-1.261136	1.457407
C	1.818755	0.133286	-0.373058
N	2.874285	-2.305810	0.515346
C	5.057535	-1.833966	-0.372664
C	5.445171	-3.176013	-0.207577
C	6.033529	-0.895083	-0.750262
C	6.764496	-3.568751	-0.425870
H	4.686441	-3.898332	0.095376
C	7.355843	-1.288824	-0.964902
H	5.770228	0.158681	-0.856360
C	7.726356	-2.626992	-0.807751
H	7.046161	-4.617171	-0.298377
H	8.101674	-0.543280	-1.251470
H	8.761106	-2.934744	-0.978037
C	3.132978	-0.210690	-0.622999
H	3.741814	0.437062	-1.248118
N	1.233589	1.259036	-0.966415
H	0.247779	1.205453	-1.193442
C	1.801766	2.493280	-1.129625
O	2.911646	2.825973	-0.779865
O	0.903282	3.301816	-1.741851

C	1.046684	4.717365	-1.500533
H	0.540055	5.201768	-2.345138
H	2.112441	4.981568	-1.513495
C	0.394761	5.065548	-0.187534
C	-0.990832	5.277478	-0.124266
C	1.140692	5.073674	1.000296
C	-1.621605	5.491766	1.102929
H	-1.578674	5.269331	-1.046547
C	0.510506	5.287825	2.229219
H	2.217097	4.894036	0.958573
C	-0.870896	5.494045	2.283131
H	-2.701177	5.657450	1.139919
H	1.100859	5.291067	3.148797
H	-1.363207	5.659456	3.244622

TS-4o

C	-4.713542	-0.362740	1.979097
C	-3.068277	1.344794	1.939063
C	-3.365517	-0.094496	-0.006721
C	-4.466594	-0.719876	0.643365
C	-5.442510	-1.738213	0.014057
C	-6.850258	-1.645685	0.652081
C	-5.678438	-1.434155	-1.482060
C	-4.922122	-3.162227	0.315270
C	-1.714130	-1.351579	-1.193465
C	0.521151	-2.963294	-0.617871
C	-1.814362	-2.774457	-1.331386
C	-2.935342	-3.436557	-2.094498
H	-5.519009	-0.868175	2.508996

H	-2.596493	2.219432	2.391092
H	-7.539224	-2.286118	0.078998
H	-6.880084	-2.004223	1.690688
H	-7.245229	-0.618247	0.626490
H	-6.267243	-2.240857	-1.946572
H	-4.759351	-1.288430	-2.052816
H	-6.261138	-0.503424	-1.576163
H	-3.850266	-3.270663	0.128097
H	-5.075785	-3.384471	1.382926
H	-5.463898	-3.923282	-0.269219
H	-3.112179	-4.462299	-1.742503
H	-3.874440	-2.887893	-2.075903
H	-2.623565	-3.504363	-3.151786
C	-0.449474	-0.752818	-1.182144
C	-2.773190	1.044971	0.598743
C	-3.994373	0.610661	2.672714
C	-0.242352	0.514957	-1.988752
H	-1.154038	0.734139	-2.552364
H	0.019954	1.436309	-1.463633
H	0.565152	0.312376	-2.711031
C	-4.269365	0.894422	4.125519
H	-5.349893	0.986786	4.321767
H	-3.895435	0.076006	4.764682
H	-3.781455	1.823184	4.456467
O	-2.870176	-0.561593	-1.250689
C	-2.003712	2.090488	-0.123138
C	-2.496981	2.616737	-1.328240
C	-0.862448	2.681918	0.449049
C	-1.856174	3.687742	-1.952934

H	-3.391563	2.178488	-1.774706
C	-0.218839	3.752732	-0.175322
H	-0.459943	2.280647	1.382000
C	-0.713291	4.256435	-1.382562
H	-2.254374	4.082875	-2.890998
H	0.678220	4.184253	0.270209
H	-0.208052	5.091996	-1.871050
N	1.533423	-3.794665	-0.272679
C	0.666028	-1.542270	-0.736550
C	2.973645	-1.892378	-0.038445
H	3.936351	-1.487754	0.252888
C	2.729464	-3.289061	-0.020880
C	1.951724	-1.015140	-0.366129
C	-0.713992	-3.540747	-1.001698
H	-0.749414	-4.628023	-1.095522
C	3.816985	-4.248703	0.321871
C	3.496202	-5.594395	0.578003
C	5.166022	-3.858168	0.389795
C	4.489837	-6.516786	0.901031
H	2.449435	-5.893342	0.515830
C	6.161358	-4.783663	0.709698
H	5.453094	-2.827560	0.177056
C	5.828447	-6.115671	0.969648
H	4.219487	-7.556866	1.101038
H	7.204530	-4.460725	0.752757
H	6.608417	-6.838500	1.221885
N	2.106862	0.367196	-0.261977
H	1.267053	0.931156	-0.305311
C	3.251603	1.083099	-0.012497

O	4.378910	0.655331	0.100558
O	2.919698	2.386896	0.090512
C	3.996717	3.313024	0.329819
H	4.775864	3.160373	-0.431857
H	4.440371	3.105123	1.314987
C	3.410586	4.693823	0.262426
C	3.076071	5.391673	1.430143
C	3.111430	5.263548	-0.983756
C	2.449605	6.638898	1.355321
H	3.299703	4.950687	2.405100
C	2.486510	6.509139	-1.061274
H	3.363604	4.718003	-1.897078
C	2.150878	7.197423	0.110051
H	2.190164	7.173848	2.272024
H	2.258874	6.945211	-2.037061
H	1.658449	8.171097	0.050505

TS-4w

C	-3.886837	-1.116840	2.954890
C	-4.812102	1.022721	2.499837
C	-3.928848	-0.321911	0.690004
C	-3.770911	-1.413362	1.584364
C	-3.833574	-2.917204	1.213551
C	-5.195684	-3.423466	1.760680
C	-3.865798	-3.193292	-0.293241
C	-2.717566	-3.728844	1.894279
C	-2.174494	0.358880	-0.731165
C	0.226624	1.774958	-0.274193
C	0.282150	0.386989	-0.636784

C	-0.960948	-0.330734	-0.800343
H	-3.695145	-1.915143	3.674629
H	-5.354374	1.914137	2.830168
H	-5.326179	-4.487009	1.501372
H	-5.270400	-3.330625	2.853436
H	-6.030980	-2.860037	1.316033
H	-4.031597	-4.269165	-0.460953
H	-2.943334	-2.911017	-0.792746
H	-4.687181	-2.645403	-0.778865
H	-1.725728	-3.333123	1.653550
H	-2.824474	-3.727445	2.989684
H	-2.752516	-4.778969	1.562099
C	-1.001611	2.455386	-0.457254
H	-0.958815	3.544878	-0.441100
C	-2.167615	1.800566	-0.795466
C	-4.677046	0.798032	1.124195
C	-4.317437	0.119320	3.443355
C	2.499364	1.948340	0.098218
C	-4.339461	0.421176	4.917852
H	-5.171451	1.092401	5.182669
H	-4.432057	-0.495776	5.520450
H	-3.406549	0.923179	5.230614
O	-3.389131	-0.333353	-0.623177
C	-5.544906	1.607650	0.193457
H	-6.593320	1.425438	0.485334
H	-5.372653	2.688131	0.263864
H	-5.435304	1.305701	-0.849634
C	-3.246114	2.602207	-1.422289
C	-3.541594	3.906494	-0.991298

C	-3.893787	2.113142	-2.570206
C	-4.472549	4.690112	-1.673490
H	-3.057965	4.298511	-0.094401
C	-4.823913	2.897085	-3.253224
H	-3.669339	1.105127	-2.923046
C	-5.121409	4.187655	-2.805568
H	-4.699246	5.696863	-1.313878
H	-5.318715	2.497679	-4.141978
H	-5.852457	4.800509	-3.338371
C	-0.920993	-1.781763	-1.130508
C	-0.347485	-2.724700	-0.266755
C	-1.344177	-2.198029	-2.403932
C	-0.213382	-4.056390	-0.657990
H	0.019414	-2.399285	0.707942
C	-1.205506	-3.530998	-2.798824
H	-1.779253	-1.465708	-3.086935
C	-0.642017	-4.463928	-1.924528
H	0.235801	-4.779804	0.026097
H	-1.538405	-3.840468	-3.792322
H	-0.527079	-5.505582	-2.232728
C	1.613867	-0.129365	-0.811869
C	2.692548	0.648368	-0.423890
H	3.696778	0.265262	-0.579037
N	1.863377	-1.358958	-1.441144
H	1.246733	-1.643621	-2.193812
N	1.297829	2.504592	0.108982
C	3.647480	2.760536	0.581977
C	3.467864	4.132317	0.836460
C	4.915962	2.195276	0.804950

C	4.525915	4.917478	1.290717
H	2.479960	4.561964	0.667762
C	5.973852	2.983219	1.261333
H	5.089130	1.131396	0.641264
C	5.785487	4.346460	1.503835
H	4.368782	5.982522	1.479586
H	6.949596	2.522609	1.430138
H	6.615565	4.961665	1.860185
C	2.798562	-2.308855	-1.114717
O	3.076003	-3.244880	-1.833350
O	3.324343	-2.087529	0.104340
C	4.518674	-2.804427	0.466401
H	4.382159	-3.112933	1.511251
H	4.597861	-3.697701	-0.168137
C	5.716631	-1.902023	0.318809
C	6.386702	-1.403556	1.442680
C	6.139066	-1.502554	-0.959891
C	7.467769	-0.528512	1.295954
H	6.056855	-1.699870	2.441979
C	7.207538	-0.617203	-1.107558
H	5.624594	-1.891497	-1.842412
C	7.876721	-0.130319	0.021149
H	7.983617	-0.149368	2.181410
H	7.524490	-0.309067	-2.106787
H	8.714947	0.560773	-0.095515

Accessing divers axially chiral diaryl ethers through various aromatization approaches

6-(2-(tert-butyl)-4,6-dimethylphenoxy)-5,7-dimethyl-2-phenylquinoline (**5a**)

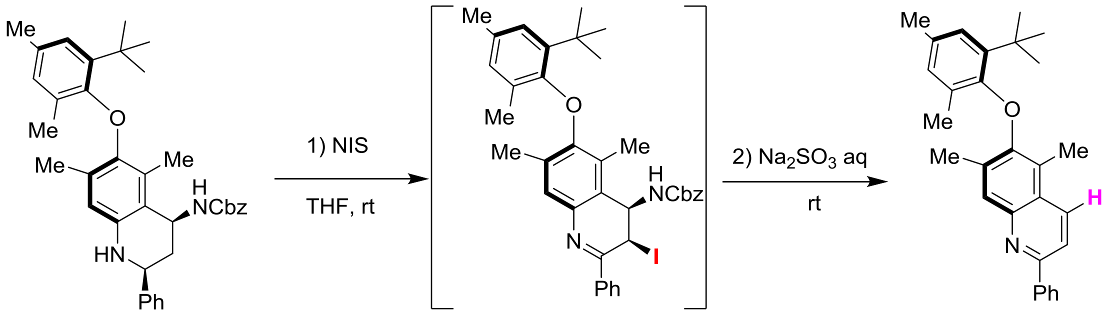

4a', 98% ee, 98: 2 dr

5a', 98% ee, >20: 1 dr

5a, 99%, 94% ee

To a solution of NIS (28 mg, 0.125 mmol, 2.5 equiv.) in THF (0.5 mL) was added dropwise a solution of **4a’** (28.1 mg, 0.05 mmol, 1.0 equiv.) in THF (0.5 mL) while stirring vigorously at rt. After stirring at rt for 5 minutes, 1 mL Na_2SO_3 aq was added into the mixture. Once the solution becomes clear and transparent, it was extracted with EtOAc for 3 times. The combined organic layer was washed with brine, dried over Na_2SO_4 and concentrated under vacuum to give a residue, which was purified by column chromatography (petroleum ether/EtOAc = 20: 1) to give the product **5a** as white foam. (20.2 mg, 99% yield)

The intermediate **5a’** could be isolated from the reaction mixture without quenching with Na_2SO_3, which was purified by column chromatography (petroleum ether: EtOAc =9: 1) as white foam (29.5 mg, 86% yield).

benzyl-((*3R,4R*)-6-(2-(tert-butyl)-4,6-dimethylphenoxy)-3-iodo-5,7-dimethyl-2-phenyl-3,4-dihydroquinolin-4-yl)carbamate (**5a’**)

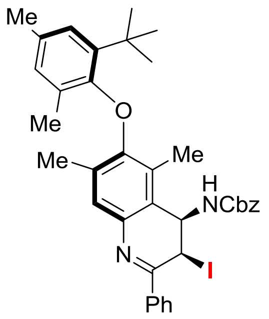

^{1}H NMR (500 MHz, Chloroform-*d*) δ 8.0 – 8.0 (m, 2H), 7.5 – 7.5 (m, 4H), 7.3 – 7.2 (m, 5H), 7.1 (d, *J* = 2.3 Hz, 1H), 6.8 (d, *J* = 2.2 Hz, 1H), 5.7 (d, *J* = 2.2 Hz, 1H), 5.2 (dd, *J* = 7.4, 2.1 Hz, 1H), 5.1 (t, *J* = 8.2 Hz, 2H), 5.0 (d, *J* = 7.2 Hz, 1H), 2.3 (s, 3H), 2.1 (s, 3H), 2.1 (s, 3H), 1.7 (s, 3H), 1.5 (s, 9H). ^{13}C NMR (126 MHz, Chloroform-*d*) δ 160.9, 155.4, 153.9, 152.2, 138.8, 137.9, 136.0, 135.8, 132.0, 131.1, 130.8, 129.5, 129.3, 128.8, 128.7, 128.4, 128.1, 127.2, 127.1, 126.7, 126.2, 121.4, 67.3, 51.3, 35.5, 30.7, 21.0, 18.2, 18.2, 16.9, 12.6. m/z HRMS (ESI) found $[M+H]^+$ 687.2072, $C_{37}H_{40}IN_2O_3^+$ requires 687.2079. $[\alpha]_D^{23}$ = -87.3 (c 0.5, $CHCl_3$). HPLC: Chiralpak IA column, 98:02 hexane/isopropanol, 1 mL/min; t_R= 9.08 min (major); 9.68 min (minor); 98% ee; >20:1 dr.

6-(2-(tert-butyl)-4,6-dimethylphenoxy)-5,7-dimethyl-2-phenylquinoline (**5a**)

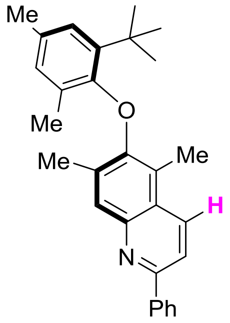

^{1}H NMR (500 MHz, Chloroform-*d*) δ 8.3 (d, *J* = 8.8 Hz, 1H), 8.2 (d, *J* = 7.7 Hz, 2H), 7.9 (s, 1H), 7.8 (d, *J* = 8.8 Hz, 1H), 7.5 (t, *J* = 7.5 Hz, 2H), 7.5 – 7.4 (m, 1H), 7.1 (d, *J* = 2.3 Hz, 1H), 6.8 (d, *J* = 2.2 Hz, 1H), 2.4 (s, 3H), 2.3 (s, 3H), 2.3 (s, 3H), 1.7 (s, 3H), 1.5 (s, 9H). ^{13}C NMR (126 MHz, Chloroform-*d*) δ 155.3, 152.7, 151.7, 145.3, 139.9,

139.1, 133.9, 132.5, 132.0, 130.7, 129.8, 129.1, 129.0, 128.9, 127.6, 127.4, 127.1, 126.6, 126.2, 119.3, 118.2, 35.5, 30.6, 21.1, 19.3, 18.2, 12.3. m/z HRMS (ESI) found $[M+H]^+$ 410.2472, $C_{29}H_{32}NO^+$ requires 410.2479. $[\alpha]_D^{23}$ = -76.6 (c 1.0, $CHCl_3$). HPLC: Chiralpak IA column, 100:0 hexane/isopropanol, 1 mL/min; t_R= 5.19 min (minor); 6.07 min (major); 94% ee.

benzyl ((*2S,4S*)-8-bromo-6-(2-(tert-butyl)-4,6-dimethylphenoxy)-5,7-dimethyl-2-phenyl-1,2,3,4-tetrahydroquinolin-4-yl)carbamate (**6a**)

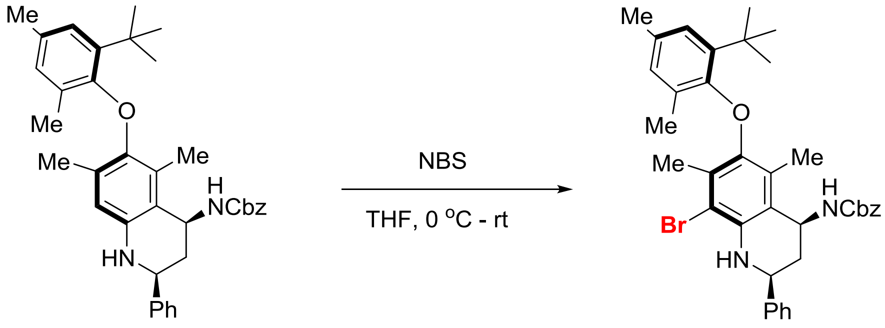

4a', 98% ee, 98: 2 dr

6a, 94%, 99% ee, 98: 2 dr

To a solution of **4a'** (56.3 mg, 0.1 mmol, 1.0 equiv.) in THF (0.5 mL) was added a solution of NBS (18.7 mg, 0.105 mmol, 1.05 equiv.) in THF dropwise at 0 °C under vigorous stirring conditions. After stirring at 0 °C for 20 min, the reaction mixture was warmed up to room temperature slowly and allowed to stir for another 10 min. After completion of the reaction as monitored by TLC analysis, the mixture was concentrated under vacuum to give a residue, which was purified by column chromatography (petroleum ether/EtOAc = 8: 1) to give the product **6a** as white foam (60.5 mg, 94% yield).

^{1}H NMR (500 MHz, Chloroform-*d*) δ 8.6 (s, 1H), 8.4 – 8.3 (m, 2H), 7.6 – 7.5 (m, 3H), 7.5 – 7.3 (m, 6H), 7.1 (d, *J* = 2.3 Hz, 1H), 6.7 (d, *J* = 2.2 Hz, 1H), 5.3 – 5.2 (m, 2H), 2.6 (s, 3H), 2.5 (s, 3H), 2.3 (s, 3H), 1.6 (s, 3H), 1.5 (s, 9H). ^{13}C NMR (126 MHz, Chloroform-*d*) δ 155.8, 152.9, 152.5, 152.4, 144.1, 142.8, 138.8, 138.2, 135.7, 134.7,

131.8, 131.4, 129.7, 128.9, 128.8, 128.7, 128.6, 128.4, 127.6, 126.7, 125.2, 120.1, 118.3, 110.3, 67.8, 35.6, 30.7, 21.0, 19.9, 18.8, 16.7. m/z HRMS (ESI) found $[M+H]^+$ 641.2362, $C_{37}H_{42}BrN_2O_3^+$ requires 641.2374. $[\alpha]_D^{23}$ = -31.9 (c 0.5, $CHCl_3$). HPLC: Chiralpak IA column, 90:10 hexane/isopropanol, 1 mL/min; t_R= 5.96 min (major); 7.14 min (minor); 99% ee; 98:2 dr.

benzyl (8-bromo-6-(2-(tert-butyl)-4,6-dimethylphenoxy)-5,7-dimethyl-2-phenylquinolin-4-yl)carbamate (**7a**)

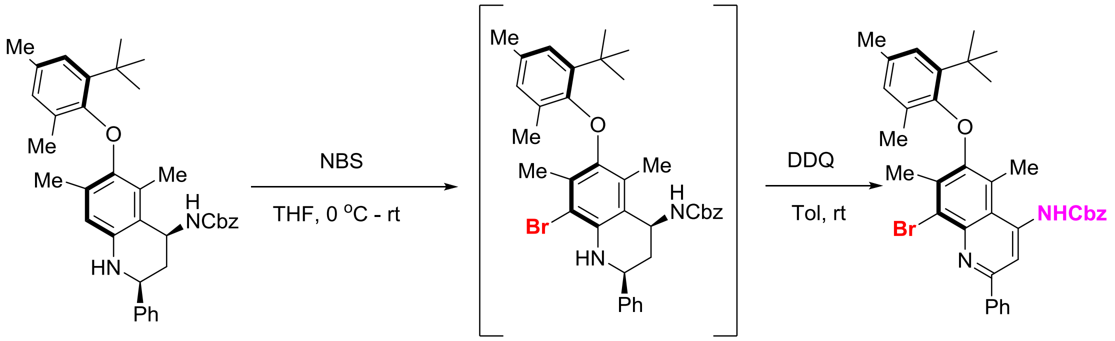

4a', 98% ee, 98: 2 dr **6a** **7a**, 88%, 94% ee

To a solution of **4a'** (56.3 mg, 0.1 mmol, 1.0 equiv.) in THF (0.5 mL) was added a solution of NBS (18.7 mg, 0.105 mmol, 1.05 equiv.) in THF (0.5 mL) dropwise at 0 °C under vigorous stirring conditions. After stirring at 0 °C for 20 min, the reaction mixture was warmed up to room temperature slowly and allowed to stir for another 10 min. After completion of the reaction as monitored by TLC analysis, the mixture was concentrated under vacuum to give a residue, which was purified by column chromatography (petroleum ether/EtOAc = 8: 1) to give the product **6a** for the next step.

To a solution of the above product **6a** in toluene (1 mL) was added 1,2-dichloro-4,5-dicyanobenzoquinone (DDQ, 0.21 mmol, 2.1 equiv.) in portions at rt. After stirring for 2 h, the reaction mixture was diluted with EtOAc and then washed

with saturated aqueous Na_2SO_3 for two times and saturated aqueous $NaHCO_3$ solution for two times. The organic layer was combined, dried over Na_2SO_4 and concentrated under vacuum to give a residue, which was purified by flash column chromatography (petroleum ether/EtOAc 12:1) to give the product **7a** as white foam (56.4 mg, 88% yield for two steps).

^{1}H NMR (500 MHz, Chloroform-*d*) δ 8.6 (s, 1H), 8.4 – 8.3 (m, 2H), 7.6 – 7.5 (m, 3H), 7.5 – 7.4 (m, 6H), 7.1 (d, *J* = 2.3 Hz, 1H), 6.7 (d, *J* = 2.2 Hz, 1H), 5.3 – 5.2 (m, 2H), 2.6 (s, 3H), 2.5 (s, 3H), 2.3 (s, 3H), 1.6 (s, 3H), 1.5 (s, 9H). ^{13}C NMR (126 MHz, Chloroform-*d*) δ 155.8, 152.9, 152.5, 152.4, 144.1, 142.8, 138.8, 138.2, 135.7, 134.7, 131.8, 131.4, 129.7, 128.9, 128.8, 128.7, 128.6, 128.4, 127.6, 126.7, 125.2, 120.1, 118.3, 110.3, 67.8, 35.6, 30.7, 21.0, 19.9, 18.8, 16.7. m/z HRMS (ESI) found $[M+H]^+$ 637.2051, $C_{37}H_{38}BrN_2O_3^+$ requires 637.2061. $[\alpha]_D^{23}$ = +33.5 (c 1.0, $CHCl_3$). HPLC: Chiralpak IC column, 95:05 hexane/isopropanol, 1 mL/min; t_R= 6.66 min (major); 7.35 min (minor); 94% ee.

8-bromo-6-(2-(tert-butyl)-4,6-dimethylphenoxy)-5,7-dimethyl-2-phenylquinoline (**8a**)

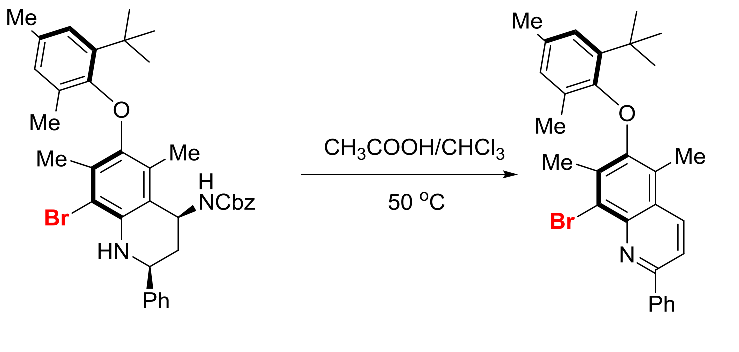

6a, 99% ee, 98: 2 dr

8a, 88%, 93% ee

To a solution of **6a** (32.1 mg, 0.05 mmol) in $CHCl_3$ (0.5 mL) was added CF_3COOH (0.5 mL) at rt. Then the reaction mixture was warmed up to 50 °C and allowed to stir

for 4 hours. After completion of the reaction as monitored by TLC analysis, the mixture was concentrated under vacuum to give a residue, which was purified by column chromatography (petroleum ether/EtOAc = 30: 1) to give the product **8a** as white foam (21.5 mg, 88% yield).

^{1}H NMR (400 MHz, Chloroform-*d*) δ 8.4 – 8.3 (m, 2H), 8.3 (d, *J* = 8.8 Hz, 1H), 7.9 (d, *J* = 8.8 Hz, 1H), 7.6 – 7.5 (m, 2H), 7.5 – 7.4 (m, 1H), 7.1 (d, *J* = 2.4 Hz, 1H), 6.7 (d, *J* = 2.3 Hz, 1H), 2.7 (s, 3H), 2.3 (s, 3H), 2.2 (s, 3H), 1.6 (s, 3H), 1.5 (s, 9H). ^{13}C NMR (101 MHz, Chloroform-*d*) δ 155.4, 152.8, 151.5, 142.4, 139.2, 138.7, 135.0, 133.1, 132.1, 131.0, 129.5, 129.0, 127.5, 126.9, 126.7, 126.4, 118.8, 118.3, 35.5, 30.6, 21.1, 19.7, 18.4, 12.5. m/z HRMS (ESI) found $[M+H]^+$ 488.1574, $C_{29}H_{31}BrNO^+$ requires 488.1584. $[\alpha]_D^{23}$ = -95.6 (c 1.0, $CHCl_3$). HPLC: Chiralpak IA column, 100:0 hexane/isopropanol, 0.5 mL/min; t_R= 10.22 min (minor); 10.71 min (major); 93% ee.

3,8-dibromo-6-(2-(tert-butyl)-4,6-dimethylphenoxy)-5,7-dimethyl-2-phenylquinoline (**9a**)

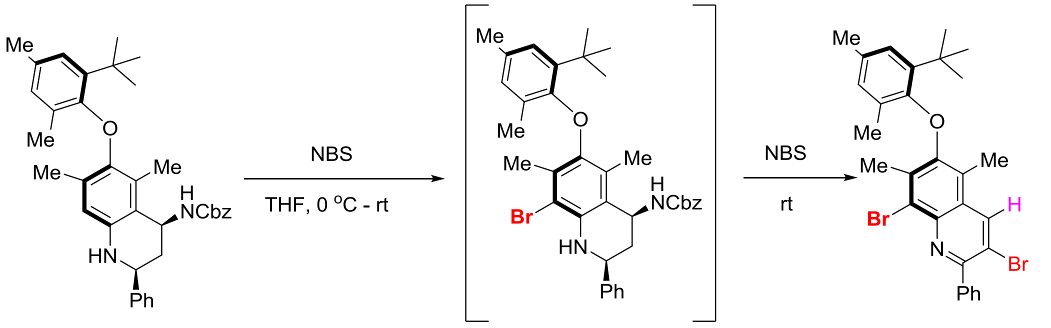

4a', 98% ee, 98: 2 dr **6a** **9a**,74%, 95% ee

To a solution of **4a'** (28.1 mg, 0.05 mmol, 1.0 equiv.) in THF (0.5 mL) was added a solution of NBS (9.08 mg, 0.051 mmol, 1.05 equiv.) in THF (0.5 mL) dropwise at 0 °C under vigorous stirring conditions. After stirring at 0 °C for 20 min, the reaction mixture was warmed up to room temperature slowly and allowed to stir for another 10 min. After completion of the reaction as monitored by TLC analysis, another 1.0 equiv.

of NBS (8.9 mg) was added into the reaction. After stirring for another 12 hours, the reaction mixture was concentrated under vacuum to give a residue, which was purified by flash column chromatography (petroleum ether/EtOAc = 12:1) to give the product **9a** as white foam. (21.0 mg, 74% yield)

^{1}H NMR (500 MHz, Chloroform-*d*) δ 8.5 (s, 1H), 8.0 – 7.9 (m, 2H), 7.5 – 7.4 (m, 3H), 7.1 (d, *J* = 2.2 Hz, 1H), 6.8 (d, *J* = 2.2 Hz, 1H), 2.7 (s, 3H), 2.3 (s, 3H), 2.1 (s, 3H), 1.6 (s, 3H), 1.5 (s, 9H). ^{13}C NMR (126 MHz, Chloroform-*d*) δ 155.8, 152.6, 152.1, 141.2, 139.5, 138.9, 136.7, 135.7, 132.5, 130.9, 130.2, 129.1, 128.1, 128.0, 126.5, 126.5, 126.2, 117.4, 35.5, 30.6, 21.1, 19.8, 18.3, 12.5. m/z HRMS (ESI) found $[M+H]^+$ 568.0678, $C_{29}H_{30}Br_2NO^+$ requires 568.0669. $[\alpha]_D^{22}$ = -81.0 (c 1.0, $CHCl_3$). HPLC: Chiralpak ID column, 100:0 hexane/isopropanol, 1 mL/min; t_R= 5.47 min (minor); 8.45 min (major); 95% ee.

Large-scale asymmetric reaction

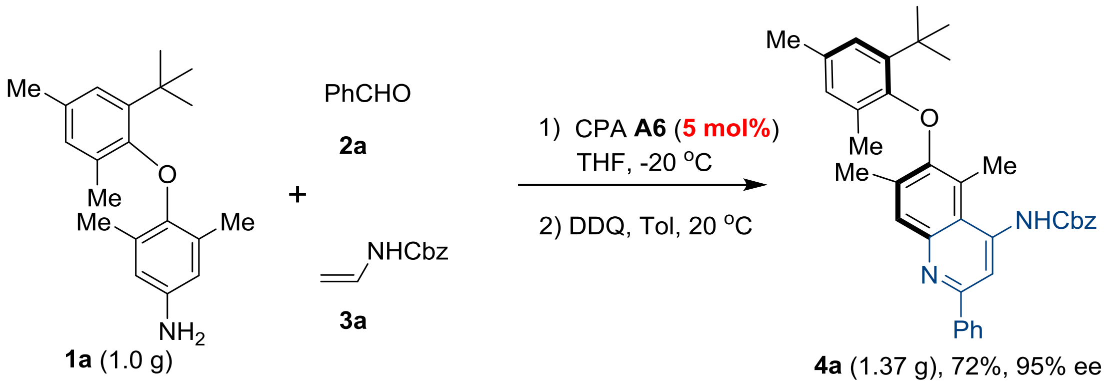

To a stirred solution of **1a** (1.01g, 3.4 mmol, 1.0 equiv.), CPA (*R*)-**A6** (99 mg, 5 mol%) and activated 4Å molecular sieves (ca. 1.0 g) in THF (17 mL) was added the corresponding aldehyde **2a** (0.52 mL, 5.1 mmol, 1.5 equiv.) at room temperature and stirred at -20 °C for 30 min. After that, a solution of enamide **3a** (1.2 g. 6.8 mmol, 2.0 equiv.) in THF (17 mL) was added dropwise into the reaction mixture via syringe and the mixture was allowed to stir at the same temperature for another 12 h. After the

completion of the Povarov reaction as monitored by TLC analysis, the reaction was quenched by NEt_3 (0.1 mL) and the solvent was concentrated under vacuum to give a residue, which was purified by flash column chromatography (petroleum ether/EtOAc = 9:1) to give the product **4a'**.

To a solution of the above product **4a'** in toluene (34 mL) was added 1,2-dichloro-4,5-dicyanobenzoquinone (DDQ, 7.1 mmol, 2.1 equiv.) in portions at rt. After stirring for 2 h, the reaction mixture was diluted with EtOAc, and washed with saturated aqueous Na_2SO_3 for two times and saturated aqueous $NaHCO_3$ solution for two times. The organic layer was combined, dried over Na_2SO_4 and concentrated under vacuum to give a residue, which was purified by flash column chromatography (petroleum ether/EtOAc = 12:1) to give the product **4a** as white foam (1.37 g, 72% yield, 95% ee).

Derivatizations of the chiral products

6-(2-(tert-butyl)-4,6-dimethylphenoxy)-5,7-dimethyl-2-phenylquinolin-4-amine (**10a**)

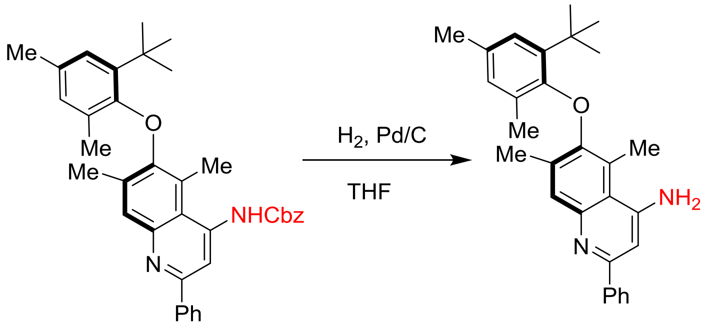

4a, 95% ee **10a**, 99%, 95% ee

To a solution of **4a** (335.2 mg, 0.6 mmol) in THF (10 mL) was added Pd/C (50 mg, 10 wt %) at rt, and the flask was evacuated and purged with H_2 for 3 times. After stirring under H_2 atmosphere (1 atm) for 12 hours, the mixture was filtered through Celite and concentrated under vacuum to give a residue, which was purified by column chromatography (petroleum ether/EtOAc = 2: 1) to give the product **10a** as white

foam (254.7 mg, 99% yield).

^{1}H NMR (500 MHz, Chloroform-*d*) δ 8.04 (d, *J* = 7.3 Hz, 2H), 7.74 (s, 1H), 7.47 (t, *J* = 7.5 Hz, 2H), 7.41 (t, *J* = 7.3 Hz, 1H), 7.10 (d, *J* = 2.3 Hz, 1H), 6.90 (s, 1H), 6.73 (d, *J* = 2.2 Hz, 1H), 4.94 (s, 2H), 2.76 (s, 3H), 2.31 (s, 3H), 2.17 (s, 3H), 1.67 (s, 3H), 1.52 (s, 9H). ^{13}C NMR (126 MHz, Chloroform-*d*) δ 156.0, 152.6, 152.4, 151.2, 147.3, 139.9, 138.6, 132.8, 131.5, 131.0, 130.4, 128.9, 128.7, 127.2, 126.3, 126.2, 120.6, 118.0, 103.2, 35.5, 30.7, 21.0, 19.0, 18.5, 16.3. m/z HRMS (ESI) found $[M+H]^+$ 425.2584, $C_{29}H_{33}N_2O^+$ requires 425.2588. $[\alpha]_D^{22}$ = +94.5 (c 1.0, $CHCl_3$). HPLC: Chiralpak IB-N column, 80:20 hexane/isopropanol, 1 mL/min; t_R= 6.38 min (major); 9.09 min (minor); 95% ee.

Sandmayer reactions of **10a**

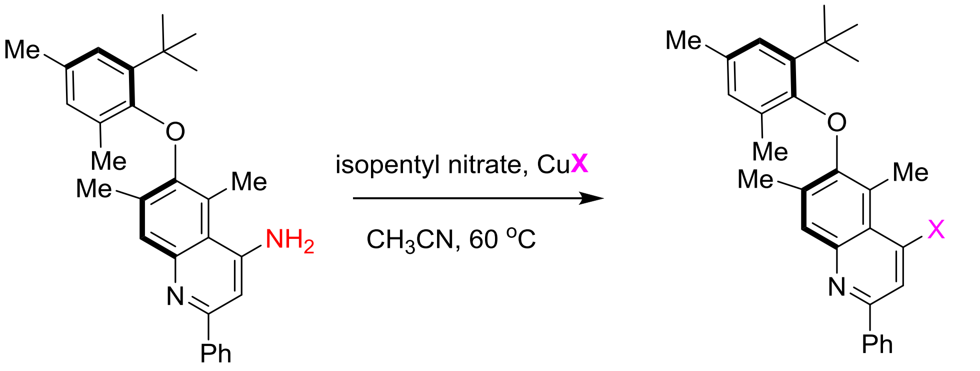

10a, 95% ee

11a, X = Br, 70%, 95% ee
12a, X = I, 46%, 96% ee

To a solution of **10a** (21.2 mg, 0.05 mmol, 1.0 equiv.), CuX (0.1 mmol, 2.0 equiv.) in CH_3CN (0.5 mL) was a solution of isopentyl nitrite (20.2 μL, 0.15 mmol, 3.0 equiv.) at rt, and the flask was allowed to watm to 60 °C. After stirring at 60 °C under N_2 atmosphere (1 atm) for 16 hours, the mixture was filtered through Celite and concentrated under vacuum to give a residue, which was purified by column chromatography (petroleum ether/EtOAc = 20: 1) to give the Sandmayer product **11a** (17.2 mg, 70% yield, yellow oil) and **12a** (12.4 mg, 46% yield, yellow solid).

4-bromo-6-(2-(tert-butyl)-4,6-dimethylphenoxy)-5,7-dimethyl-2-phenylquinoline (**11a**)

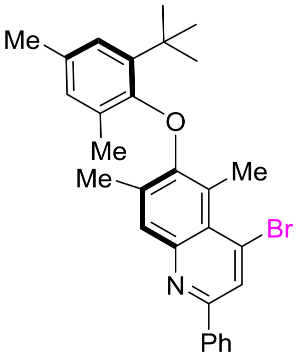

^{1}H NMR (500 MHz, Chloroform-*d*) δ 7.84 (s, 1H), 7.52 (t, *J* = 7.5 Hz, 2H), 7.49 – 7.42 (m, 1H), 7.09 (d, *J* = 2.3 Hz, 1H), 6.74 (d, *J* = 2.3 Hz, 1H), 2.84 (s, 3H), 2.31 (s, 3H), 2.22 (s, 3H), 1.66 (s, 3H), 1.50 (s, 9H). ^{13}C NMR (126 MHz, Chloroform-*d*) δ 154.8, 153.4, 152.3, 147.0, 138.7, 138.2, 134.0, 131.9, 131.0, 130.9, 130.9, 129.6, 129.0, 127.3, 126.4, 125.6, 125.5, 122.4, 35.5, 30.7, 21.0, 19.2, 18.5, 17.2. m/z HRMS (ESI) found $[M+H]^+$ 488.1574, $C_{29}H_{31}BrNO^+$ requires 488.1584. $[\alpha]_D^{22}$ = +48.9 (c 1.0, $CHCl_3$). HPLC: Chiralpak IB-N column, 100:0 hexane/isopropanol, 1 mL/min; t_R= 8.91 min (major); 13.66 min (minor); 95% ee.

6-(2-(tert-butyl)-4,6-dimethylphenoxy)-4-iodo-5,7-dimethyl-2-phenylquinoline (**12a**)

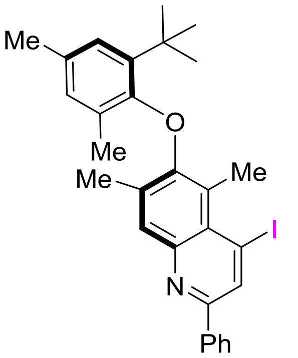

^{1}H NMR (500 MHz, Chloroform-*d*) δ 8.56 (s, 1H), 8.10 (dd, *J* = 7.2, 1.8 Hz, 2H), 7.81 (s, 1H), 7.51 (t, *J* = 7.5 Hz, 2H), 7.45 (t, *J* = 7.3 Hz, 1H), 7.09 (d, *J* = 2.3 Hz, 1H), 6.75 (d, *J* = 2.2 Hz, 1H), 2.85 (s, 3H), 2.31 (s, 3H), 2.22 (s, 3H), 1.65 (s, 3H), 1.49 (s,

9H). ^{13}C NMR (126 MHz, Chloroform-*d*) δ 154.6, 153.3, 152.2, 145.8, 138.7, 137.9, 134.1, 133.7, 132.0, 131.2, 130.8, 129.5, 129.0, 127.6, 127.4, 126.5, 126.4, 121.9, 102.7, 35.5, 30.7, 19.2, 18.5, 18.0. m/z HRMS (ESI) found $[M+H]^+$ 536.1437, $C_{29}H_{31}INO^+$ requires 536.1445. $[\alpha]_D^{22}$ = +58.2 (c 1.0, $CHCl_3$). HPLC: Chiralpak IB-N column, 100:0 hexane/ isopropanol, 1 mL/min; t_R= 11.59 min (major); 15.93 min (minor); 96% ee.

3-bromo-6-(2-(tert-butyl)-4,6-dimethylphenoxy)-5,7-dimethyl-2-phenylquinolin-4-amine (**13a**)

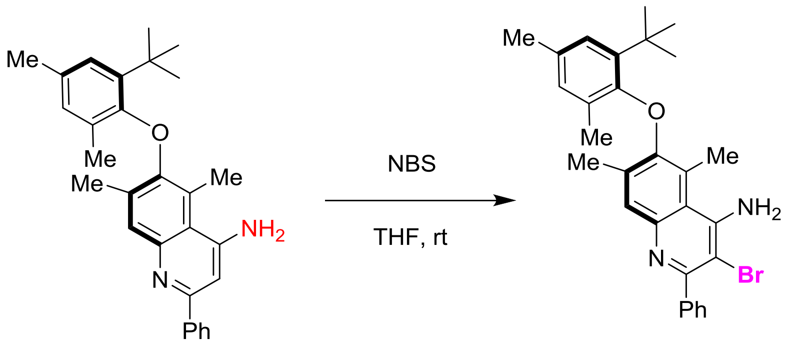

10a, 95% ee

13a, 97%, 95% ee

To a solution of **10a** (42.5 mg, 0.1 mmol, 1.0 equiv.) in THF (0.5 mL) was added a solution of NBS (17.8 mg, 0.1 mmol, 1.0 equiv.) in THF (0.5 mL) slowly under vigorous stirring conditions at rt. After stirring at rt for another 30 min, the mixture was concentrated under vacuum to give a residue, which was purified by column chromatography (petroleum ether/EtOAc = 10: 1) to give the product **13a** as white foam (48.9 mg, 97% yield).

^{1}H NMR (500 MHz, Chloroform-*d*) δ 7.7 (s, 1H), 7.7 – 7.6 (m, 2H), 7.5 – 7.4 (m, 3H), 7.1 (d, *J* = 2.3 Hz, 1H), 6.7 (d, *J* = 2.3 Hz, 1H), 5.6 (s, 2H), 2.8 (s, 3H), 2.3 (s, 3H), 2.1 (s, 3H), 1.7 (s, 3H), 1.5 (s, 9H). ^{13}C NMR (126 MHz, Chloroform-*d*) δ 157.0, 152.2, 152.0, 148.7, 144.6, 141.5, 138.7, 132.9, 131.8, 130.9, 130.6, 129.2, 128.4, 128.0, 126.4, 126.3, 119.9, 118.0, 102.1, 35.5, 30.7, 21.0, 19.1, 18.5, 16.5. m/z HRMS (ESI) found $[M+H]^+$ 503.1667, $C_{29}H_{32}BrN_2O^+$ requires 503.1693. $[\alpha]_D^{21}$ = +121.7 (c

1.0, $CHCl_3$). HPLC: Chiralpak IA column, 95:05 hexane/ isopropanol, 1 mL/min; t_R= 7.53 min (minor); 8.46 min (major); 95% ee.

6-(2-(tert-butyl)-4,6-dimethylphenoxy)-3-iodo-5,7-dimethyl-2-phenylquinolin-4-amine (**14a**)

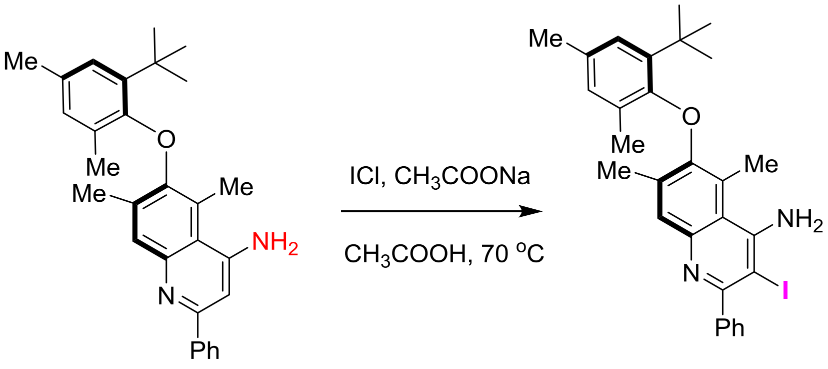

10a, 95% ee

14a, 99%, 94% ee

To a solution of **10a** (127.4 mg, 0.3 mmol, 1.0 equiv.) and CH_3COONa (49 mg, 0.6 mmol, 2.0 equiv.) in CH_3COOH (1 mL) was a solution of ICl (58.4 mg, 0.36 mmol, 1.2 equiv.) in CH_3COOH (1 mL) at 70 ^oC. After stirring at the same temperature for 12 hours, the mixture was diluted with EtOAc and washed with a saturated aqueous solution of $NaHCO_3$. The combined organic layer was washed with brine, dried over Na_2SO_4 and concentrated under vacuum to give a residue, which was purified by column chromatography (petroleum ether/EtOAc = 10: 1) to give the product **14a** as yellow foams (163.8 mg, 99% yield).

^{1}H NMR (500 MHz, Chloroform-*d*) δ 7.7 (s, 1H), 7.6 – 7.5 (m, 2H), 7.4 (dt, *J* = 14.1, 6.8 Hz, 3H), 7.1 (d, *J* = 2.3 Hz, 1H), 6.7 (d, *J* = 2.3 Hz, 1H), 5.8 (s, 2H), 2.8 (s, 3H), 2.3 (s, 3H), 2.1 (s, 3H), 1.7 (s, 3H), 1.5 (s, 9H). ^{13}C NMR (126 MHz, Chloroform-*d*) δ 160.6, 152.2, 152.0, 151.5, 145.1, 144.2, 138.7, 133.3, 131.8, 130.9, 130.2, 129.1, 128.4, 128.0, 126.5, 126.4, 119.7, 117.3, 80.4, 35.5, 30.7, 21.0, 19.1, 18.6, 16.9. m/z HRMS (ESI) found $[M+H]^+$ 551.1545, $C_{29}H_{32}IN_2O^+$ requires 551.1554. $[\alpha]_D^{21}$ =

+73.7 (c 1.0, $CHCl_3$). HPLC: Chiralpak IA column, 95:05 hexane/ isopropanol, 1 mL/min; t_R= 7.35 min (minor); 9.06 min (major); 94% ee.

6-(2-(tert-butyl)-4,6-dimethylphenoxy)-5,7-dimethyl-2,3-diphenylquinolin-4-amine (**15a**)

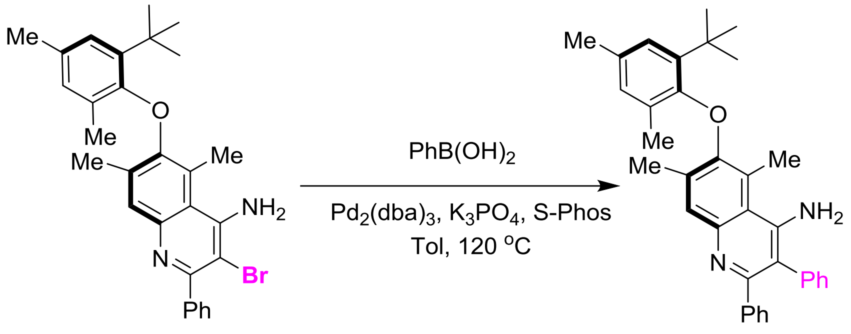

13a, 95% ee

15a, 93%, 94% ee

To a flask equipped with **13a** (50.4 mg, 0.1 mmol, 1.0 equiv.), phenylboronic acid (24.4 mg, 0.2 mmol, 2.0 equiv.), $Pd_2(dba)_3$ (9.2 mg, 0.01 mmol, 10 mol%), S-Phos (8.2 mg, 0.02 mmol, 20 mol%) and K_3PO_4 (169.8 mg, 0.8 mmol, 8.0 equiv.) was added toluene (5 mL) at rt under N_2 atmosphere (1 atm). After stirring at 120 °C for 12 hours, the mixture was filtered through Celite and concentrated under vacuum to give a residue, which was purified by column chromatography (petroleum ether/EtOAc = 6: 1) to give the product **15a** as yellow foam (46.6 mg, 93% yield).

^{1}H NMR (500 MHz, Chloroform-*d*) δ 7.8 (s, 1H), 7.4 – 7.3 (m, 4H), 7.3 (dt, *J* = 19.2, 6.5 Hz, 3H), 7.2 (dd, *J* = 5.3, 2.0 Hz, 3H), 7.1 (d, *J* = 2.3 Hz, 1H), 6.8 (d, *J* = 2.2 Hz, 1H), 4.9 (s, 2H), 2.8 (s, 3H), 2.3 (s, 3H), 2.2 (s, 3H), 1.8 (s, 3H), 1.5 (s, 9H). ^{13}C NMR (126 MHz, Chloroform-*d*) δ 157.0, 152.4, 151.7, 149.5, 145.6, 141.3, 138.7, 136.9, 132.6, 131.7, 131.6, 131.5, 130.9, 130.5, 129.7, 129.2, 127.6, 127.5, 127.3, 126.4, 120.5, 117.6, 117.0, 35.5, 30.7, 21.0, 19.1, 18.6, 16.5. m/z HRMS (ESI) found $[M+H]^+$ 501.2893, $C_{35}H_{37}N_2O^+$ requires 501.2901. $[\alpha]_D^{21}$ = -142.3 (c 1.0, $CHCl_3$).

HPLC: Chiralpak IA column, 95:05 hexane/isopropanol, 1 mL/min; t_R= 8.38 min (minor); 11.98 min (major); 94% ee.

8-(2-(tert-butyl)-4,6-dimethylphenoxy)-7,9-dimethyl-4-phenyl-1H-pyrrolo[3,2-c]quinoline (**16a**)

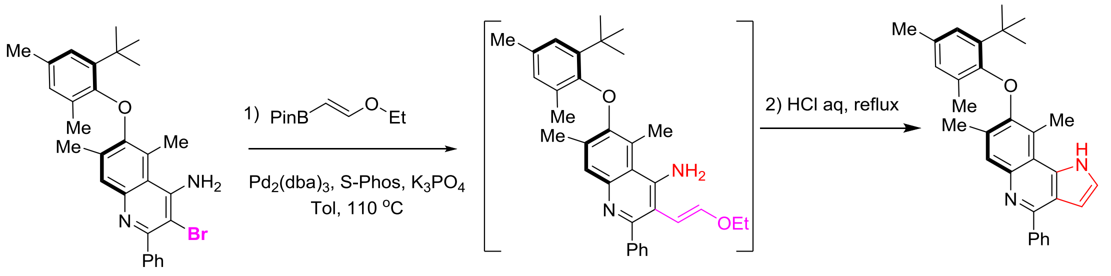

13a, 95% ee

16a, 61%, 94% ee

To a flask equipped with **13a** (50.4 mg, 0.1 mmol, 1.0 equiv.), (*E*)-1-Ethoxyethene-2-ylboronic acid pinacol ester (0.21 mL, 1.0 mmol, 10.0 equiv.), $Pd_2(dba)_3$ (9.2 mg, 0.01 mmol, 10 mol%), S-Phos (8.2 mg, 0.02 mmol, 20 mol%) and K_3PO_4 (254.78 mg, 1.2 mmol, 12.0 equiv.) was added toluene (5 mL) at rt under N_2 atmosphere (1 atm). After stirring at 110 ^{o}C for 12 hours, 2 M HCl aq solution (1 mL) was added into the reaction mixture, and was allowed to continue stirring for another 2 h. Then the mixture was then cooled to room temperature and diluted with EtOAc. The organic layer was washed with brine, dried over Na_2SO_4 and concentrated under vacuum to give a residue, which was purified by column chromatography (petroleum ether/EtOAc = 4: 1) to give the product **16a** as a yellow solid (27.2 mg, 61% yield).

^{1}H NMR (500 MHz, Chloroform-*d*) δ 9.4 (s, 1H), 8.1 – 8.0 (m, 2H), 8.0 (s, 1H), 7.6 (t, *J* = 7.5 Hz, 2H), 7.5 – 7.4 (m, 1H), 7.3 (t, *J* = 2.8 Hz, 1H), 7.1 (d, *J* = 2.3 Hz, 1H), 7.0 (dd, *J* = 3.2, 1.9 Hz, 1H), 6.7 (d, *J* = 2.2 Hz, 1H), 2.8 (s, 3H), 2.3 (s, 3H), 2.3 (s, 3H), 1.7 (s, 3H), 1.5 (s, 9H). ^{13}C NMR (126 MHz, Chloroform-*d*) δ 153.2, 152.7, 151.5, 138.9, 136.3, 131.7, 130.9, 130.7, 129.5, 129.2, 128.8, 128.7, 126.7, 126.3, 123.1, 119.5, 118.0, 116.9, 104.5, 35.5, 30.7, 21.0, 19.0, 18.4, 15.6. m/z HRMS (ESI) found $[M+H]^+$ 449.2576, $C_{31}H_{33}N_2O^+$ requires 449.2588. $[\alpha]_D^{23}$ = +49.0 (c 1.0, $CHCl_3$).

HPLC: Chiralpak IB-N column, 90:10 hexane/isopropanol, 1 mL/min; t_R= 7.52 min (major); 8.51 min (minor); 94% ee.

6-(2-(tert-butyl)-4,6-dimethylphenoxy)-5,7-dimethyl-2-phenyl-3-(phenylethynyl)quinolin-4-amine (**17a**)

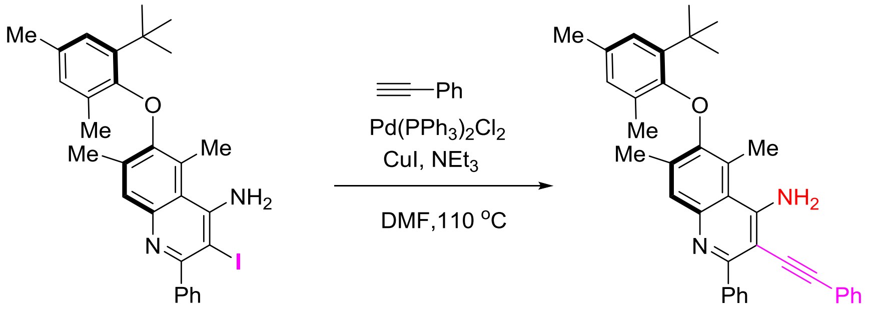

14a, 94% ee **17a**, 58%, 94% ee

To a flask equipped with **14a** (55.0 mg, 0.1 mmol, 1.0 equiv.), CuI (3.8 mg, 0.02 mmol, 20 mol%) and $Pd(PPh_3)_2Cl$ (14 mg, 0.02 mmol, 20 mol%) was added a solution of NEt_3 (70 μL, 0.5 mmol, 5.0 equiv.) in DMF (1 mL) under N_2 atmosphere (1 atm) at room temperature. The reaction mixture was then allowed to warm to 110 °C and a solution of ethynylbenzene (33 μL, 0.3 mmol, 3.0 equiv.) in DMF (1 mL) was added slowly under stirring conditions. After stirring at 110 °C for 12 hours, the mixture was cooled to room temperature and diluted with EtOAc. The organic layer was washed with brine, dried over Na_2SO_4 and concentrated under vacuum to give a residue, which was purified by column chromatography (petroleum ether/EtOAc = 8:1) to give the product **17a** as yellow foam (30.4 mg, 58% yield).

^{1}H NMR (500 MHz, Chloroform-*d*) δ 8.0 – 8.0 (m, 2H), 7.7 (s, 1H), 7.5 (dd, *J* = 8.2, 6.5 Hz, 2H), 7.5 – 7.4 (m, 1H), 7.4 – 7.4 (m, 2H), 7.3 – 7.3 (m, 3H), 7.1 (d, *J* = 2.3 Hz, 1H), 6.7 (d, *J* = 2.3 Hz, 1H), 5.9 (s, 2H), 2.8 (s, 3H), 2.3 (s, 3H), 2.2 (s, 3H), 1.7 (s, 3H), 1.5 (s, 9H). ^{13}C NMR (126 MHz, Chloroform-*d*) δ 158.2, 153.6, 152.3, 151.8, 145.2, 140.6, 138.7, 133.4, 131.7, 131.0, 131.0, 130.9, 129.5, 128.7, 128.5, 128.3, 127.9, 126.4, 126.3, 123.5, 120.5, 116.1, 100.3, 97.9, 85.5, 35.5, 30.7, 21.0, 19.1, 18.6, 16.4. m/z HRMS (ESI) found $[M+H]^+$ 525.2897, $C_{37}H_{37}N_2O^+$ requires 525.2901.

$[\alpha]_D^{23}$ = +137.4 (c 0.5, $CHCl_3$). HPLC: Chiralpak IB-N column, 90:10 hexane/isopropanol, 1 mL/min; t_R= 7.10 min (minor); 8.21 min (major); 94% ee.

8-(2-(tert-butyl)-4,6-dimethylphenoxy)-7,9-dimethyl-2,4-diphenyl-1H-pyrrolo[3,2-c]quinoline (**18a**)

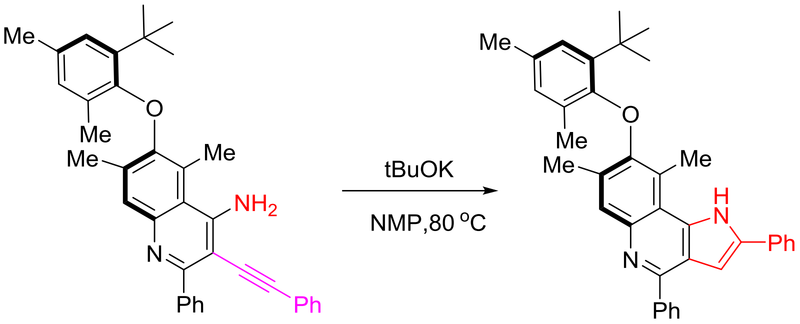

17a, 94% ee **18a**, 92%, 94% ee

To a solution of **17a** (26.2 mg, 0.05 mmol, 1.0 equiv.) in NMP (1 mL) was added tBuOK (14 mg, 0.125 mmol, 2.5 equiv.) at rt, and the reaction was allowed to warm up to 80 °C . After stirring at 80 °C for 12 hours, the mixture was cooled to room temperature and diluted with EtOAc. The organic layer was washed with brine, dried over Na_2SO_4 and concentrated under vacuum to give a residue, which was purified by column chromatography (petroleum ether/EtOAc = 8:1) to give the product **18a** as yellow foam (24.2 mg, 92% yield).

^{1}H NMR (500 MHz, Chloroform-*d*) δ 9.5 (s, 1H), 8.1 (d, *J* = 7.5 Hz, 2H), 8.0 (s, 1H), 7.7 (d, *J* = 7.7 Hz, 2H), 7.6 (t, *J* = 7.5 Hz, 2H), 7.5 (t, *J* = 7.3 Hz, 1H), 7.5 (t, *J* = 7.5 Hz, 2H), 7.4 (t, *J* = 7.4 Hz, 1H), 7.2 (d, *J* = 2.0 Hz, 1H), 7.1 (d, *J* = 2.2 Hz, 1H), 6.8 (s, 1H), 2.8 (s, 3H), 2.3 (s, 3H), 2.3 (s, 3H), 1.7 (s, 3H), 1.5 (s, 9H). ^{13}C NMR (126 MHz, Chloroform-*d*) δ 153.1, 152.8, 151.7, 141.5, 140.4, 138.9, 137.2, 136.8, 131.9, 131.8, 130.9, 129.7, 129.4, 129.1, 128.8, 128.7, 128.1, 126.8, 126.3, 125.1, 121.0, 117.6, 116.6, 101.3, 35.5, 30.7, 21.1, 19.0, 18.4, 15.8. m/z HRMS (ESI) found $[M+H]^+$ 525.2911, $C_{37}H_{37}N_2O^+$ requires 525.2901. $[\alpha]_D^{22}$ = +92.2 (c 1.0, $CHCl_3$). HPLC:

Chiralpak IA column, 90:10 hexane/ isopropanol, 1 mL/min; t_R= 6.03 min (minor); 6.77 min (major); 94% ee.

Reference

[1] Bao, H.; Chen, Y.; Yang, X. *Angew. Chem., Int. Ed.* **2023**, *62*, e202300481.

X-Ray structures

The compound **4b** (5 mg) was dissolved in a mixed solvent of 10:1 n-hexane:DCM solvent (0.5mL) at room temperature, which was filtered through the filter membrane into a vial. The single crystal of **4b** was obtained by slowly evaporating the solvent at room temperature.

Crystal measurement: X-ray crystal structures of (*R*)-**4b** were determined at 173 K by using Bruker APEX-II CCD diffractometer. ORTEP representation with 50% probability thermal ellipsoid. Crystal data have been deposited to CCDC with number 2330129.

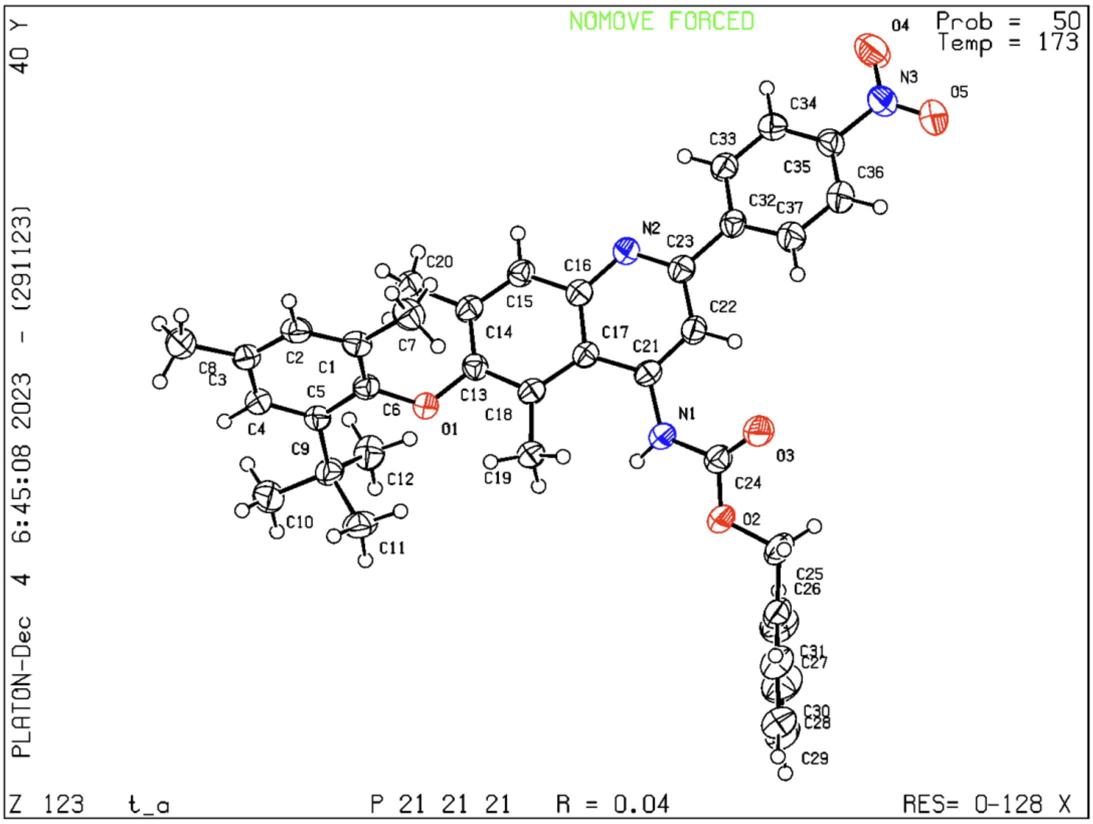

X-Ray structure of **4b** (with CCDC number 2330129)

Crystal data and structure refinement for 4b

Identification code	
Empirical formula	$C_{37}H_{37}N_3O_5$
Formula weight	603.69
Temperature/K	173(2)
Crystal system	Orthorhombic
Space group	$P2_12_12_1$
a/Å	7.2456(2)
b/Å	13.0727(3)
c/Å	33.4427(9)
α/°	90
β/°	90
γ/°	90
Volume/Å^3	3167.68(14)
Z	4
ρ_{calc}g/cm^3	1.266
μ/mm^{-1}	0.681
F(000)	1280.0
Crystal size/mm^3	0.18 × 0.16 × 0.14
Radiation	CuKα (λ = 1.54178)
2Θ range for data collection/°	5.284 to 136.808
Index ranges	$-8 \leq h \leq 8$, $-15 \leq k \leq 15$, $-40 \leq l \leq 40$
Reflections collected	50851
Independent reflections	5816 [R_{int} = 0.0761, R_{sigma} = 0.0355]
Data/restraints/parameters	5816/0/416
Goodness-of-fit on F^2	1.045
Final R indexes [I>=2σ (I)]	R_1 = 0.0428, wR_2 = 0.1088
Final R indexes [all data]	R_1 = 0.0521, wR_2 = 0.1158
Largest diff. peak/hole / e Å^{-3}	0.33/-0.21
Flack parameter	0.03(10)

The compound **4y** (5 mg) was dissolved in a mixed solvent of 10:1 n-hexane:DCM solvent (0.5mL) at room temperature, which was filtered through the filter membrane into a vial. The single crystal of **4y** was obtained by slowly evaporating the solvent at room temperature.

Crystal measurement: X-ray crystal structures of (R_{ether}, $R_{c\text{-}c}$, $R_{c\text{-}c}$)-**4y** were determined at 173 K by using Bruker APEX-II CCD diffractometer. ORTEP representation with 50% probability thermal ellipsoid. Crystal data have been deposited to CCDC with number 2330130.

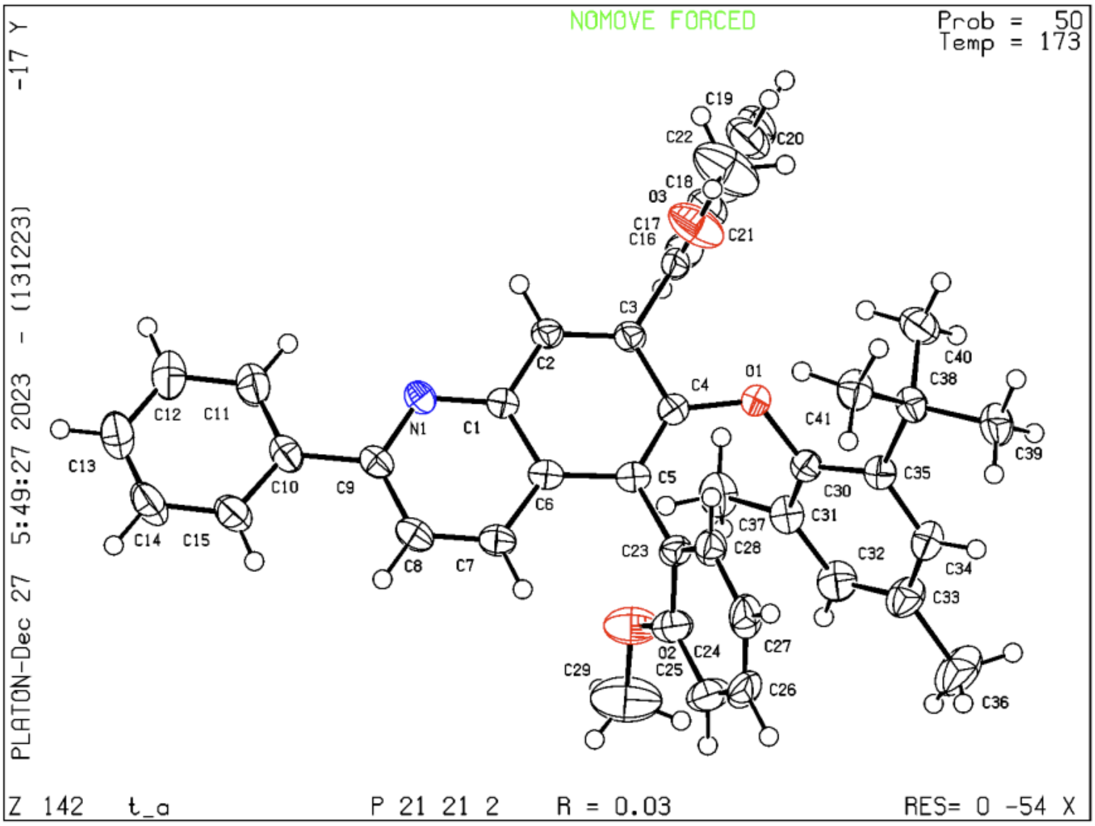

X-Ray structure of **4y** (with CCDC number 2330130)

Crystal data and structure refinement for 4y

Identification code	
Empirical formula	$C_{41}H_{39}NO_3$
Formula weight	593.73
Temperature/K	173(2)
Crystal system	Orthorhombic
Space group	$P2_12_12$
a/Å	14.5010(3)
b/Å	24.7204(5)
c/Å	9.2258(2)
α/°	90
β/°	90
γ/°	90
Volume/Å^3	3307.18(12)
Z	4
ρ_{calc}g/cm^3	1.192
μ/mm^{-1}	0.580
F(000)	1264.0
Crystal size/mm^3	0.2 × 0.2 × 0.2
Radiation	CuKα (λ = 1.54178)
2Θ range for data collection/°	7.068 to 136.676
Index ranges	$-17 \le h \le 17$, $-29 \le k \le 29$, $-11 \le l \le 11$
Reflections collected	43239
Independent reflections	5816 [R_{int} = 0.0607, R_{sigma} = 0.0342]
Data/restraints/parameters	6051/0/414
Goodness-of-fit on F^2	1.001
Final R indexes [I>=2σ (I)]	R_1 = 0.0331, wR_2 = 0.0914
Final R indexes [all data]	R_1 = 0.0387, wR_2 = 0.0946
Largest diff. peak/hole / e Å^{-3}	0.18/-0.15
Flack parameter	-0.06(7)

The compound **4o** (5 mg) was dissolved in a mixed solvent of 10:1 n-hexane:DCM solvent (0.5mL) at room temperature, which was filtered through the filter membrane into a vial. The single crystal of **4o** was obtained by slowly evaporating the solvent at room temperature.

Crystal measurement: X-ray crystal structures of **4o** were determined at 173 K by using Bruker APEX-II CCD diffractometer. ORTEP representation with 50% probability thermal ellipsoid. Crystal data have been deposited to CCDC with number 2353648.

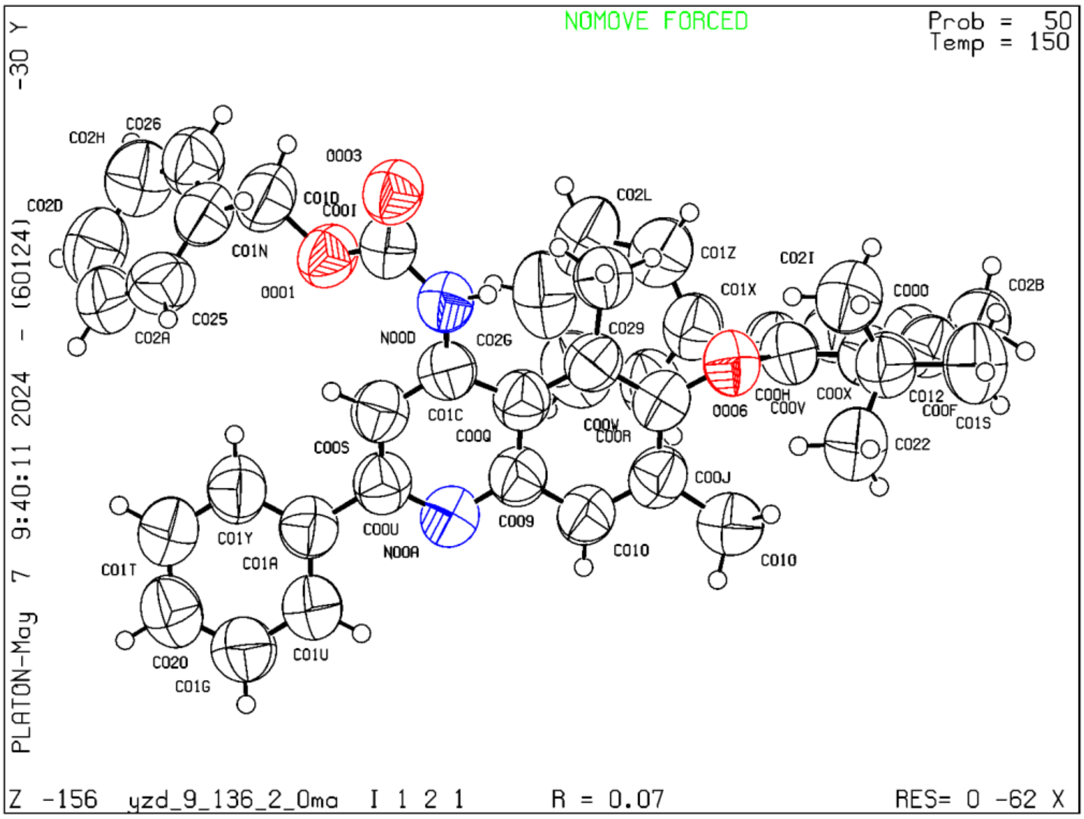

X-Ray structure of **4o** (with CCDC number 2353648)

Crystal data and structure refinement for 4o

Identification code	YZD_9_136_2_0ma
Empirical formula	$C_{42}H_{40}N_2O_3$
Formula weight	620.76
Temperature/K	150.15
Crystal system	monoclinic
Space group	I2
a/Å	15.5623(19)
b/Å	9.0385(6)
c/Å	24.0940(17)
α/°	90
β/°	90.917(8)
γ/°	90
Volume/$Å^3$	3388.6(5)
Z	4
$\rho_{calc}g/cm^3$	1.217
μ/mm^{-1}	0.380
F(000)	1320.0
Crystal size/mm^3	0.1 × 0.1 × 0.1
Radiation	GaKα (λ = 1.34138)
2Θ range for data collection/°	5.84 to 108.004
Index ranges	-18 ≤ h ≤ 18, -10 ≤ k ≤ 10, -29 ≤ l ≤ 29
Reflections collected	50806
Independent reflections	6192 [R_{int} = 0.1267, R_{sigma} = 0.1039]
Data/restraints/parameters	6192/8/372
Goodness-of-fit on F^2	1.013
Final R indexes [I>=2σ (I)]	R_1 = 0.0696, wR_2 = 0.1810
Final R indexes [all data]	R_1 = 0.1454, wR_2 = 0.2298
Largest diff. peak/hole / e $Å^{-3}$	0.12/-0.12
Flack parameter	0.4(10)

HPLC traces

Benzyl ((2S,4S)-6-(2-(tert-butyl)-4,6-dimethylphenoxy)-5,7-dimethyl-2-phenyl-1,2,3,4-tetrahydroquinolin-4-yl)carbamate (**4a’**)

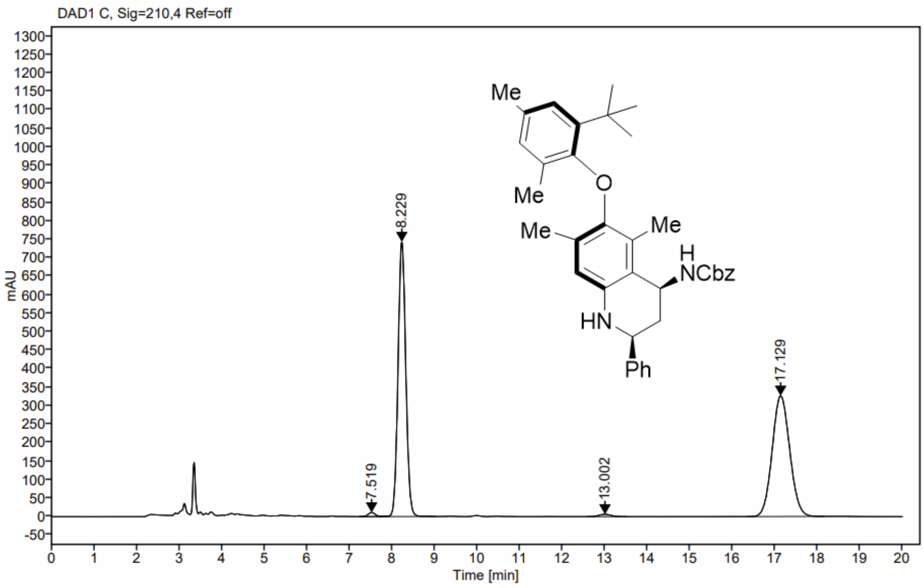

RT [min]	Width [min]	Area	Height	Area%
7.519	0.1882	137.9804	11.1636	0.7252
8.229	0.1974	9386.4795	742.8295	49.3350
13.002	0.3143	125.2591	6.1856	0.6584
17.129	0.4469	9376.2773	326.6440	49.2814

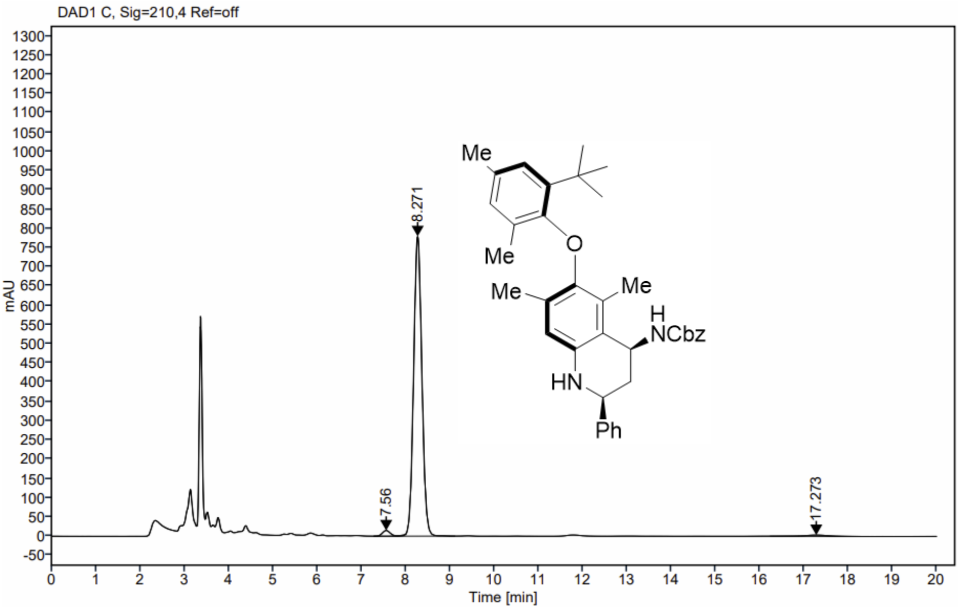

RT [min]	Width [min]	Area	Height	Area%
7.560	0.2036	198.2591	14.8666	1.9002
8.271	0.2033	10106.9980	779.1707	96.8682
17.273	0.5232	128.5069	3.2647	1.2316

Benzyl (6-(2-(tert-butyl)-4,6-dimethylphenoxy)-5,7-dimethyl-2-phenylquinolin-4-yl) carbamate (**4a**)

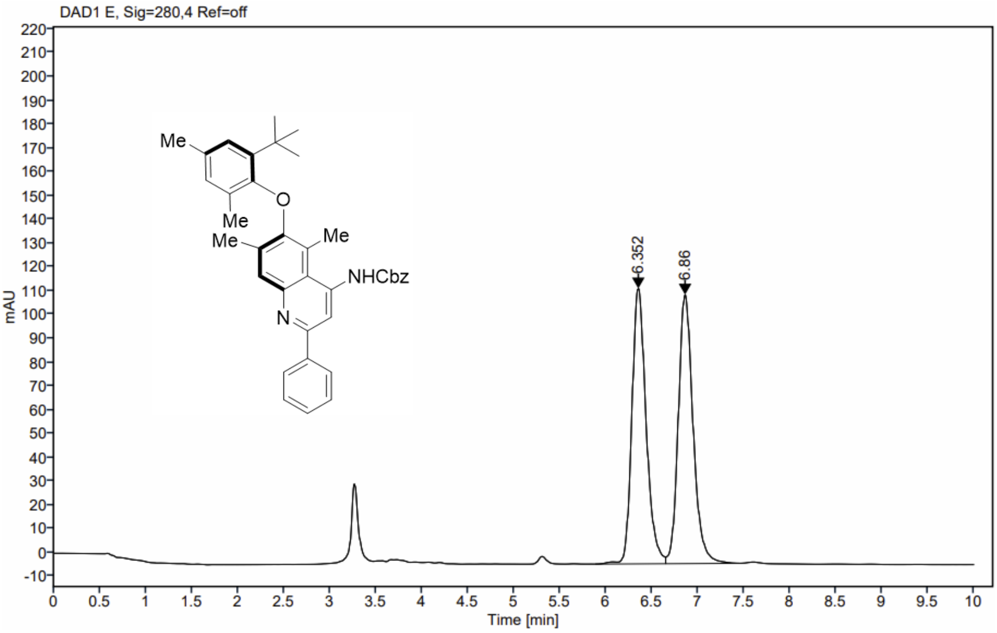

RT [min]	Width [min]	Area	Height	Area%
6.352	0.1634	1243.6606	115.7256	49.6091
6.860	0.1704	1263.2618	112.9995	50.3909

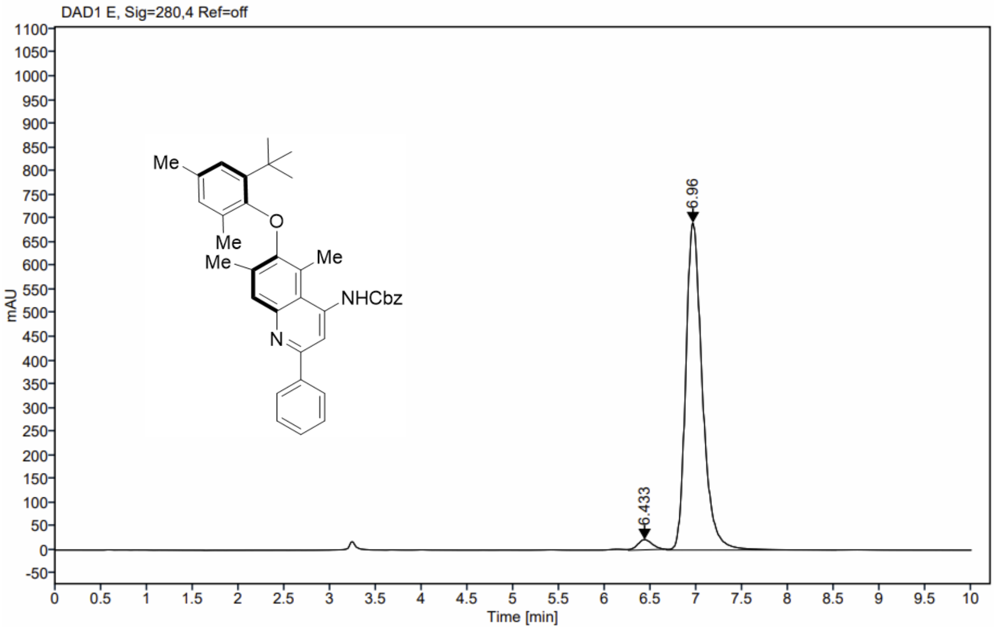

RT [min]	Width [min]	Area	Height	Area%
6.433	0.1841	238.6465	21.6038	2.6783
6.960	0.2091	8671.7236	691.0334	97.3217

Benzyl (6-(2-(tert-butyl)-4,6-dimethylphenoxy)-5,7-dimethyl-2-(4-nitrophenyl)quinolin-4-yl)carbamate (**4b**)

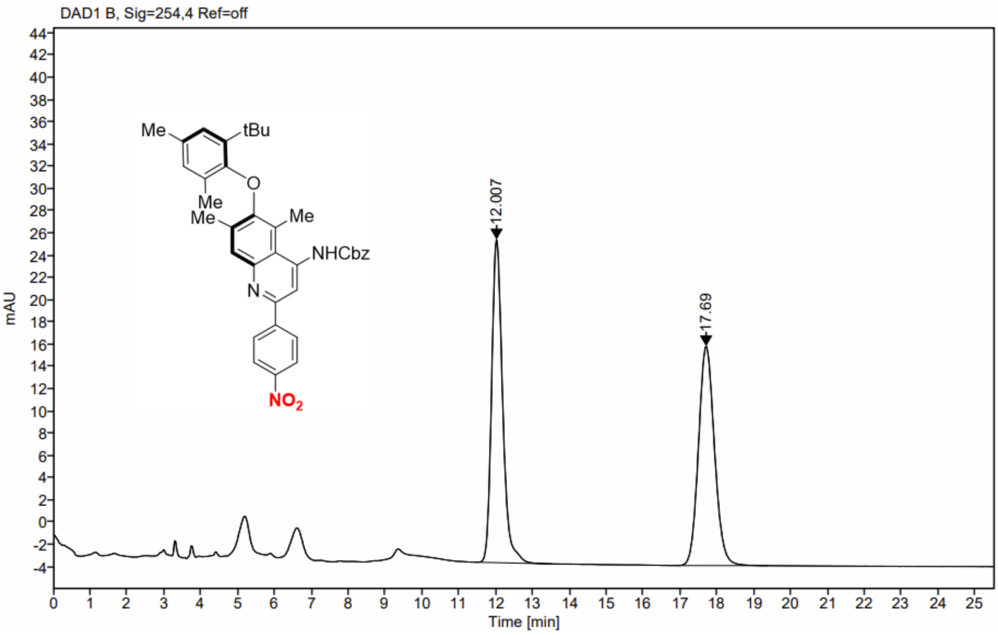

RT [min]	Width [min]	Area	Height	Area%
12.007	0.3159	602.4175	29.0506	50.3306
17.690	0.4639	594.5038	19.7089	49.6694

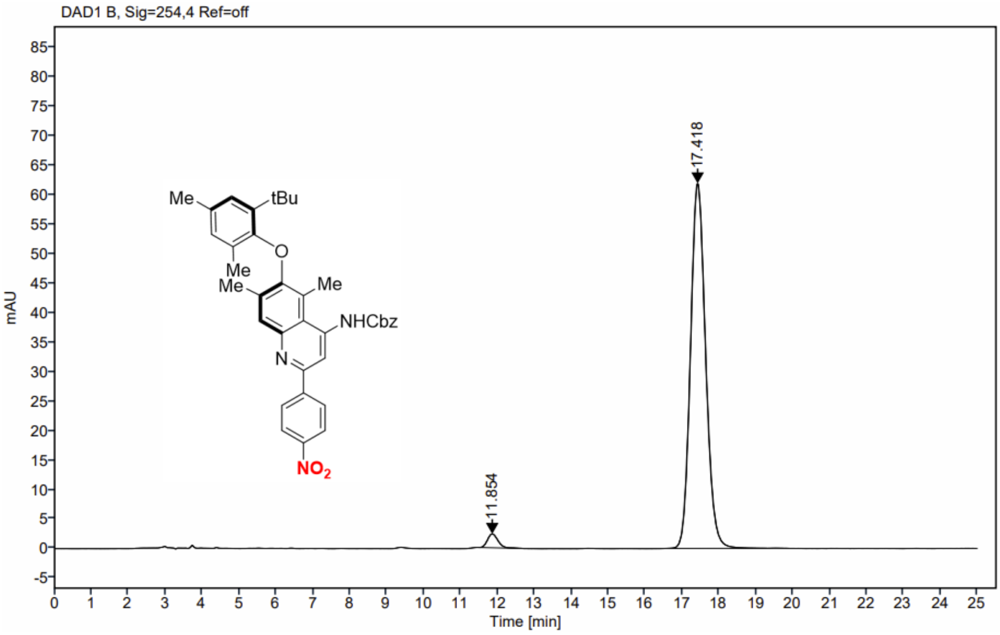

RT [min]	Width [min]	Area	Height	Area%
11.854	0.2988	46.5491	2.3531	2.5706
17.418	0.4384	1764.2889	61.9165	97.4294

Benzyl (6-(2-(tert-butyl)-4,6-dimethylphenoxy)-2-(4-cyanophenyl)-5,7-dimethylquinolin-4-yl)carbamate (**4c**)

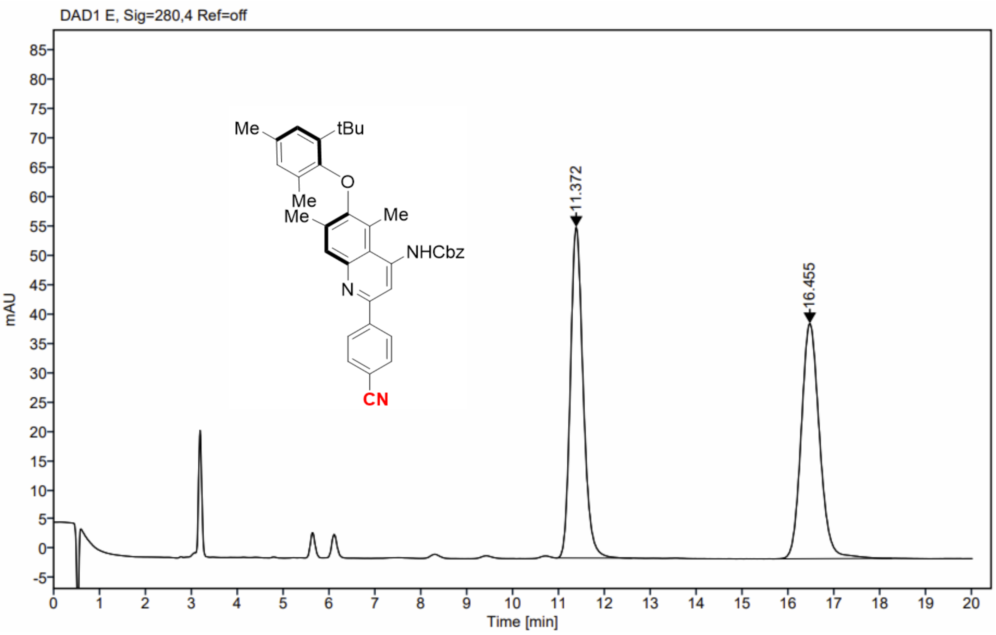

RT [min]	Width [min]	Area	Height	Area%
11.372	0.2974	1090.0466	56.4180	49.6048
16.455	0.4241	1107.4150	40.1081	50.3952

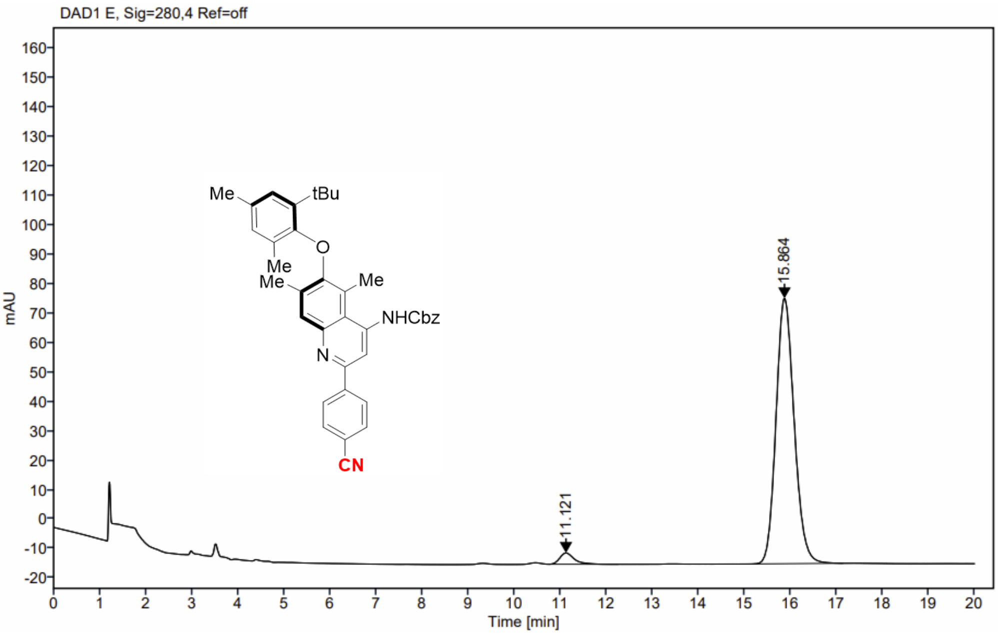

RT [min]	Width [min]	Area	Height	Area%
11.121	0.3242	83.3773	3.8272	3.2633
15.864	0.4239	2471.6135	90.1357	96.7367

Benzyl (6-(2-(tert-butyl)-4,6-dimethylphenoxy)-2-(4-methoxyphenyl)-5,7-dimethylquinolin-4-yl)carbamate (**4d**)

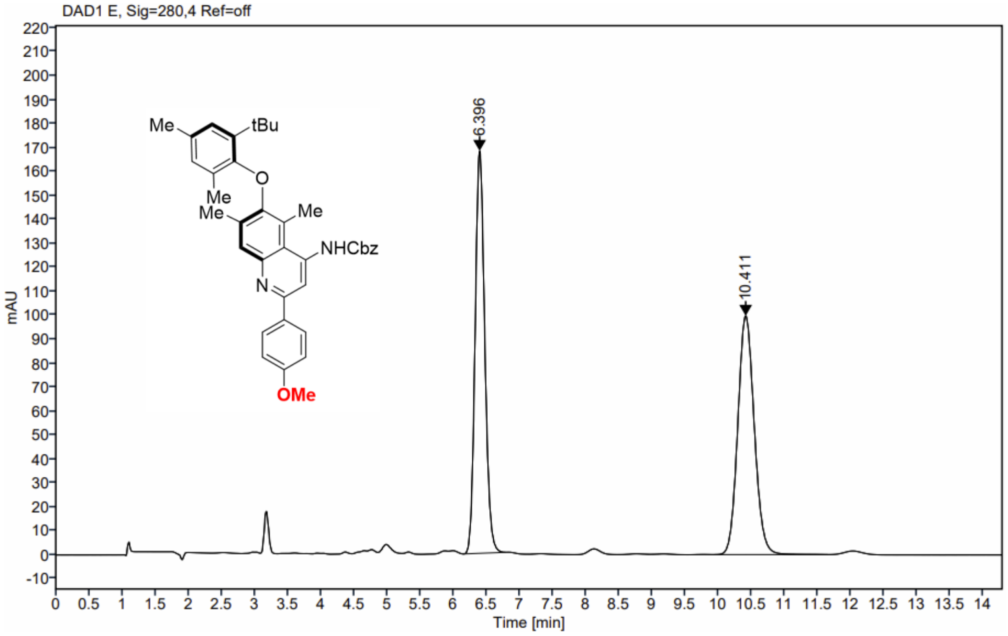

RT [min]	Width [min]	Area	Height	Area%
6.396	0.1561	1700.7318	168.0076	49.6628
10.411	0.2678	1723.8292	99.6766	50.3372

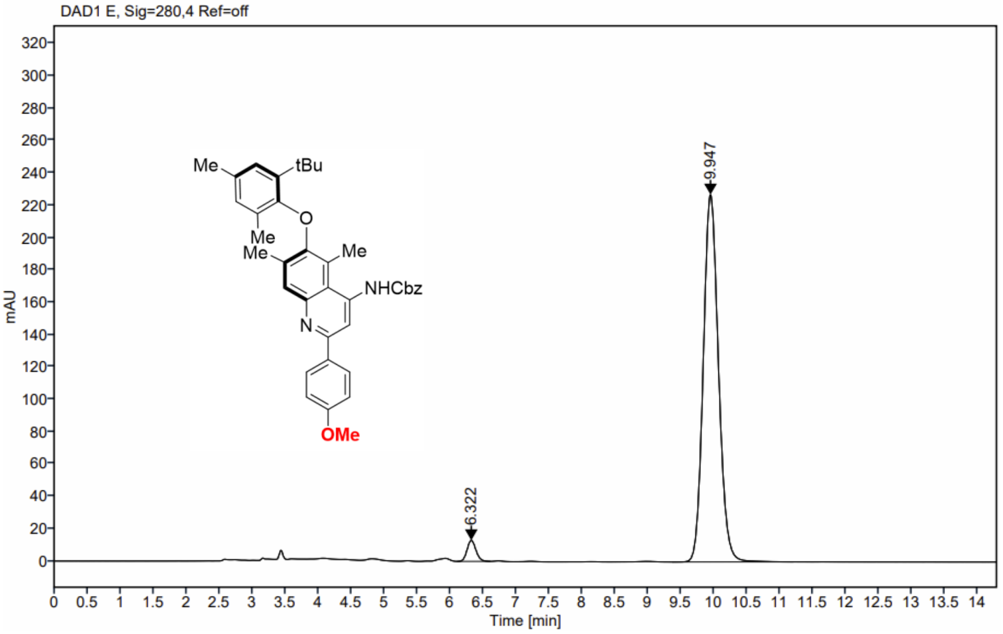

RT [min]	Width [min]	Area	Height	Area%
6.322	0.1452	121.9404	13.0292	3.2411
9.947	0.2469	3640.3662	227.2005	96.7589

Benzyl (6-(2-(tert-butyl)-4,6-dimethylphenoxy)-5,7-dimethyl-2-(3-(trifluoromethyl)phenyl)quinolin-4-yl)carbamate (**4e**)

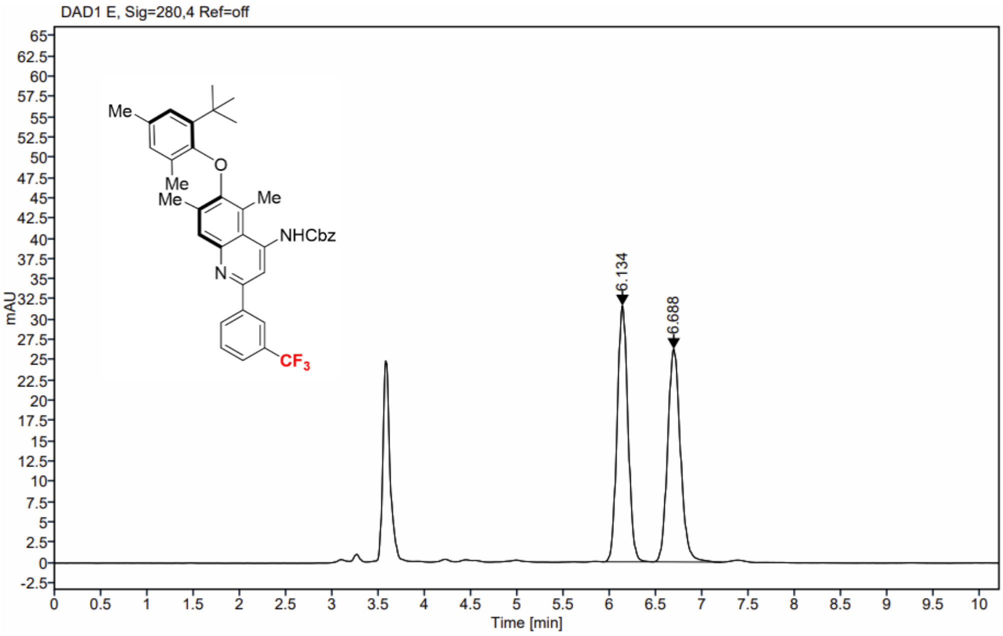

RT [min]	Width [min]	Area	Height	Area%
6.134	0.1243	255.5963	31.6184	50.2500
6.688	0.1484	253.0531	26.2752	49.7500

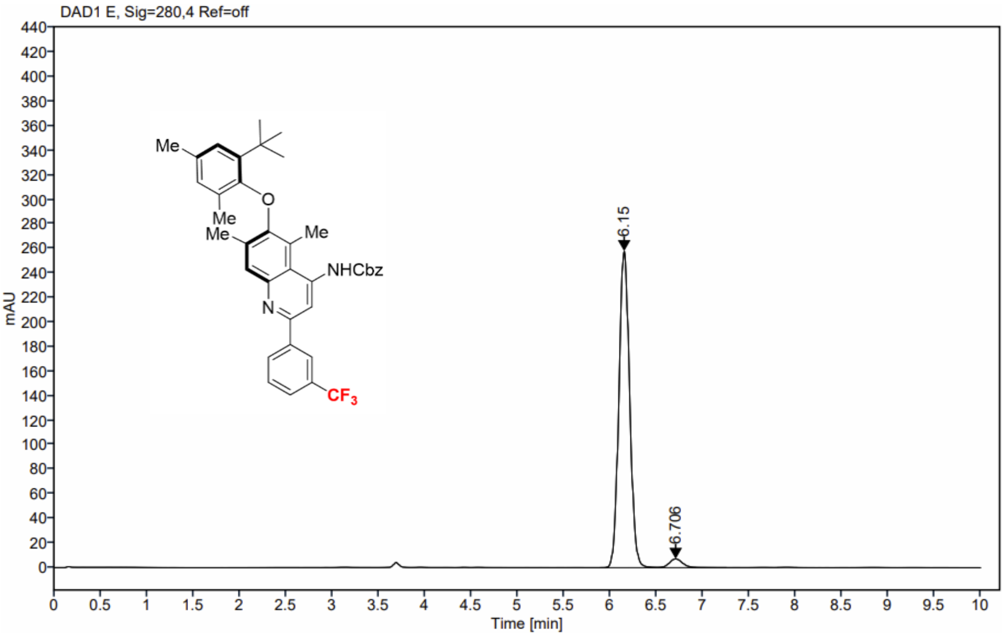

RT [min]	Width [min]	Area	Height	Area%
6.150	0.1242	2084.5168	258.0380	96.6933
6.706	0.1522	71.2849	7.1570	3.3067

Benzyl (6-(2-(tert-butyl)-4,6-dimethylphenoxy)-5,7-dimethyl-2-(o-tolyl)quinolin-4-yl) carbamate (**4f**)

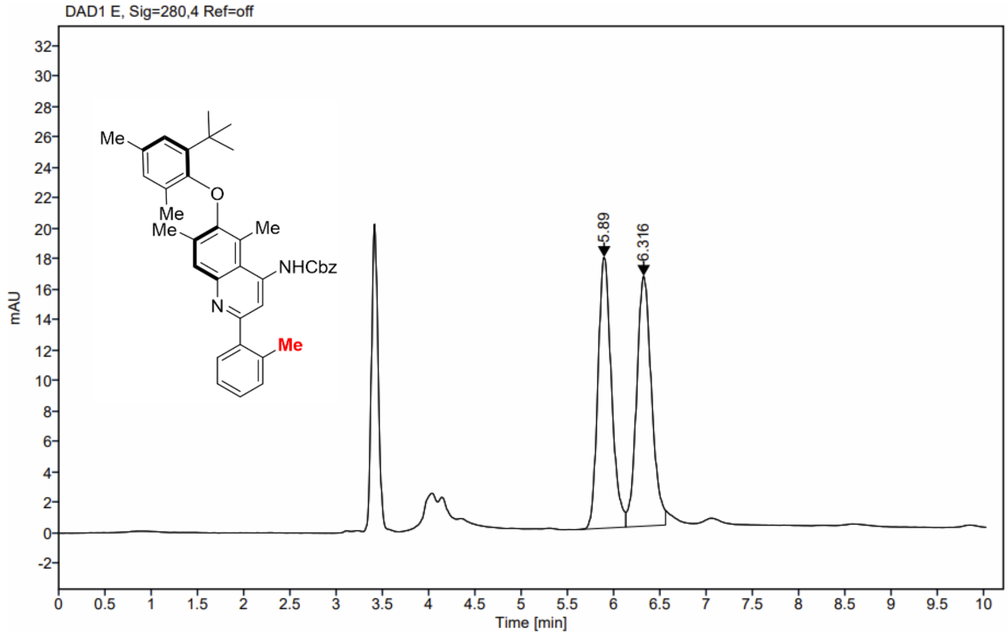

RT [min]	Width [min]	Area	Height	Area%
5.890	0.1545	180.7227	17.7941	49.5741
6.316	0.1866	183.8279	16.4228	50.4259

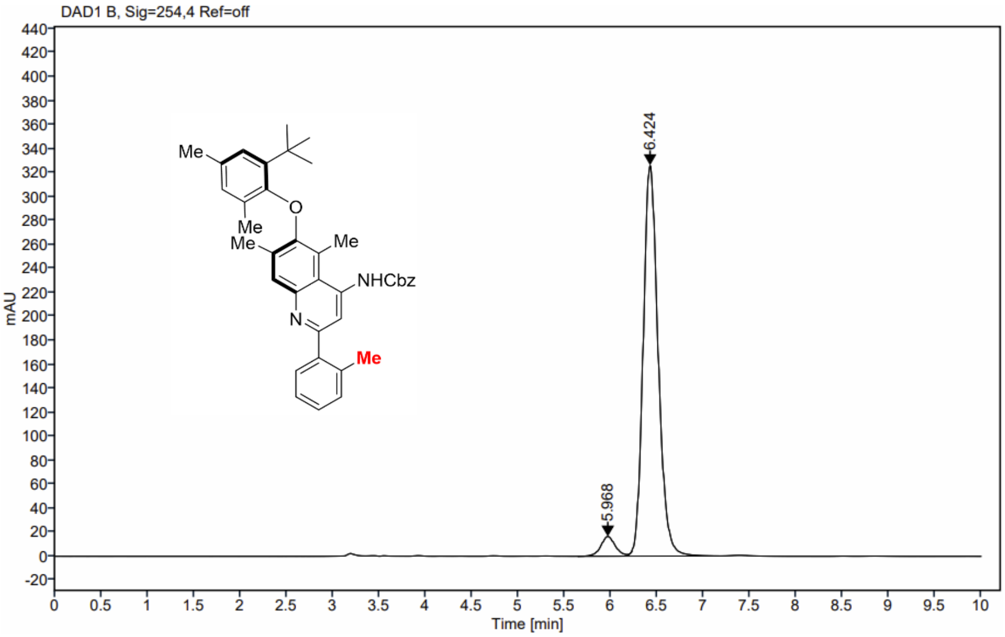

RT [min]	Width [min]	Area	Height	Area%
5.968	0.1633	178.7342	16.3857	4.6591
6.424	0.1729	3657.4666	326.0944	95.3409

Benzyl (2-(2-bromophenyl)-6-(2-(tert-butyl)-4,6-dimethylphenoxy)-5,7-dimethylquinolin-4-yl)carbamate (**4g**)

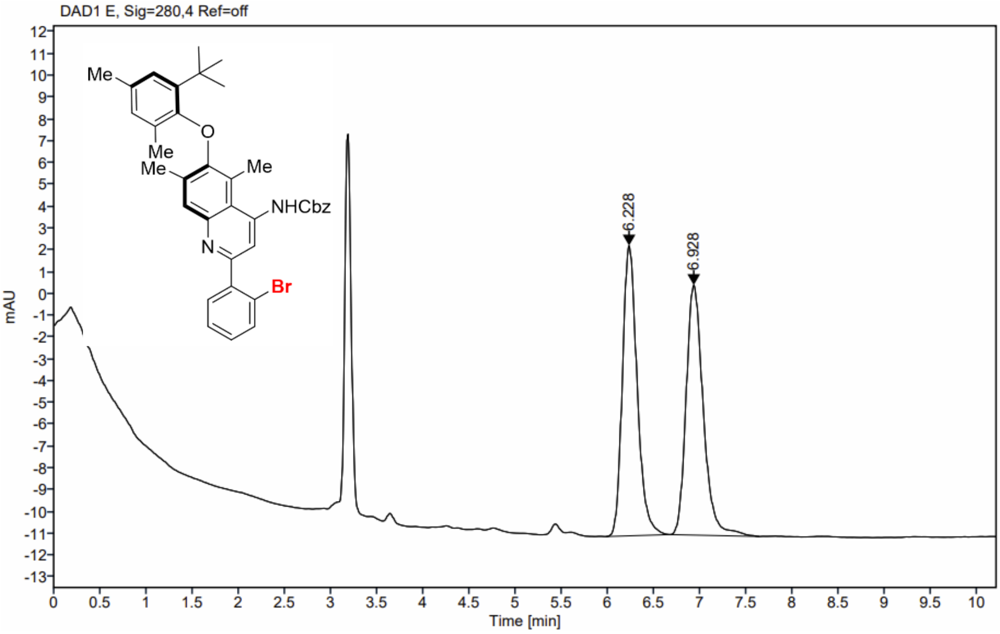

RT [min]	Width [min]	Area	Height	Area%
6.228	0.1695	147.7439	13.3086	49.9328
6.928	0.1988	148.1417	11.4629	50.0672

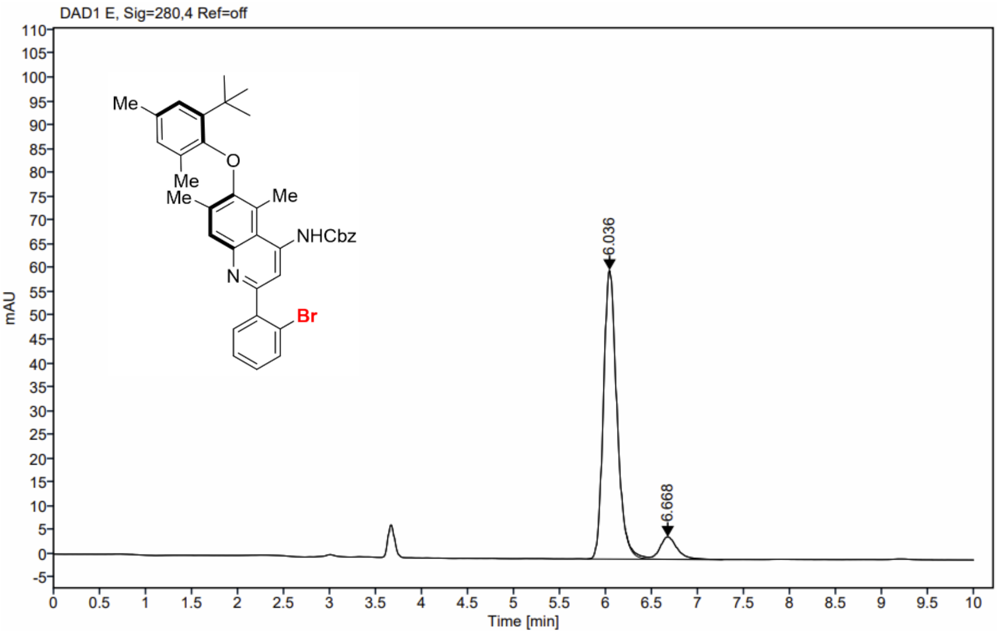

RT [min]	Width [min]	Area	Height	Area%
6.036	0.1567	617.2537	60.6605	90.3228
6.668	0.2079	66.1326	4.7088	9.6772

Benzyl (6-(2-(tert-butyl)-4,6-dimethylphenoxy)-2-(3,4-dichlorophenyl)-5,7-dimethyl quinolin-4-yl)carbamate (**4h**)

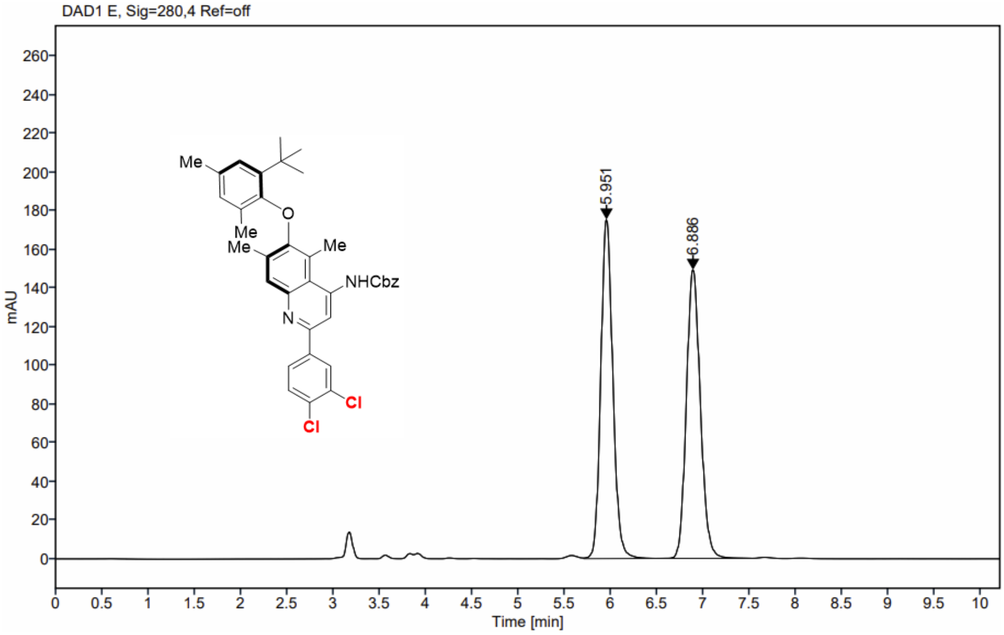

RT [min]	Width [min]	Area	Height	Area%
5.951	0.1508	1588.6599	175.5397	50.2937
6.886	0.1606	1570.1035	149.3665	49.7063

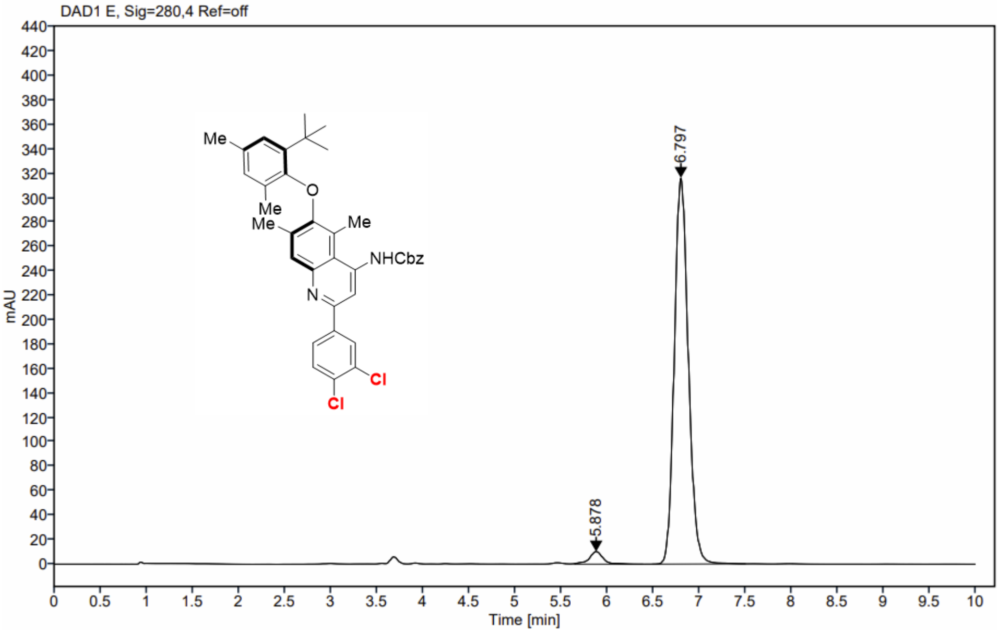

RT [min]	Width [min]	Area	Height	Area%
5.878	0.1749	108.2991	10.3212	3.1416
6.797	0.1631	3338.9382	316.3876	96.8584

Benzyl (6-(2-(tert-butyl)-4,6-dimethylphenoxy)-5,7-dimethyl-2-(naphthalen-2-yl)quinolin-4-yl)carbamate (**4i**)

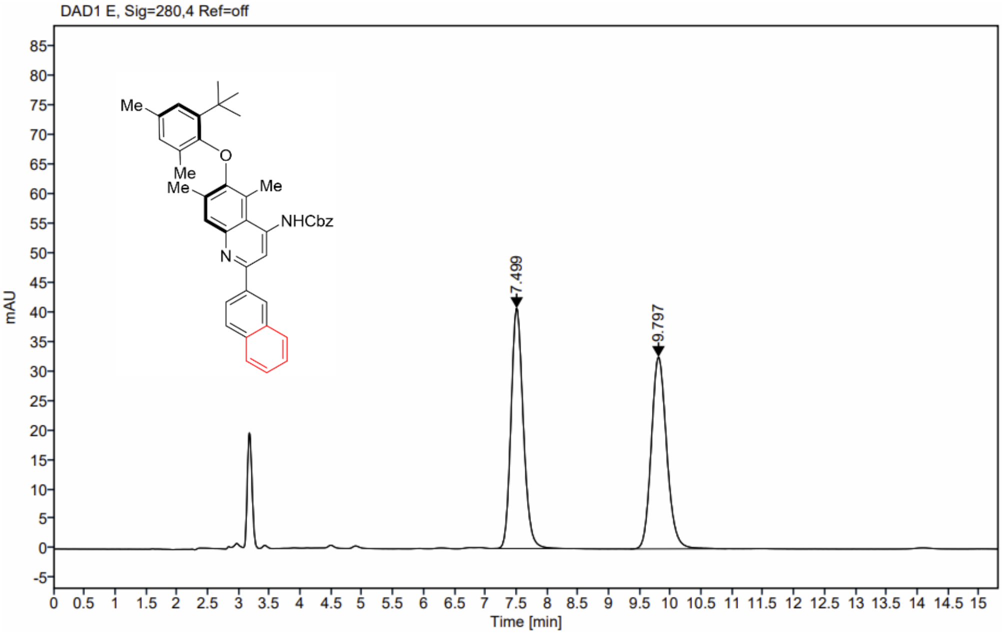

RT [min]	Width [min]	Area	Height	Area%
7.499	0.2110	561.3271	40.6711	49.8664
9.797	0.2668	564.3353	32.4636	50.1336

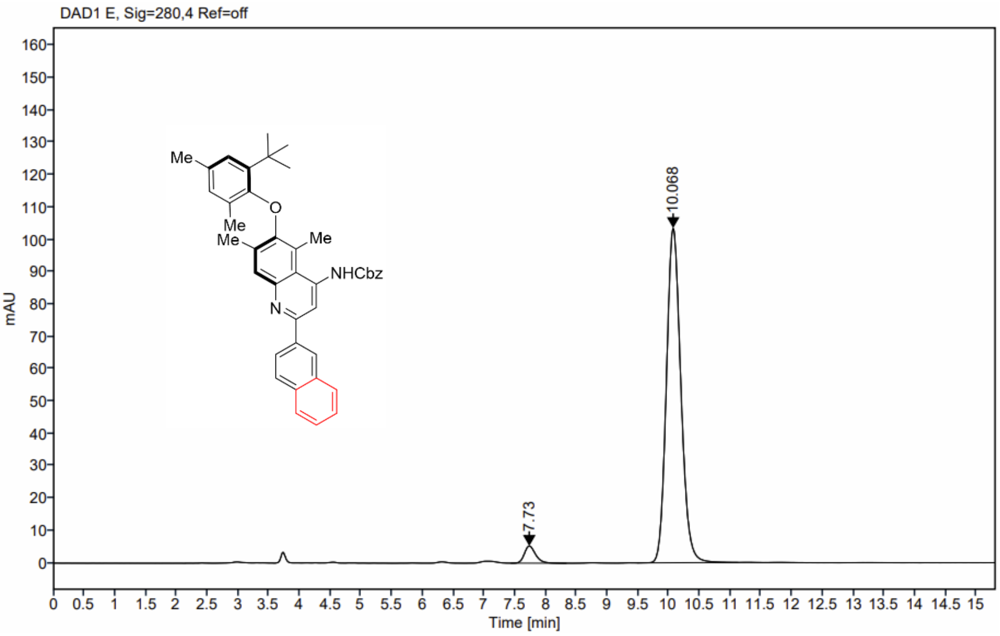

RT [min]	Width [min]	Area	Height	Area%
7.730	0.1934	67.1292	5.3143	3.8328
10.068	0.2519	1684.3134	103.4887	96.1672

Benzyl (6-(2-(tert-butyl)-4,6-dimethylphenoxy)-5,7-dimethyl-2-(naphthalen-1-yl)quinolin-4-yl)carbamate (**4j**)

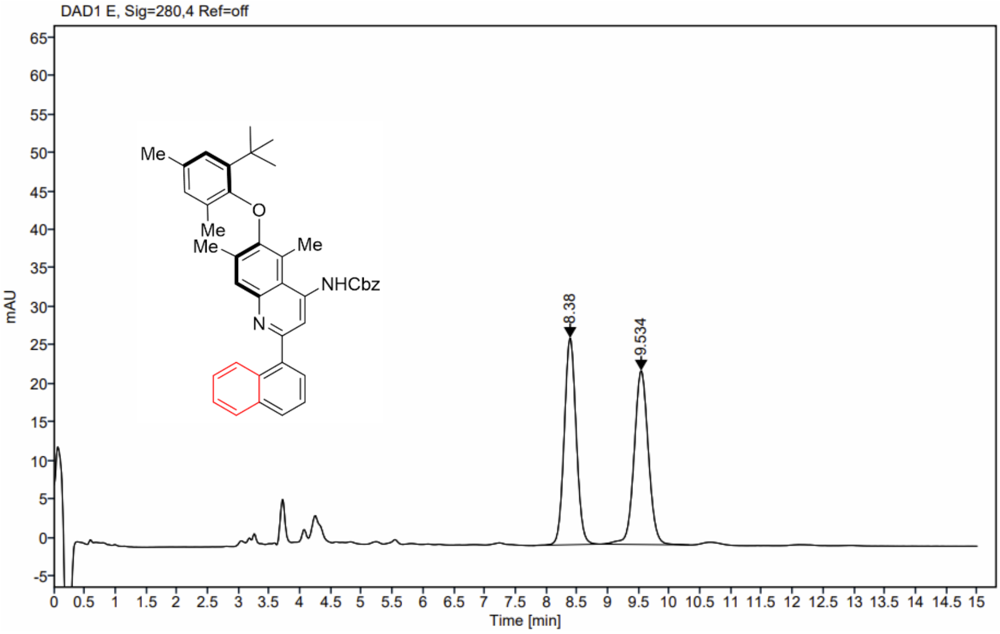

RT [min]	Width [min]	Area	Height	Area%
8.380	0.2089	360.7036	26.8161	49.6971
9.534	0.2489	365.1001	22.5437	50.3029

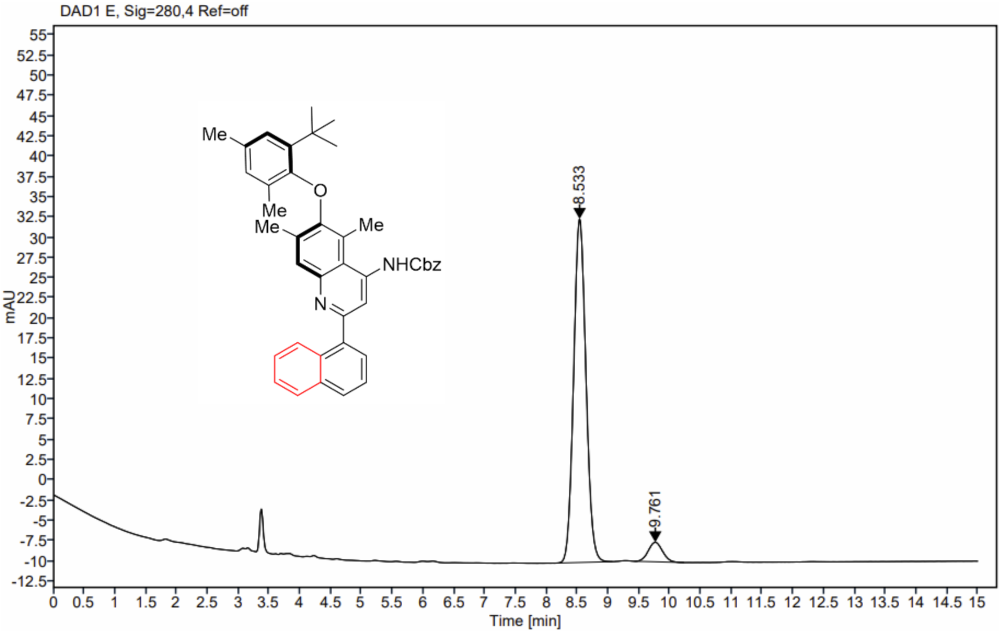

RT [min]	Width [min]	Area	Height	Area%
8.533	0.2158	588.7911	42.4673	93.8019
9.761	0.2441	38.9055	2.4134	6.1981

Benzyl (6-(2-(tert-butyl)-4,6-dimethylphenoxy)-5,7-dimethyl-2-propylquinolin-4-yl)carbamate (**4k**)

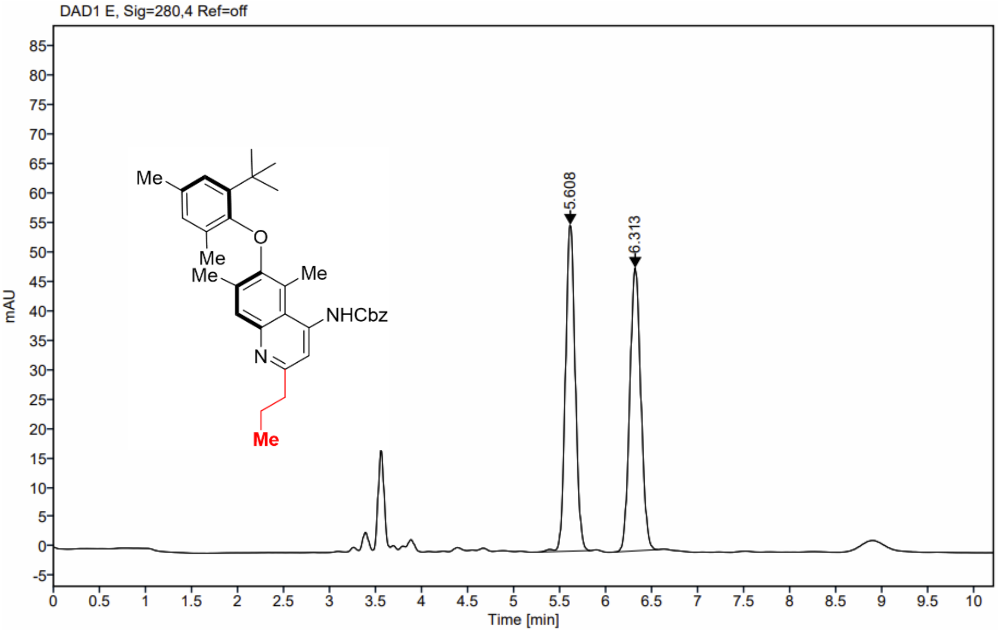

RT [min]	Width [min]	Area	Height	Area%
5.608	0.1153	405.1057	55.4190	50.1592
6.313	0.1297	402.5343	48.0510	49.8408

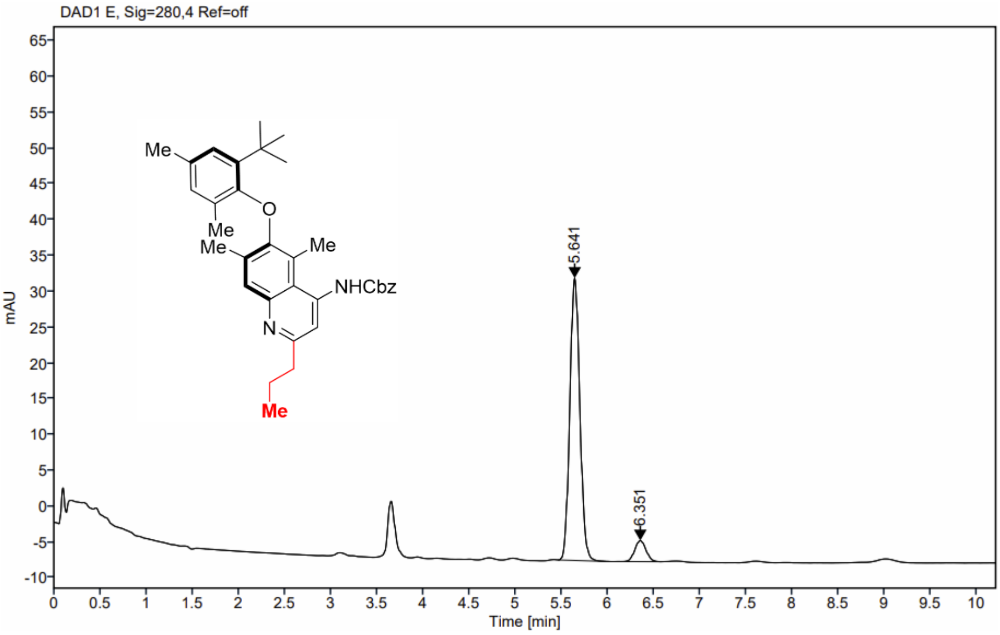

RT [min]	Width [min]	Area	Height	Area%
5.641	0.1178	296.7046	39.4404	92.2602
6.351	0.1310	24.8909	2.9321	7.7398

Benzyl (6-(2-(tert-butyl)-4,6-dimethylphenoxy)-2-isopropyl-5,7-dimethylquinolin-4-yl)carbamate (**4l**)

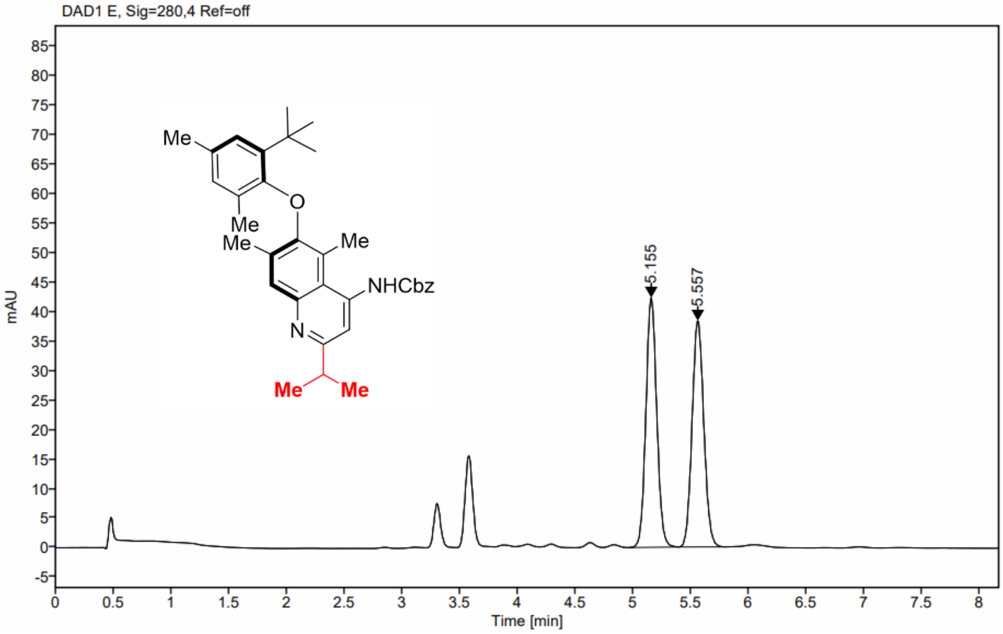

RT [min]	Width [min]	Area	Height	Area%
5.155	0.1022	276.1641	42.3099	50.5246
5.557	0.1103	270.4288	38.3404	49.4754

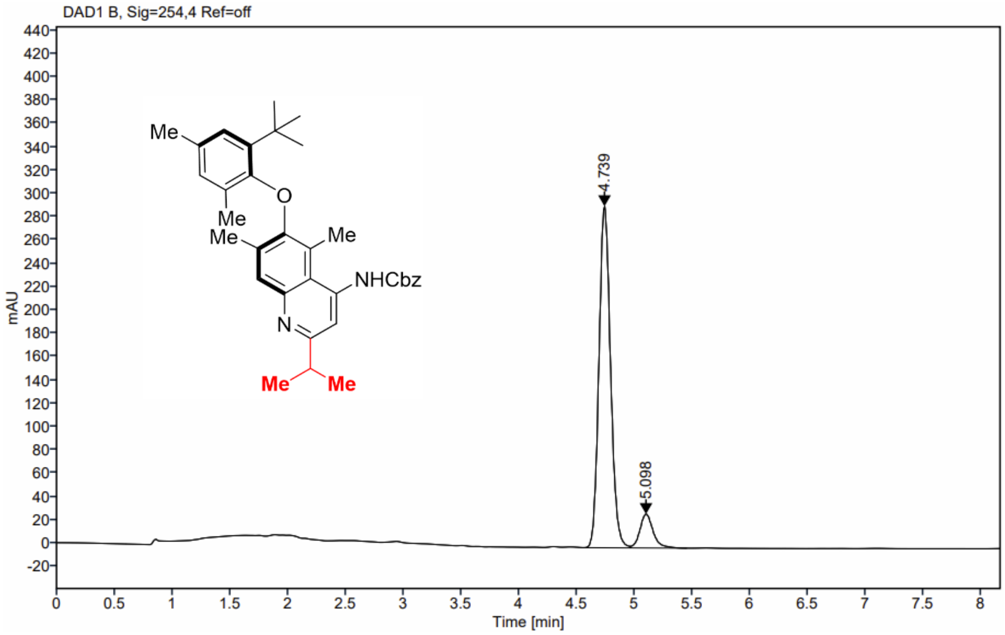

RT [min]	Width [min]	Area	Height	Area%
4.739	0.1072	2037.4976	292.7923	89.8192
5.098	0.1208	230.9447	29.0387	10.1808

Benzyl (6-(2-(tert-butyl)-4,6-dimethylphenoxy)-2-cyclohexyl-5,7-dimethylquinolin-4-yl)carbamate (**4m**)

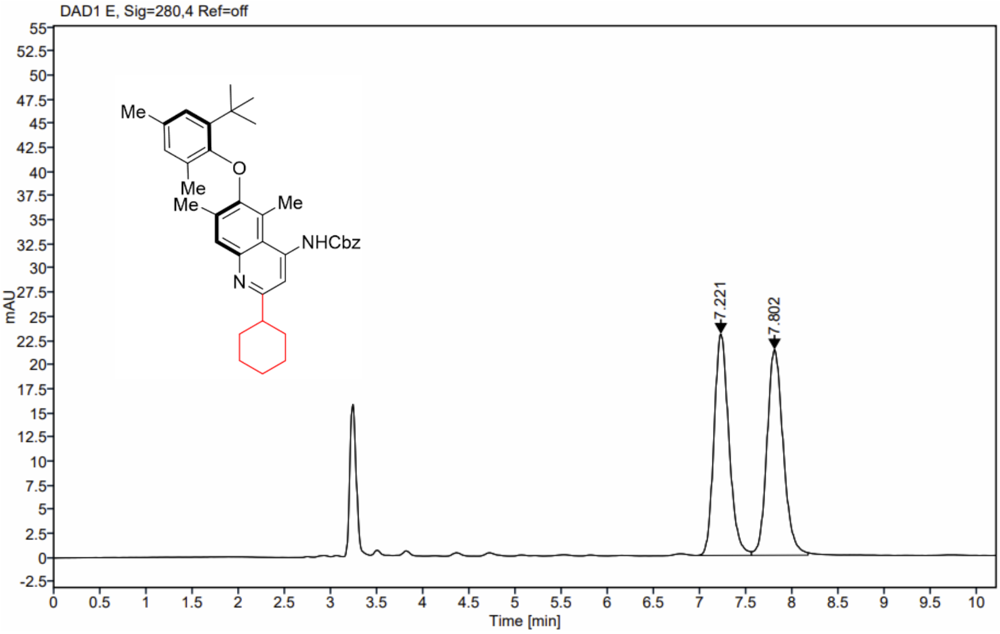

RT [min]	Width [min]	Area	Height	Area%
7.221	0.1706	257.1213	22.9635	49.6186
7.802	0.2041	261.0739	21.3184	50.3814

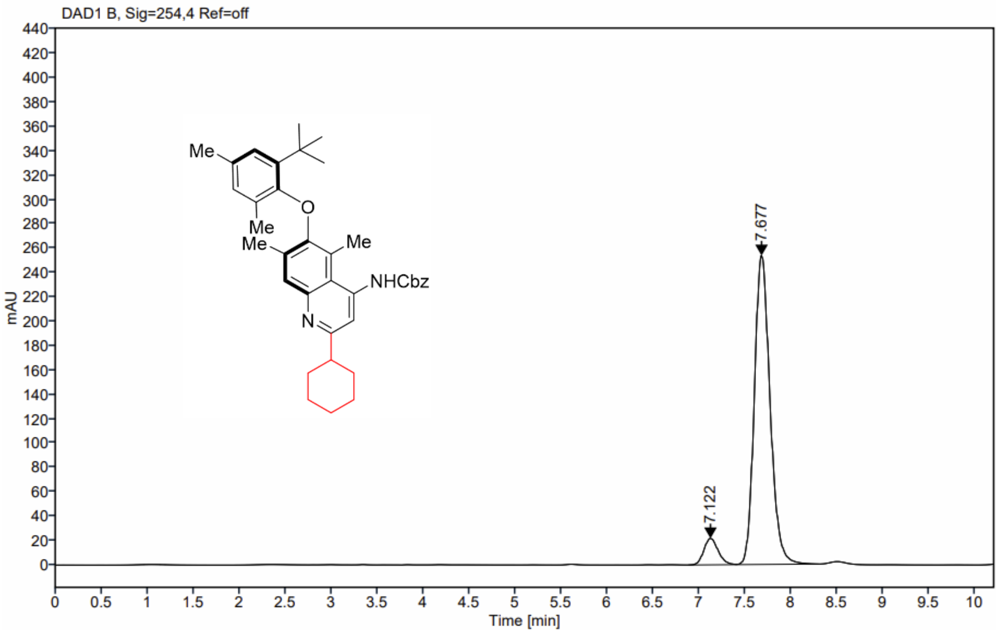

RT [min]	Width [min]	Area	Height	Area%
7.122	0.1683	234.9944	21.7081	7.2392
7.677	0.1825	3011.1387	253.6090	92.7608

Benzyl (6-((3-(tert-butyl)-5-methyl-[1,1'-biphenyl]-2-yl)oxy)-5,7-dimethyl-2-phenylquinolin-4-yl)carbamate (**4o**)

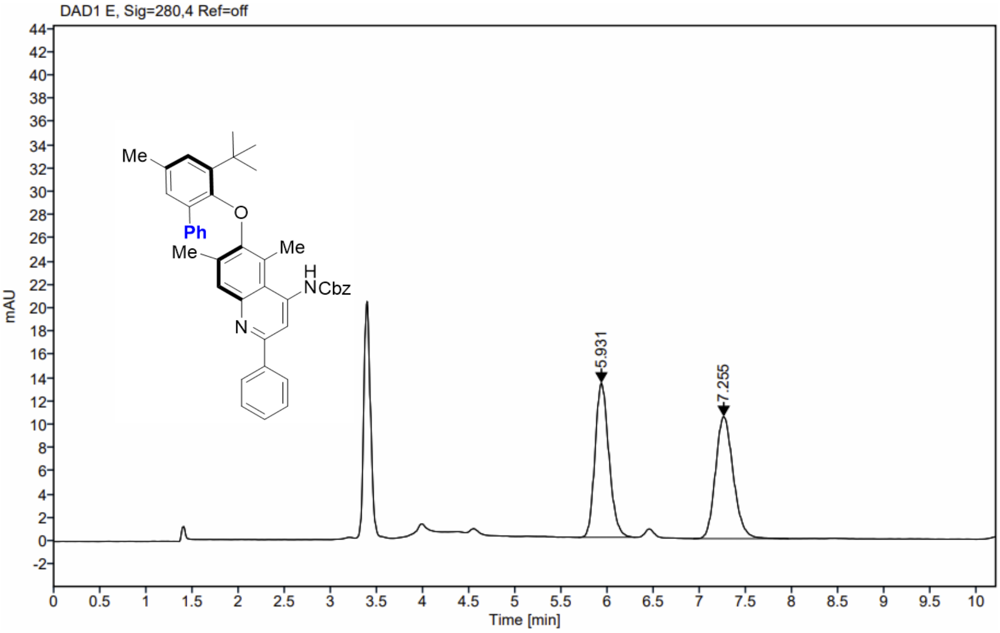

RT [min]	Width [min]	Area	Height	Area%
5.931	0.1621	138.5769	13.2359	49.6984
7.255	0.2063	140.2590	10.4715	50.3016

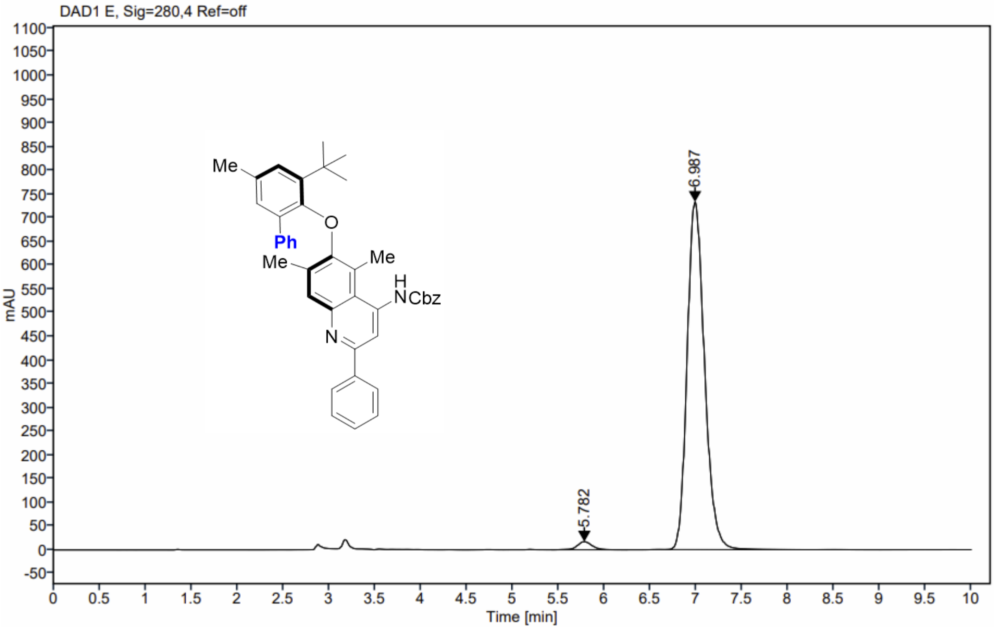

RT [min]	Width [min]	Area	Height	Area%
5.782	0.1702	191.1414	17.1312	1.9386
6.987	0.2039	9668.6729	733.0953	98.0614

Benzyl (6-((3-(tert-butyl)-5-methyl-3'-(trifluoromethyl)-[1,1'-biphenyl]-2-yl)oxy)-5,7-dimethyl-2-phenylquinolin-4-yl)carbamate (**4p**)

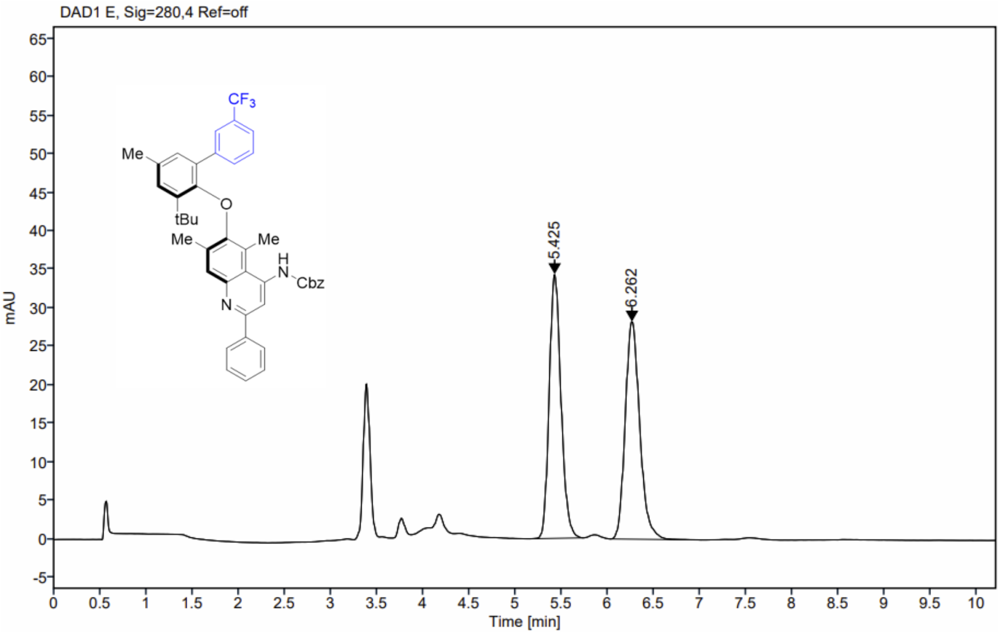

RT [min]	Width [min]	Area	Height	Area%
5.425	0.1357	304.5566	34.2626	49.6650
6.262	0.1696	308.6650	28.2162	50.3350

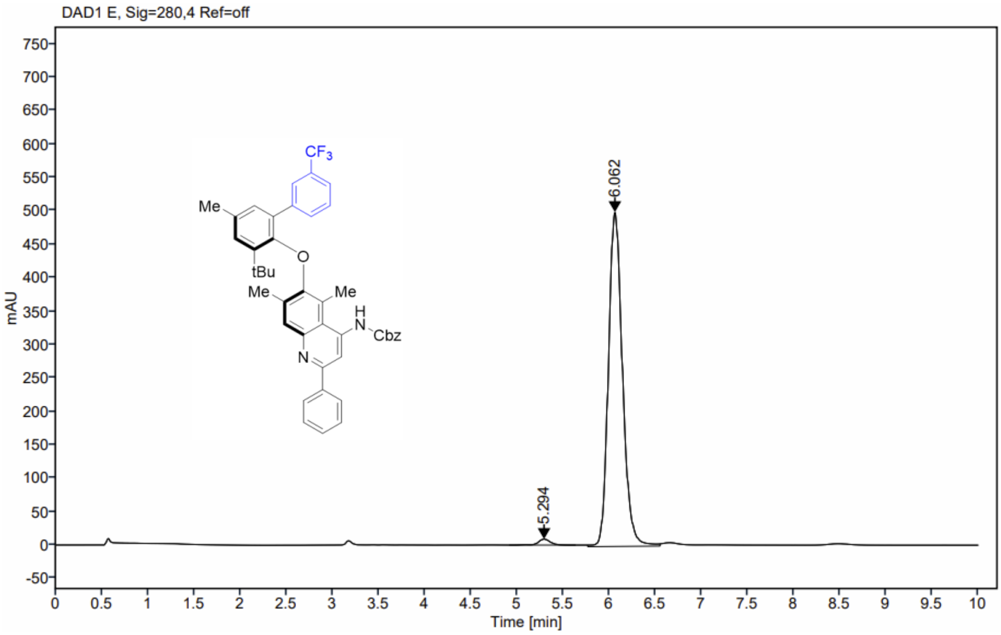

RT [min]	Width [min]	Area	Height	Area%
5.294	0.1384	81.1942	8.8990	1.5096
6.062	0.1765	5297.3911	500.2465	98.4904

Benzyl (6-(2-(tert-butyl)-4-methyl-6-(naphthalen-1-yl)phenoxy)-5,7-dimethyl-2-phenylquinolin-4-yl)carbamate (**4q**)

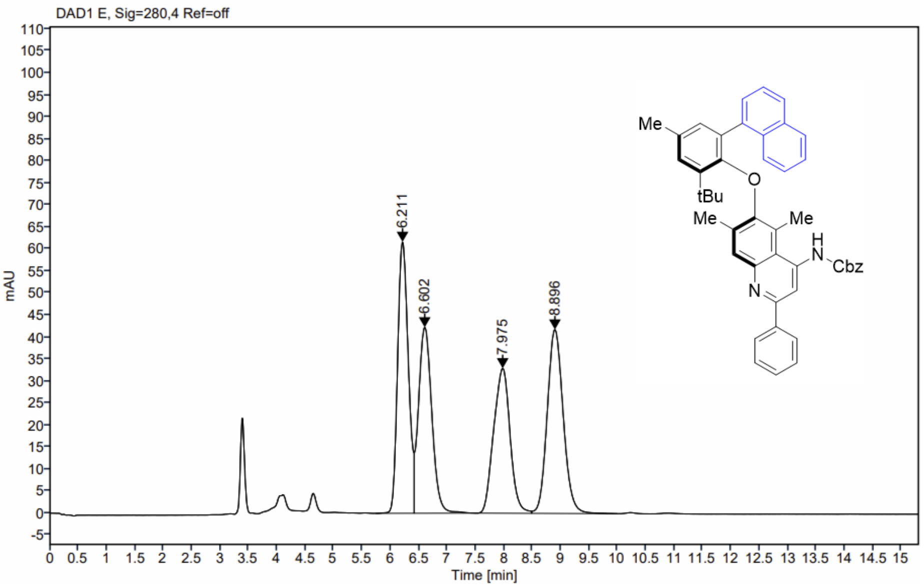

RT [min]	Width [min]	Area	Height	Area%
6.211	0.2042	804.7975	61.6779	26.9680
6.602	0.2564	696.5991	42.2449	23.3424
7.975	0.3285	662.0719	32.9377	22.1854
8.896	0.3052	820.7975	41.7878	27.5042

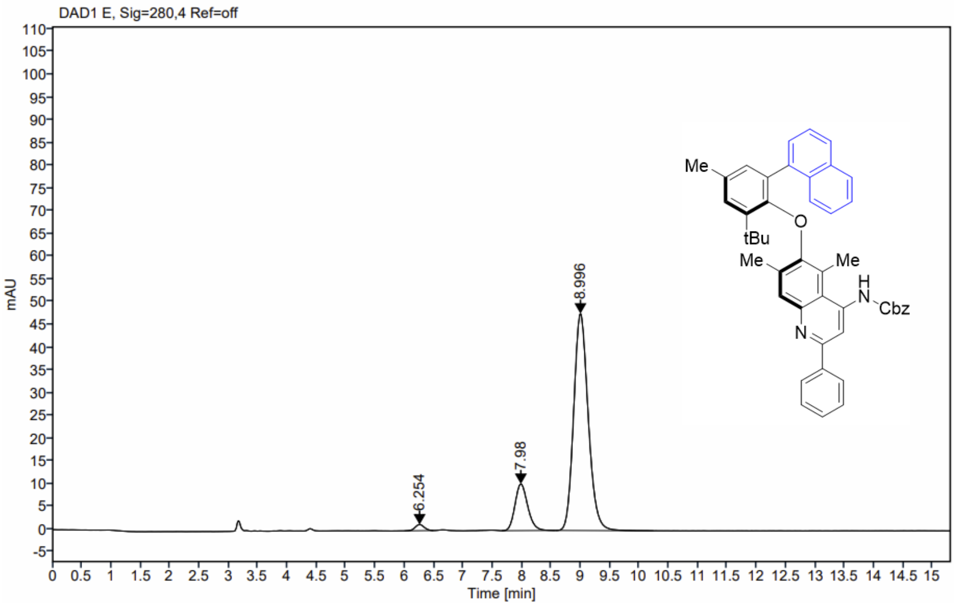

RT [min]	Width [min]	Area	Height	Area%
6.254	0.1660	14.5674	1.3486	1.4639
7.980	0.2314	154.1592	10.2559	15.4922
8.996	0.2681	826.3469	47.7095	83.0438

Benzyl (6-((3-(tert-butyl)-5-methyl-2',3',4',5'-tetrahydro-[1,1'-biphenyl]-2-yl)oxy)-5,7-dimethyl-2-phenylquinolin-4-yl)carbamate (**4r**)

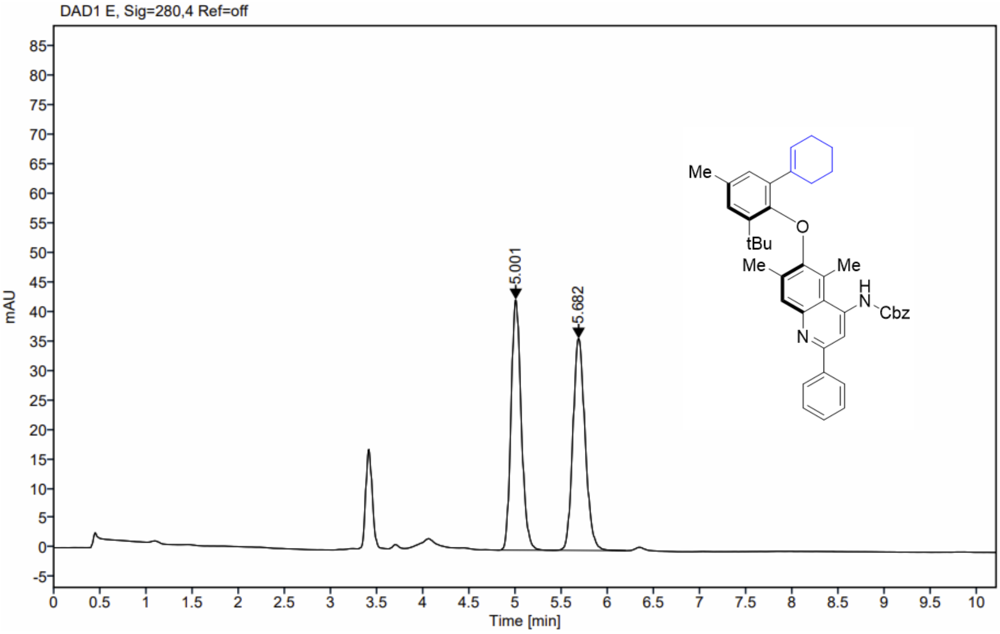

RT [min]	Width [min]	Area	Height	Area%
5.001	0.1176	326.1345	42.4779	49.6522
5.682	0.1415	330.7037	35.8909	50.3478

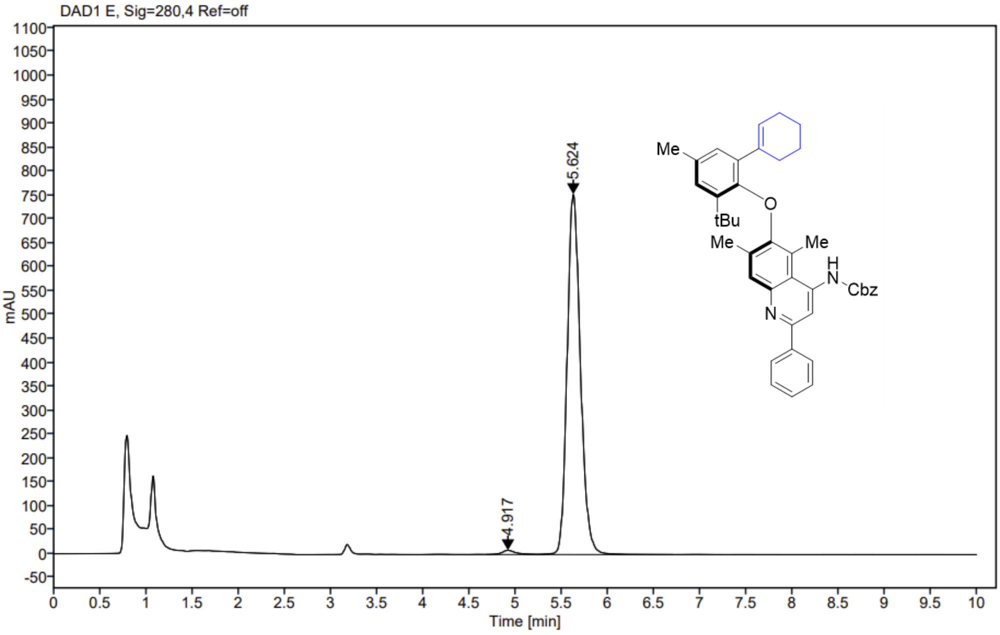

RT [min]	Width [min]	Area	Height	Area%
4.917	0.1523	91.7652	8.7552	1.2042
5.624	0.1525	7528.9609	753.7800	98.7958

Benzyl (6-(2-(tert-butyl)-6-methylphenoxy)-5,7-dimethyl-2-phenylquinolin-4-yl)carbamate (**4s**)

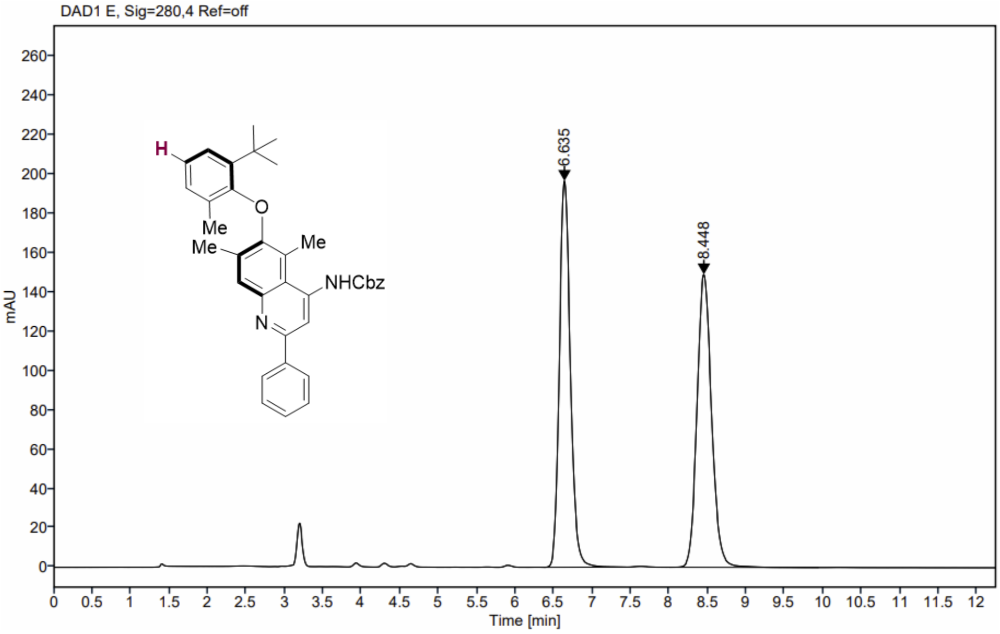

RT [min]	Width [min]	Area	Height	Area%
6.635	0.1538	1951.2516	196.5318	49.9216
8.448	0.2033	1957.3807	148.9954	50.0784

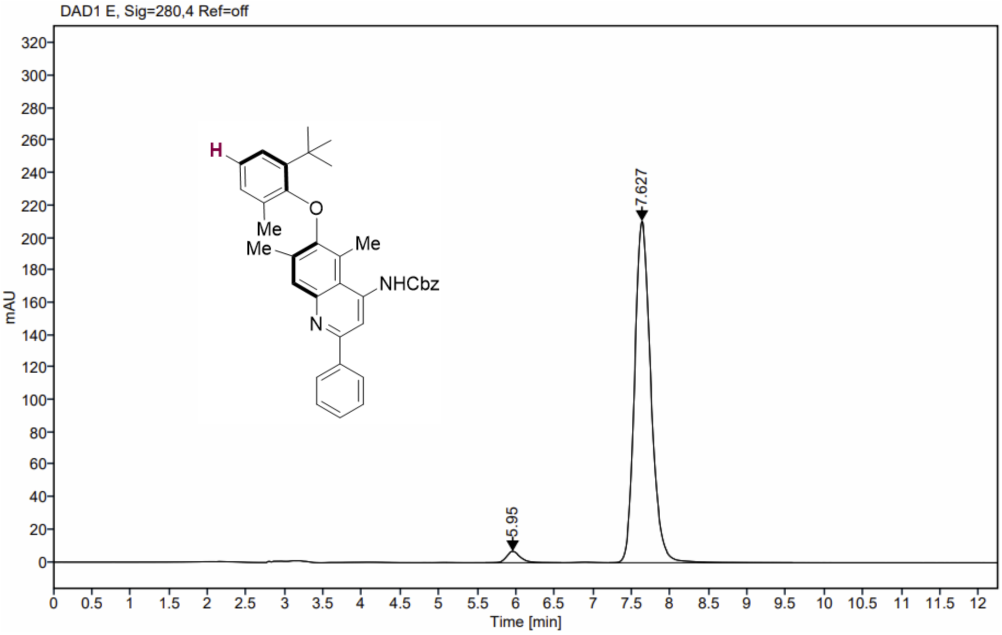

RT [min]	Width [min]	Area	Height	Area%
5.950	0.1878	87.4084	6.9959	2.7503
7.627	0.2251	3090.7439	210.7748	97.2497

Benzyl (6-(2-(tert-butyl)-6-methylphenoxy)-5,7-dimethyl-2-phenylquinolin-4-yl)carbamate (**4t**)

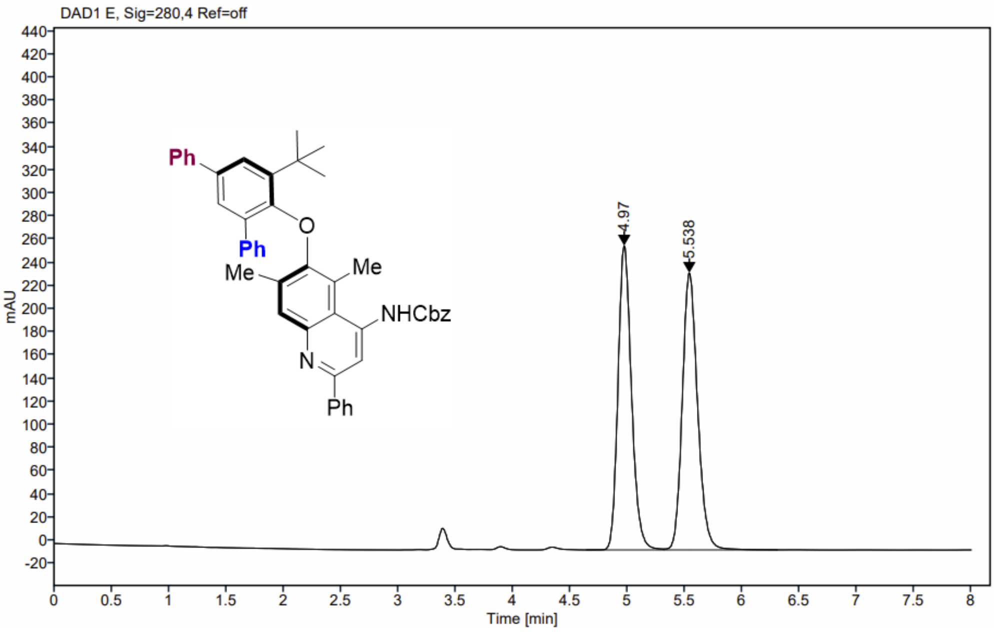

RT [min]	Width [min]	Area	Height	Area%
4.970	0.1253	2145.8152	262.7496	49.2273
5.538	0.1419	2213.1755	239.2325	50.7727

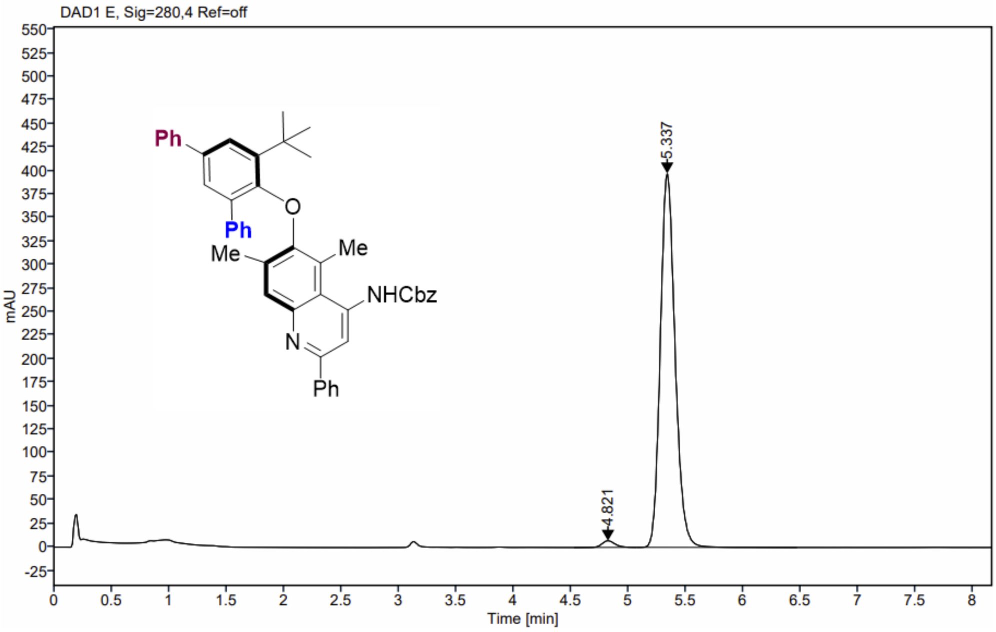

RT [min]	Width [min]	Area	Height	Area%
4.821	0.1189	55.0928	7.0700	1.5299
5.337	0.1361	3545.9521	397.4685	98.4701

Benzyl (6-((5'-(tert-butyl)-4,4''-dimethoxy-[1,1':3',1''-terphenyl]-4'-yl)oxy)-5,7-dimethyl-2-phenylquinolin-4-yl)carbamate (**4u**)

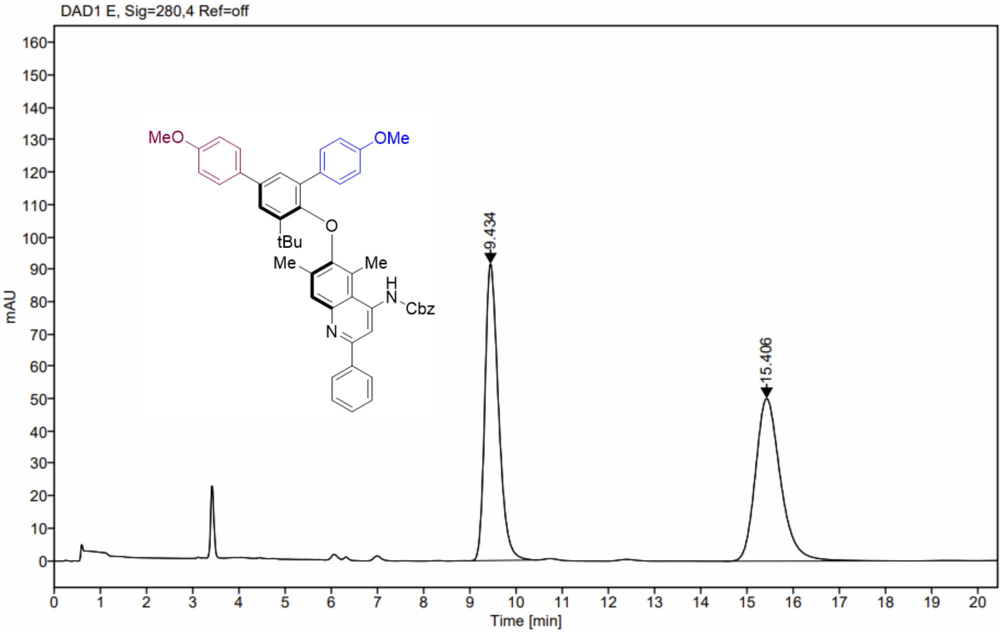

RT [min]	Width [min]	Area	Height	Area%
9.434	0.3117	1868.0295	91.6784	49.7495
15.406	0.5720	1886.8447	50.2538	50.2505

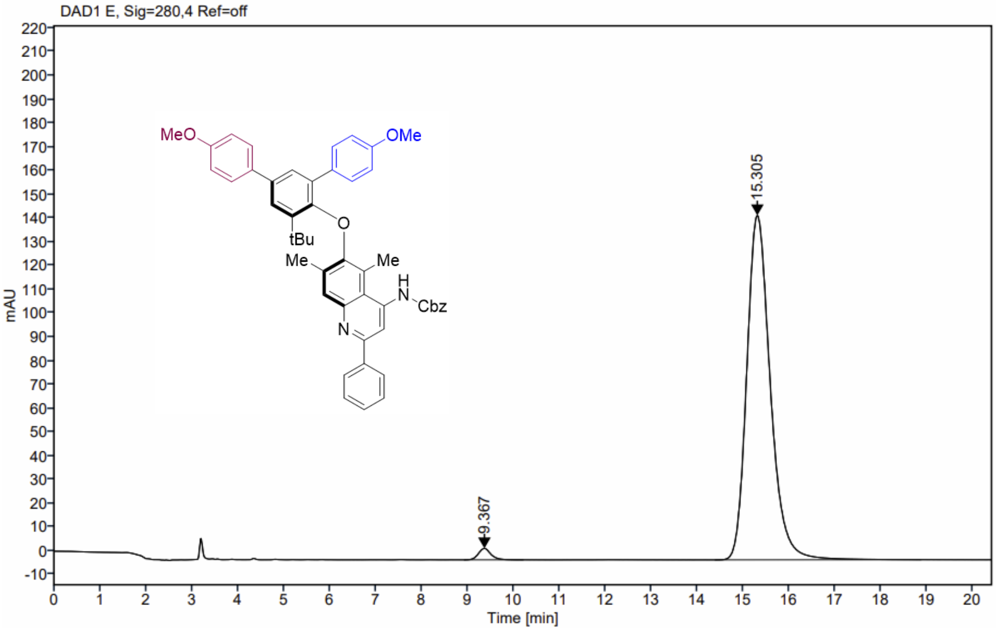

RT [min]	Width [min]	Area	Height	Area%
9.367	0.2862	90.7752	4.8090	1.7316
15.305	0.5446	5151.4297	144.9481	98.2684

Benzyl (6-(2-(tert-butyl)-4,6-dimethylphenoxy)-5,7-diethyl-2-phenylquinolin-4-yl)carbamate (**4v**)

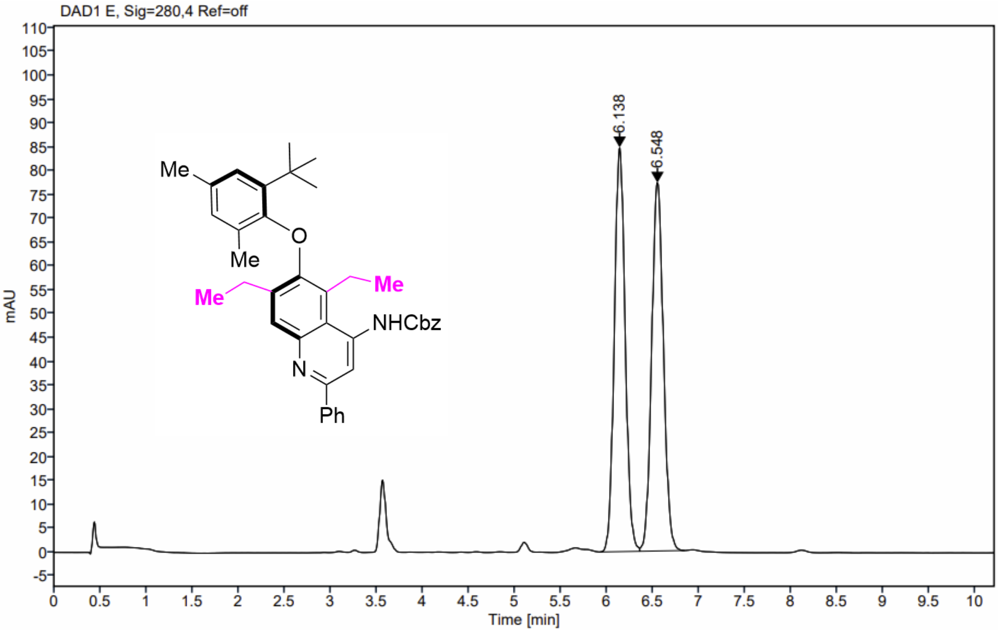

RT [min]	Width [min]	Area	Height	Area%
6.138	0.1276	695.0165	84.7933	50.3278
6.548	0.1376	685.9633	77.2589	49.6722

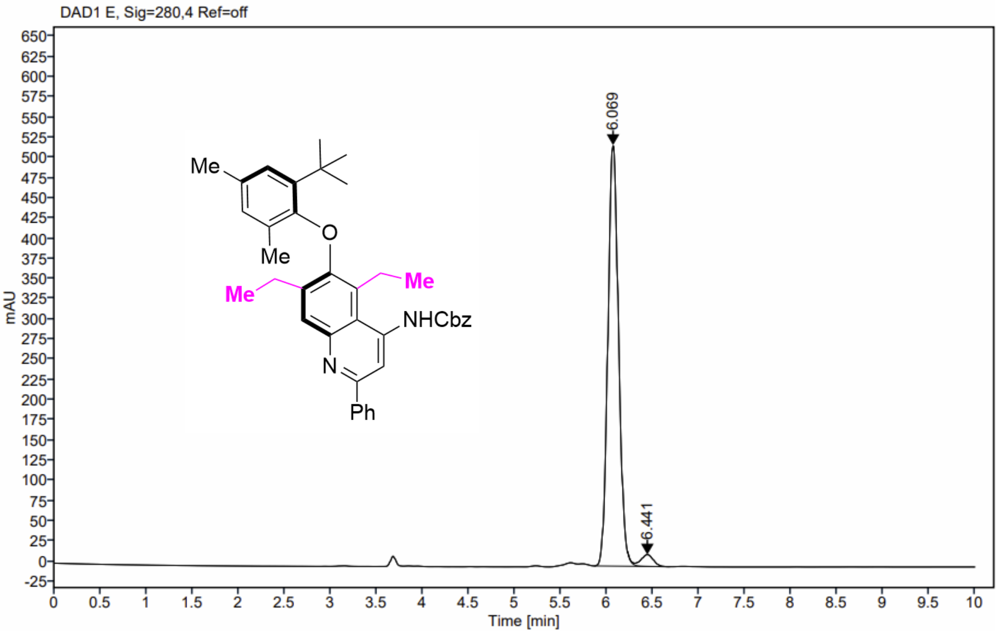

RT [min]	Width [min]	Area	Height	Area%
6.069	0.1260	4202.7861	521.4742	96.7533
6.441	0.1487	141.0303	14.6036	3.2467

Benzyl (6-(2-(tert-butyl)-4,6-dimethylphenoxy)-2,5,7-triphenylquinolin-4-yl)carbamate (**4w**)

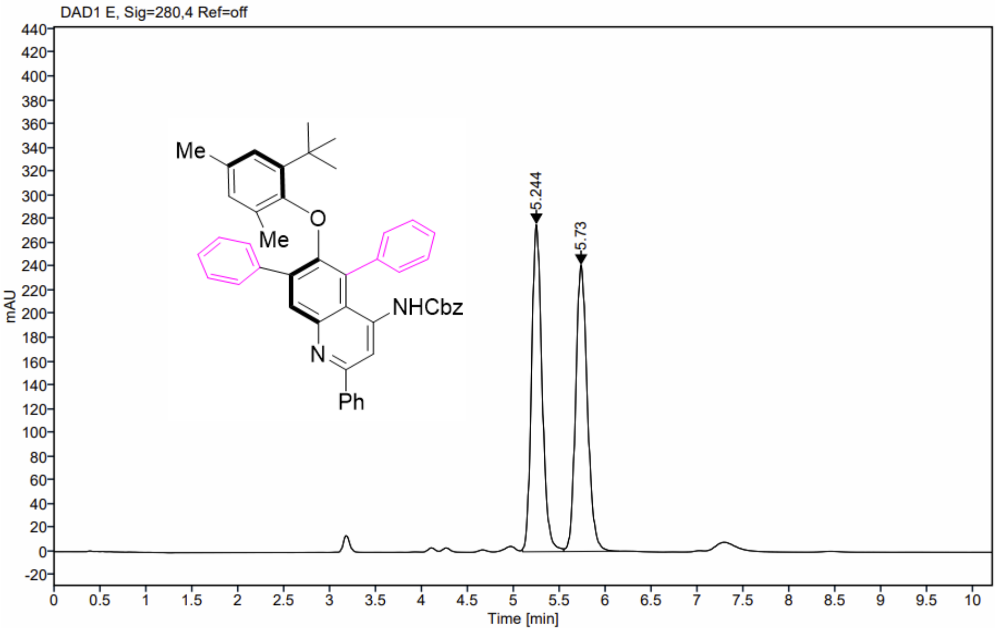

RT [min]	Width [min]	Area	Height	Area%
5.244	0.1319	2182.0945	275.7204	50.5765
5.730	0.1372	2132.3499	241.1322	49.4235

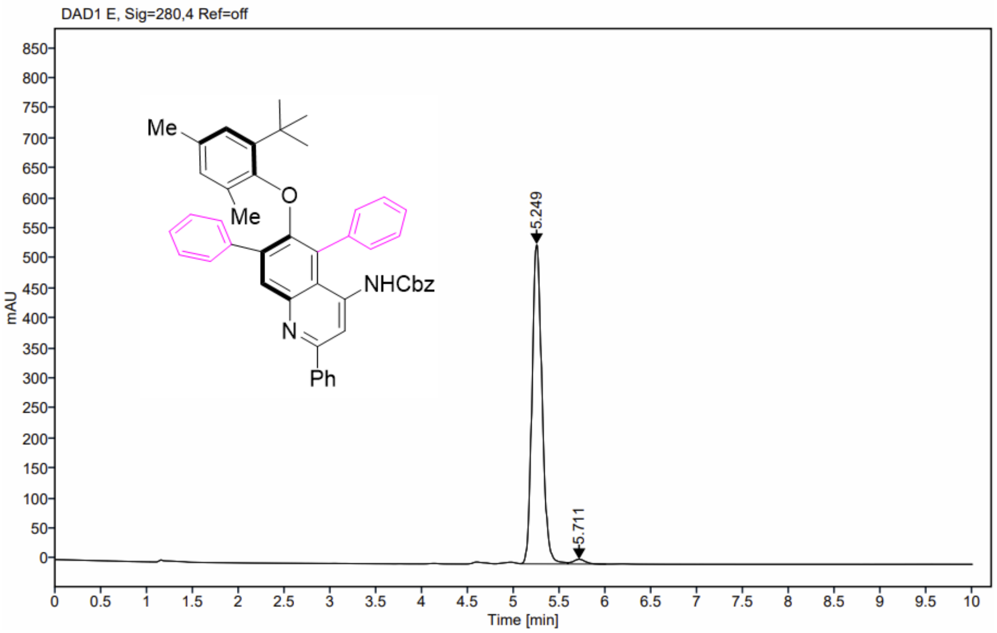

RT [min]	Width [min]	Area	Height	Area%
5.249	0.1262	4031.1233	532.5284	98.2347
5.711	0.1534	72.4384	7.8690	1.7653

Benzyl (6-(2-(tert-butyl)-4,6-dimethylphenoxy)-5,7-bis(4-methoxyphenyl)-2-phenylquinolin-4-yl)carbamate (**4x**)

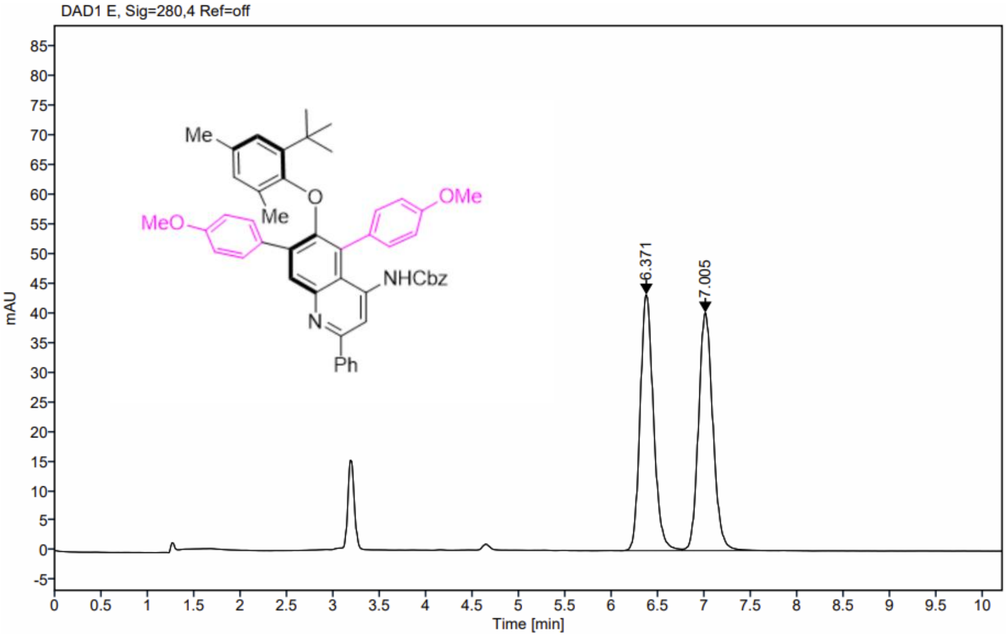

RT [min]	Width [min]	Area	Height	Area%
6.371	0.1547	431.7957	43.1688	50.0309
7.005	0.1654	431.2629	40.1168	49.9691

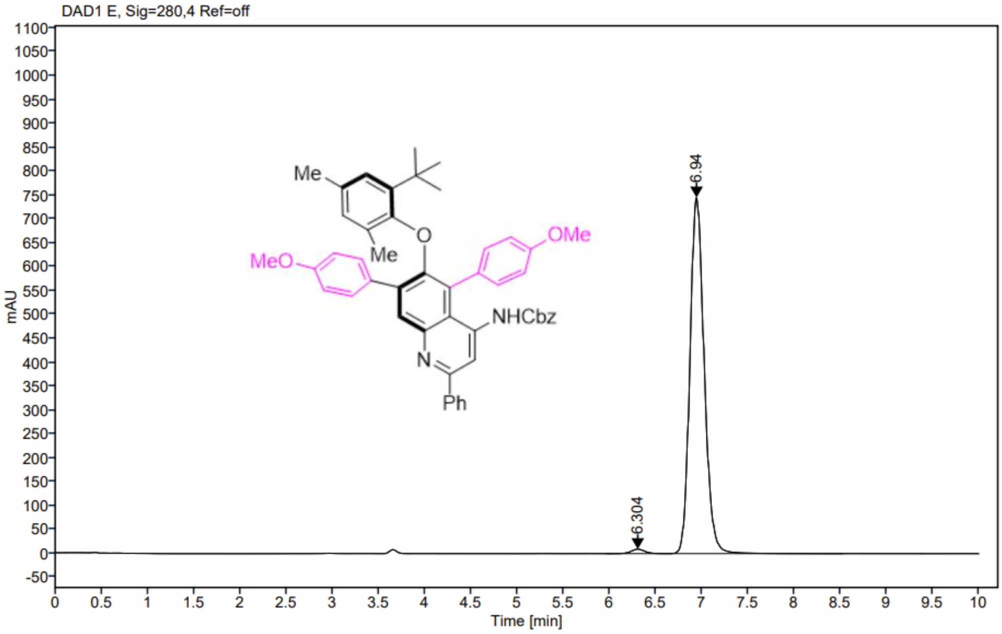

RT [min]	Width [min]	Area	Height	Area%
6.304	0.1564	93.4191	9.2072	1.1525
6.940	0.1654	8012.0195	745.3720	98.8475

(*R*)-6-(2-(tert-butyl)-4,6-dimethylphenoxy)-5,7-bis(2-methoxyphenyl)-2-phenylquinoline (**4y**)

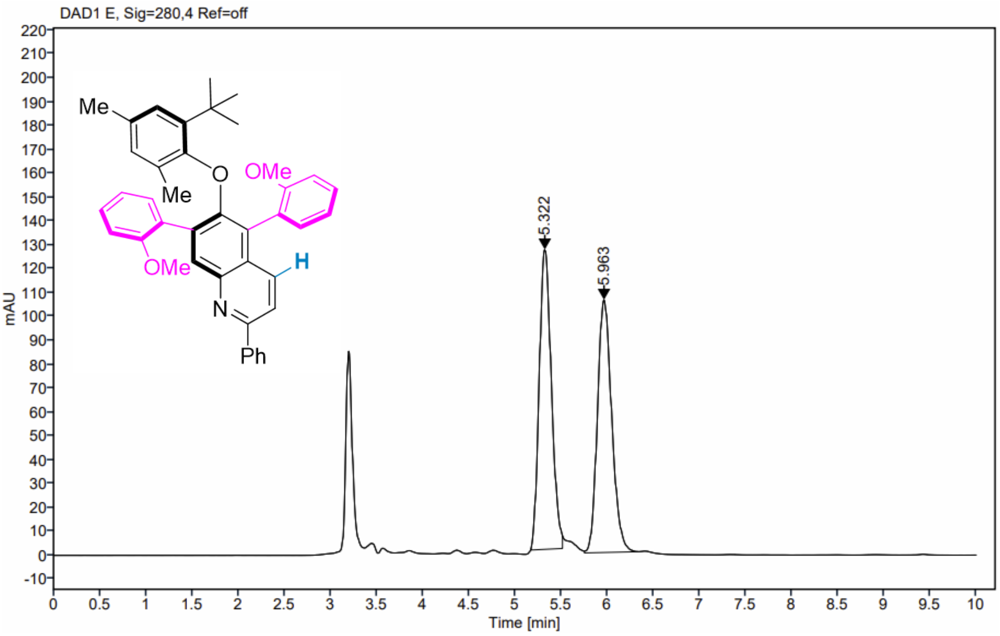

RT [min]	Width [min]	Area	Height	Area%
5.322	0.1530	1152.8192	125.6185	50.2770
5.963	0.1676	1140.1150	105.8804	49.7230

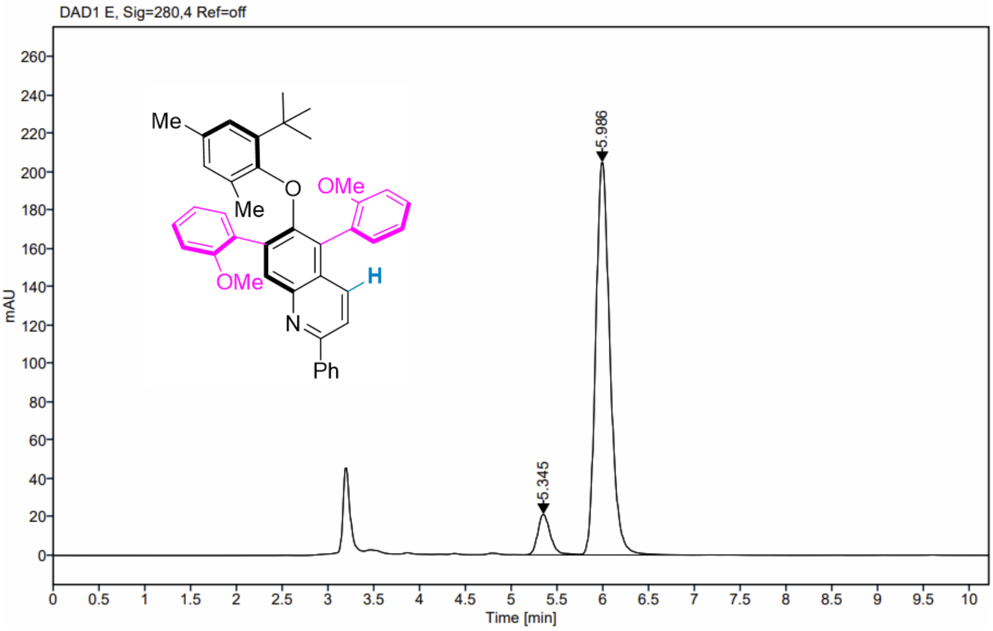

RT [min]	Width [min]	Area	Height	Area%
5.345	0.1482	203.6782	21.1782	8.3818
5.986	0.1667	2226.3269	204.9974	91.6182

Benzyl (*R*)-(6-(2-(tert-butyl)-4,6-dimethylphenoxy)-5,7-bis(2-fluorophenyl)-2-phenyl quinolin-4-yl)carbamate (**4z**)

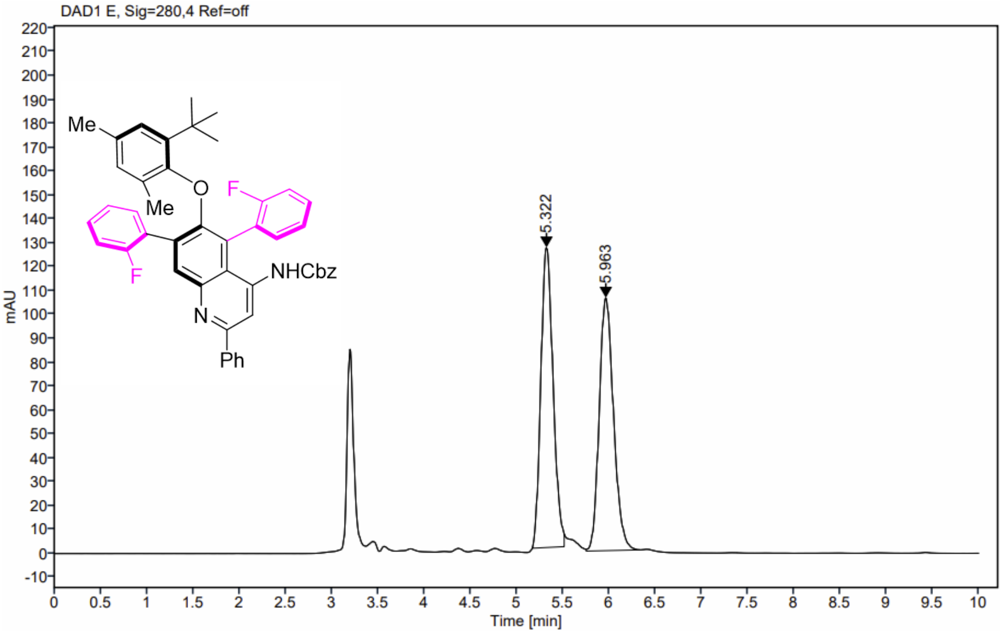

RT [min]	Width [min]	Area	Height	Area%
5.322	0.1530	1152.8192	125.6185	50.2770
5.963	0.1676	1140.1150	105.8804	49.7230

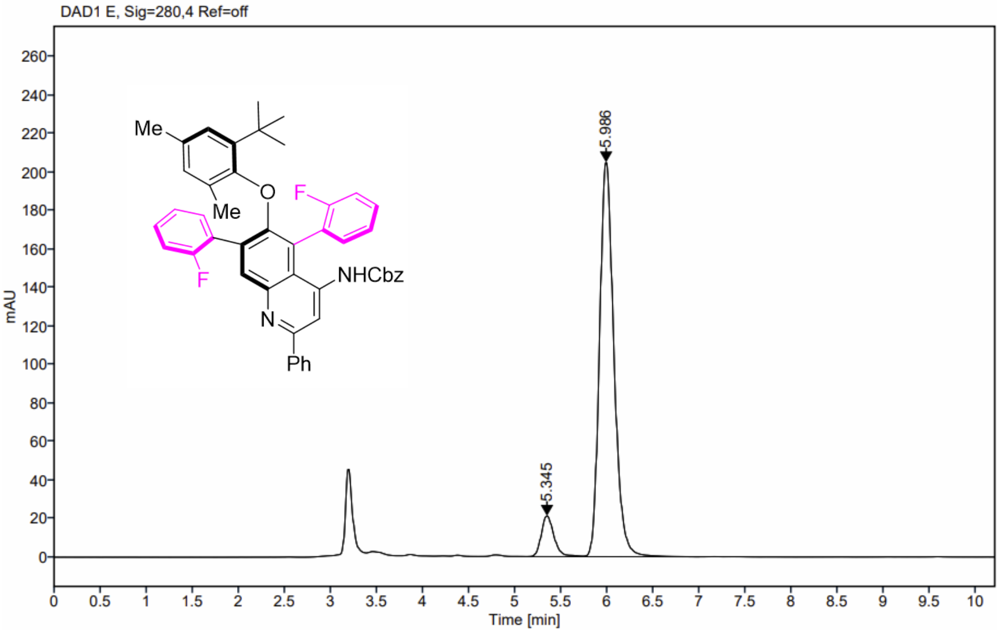

RT [min]	Width [min]	Area	Height	Area%
5.345	0.1482	203.6782	21.1782	8.3818
5.986	0.1667	2226.3269	204.9974	91.6182

Benzyl (6-(2-(tert-butyl)-4,6-dimethylphenoxy)-2-phenyl-5,7-di((E)-styryl)quinolin-4-yl)carbamate (**4aa**)

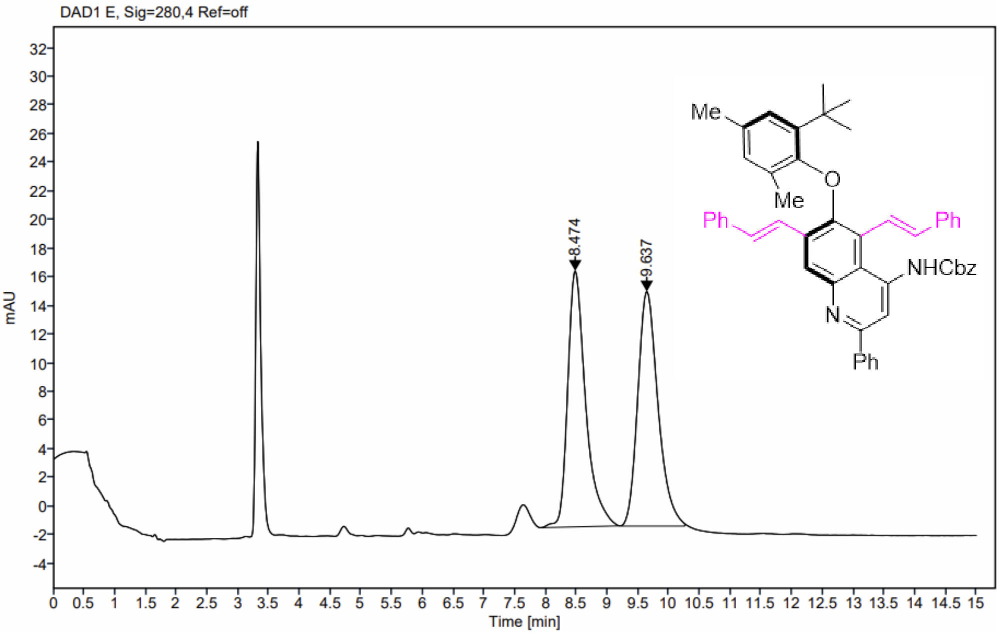

RT [min]	Width [min]	Area	Height	Area%
8.474	0.3043	361.9604	17.8707	49.0940
9.637	0.3820	375.3196	16.3764	50.9060

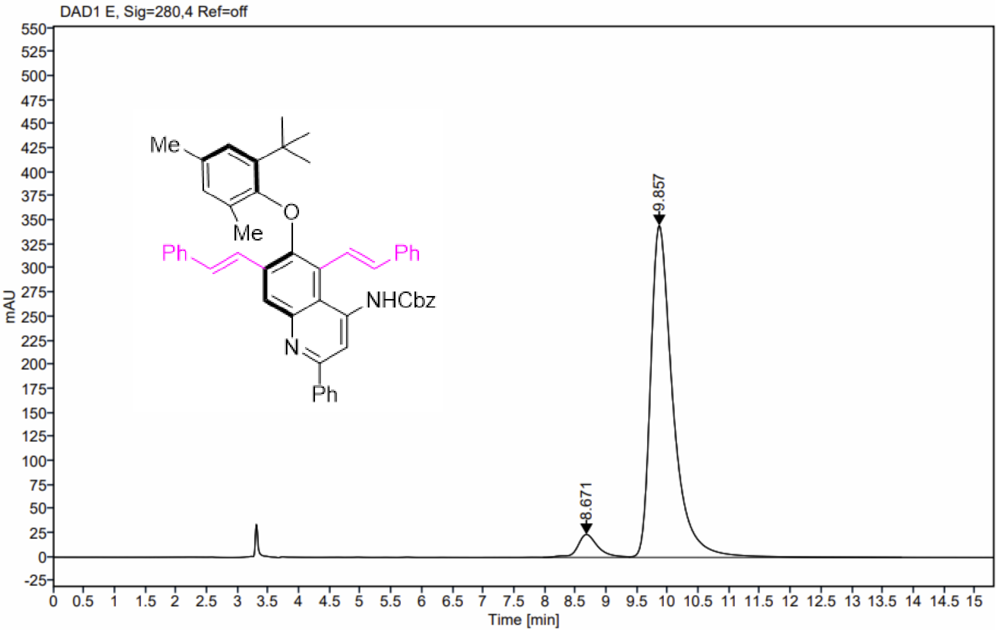

RT [min]	Width [min]	Area	Height	Area%
8.671	0.3418	547.4899	23.8504	5.8848
9.857	0.3781	8756.0439	345.0016	94.1152

Benzyl (5,7-dibromo-6-(2-(tert-butyl)-4,6-dimethylphenoxy)-2-phenylquinolin-4-yl)carbamate (**4ab**)

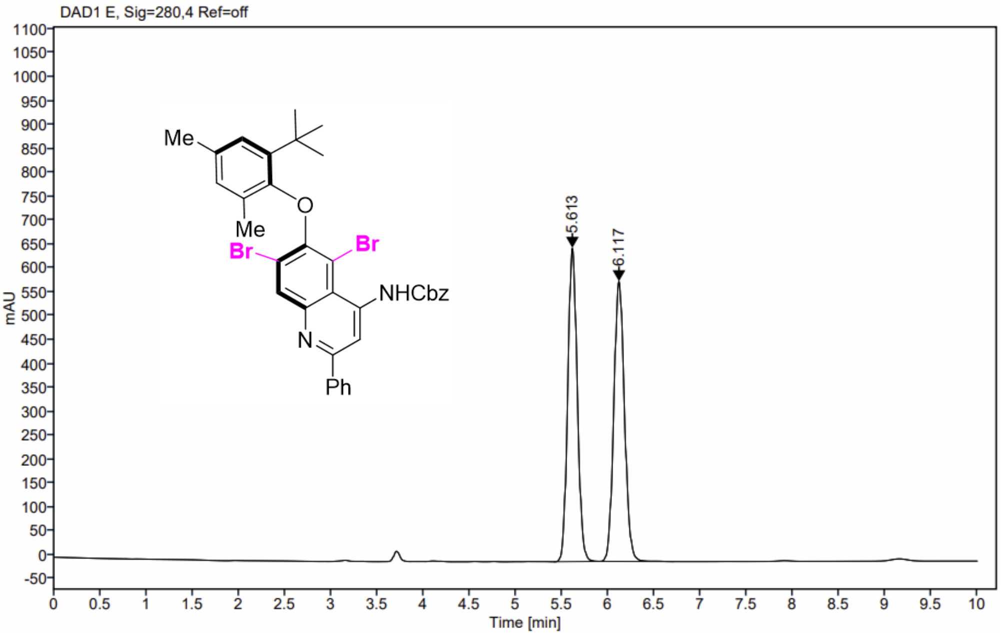

RT [min]	Width [min]	Area	Height	Area%
5.613	0.1119	4720.8721	656.3844	49.9622
6.117	0.1241	4728.0146	586.2203	50.0378

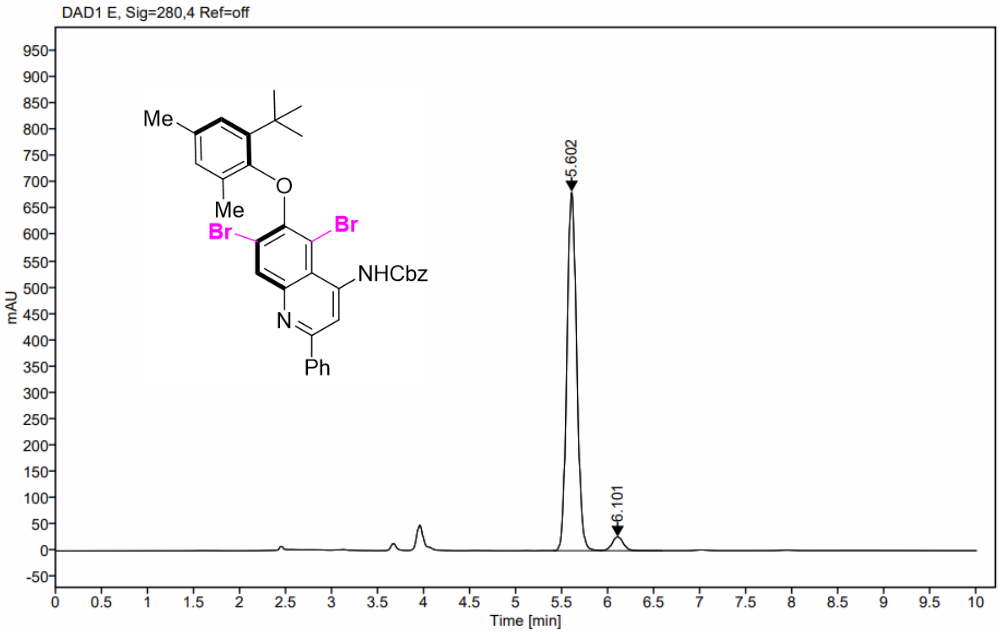

RT [min]	Width [min]	Area	Height	Area%
5.602	0.1139	5024.6650	682.0737	95.7455
6.101	0.1305	223.2719	26.4404	4.2545

Benzyl (6-(2-cyclohexyl-4,6-dimethylphenoxy)-2,5,7-triphenylquinolin-4-yl)carbamate (**4ac**)

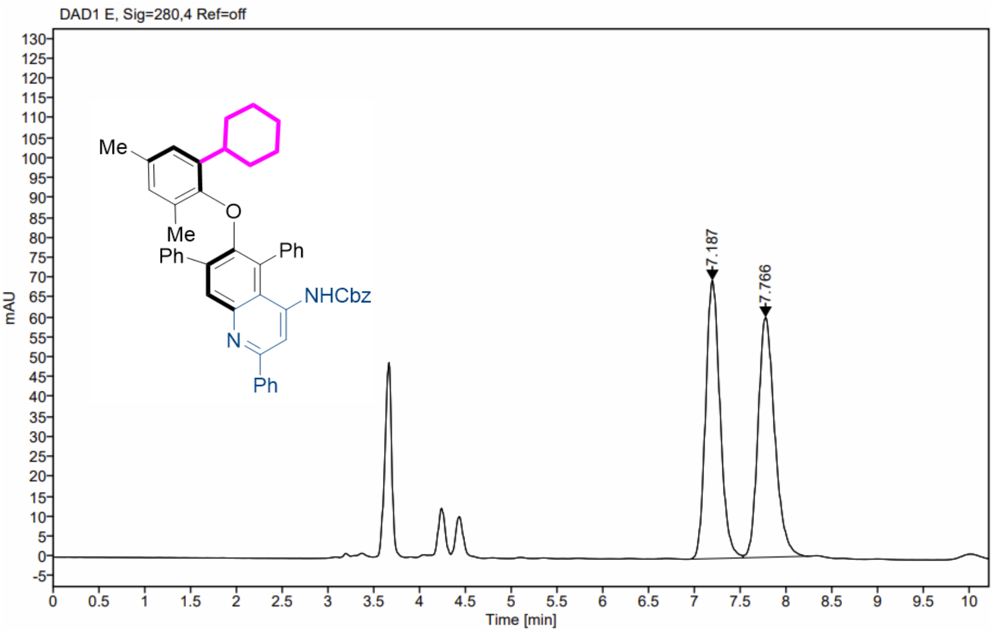

RT [min]	Width [min]	Area	Height	Area%
7.187	0.1692	761.2791	69.8002	49.9671
7.766	0.1937	762.2813	60.2234	50.0329

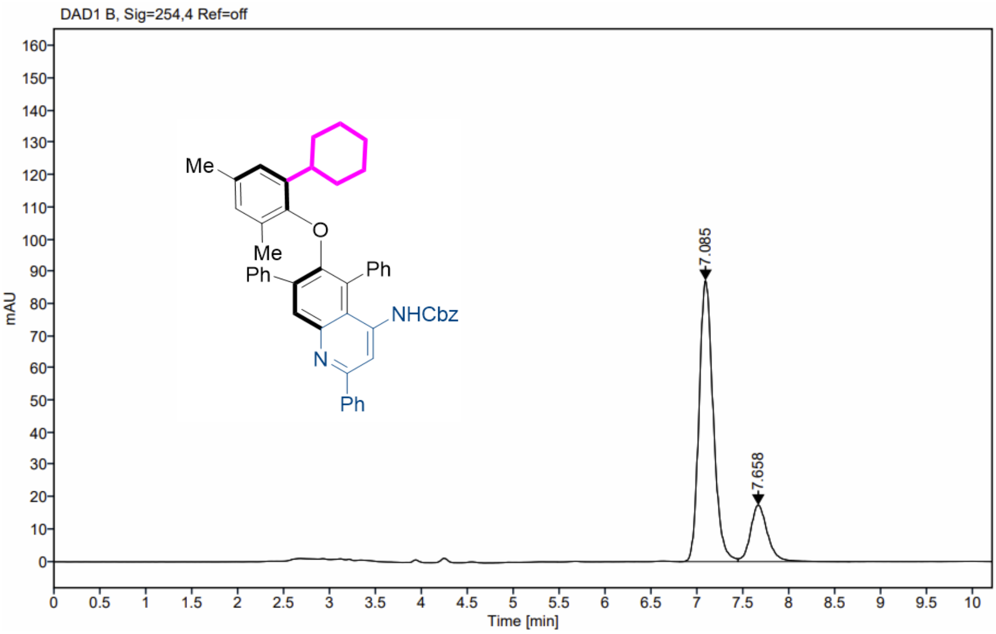

RT [min]	Width [min]	Area	Height	Area%
7.085	0.1650	935.4630	87.3329	80.5344
7.658	0.1965	226.1071	17.5223	19.4656

Benzyl ((*3R*,*4R*)-6-(2-(tert-butyl)-4,6-dimethylphenoxy)-3-iodo-5,7-dimethyl-2-phenyl-3,4-dihydroquinolin-4-yl)carbamate (**5a'**)

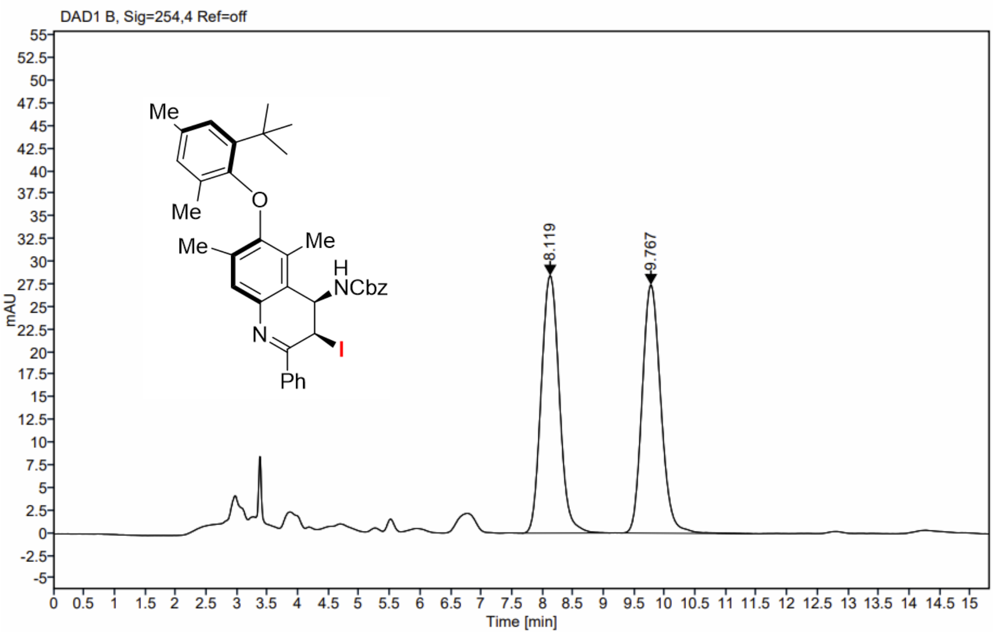

RT [min]	Width [min]	Area	Height	Area%
8.119	0.3262	586.0923	28.4619	50.2597
9.767	0.3286	580.0344	27.4440	49.7403

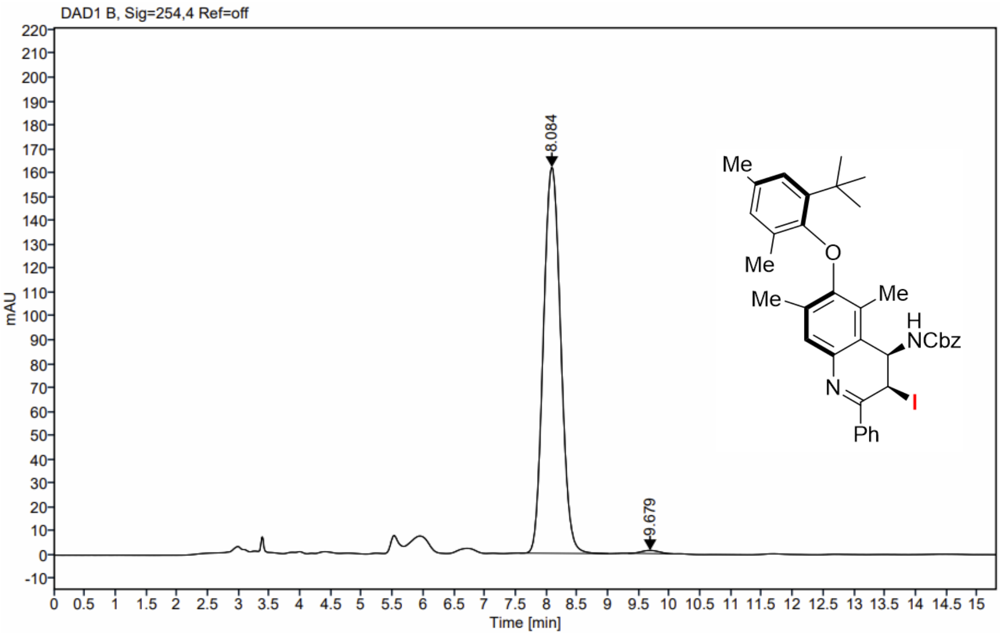

RT [min]	Width [min]	Area	Height	Area%
8.084	0.3096	3156.6606	161.7870	99.1918
9.679	0.2974	25.7211	1.3080	0.8082

6-(2-(tert-butyl)-4,6-dimethylphenoxy)-5,7-dimethyl-2-phenylquinoline (**5a**)

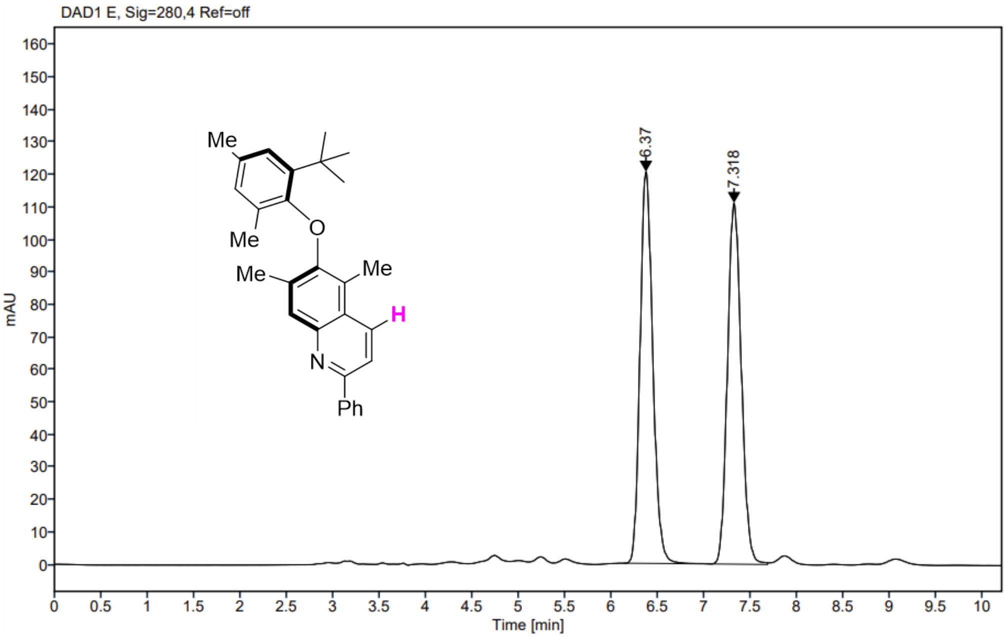

RT [min]	Width [min]	Area	Height	Area%
6.370	0.1471	1147.5576	120.5232	49.9721
7.318	0.1723	1148.8385	111.1361	50.0279

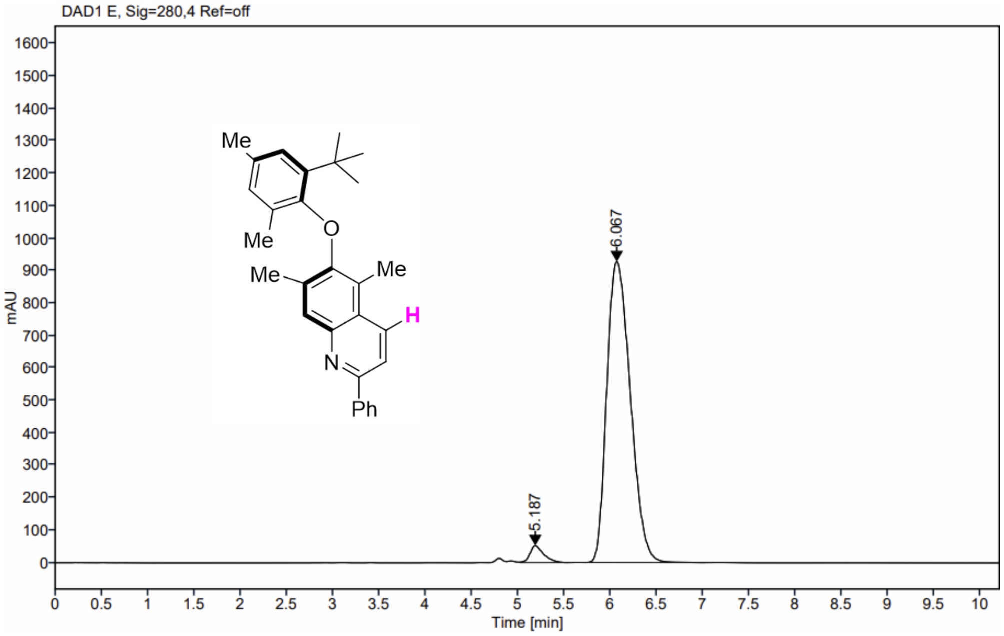

RT [min]	Width [min]	Area	Height	Area%
5.187	0.1726	537.6749	51.9339	3.1731
6.067	0.2824	16406.9629	927.2424	96.8269

Benzyl ((*2S,4S*)-8-bromo-6-(2-(tert-butyl)-4,6-dimethylphenoxy)-5,7-dimethyl-2-phenyl-1,2,3,4-tetrahydroquinolin-4-yl)carbamate (**6a**)

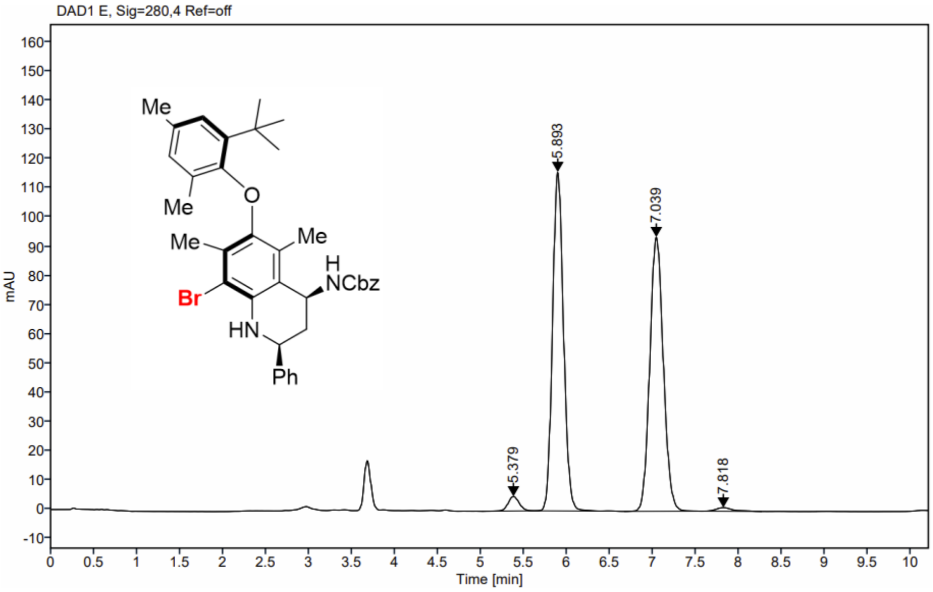

RT [min]	Width [min]	Area	Height	Area%
5.379	0.1390	44.7374	4.9709	2.1069
5.893	0.1378	1032.3514	115.9835	48.6177
7.039	0.1700	1029.6042	93.8433	48.4884
7.818	0.2004	16.7116	1.2466	0.7870

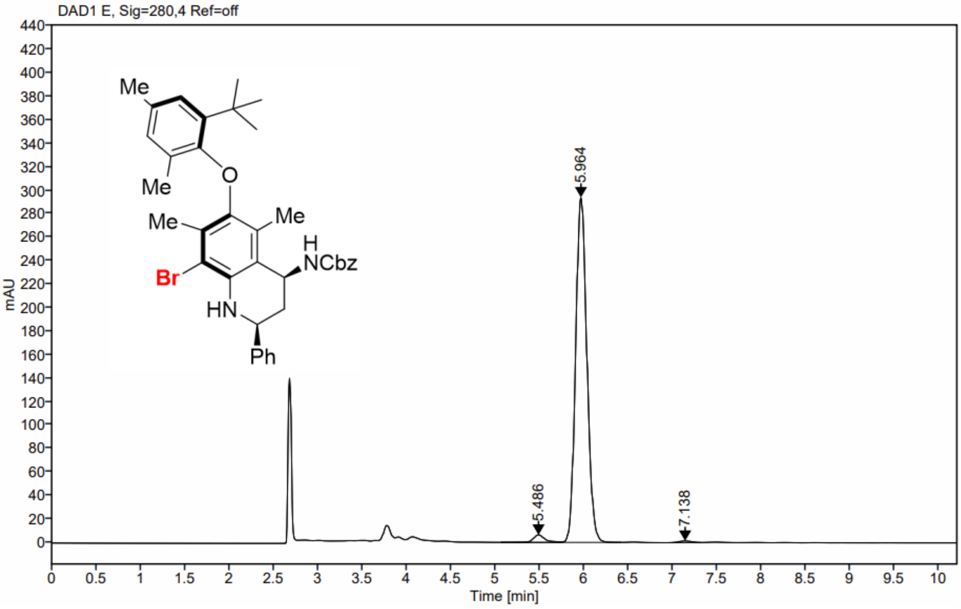

RT [min]	Width [min]	Area	Height	Area%
5.486	0.1374	58.7877	6.3868	2.2125
5.964	0.1366	2584.6453	293.7945	97.2747
7.138	0.1537	13.6240	1.3982	0.5127

Benzyl (8-bromo-6-(2-(tert-butyl)-4,6-dimethylphenoxy)-5,7-dimethyl-2-phenylquinolin-4-yl)carbamate (**7a**)

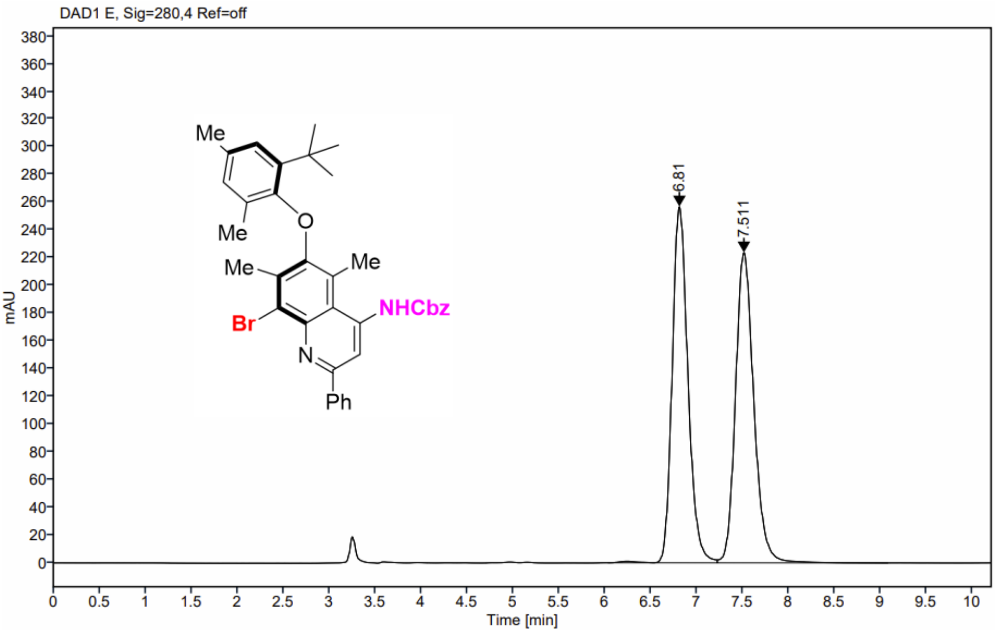

RT [min]	Width [min]	Area	Height	Area%
6.810	0.1829	3073.3899	257.0195	49.7449
7.511	0.2118	3104.9109	223.9115	50.2551

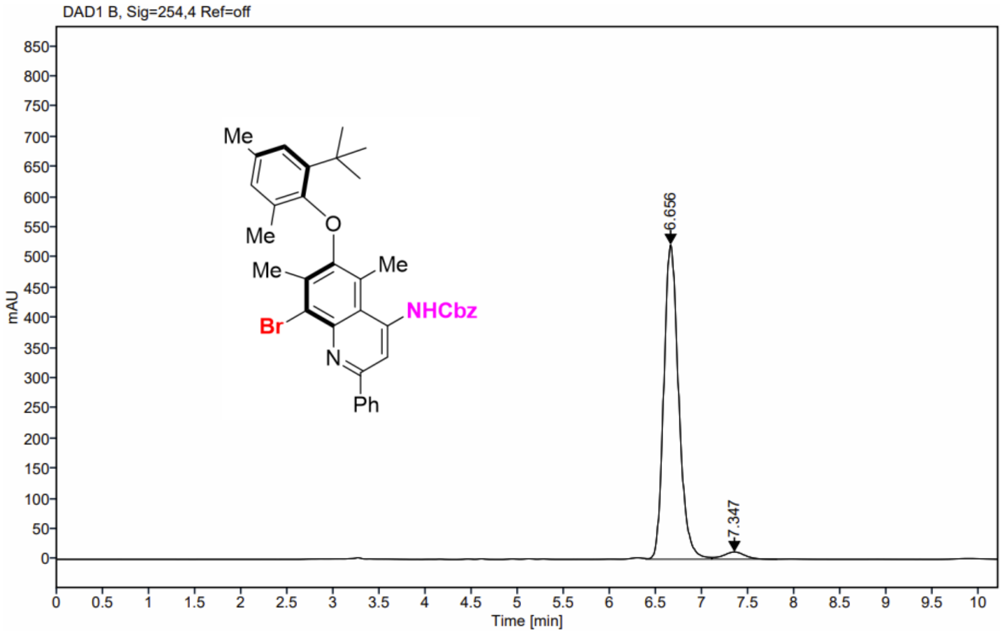

RT [min]	Width [min]	Area	Height	Area%
6.656	0.1868	5840.2881	521.1807	96.7904
7.347	0.2692	193.6680	11.9894	3.2096

8-Bromo-6-(2-(tert-butyl)-4,6-dimethylphenoxy)-5,7-dimethyl-2-phenylquinoline (**8a**)

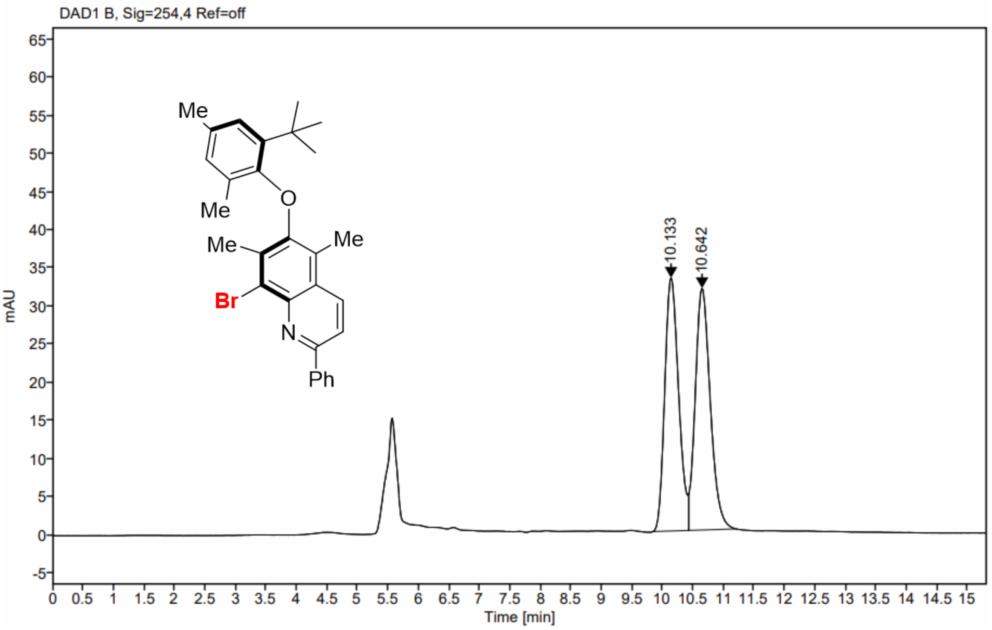

RT [min]	Width [min]	Area	Height	Area%
10.133	0.2637	524.2395	33.1390	49.1805
10.642	0.2852	541.7111	31.6562	50.8195

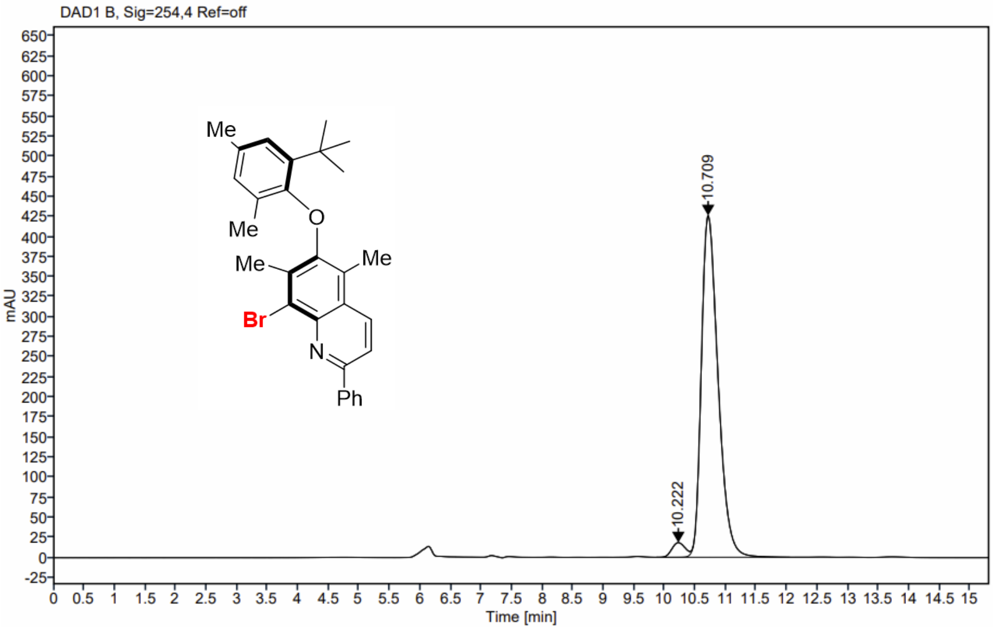

RT [min]	Width [min]	Area	Height	Area%
10.222	0.2387	277.0968	18.2961	3.2958
10.709	0.2946	8130.5820	426.0491	96.7042

3,8-Dibromo-6-(2-(tert-butyl)-4,6-dimethylphenoxy)-5,7-dimethyl-2-phenylquinoline (**9a**)

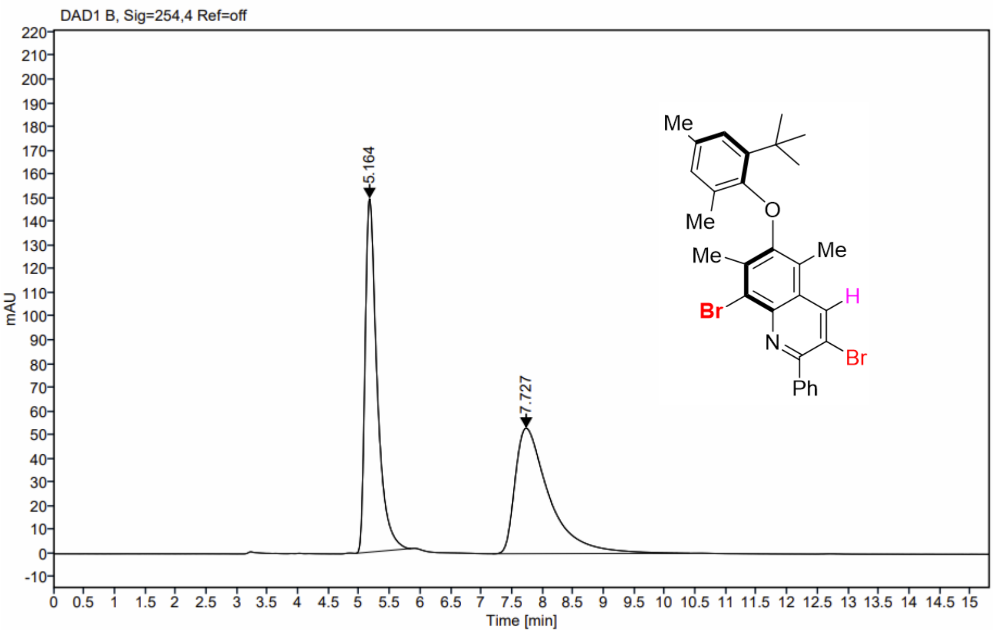

RT [min]	Width [min]	Area	Height	Area%
5.164	0.2065	2052.9731	149.1993	49.8880
7.727	0.5723	2062.1882	52.9634	50.1120

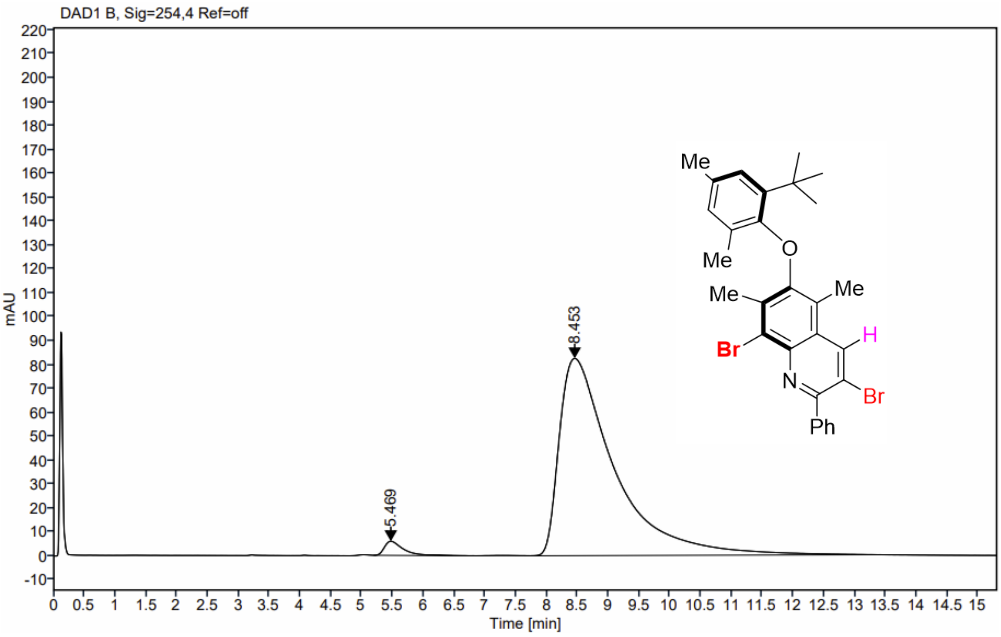

RT [min]	Width [min]	Area	Height	Area%
5.469	0.3082	125.4175	5.8942	2.3822
8.453	0.9006	5139.4307	82.5206	97.6178

6-(2-(Tert-butyl)-4,6-dimethylphenoxy)-5,7-dimethyl-2-phenylquinolin-4-amine (**10 a**)

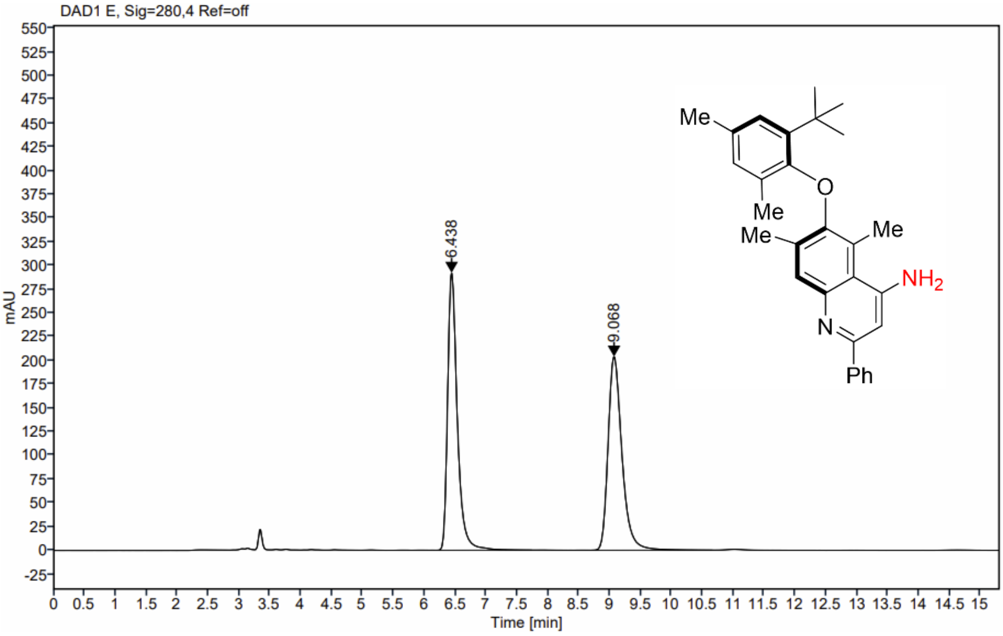

RT [min]	Width [min]	Area	Height	Area%
6.438	0.1659	3204.3237	292.3577	50.1847
9.068	0.2379	3180.7336	203.9940	49.8153

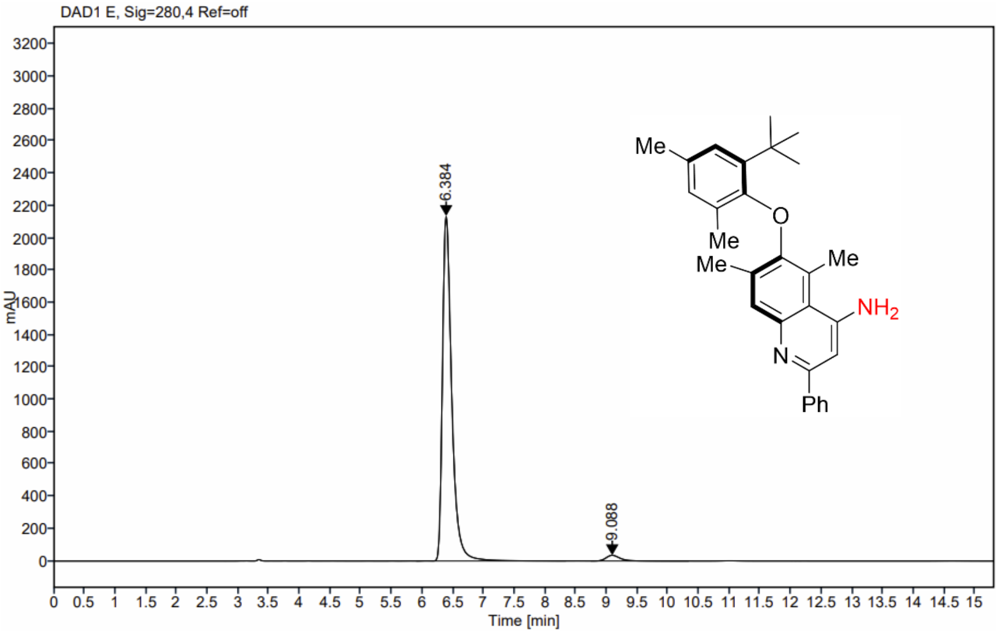

RT [min]	Width [min]	Area	Height	Area%
6.384	0.1585	22426.1289	2136.5110	97.5862
9.088	0.2421	554.7144	35.1509	2.4138

4-Bromo-6-(2-(tert-butyl)-4,6-dimethylphenoxy)-5,7-dimethyl-2-phenylquinoline (**11a**)

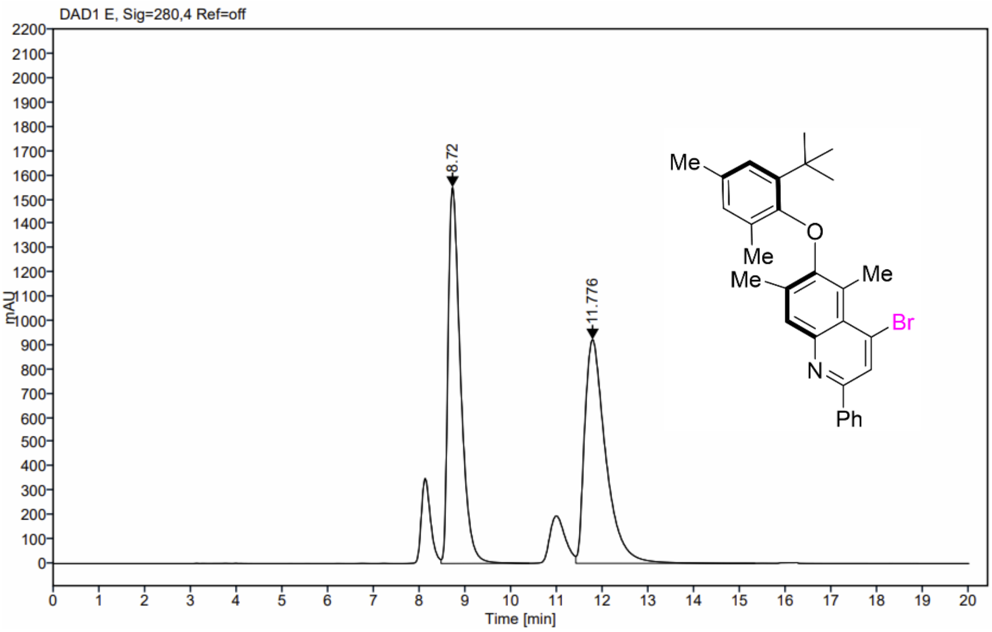

RT [min]	Width [min]	Area	Height	Area%
8.720	0.2862	29281.9199	1551.6641	49.8229
11.776	0.4888	29490.0859	922.8480	50.1771

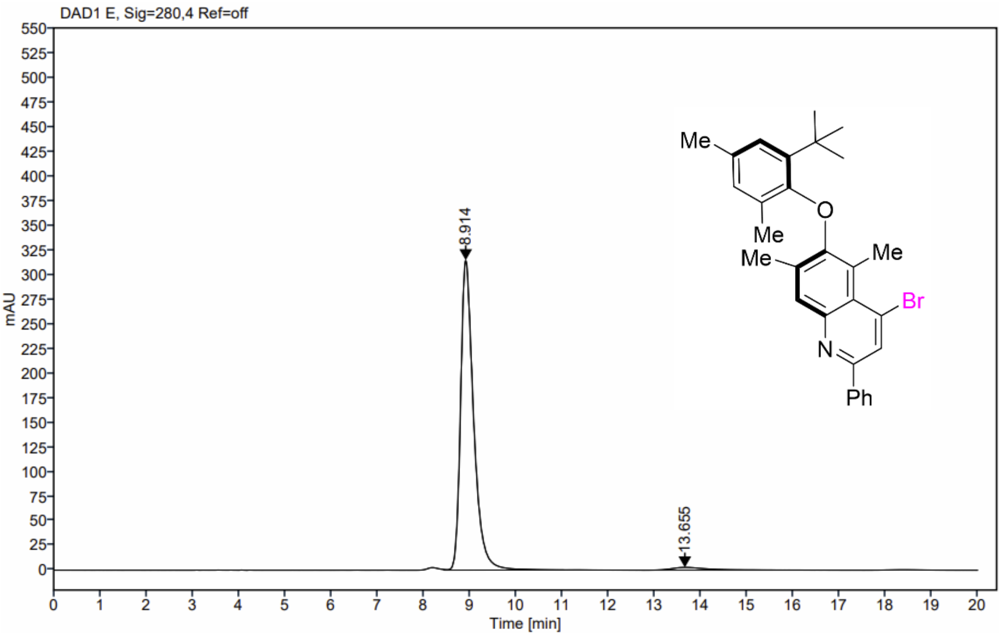

RT [min]	Width [min]	Area	Height	Area%
8.914	0.3260	6159.3896	314.9321	97.5121
13.655	0.9345	157.1491	2.8027	2.4879

6-(2-(Tert-butyl)-4,6-dimethylphenoxy)-4-iodo-5,7-dimethyl-2-phenylquinoline (**12 a**)

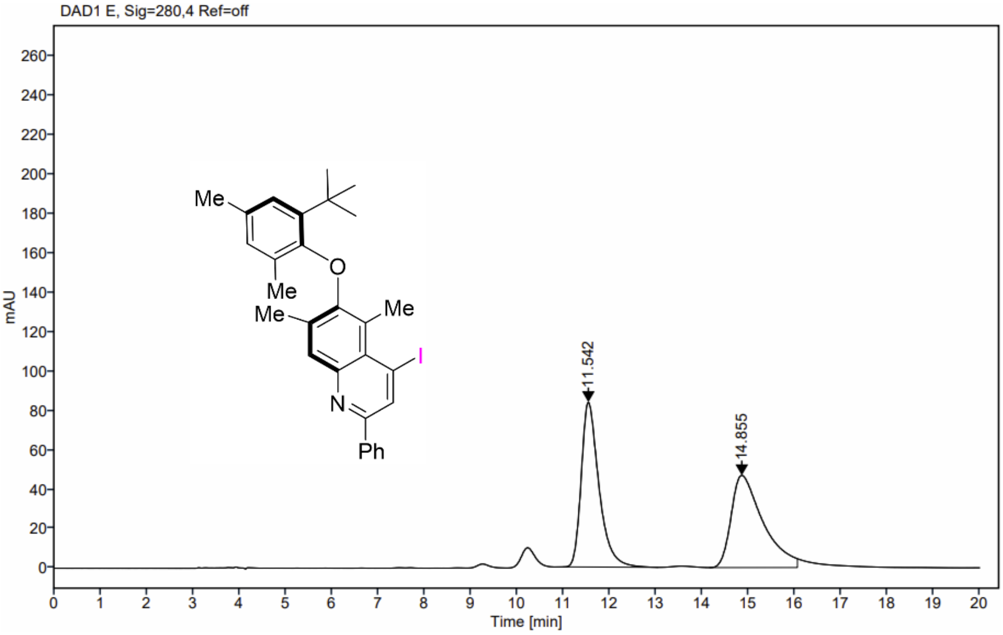

RT [min]	Width [min]	Area	Height	Area%
11.542	0.3963	2225.4077	83.6687	49.9237
14.855	0.7949	2232.2061	46.8029	50.0763

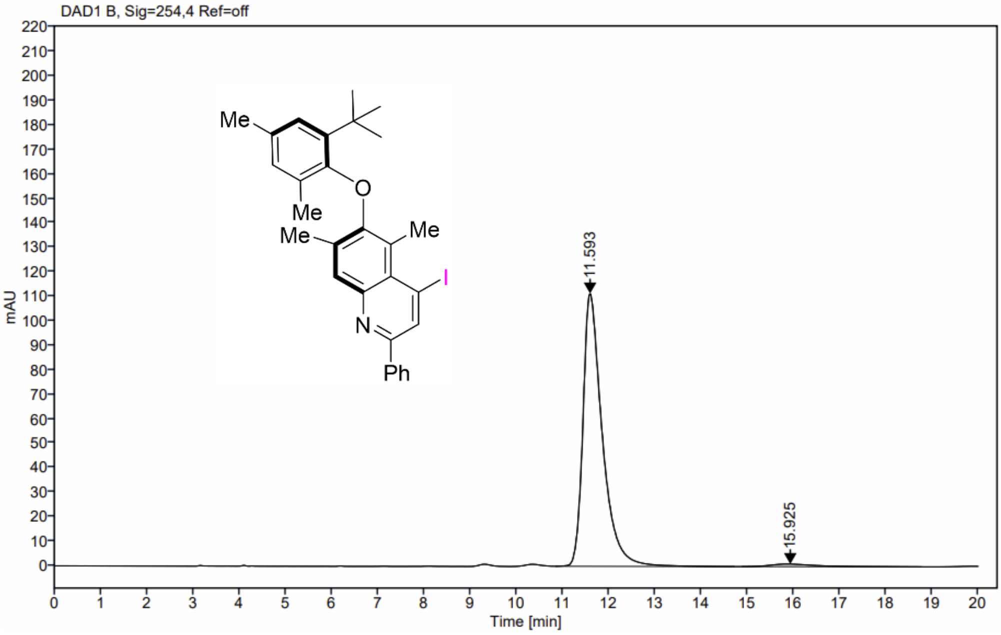

RT [min]	Width [min]	Area	Height	Area%
11.593	0.4268	3235.4238	111.4143	97.8014
15.925	1.1534	72.7319	1.0509	2.1986

3-Bromo-6-(2-(tert-butyl)-4,6-dimethylphenoxy)-5,7-dimethyl-2-phenylquinolin-4-amine (**13a**)

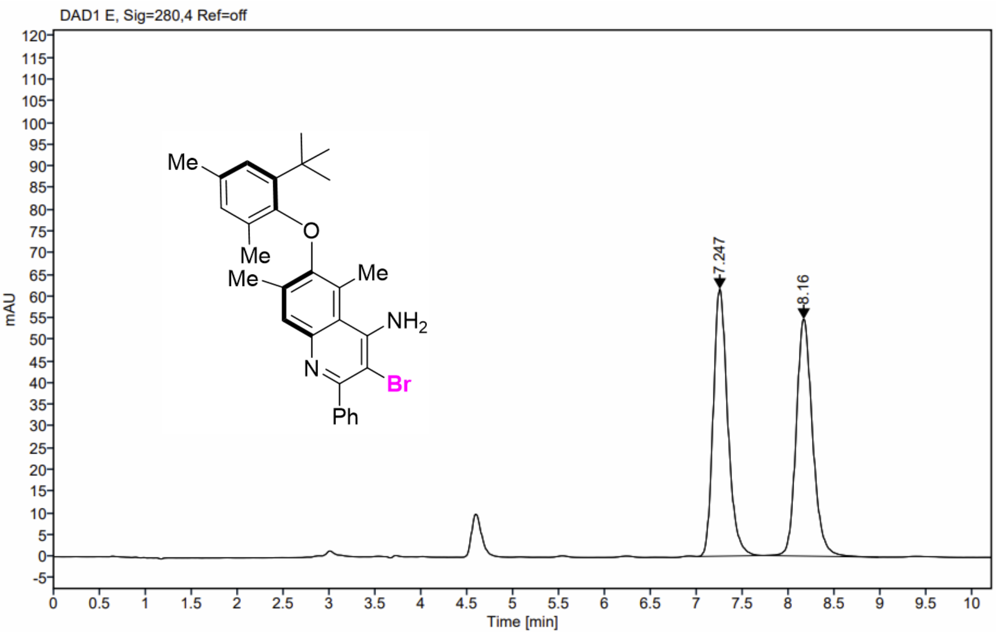

RT [min]	Width [min]	Area	Height	Area%
7.247	0.1675	673.2847	61.6081	49.9036
8.160	0.1904	675.8857	54.6171	50.0964

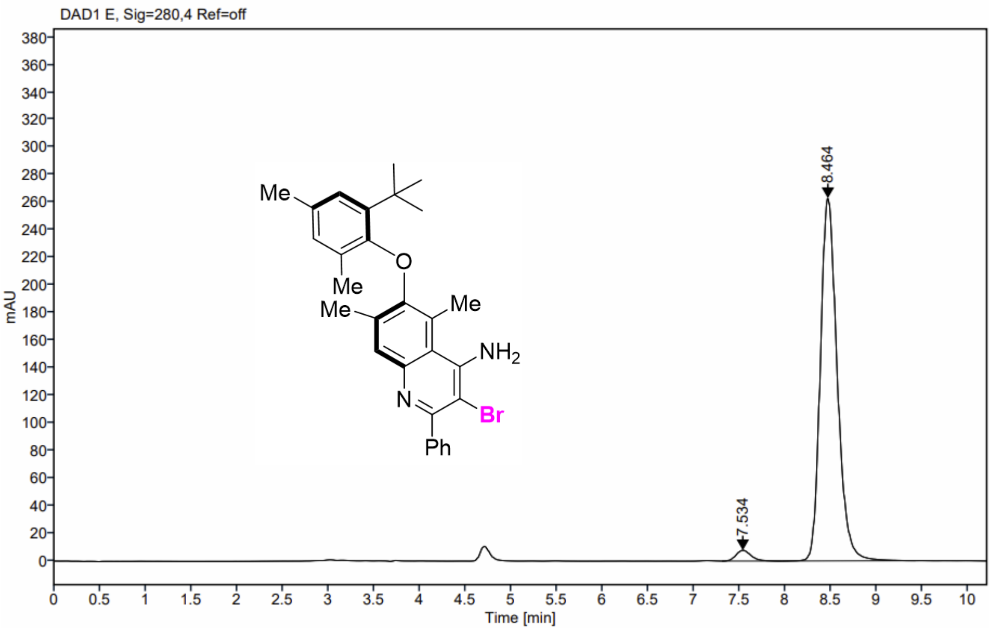

RT [min]	Width [min]	Area	Height	Area%
7.534	0.1753	88.8425	7.7767	2.5699
8.464	0.1973	3368.1333	263.1548	97.4301

6-(2-(Tert-butyl)-4,6-dimethylphenoxy)-3-iodo-5,7-dimethyl-2-phenylquinolin-4-amine (**14a**)

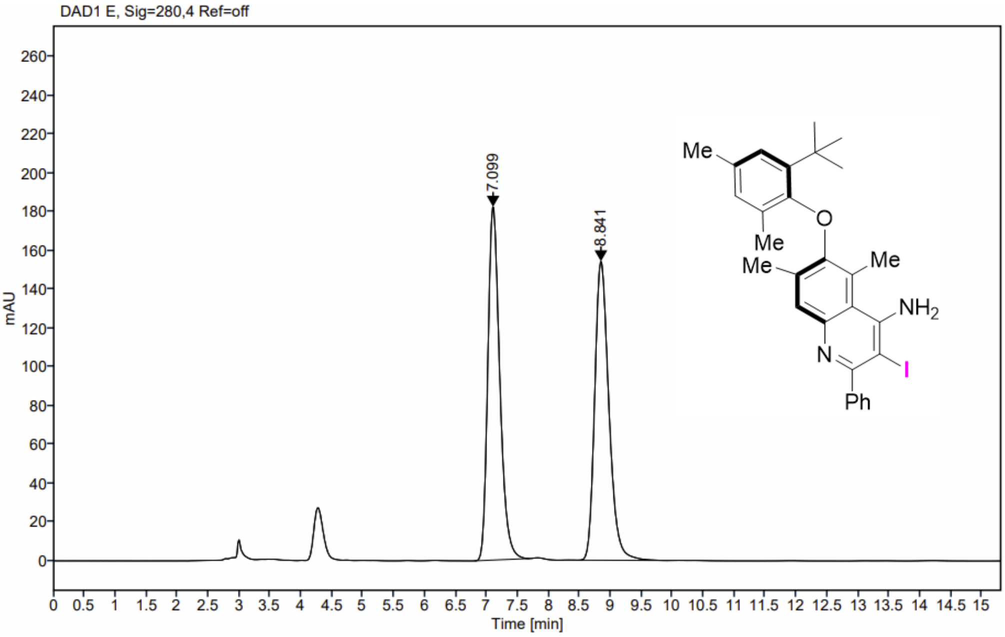

RT [min]	Width [min]	Area	Height	Area%
7.099	0.2059	2436.8784	182.3770	49.8259
8.841	0.2455	2453.9063	154.3056	50.1741

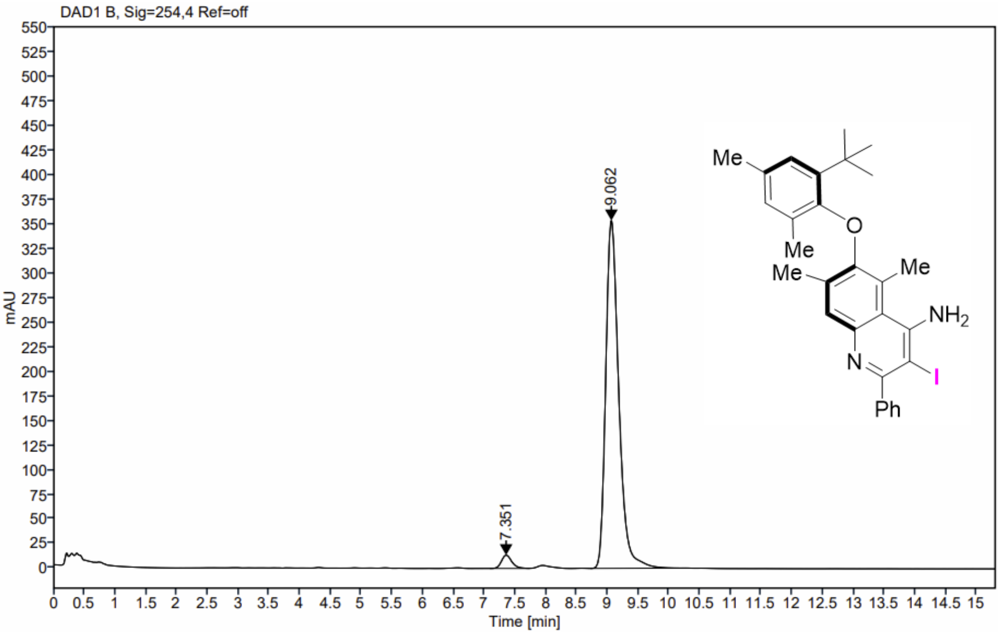

RT [min]	Width [min]	Area	Height	Area%
7.351	0.1811	158.6037	13.5005	2.9547
9.062	0.2257	5209.2021	353.9557	97.0453

6-(2-(Tert-butyl)-4,6-dimethylphenoxy)-5,7-dimethyl-2,3-diphenylquinolin-4-amine (**15a**)

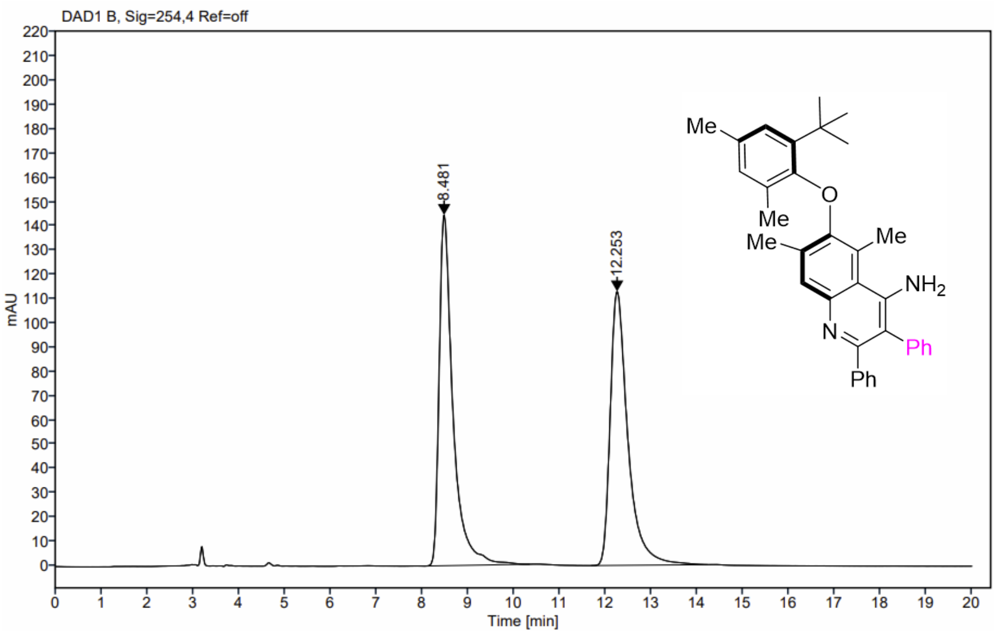

RT [min]	Width [min]	Area	Height	Area%
8.481	0.3085	3030.3594	144.6182	50.1230
12.253	0.3994	3015.4907	112.9603	49.8770

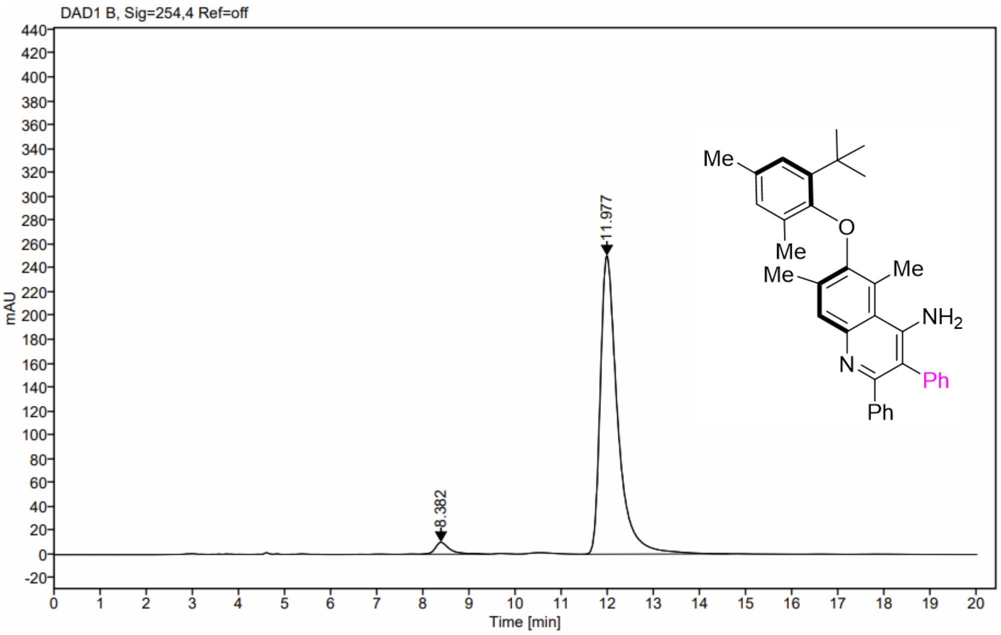

RT [min]	Width [min]	Area	Height	Area%
8.382	0.3252	220.6234	9.9312	3.2674
11.977	0.3905	6531.6030	250.2370	96.7326

8-(2-(Tert-butyl)-4,6-dimethylphenoxy)-7,9-dimethyl-4-phenyl-1H-pyrrolo[3,2-c]quinoline (**16a**)

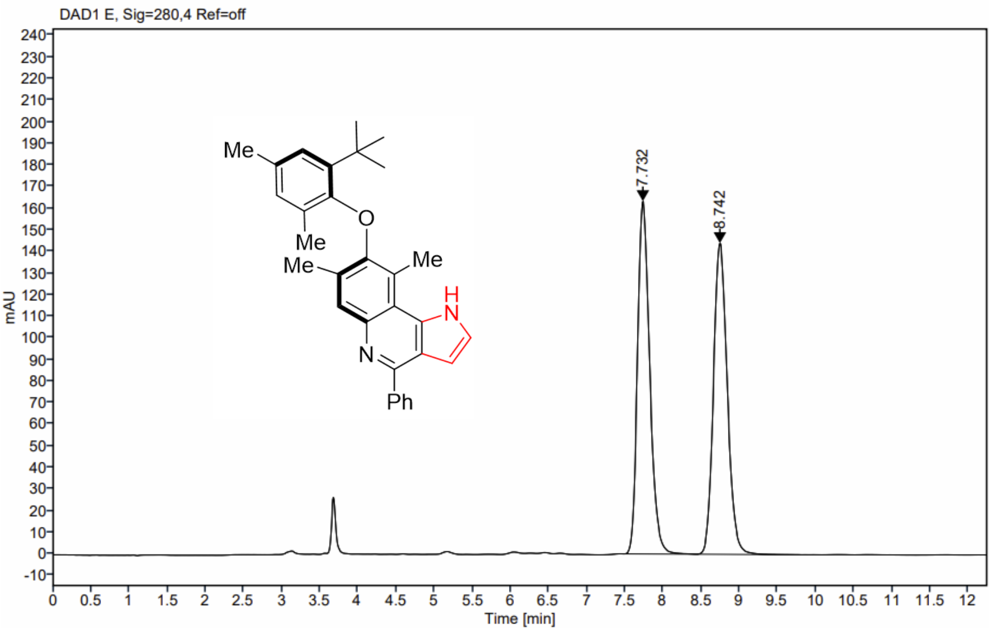

RT [min]	Width [min]	Area	Height	Area%
7.732	0.1700	1819.9124	163.3176	50.0199
8.742	0.1973	1818.4647	144.0466	49.9801

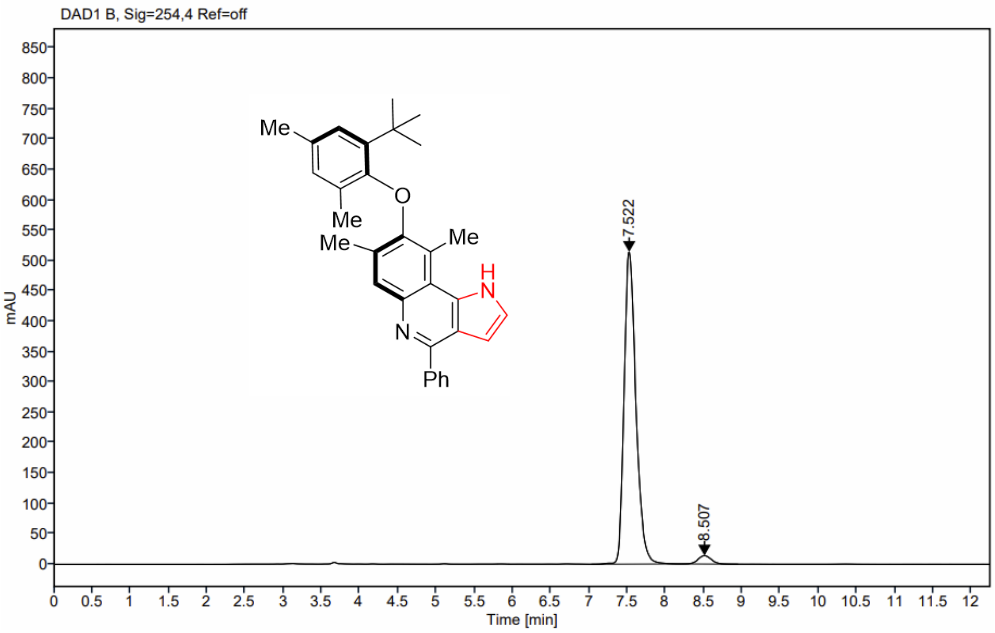

RT [min]	Width [min]	Area	Height	Area%
7.522	0.1660	5550.1191	514.0706	97.0911
8.507	0.1860	166.2820	13.8554	2.9089

6-(2-(Tert-butyl)-4,6-dimethylphenoxy)-5,7-dimethyl-2-phenyl-3-(phenylethynyl)quinolin-4-amine (**17a**)

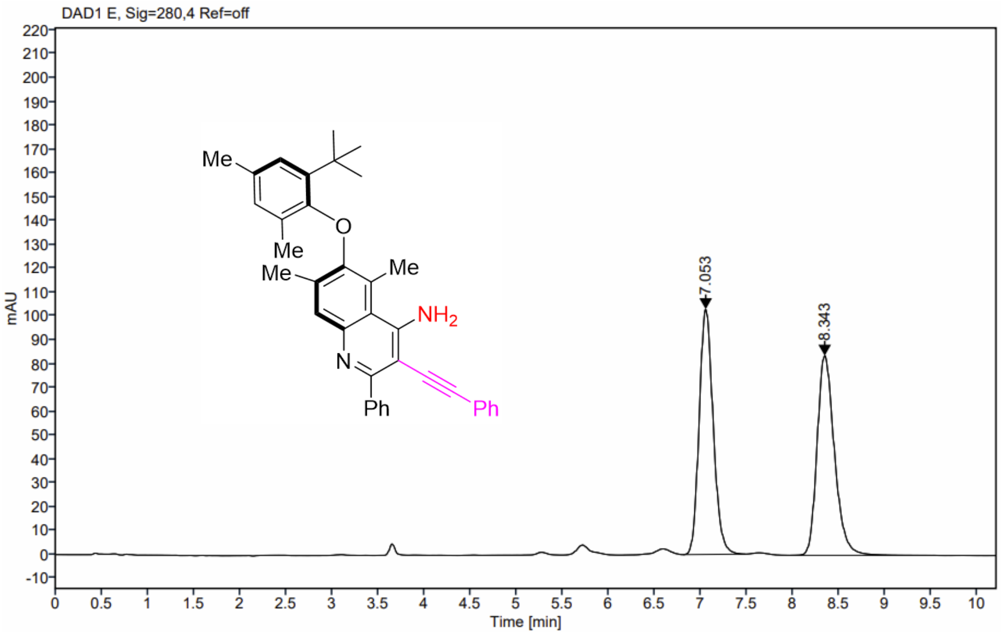

RT [min]	Width [min]	Area	Height	Area%
7.053	0.1632	1086.6345	102.9151	49.7446
8.343	0.2008	1097.7905	83.7918	50.2554

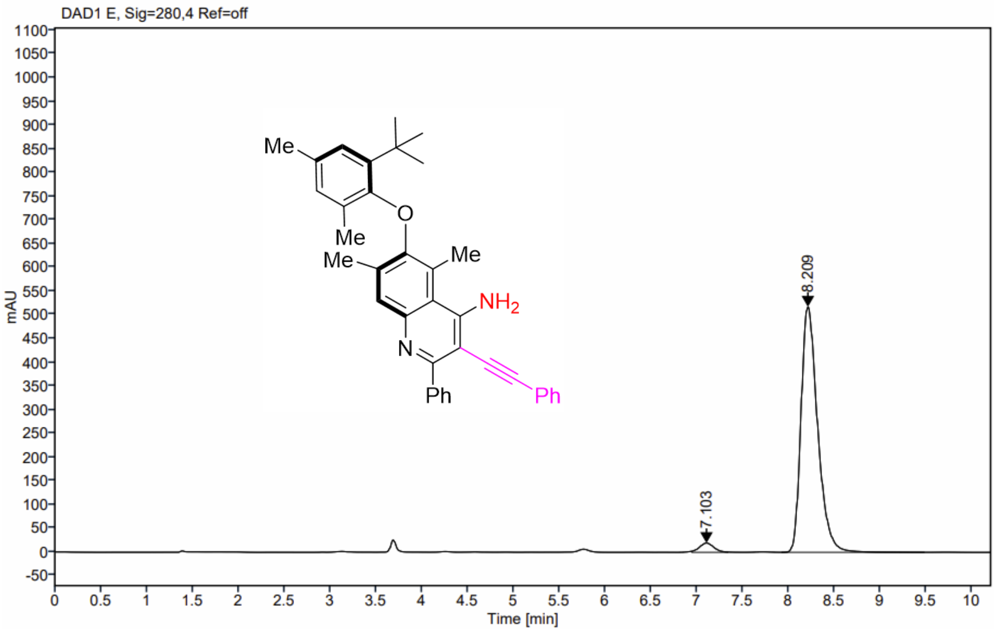

RT [min]	Width [min]	Area	Height	Area%
7.103	0.1818	212.5698	19.4922	3.1564
8.209	0.1930	6522.0103	517.6225	96.8436

8-(2-(Tert-butyl)-4,6-dimethylphenoxy)-7,9-dimethyl-2,4-diphenyl-1H-pyrrolo[3,2-c] quinoline (**18a**)

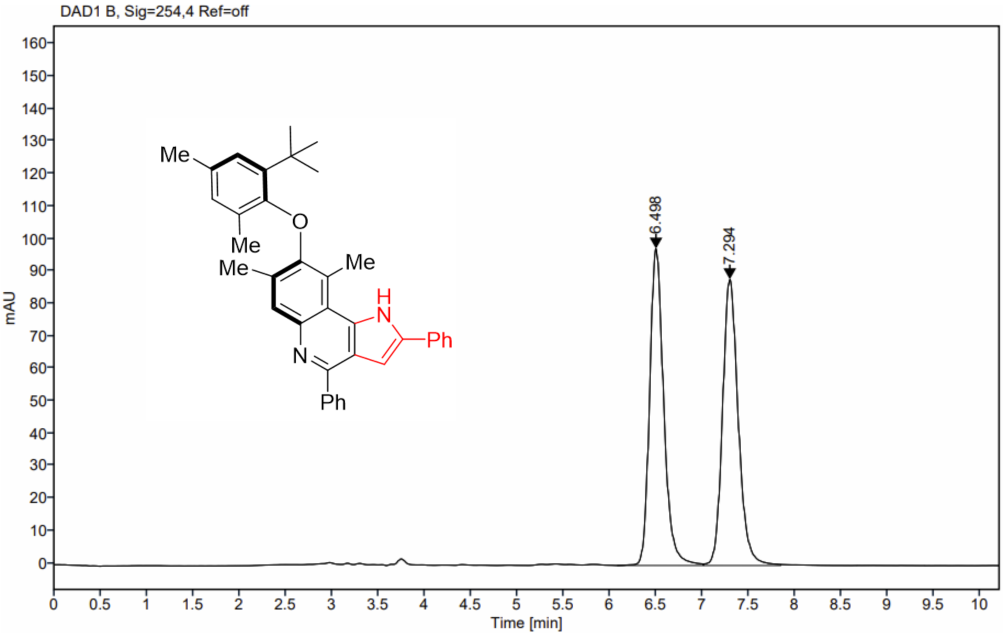

RT [min]	Width [min]	Area	Height	Area%
6.498	0.1618	1036.0564	97.6363	49.8566
7.294	0.1969	1042.0156	88.1986	50.1434

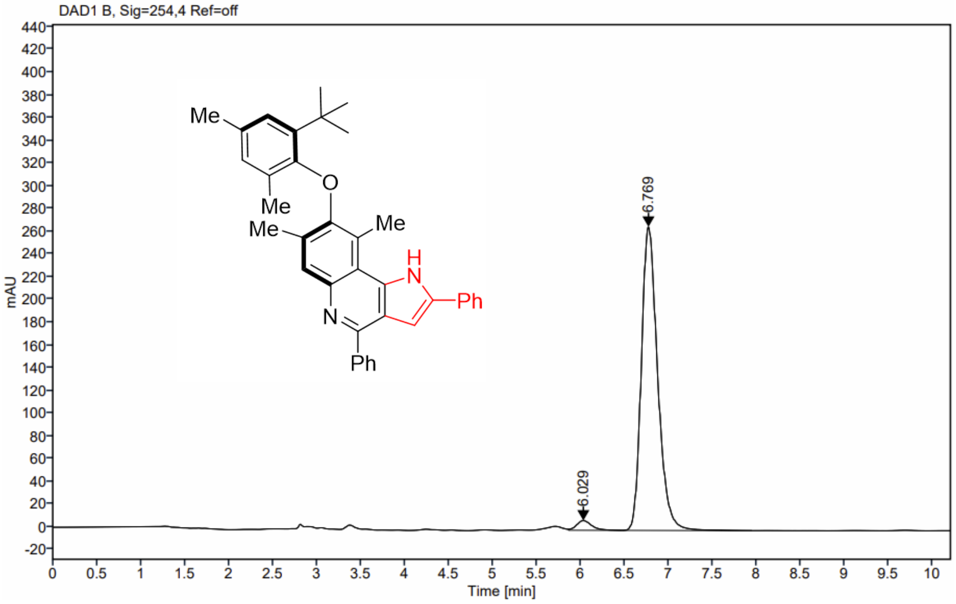

RT [min]	Width [min]	Area	Height	Area%
6.029	0.1815	101.9700	8.5293	2.8795
6.769	0.1982	3439.3240	267.1265	97.1205

NMR spectra

1,3-dibromo-2-(2-(tert-butyl)-4,6-dimethylphenoxy)-5-nitrobenzene (**S3ab**)

S3ab: ^{1}H NMR (500 MHz, Chloroform-*d*)

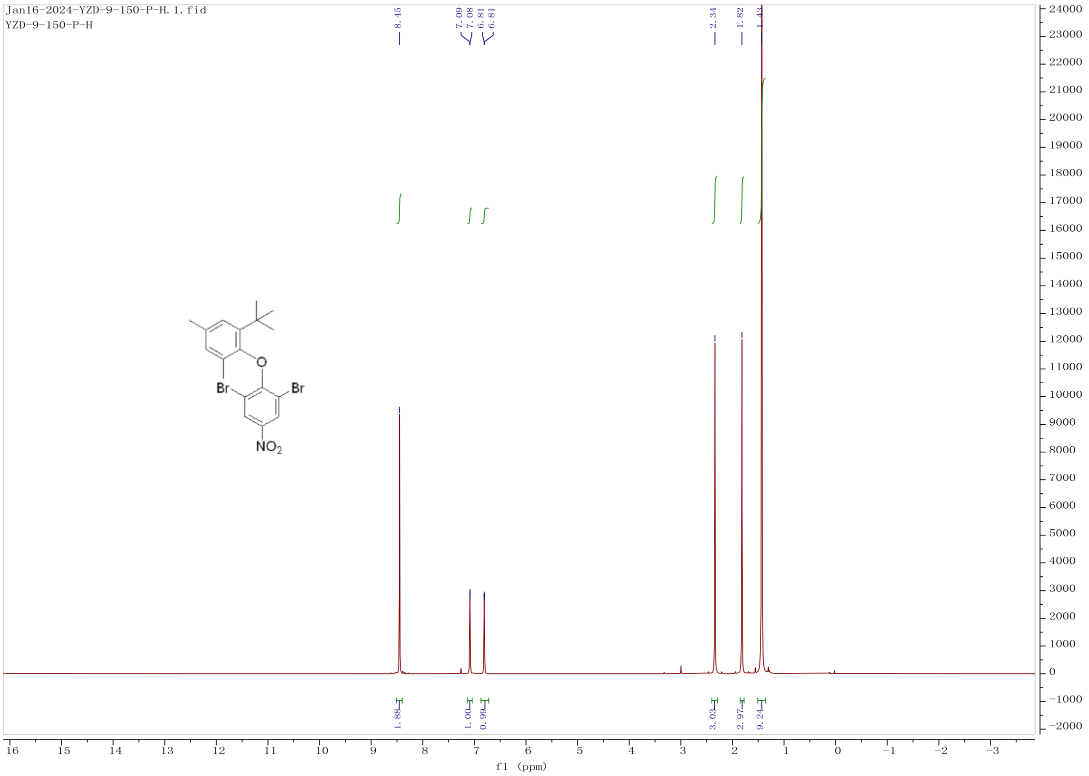

S3ab: ^{13}C NMR (126 MHz, Chloroform-*d*)

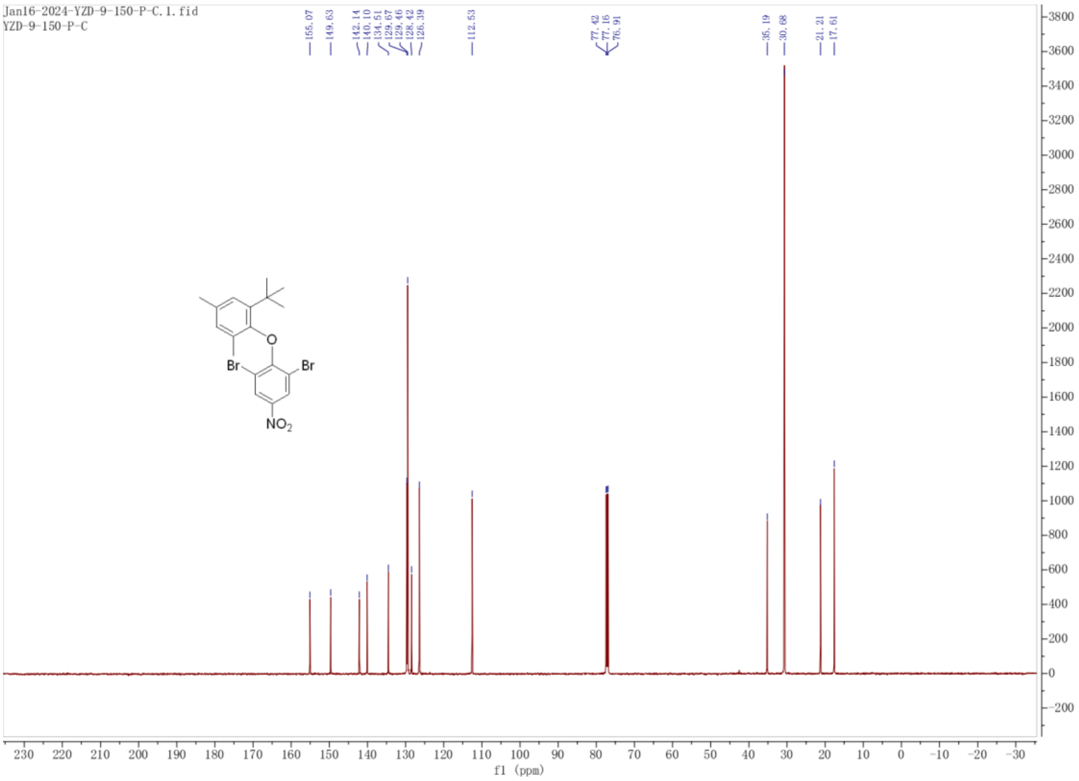

4-(2-(tert-butyl)-4,6-dimethylphenoxy)-3,5-dimethylaniline (**1a**)

1a: ^{1}H NMR (500 MHz, Chloroform-*d*)

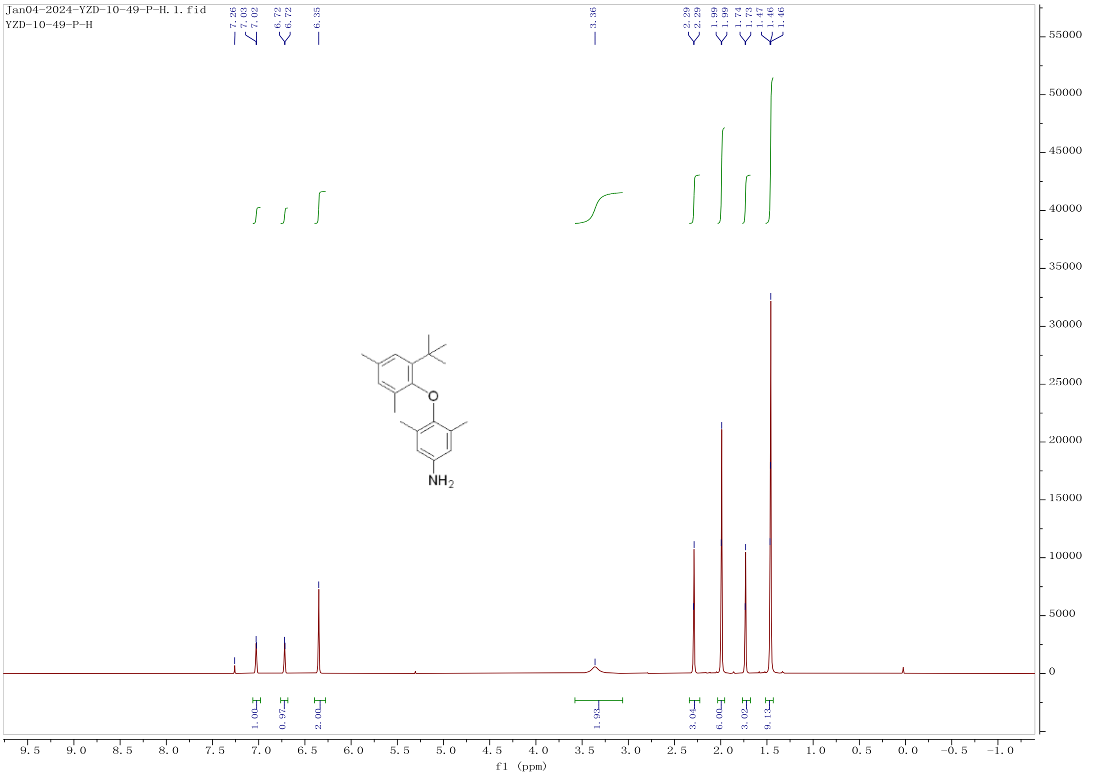

1a: ^{13}C NMR (126 MHz, Chloroform-*d*)

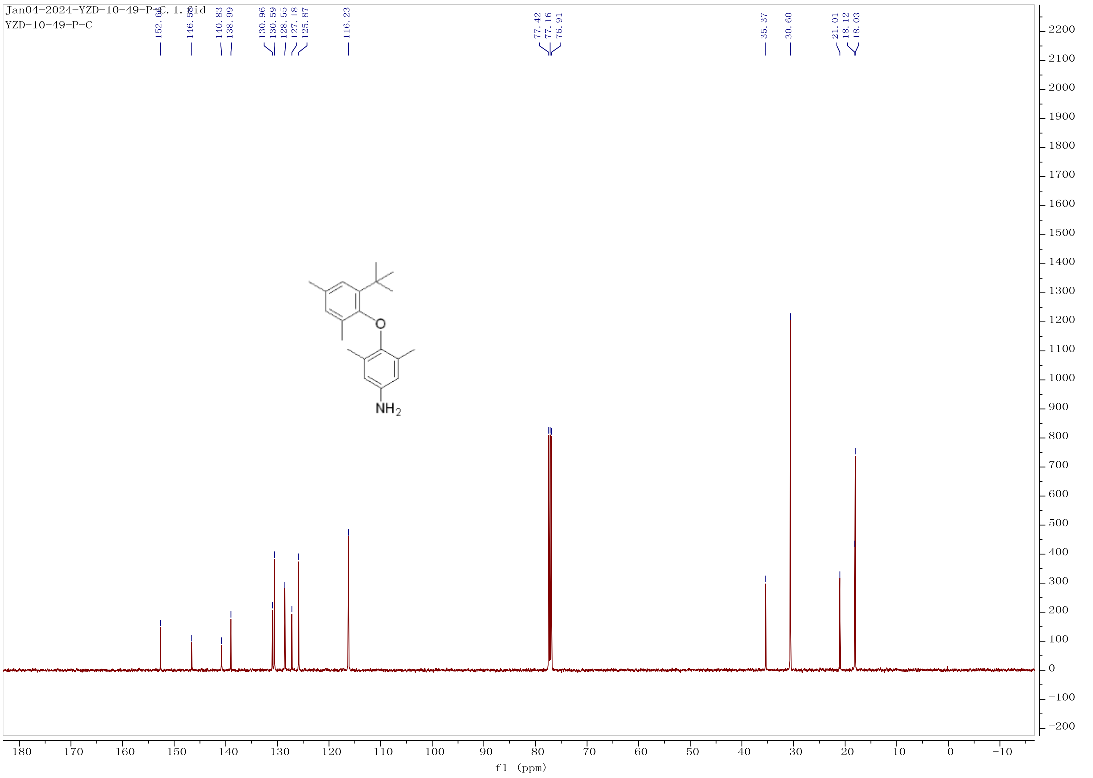

4-((3-(tert-butyl)-5-methyl-[1,1'-biphenyl]-2-yl)oxy)-3,5-dimethylaniline (**1o**)

1o: ^{1}H NMR (500 MHz, Chloroform-*d*)

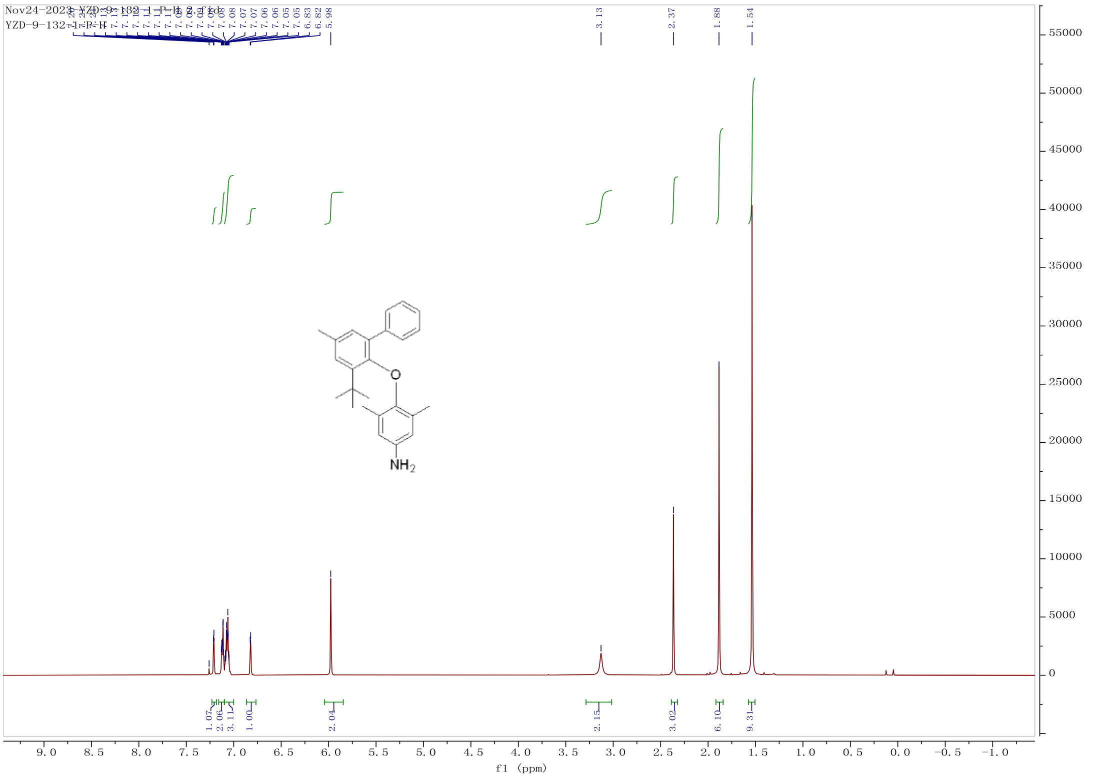

1o: ^{13}C NMR (126 MHz, Chloroform-*d*)

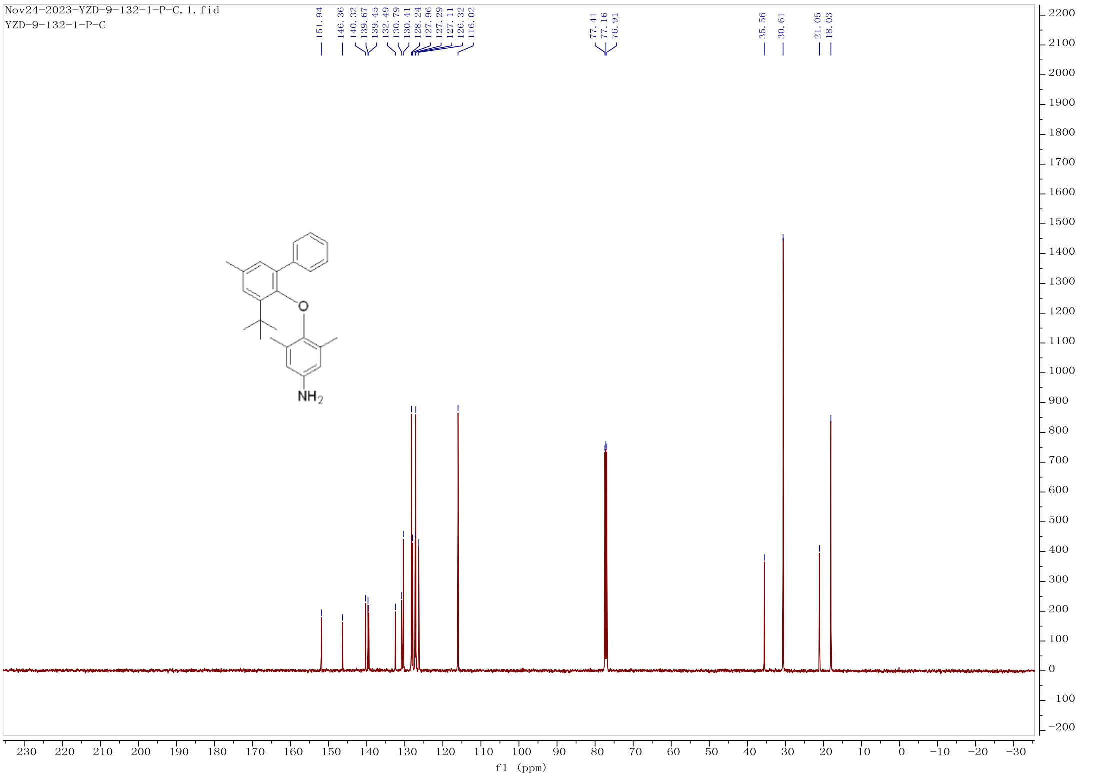

4-((3-(tert-butyl)-5-methyl-3'-(trifluoromethyl)-[1,1'-biphenyl]-2-yl)oxy)-3,5-dimethylaniline (**1p**)

1p: ^{1}H NMR (500 MHz, Chloroform-*d*)

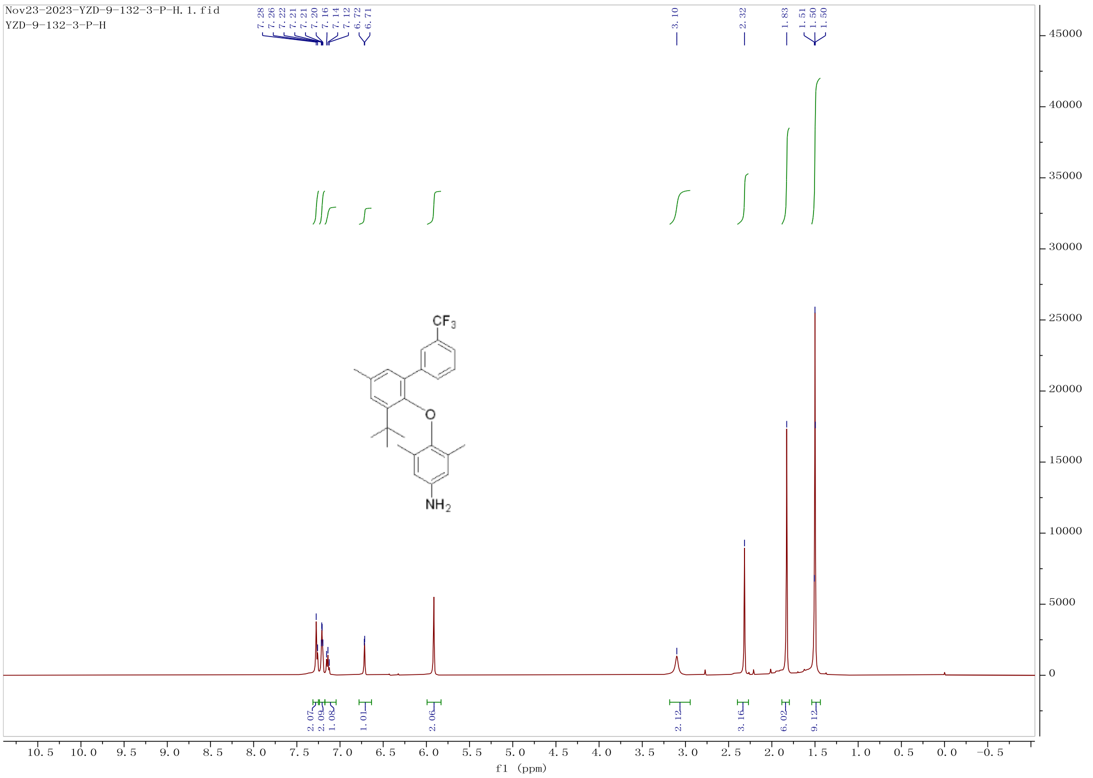

1p: ^{13}C NMR (126 MHz, Chloroform-*d*)

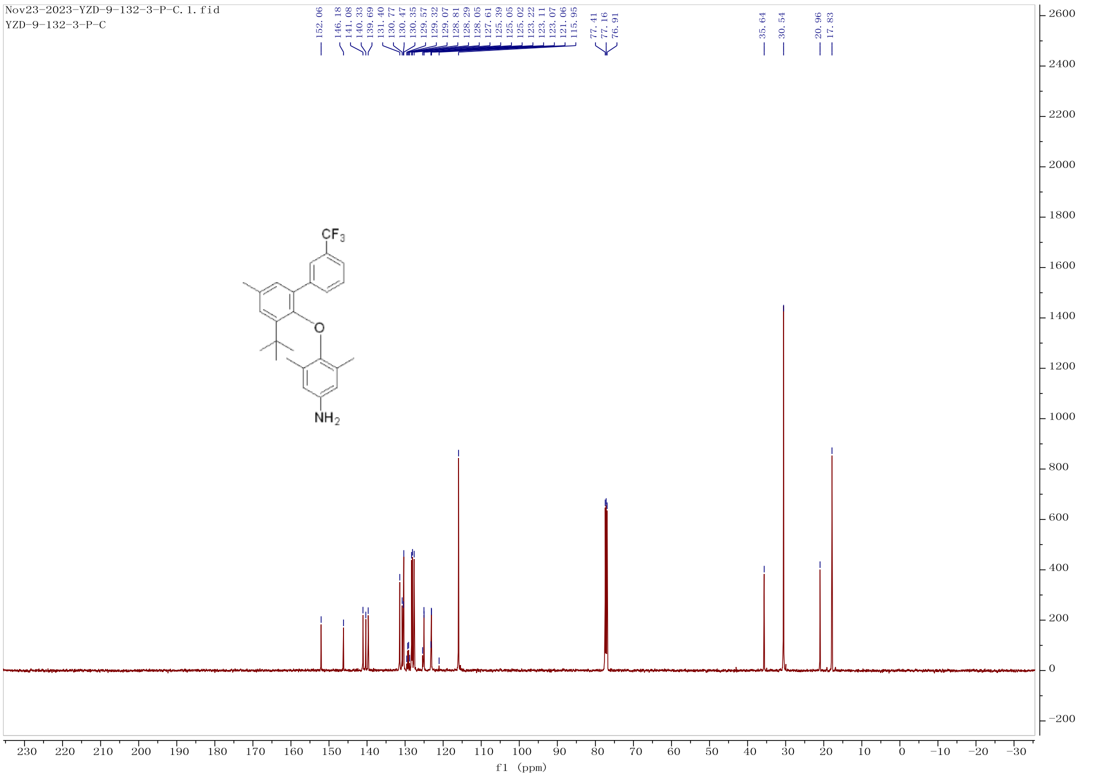

1p: ^{19}F NMR (471 MHz, Chloroform-*d*)

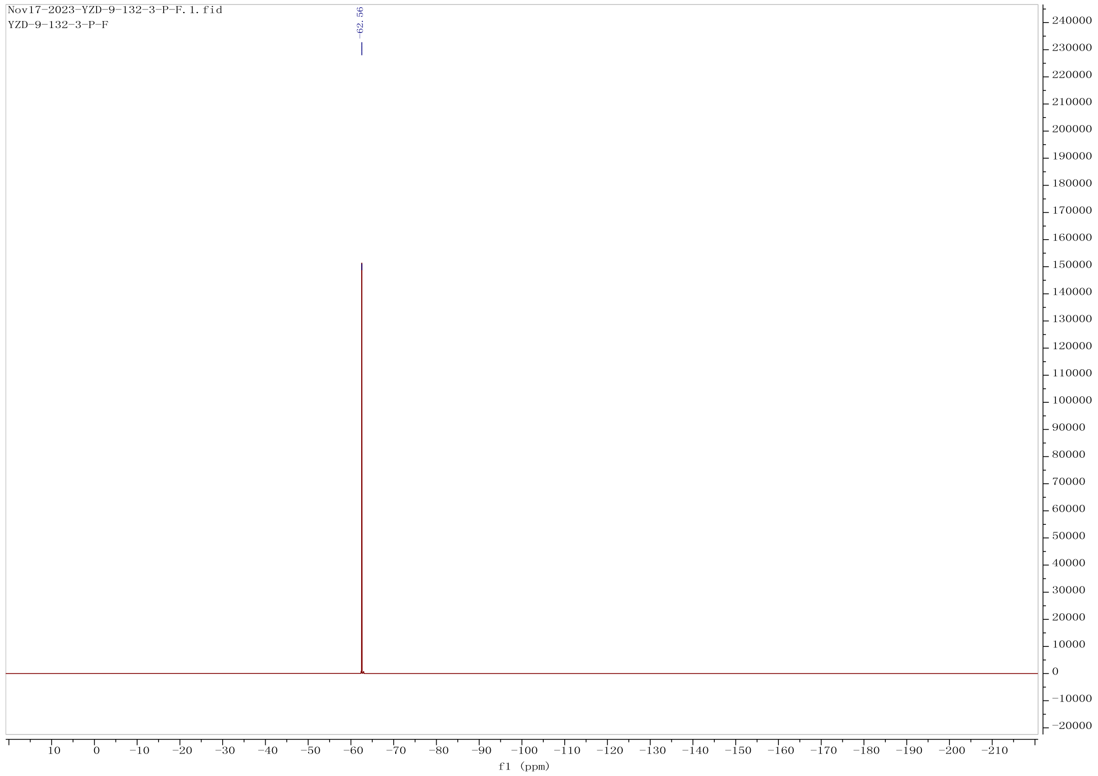

4-(2-(tert-butyl)-4-methyl-6-(naphthalen-1-yl)phenoxy)-3,5-dimethylaniline (**1q**)

1q: ^{1}H NMR (500 MHz, Chloroform-*d*)

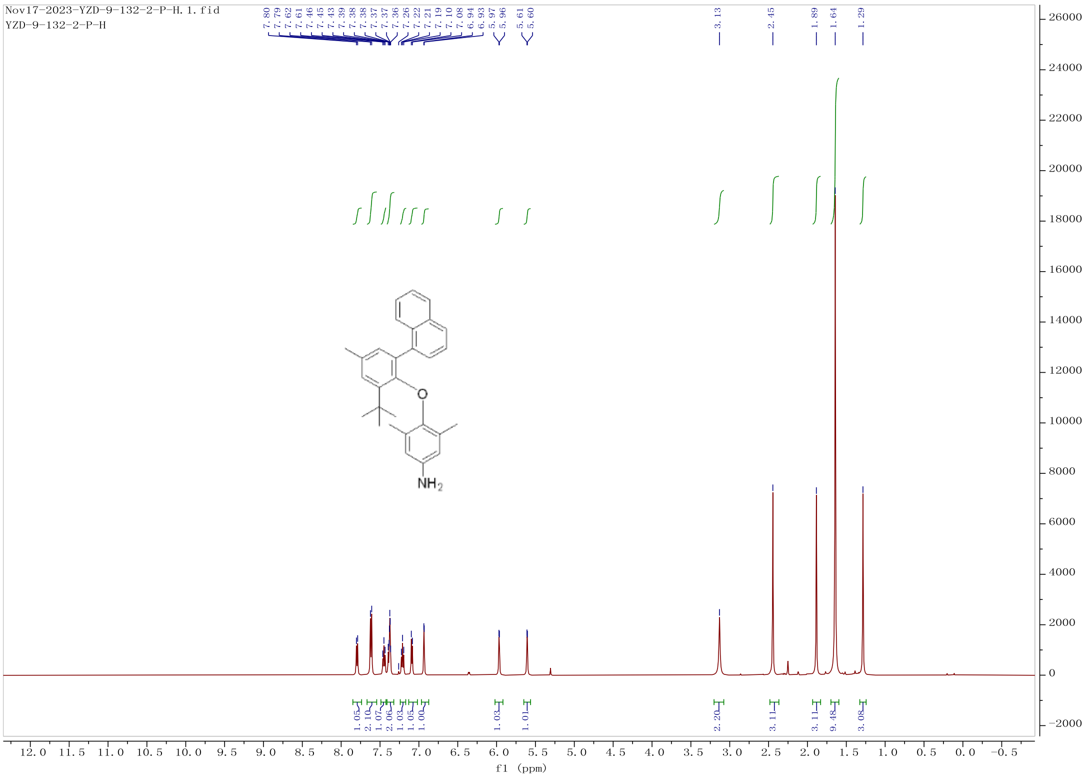

1q: ^{13}C NMR (126 MHz, Chloroform-*d*)

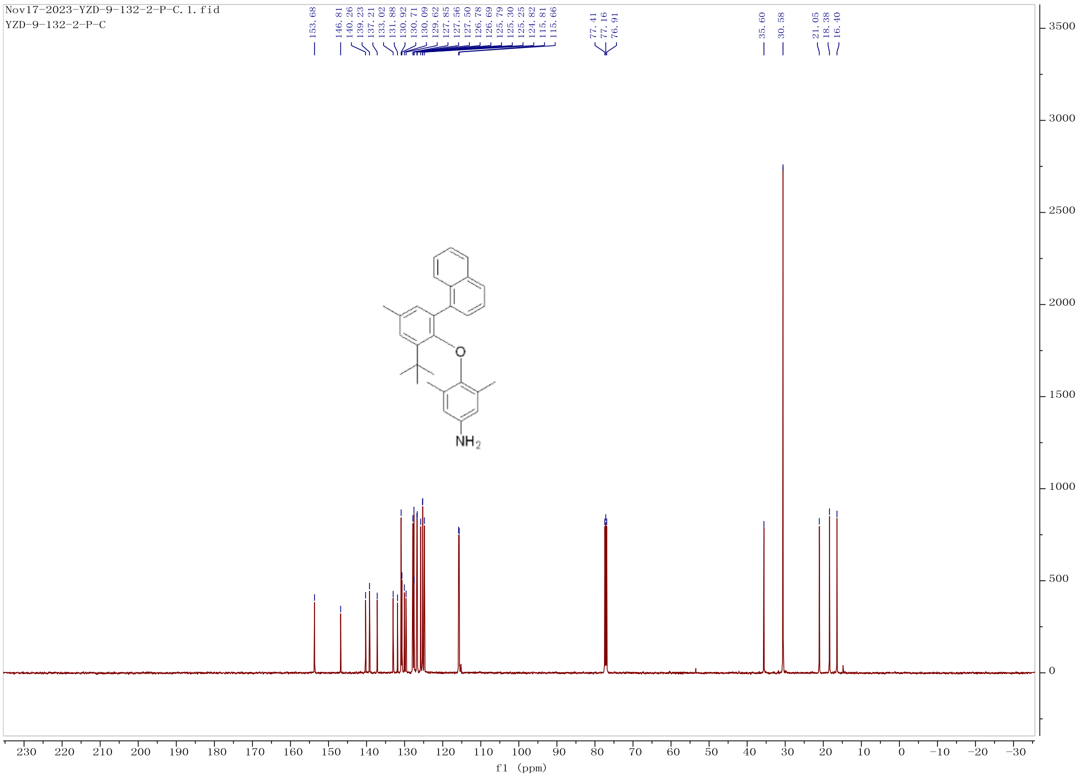

4-((3-(tert-butyl)-5-methyl-2',3',4',5'-tetrahydro-[1,1'-biphenyl]-2-yl)oxy)-3,5-dimethylaniline (**1r**)

1r: ^{1}H NMR (500 MHz, Chloroform-*d*)

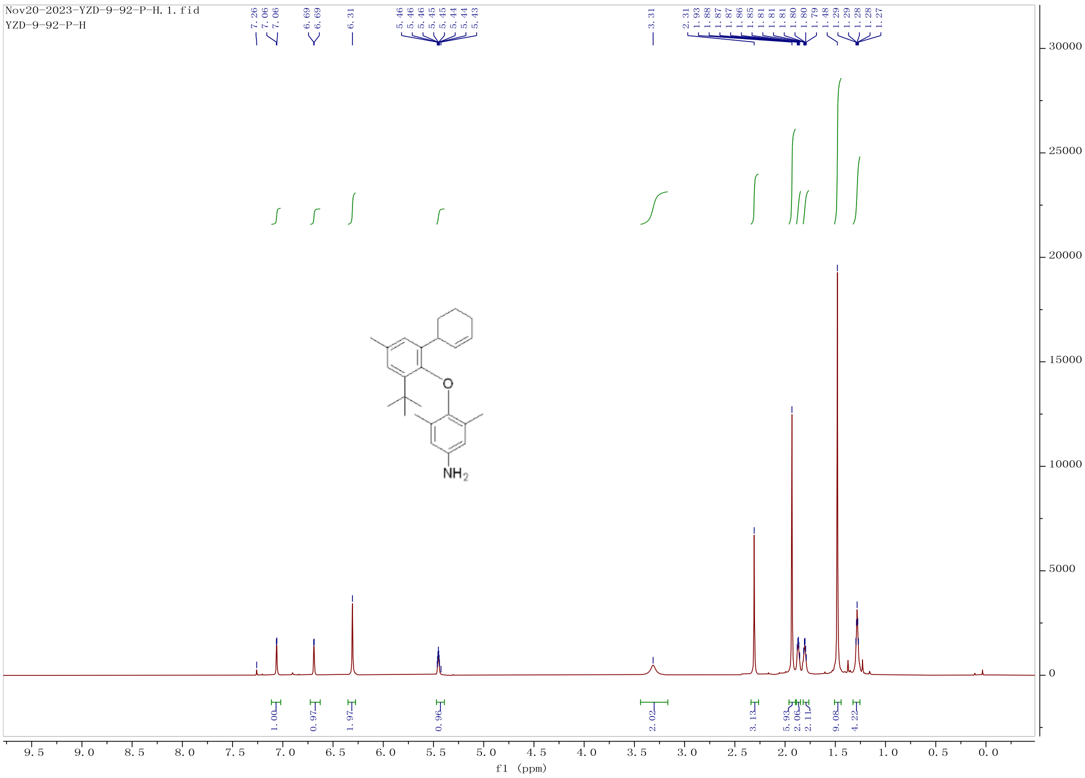

1r: ^{13}C NMR (126 MHz, Chloroform-*d*)

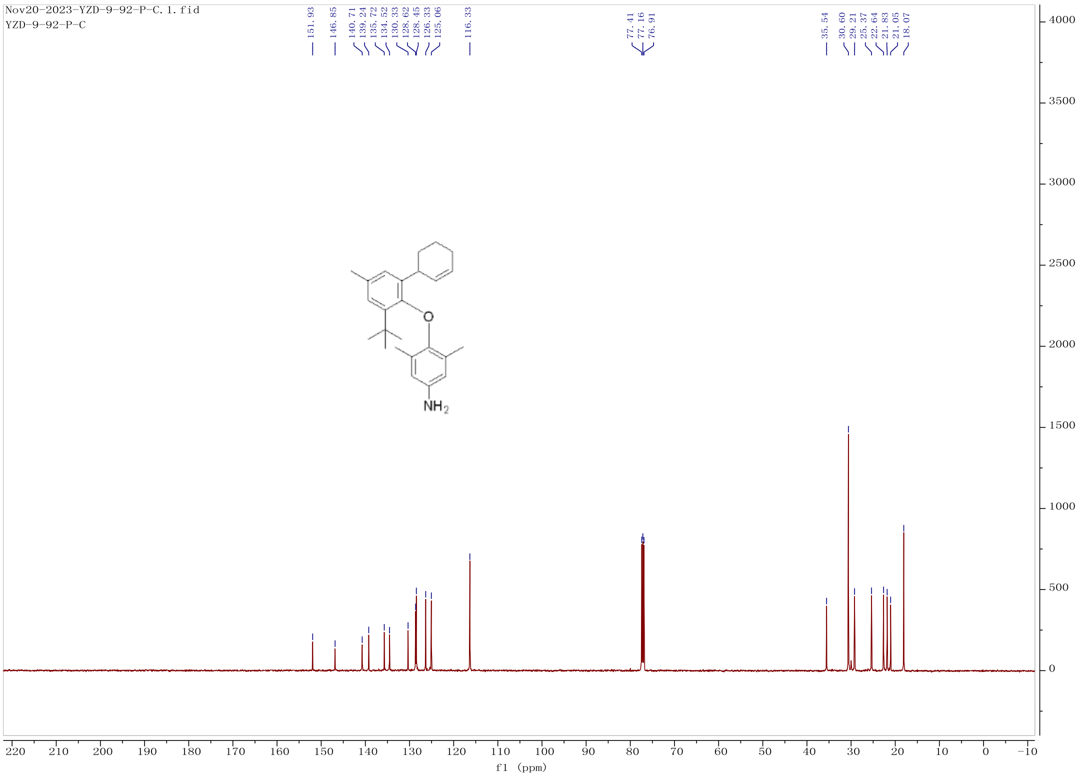

4-(2-(tert-butyl)-6-methylphenoxy)-3,5-dimethylaniline (**1s**)

1s: ^{1}H NMR (500 MHz, Chloroform-*d*)

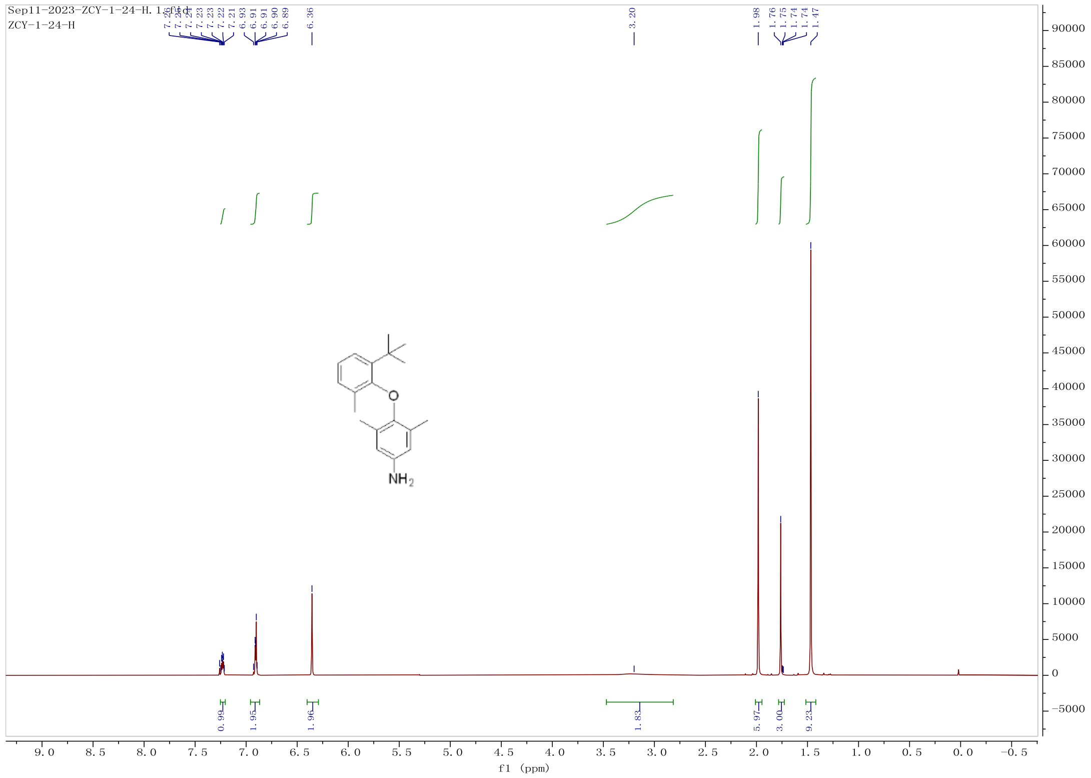

1s: ^{13}C NMR (126 MHz, Chloroform-*d*)

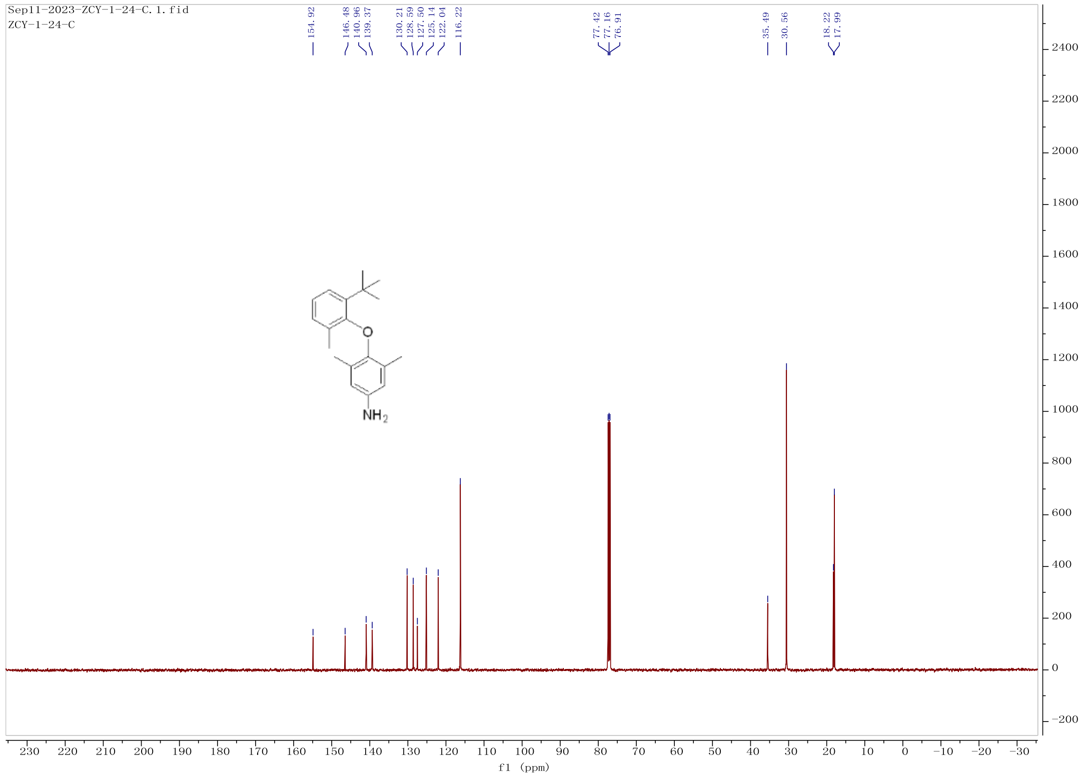

4-((5'-(tert-butyl)-[1,1':3',1''-terphenyl]-4'-yl)oxy)-3,5-dimethylaniline (**1t**)

1t: ^{1}H NMR (500 MHz, Chloroform-*d*)

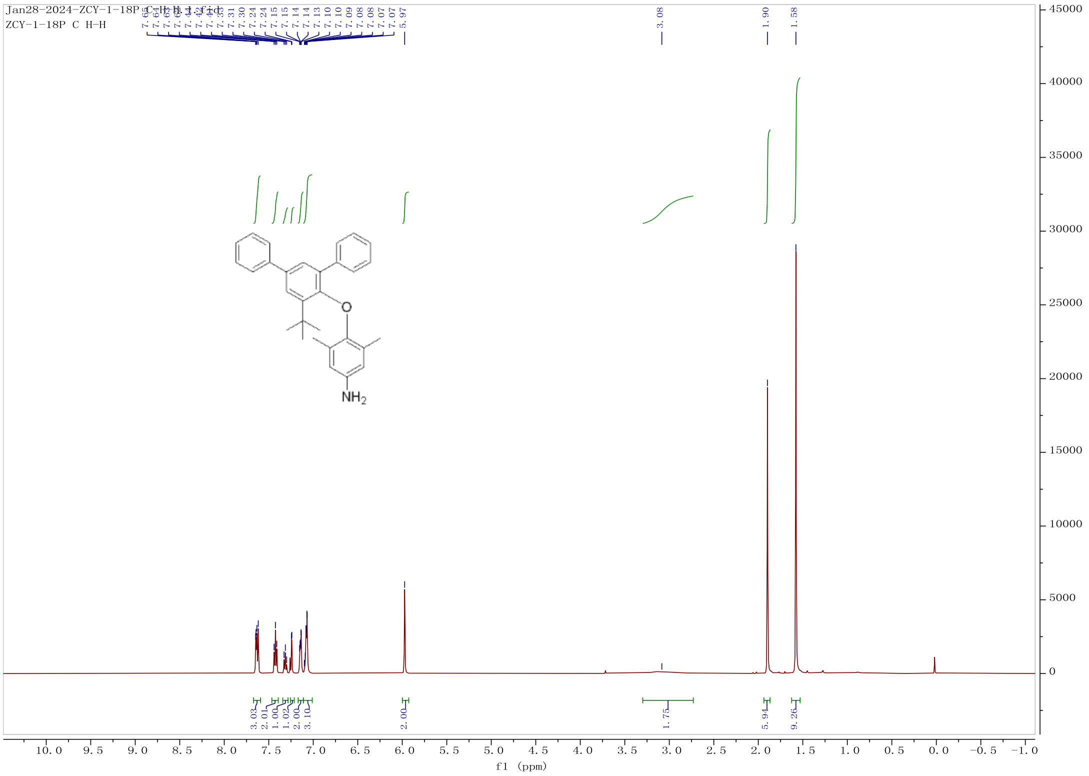

1t: ^{13}C NMR (126 MHz, Chloroform-*d*)

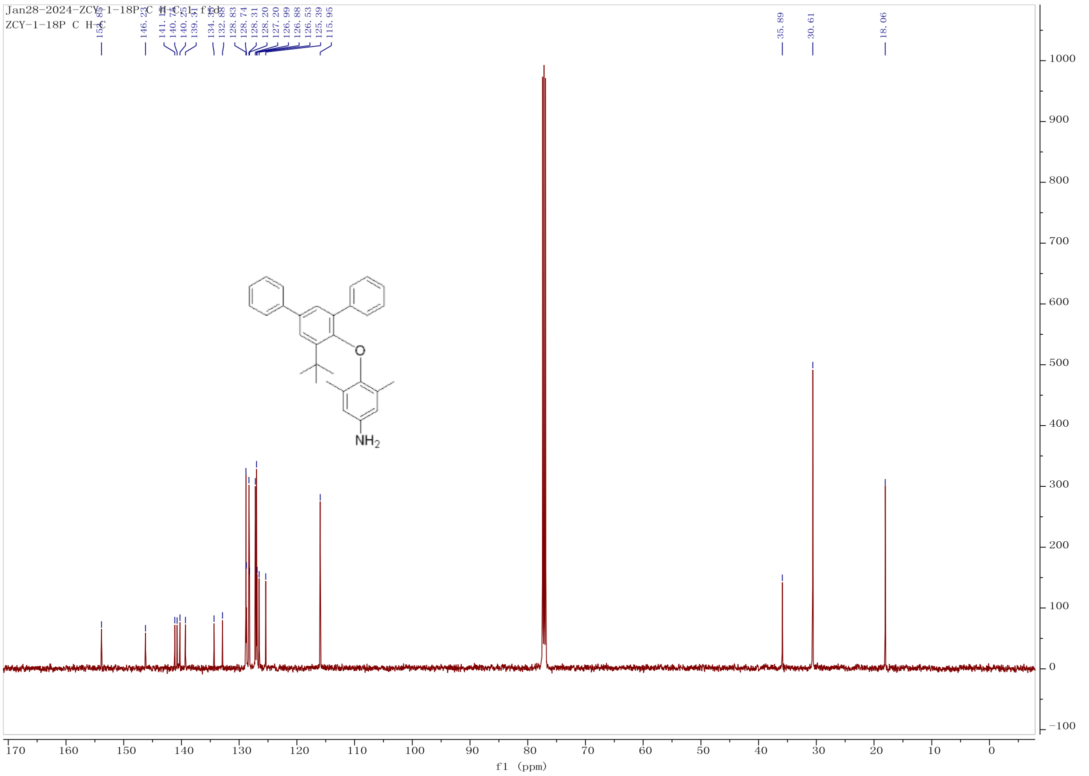

4-((5'-(tert-butyl)-4,4"-dimethoxy-[1,1':3',1"-terphenyl]-4'-yl)oxy)-3,5-dimethylaniline (**1u**)

1u: ^{1}H NMR (500 MHz, Chloroform-*d*)

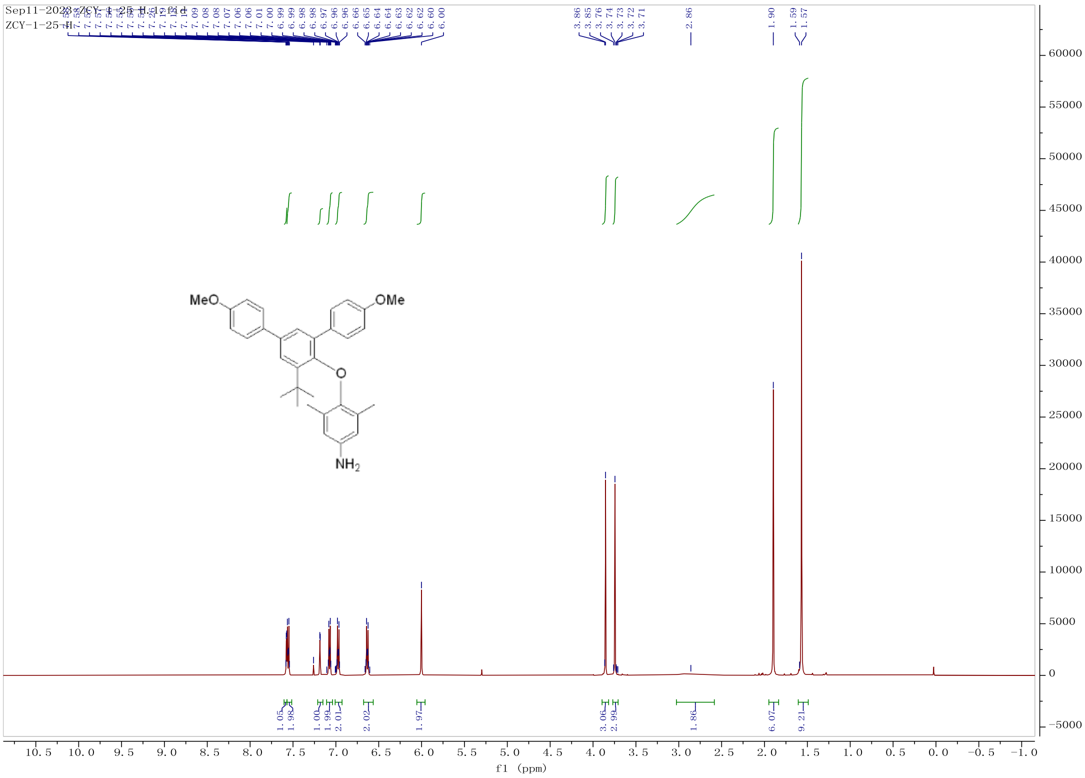

1u: ^{13}C NMR (126 MHz, Chloroform-*d*)

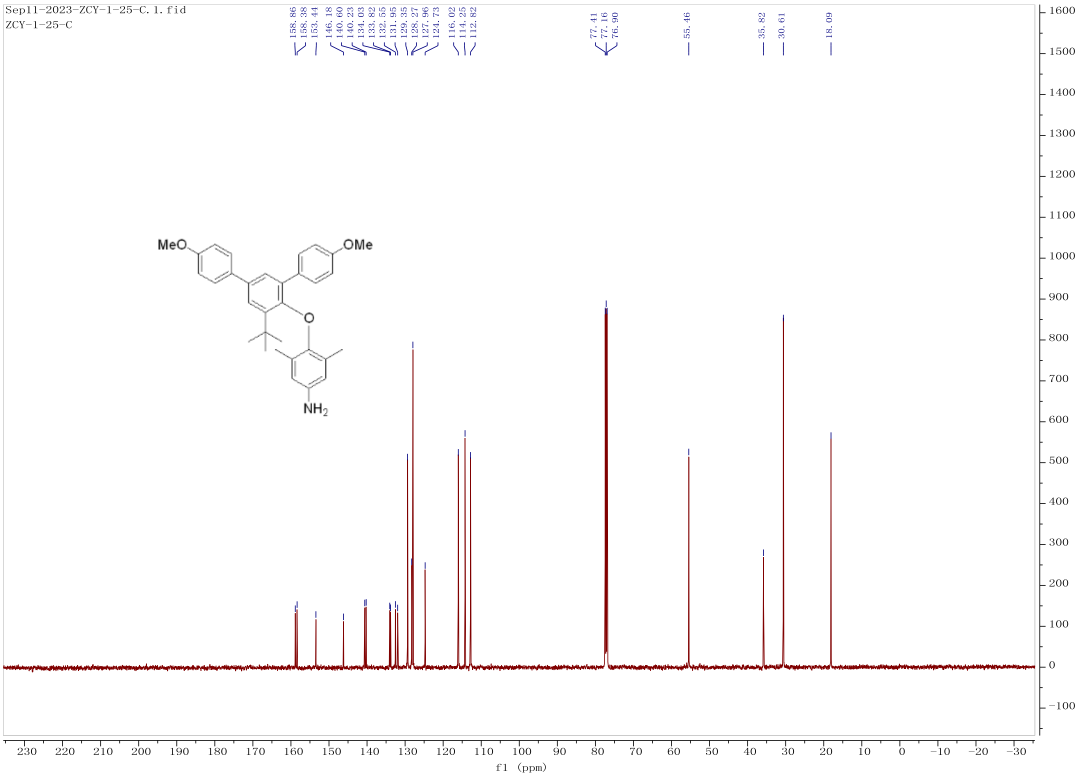

4-(2-(tert-butyl)-4,6-dimethylphenoxy)-3,5-diethylaniline (**1v**)

1v: ^{1}H NMR (500 MHz, Chloroform-*d*)

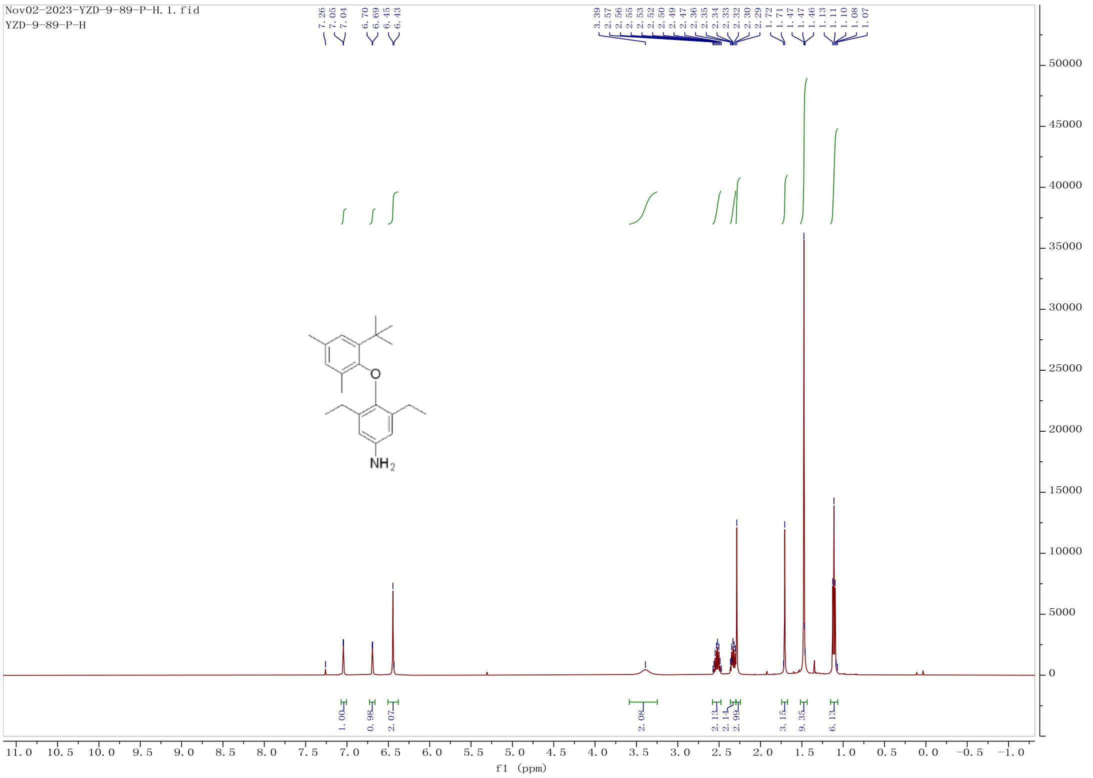

1v: ^{13}C NMR (126 MHz, Chloroform-*d*)

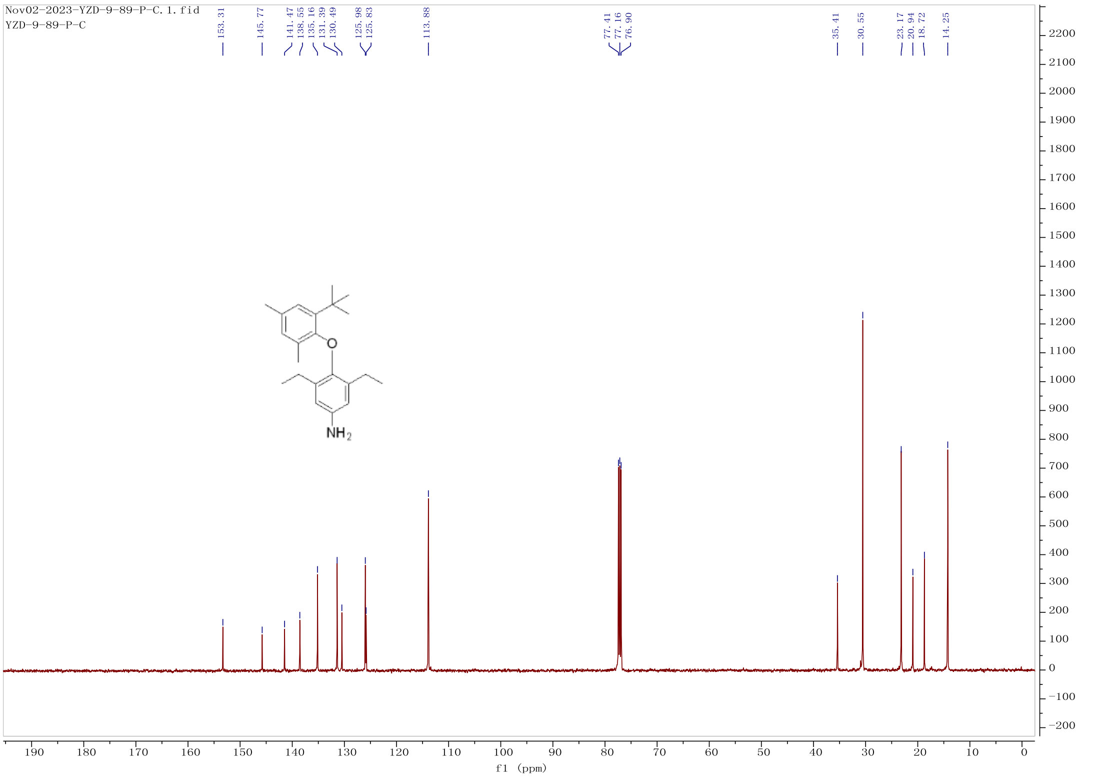

2'-(2-(tert-butyl)-4,6-dimethylphenoxy)-[1,1':3',1''-terphenyl]-5'-amine (**1w**)

1w: ^{1}H NMR (500 MHz, Chloroform-*d*)

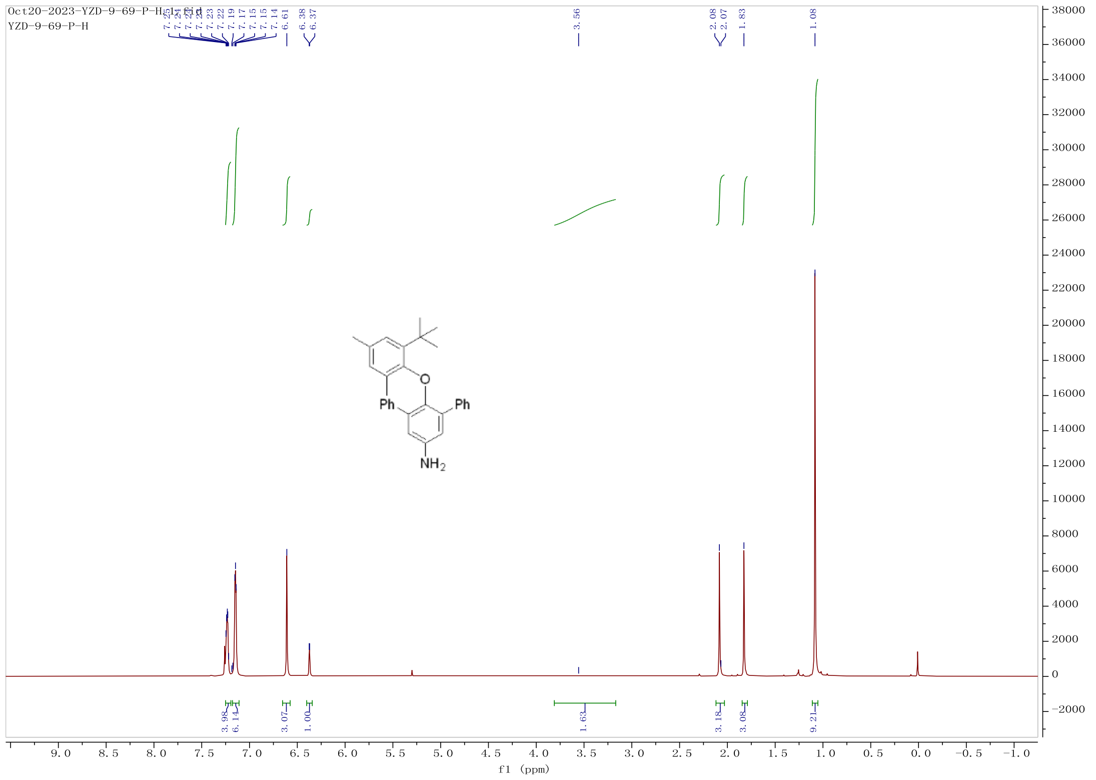

1w: ^{13}C NMR (126 MHz, Chloroform-*d*)

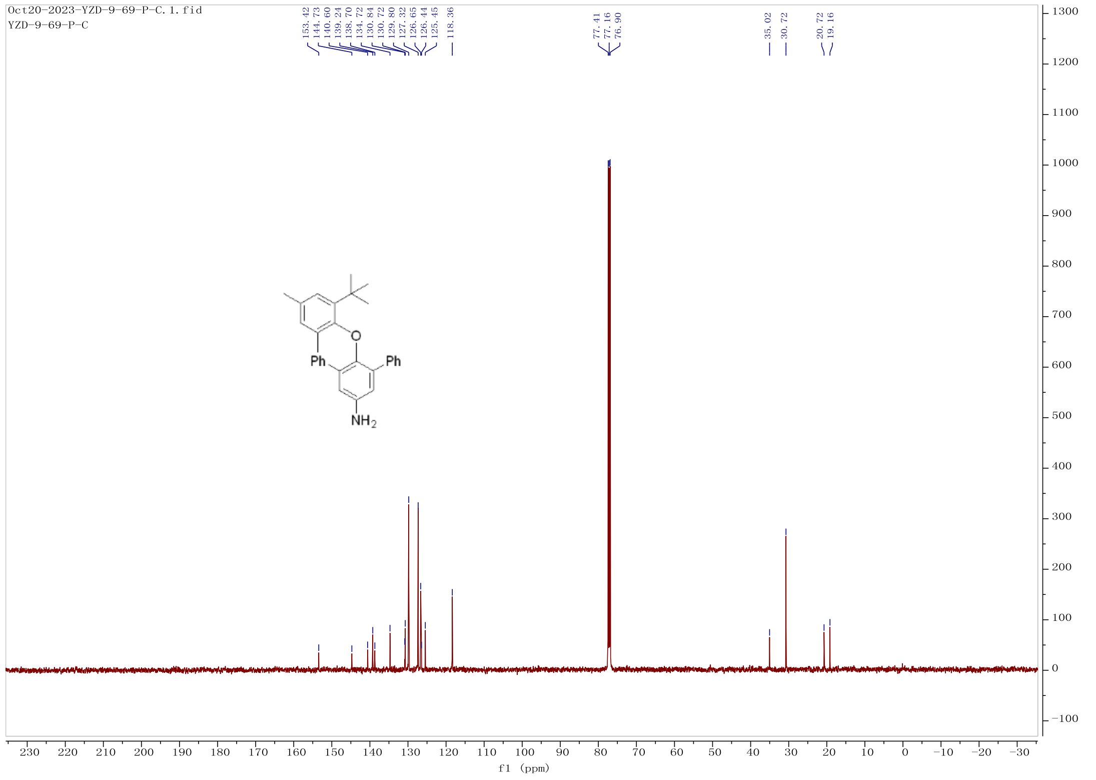

2'-(2-(tert-butyl)-4,6-dimethylphenoxy)-4,4"-dimethoxy-[1,1':3',1"-terphenyl]-5'-amine (**1x**)

1x: ^{1}H NMR (500 MHz, Chloroform-*d*)

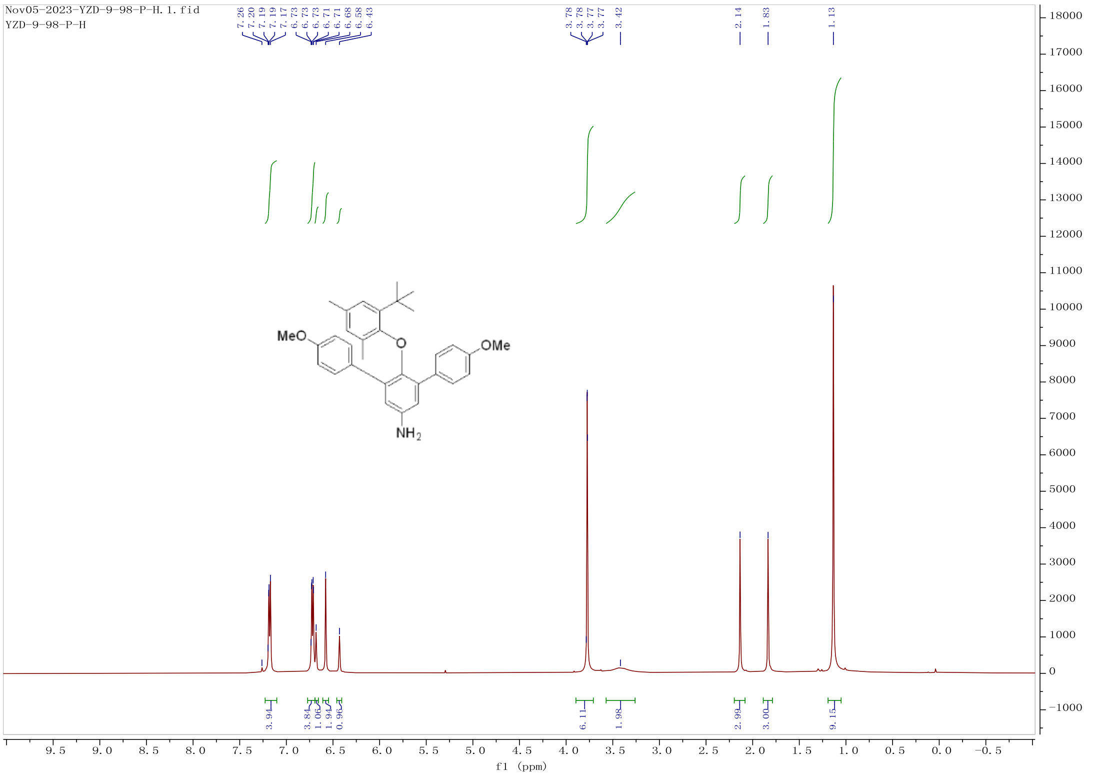

1x: ^{13}C NMR (126 MHz, Chloroform-*d*)

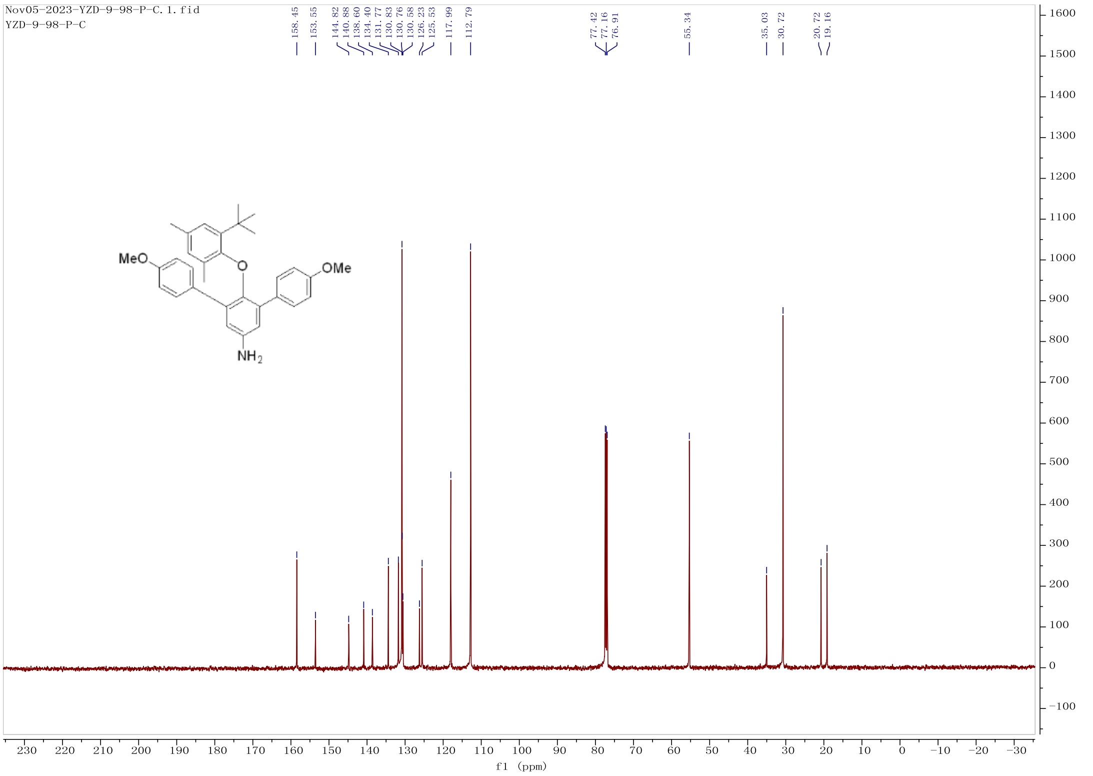

2'-(2-(tert-butyl)-4,6-dimethylphenoxy)-2,2''-dimethoxy-[1,1':3',1''-terphenyl]-5'-amine (**1y**)

1y: ^{1}H NMR (500 MHz, Chloroform-*d*)

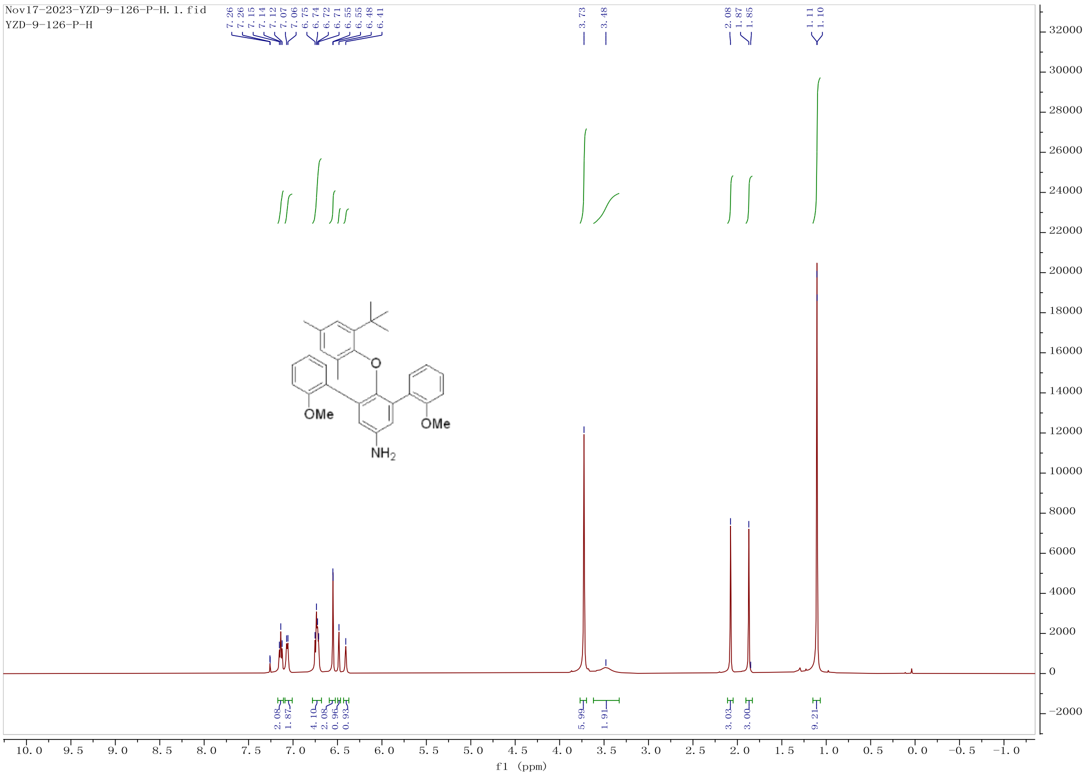

1y: ^{13}C NMR (126 MHz, Chloroform-*d*)

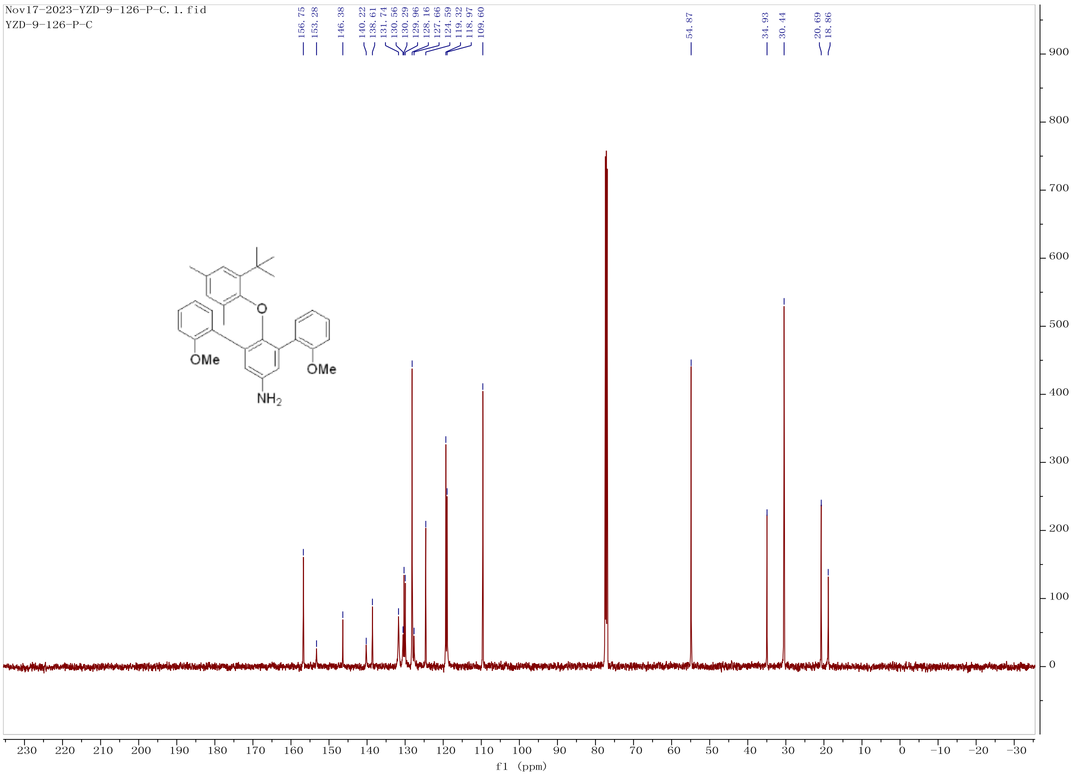

2'-(2-(tert-butyl)-4,6-dimethylphenoxy)-2,2"-difluoro-[1,1':3',1"-terphenyl]-5'-amine (**1z**)

1z: ^{1}H NMR (500 MHz, Chloroform-*d*)

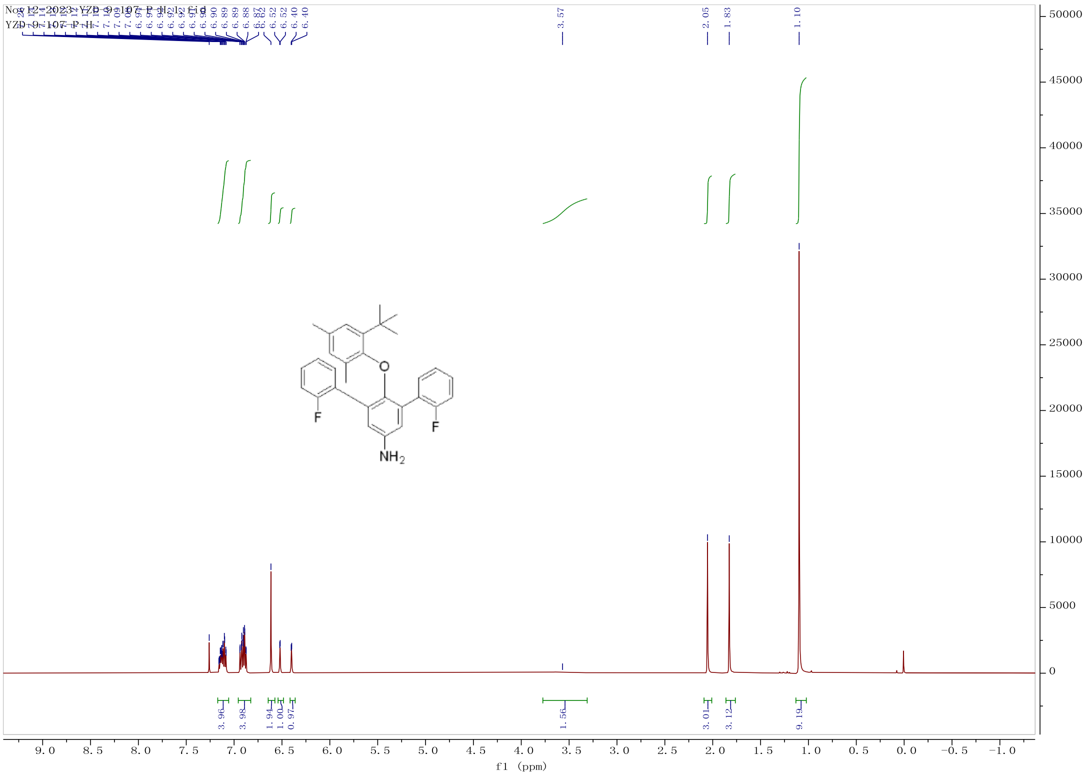

1z: ^{13}C NMR (126 MHz, Chloroform-*d*)

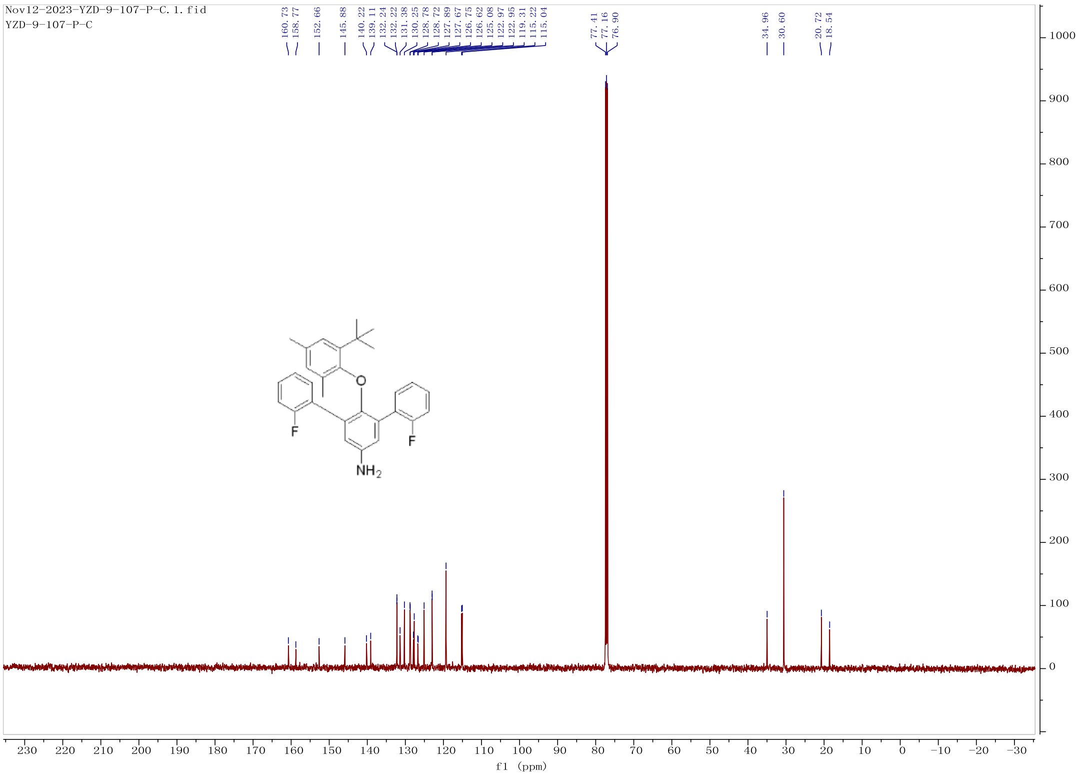

1z: ^{19}F NMR (471 MHz, Chloroform-*d*)

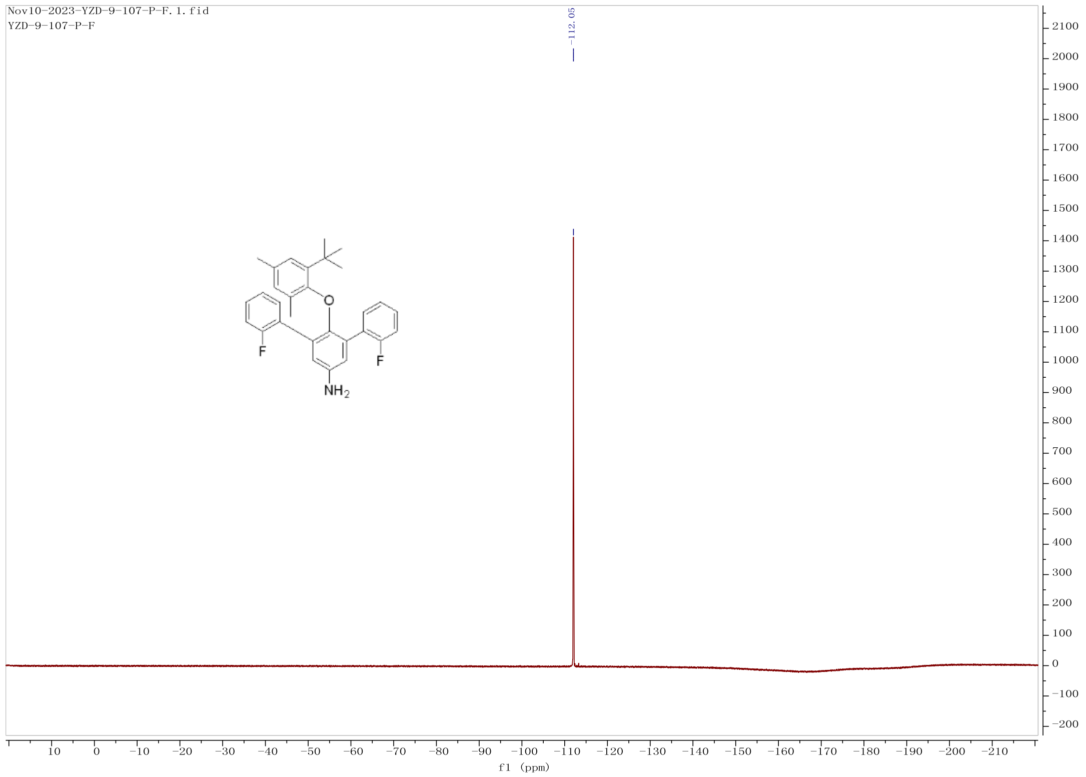

4-(2-(tert-butyl)-4,6-dimethylphenoxy)-3,5-di((E)-styryl)aniline (**1aa**)

1aa: ^{1}H NMR (500 MHz, Chloroform-*d*)

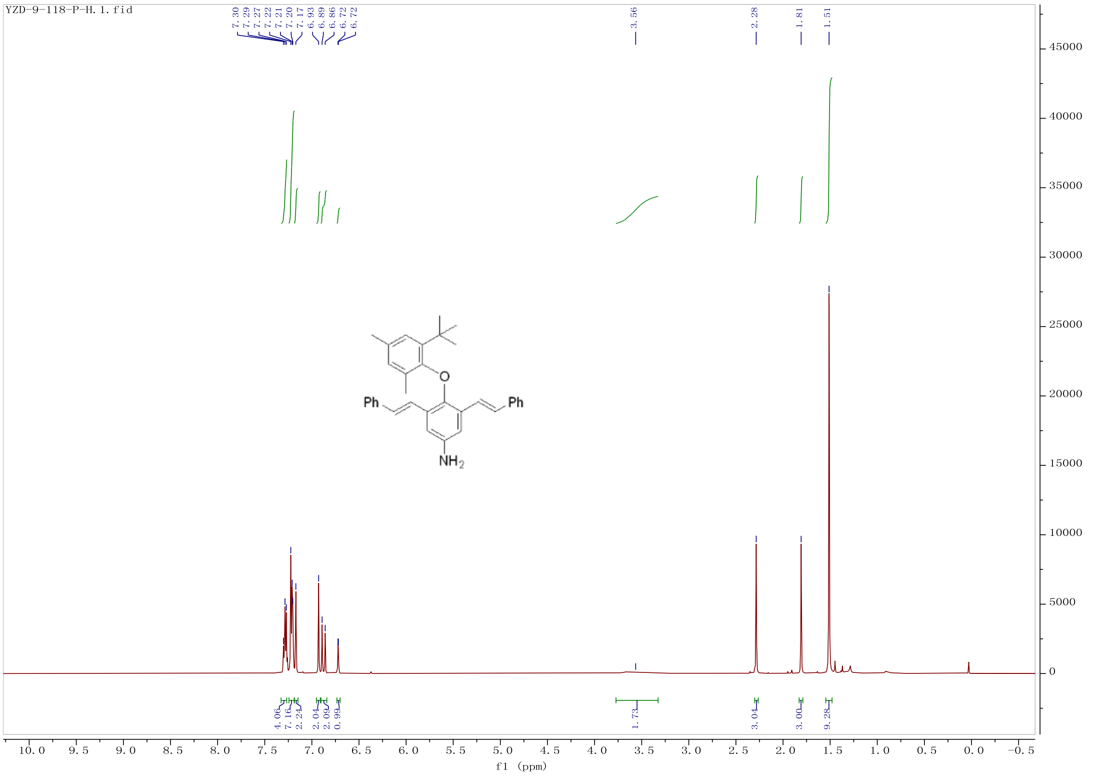

1aa: ^{13}C NMR (126 MHz, Chloroform-*d*)

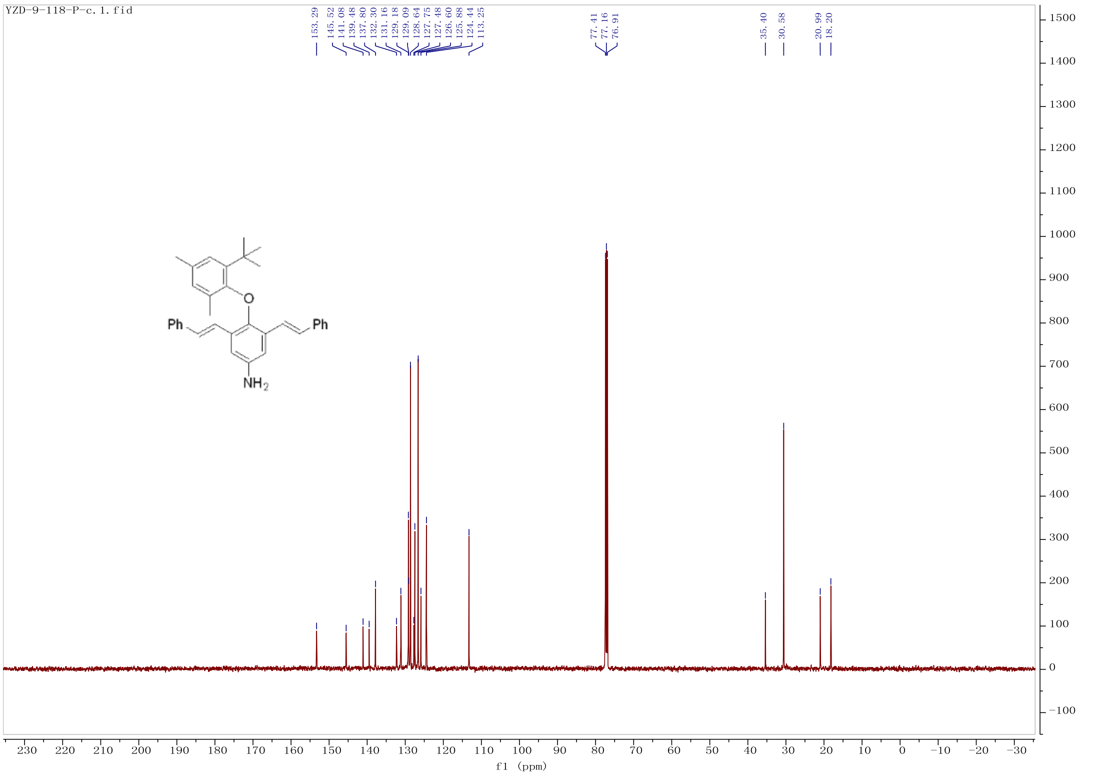

3,5-dibromo-4-(2-(tert-butyl)-4,6-dimethylphenoxy)aniline (**1ab**)

1ab: ^{1}H NMR (400 MHz, Chloroform-*d*)

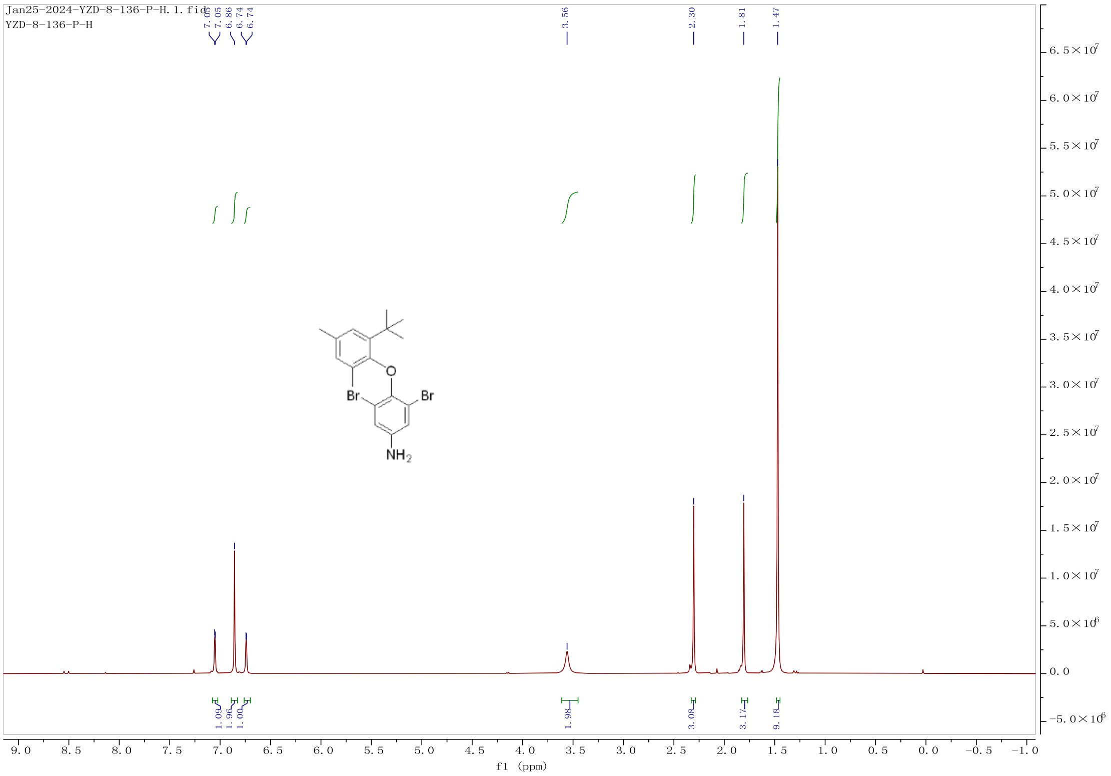

1ab: ^{13}C NMR (101 MHz, Chloroform-*d*)

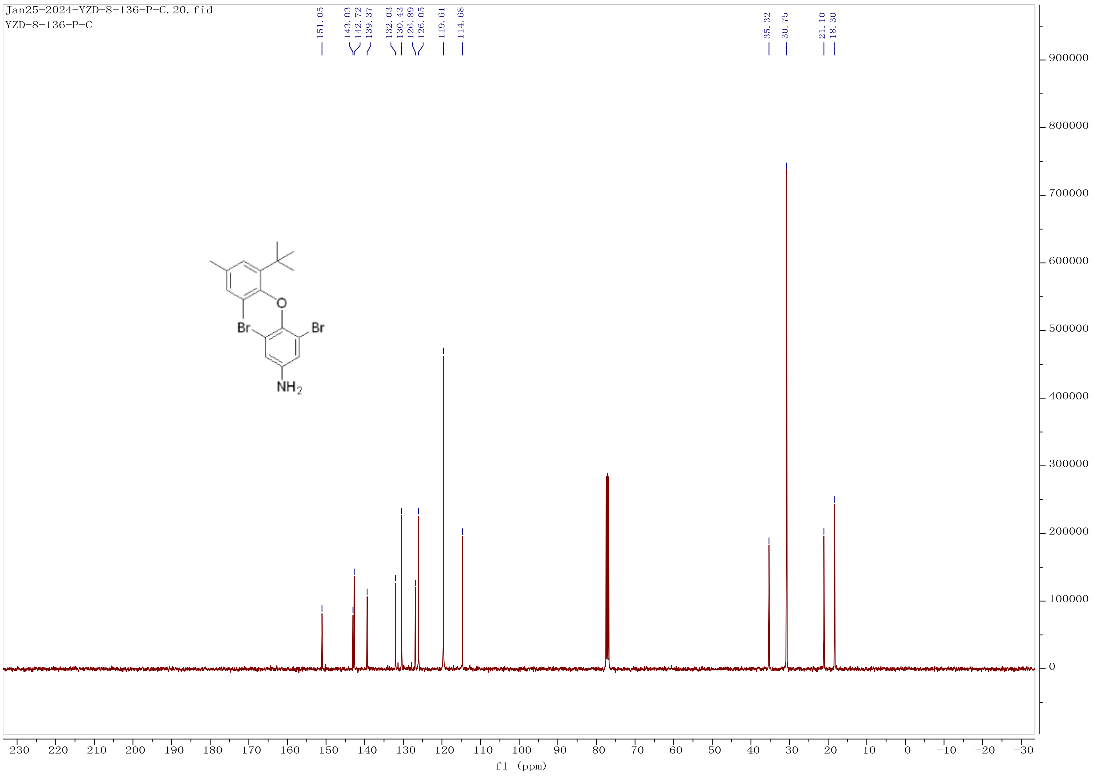

2'-(2-cyclohexyl-4,6-dimethylphenoxy)-[1,1':3',1''-terphenyl]-5'-amine (**1ac**)

1ac: ^{1}H NMR (500 MHz, Chloroform-*d*)

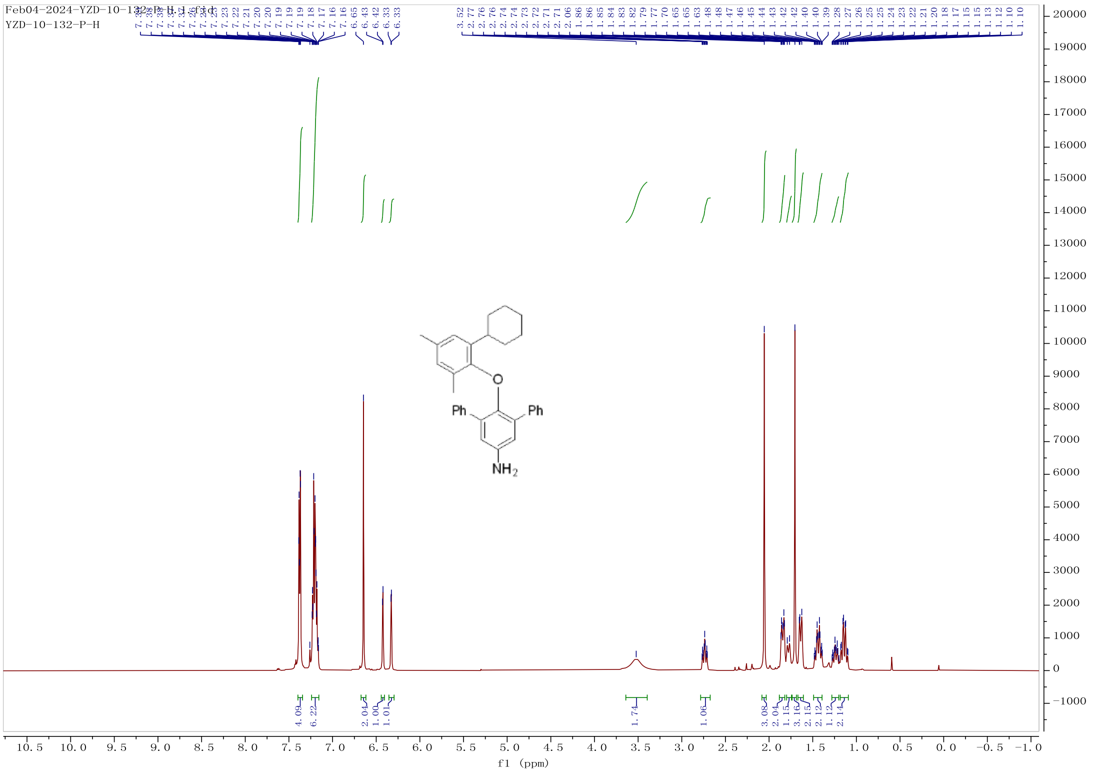

1ac: ^{13}C NMR (126 MHz, Chloroform-*d*)

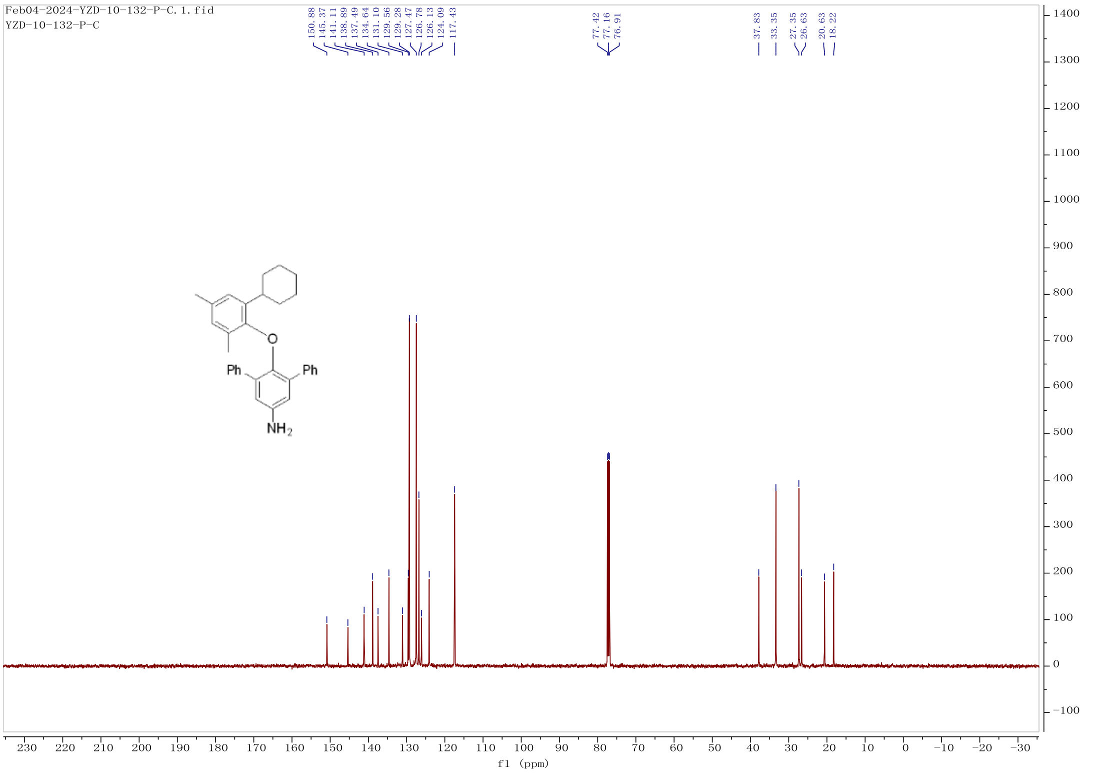

benzyl-((2S,4S)-6-(2-(tert-butyl)-4,6-dimethylphenoxy)-5,7-dimethyl-2-phenyl-1,2,3,4-tetrahydroquinolin-4-yl)carbamate (**4a'**)

4a': ^{1}H NMR (500 MHz, Chloroform-*d*)

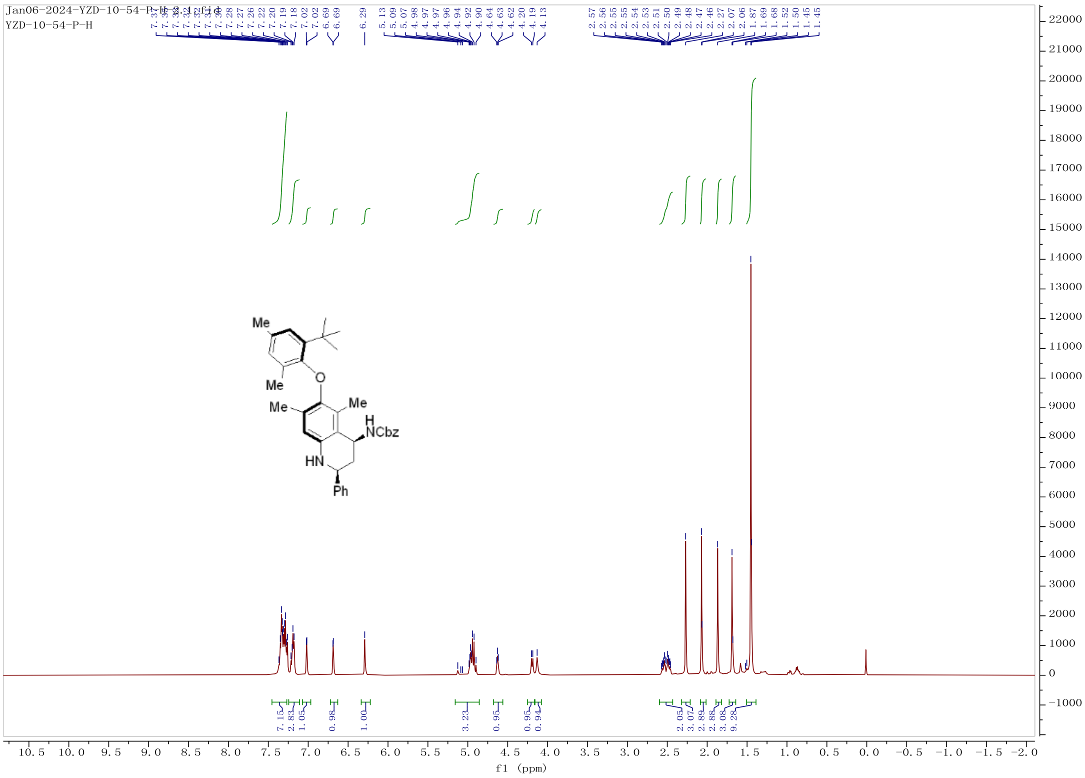

4a': ^{13}C NMR (126 MHz, Chloroform-*d*)

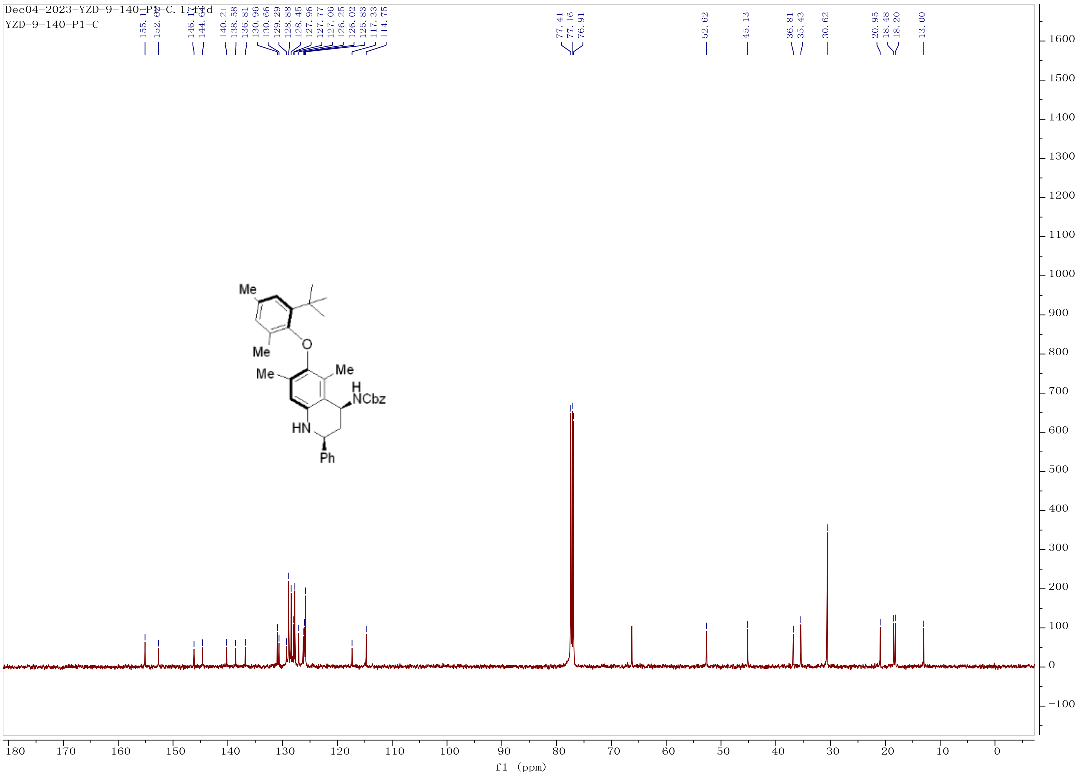

benzyl (6-(2-(tert-butyl)-4,6-dimethylphenoxy)-5,7-dimethyl-2-phenylquinolin-4-yl)carbamate (**4a**)

4a: ^{1}H NMR (400 MHz, Chloroform-*d*)

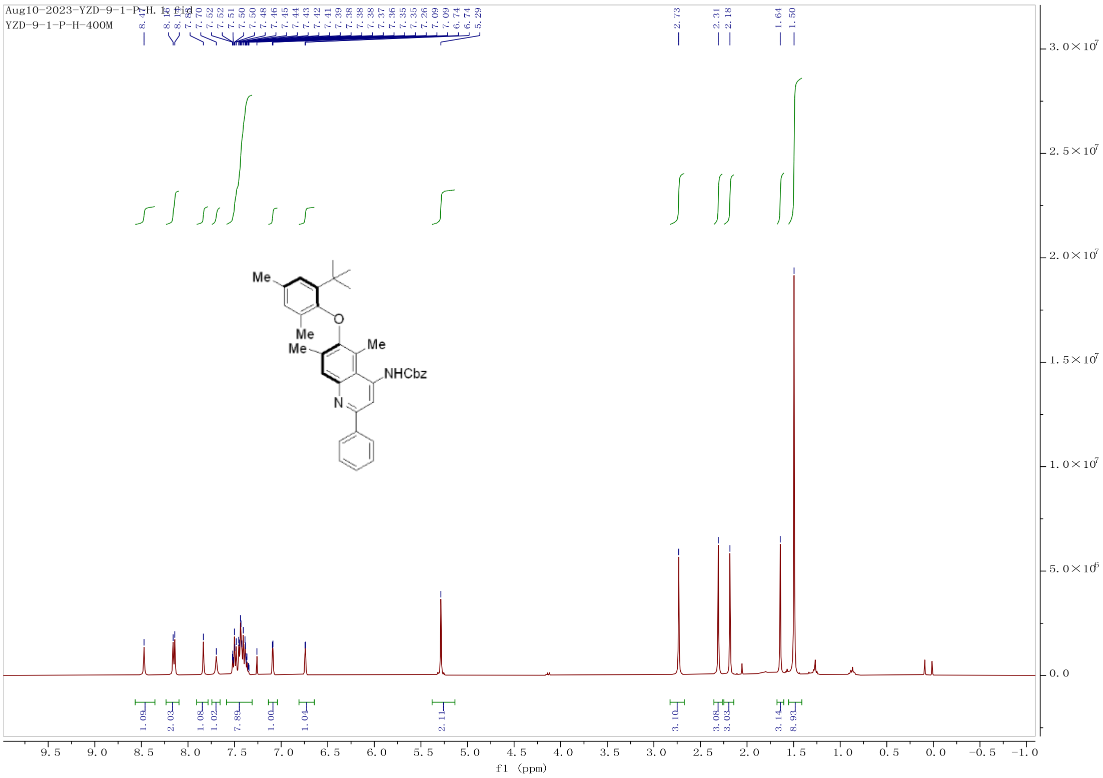

4a: ^{13}C NMR (101 MHz, Chloroform-*d*)

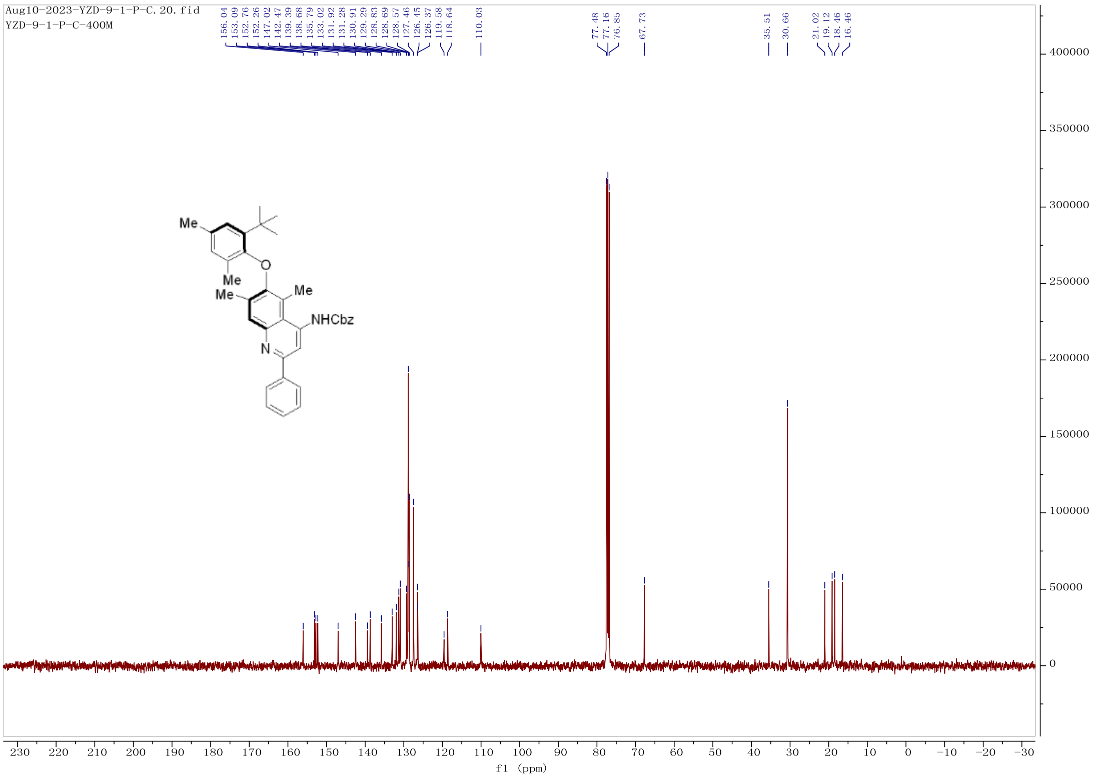

benzyl (6-(2-(tert-butyl)-4,6-dimethylphenoxy)-5,7-dimethyl-2-(4-nitrophenyl)quinolin-4-yl)carbamate (**4b**)

4b: ^{1}H NMR (500 MHz, Chloroform-*d*)

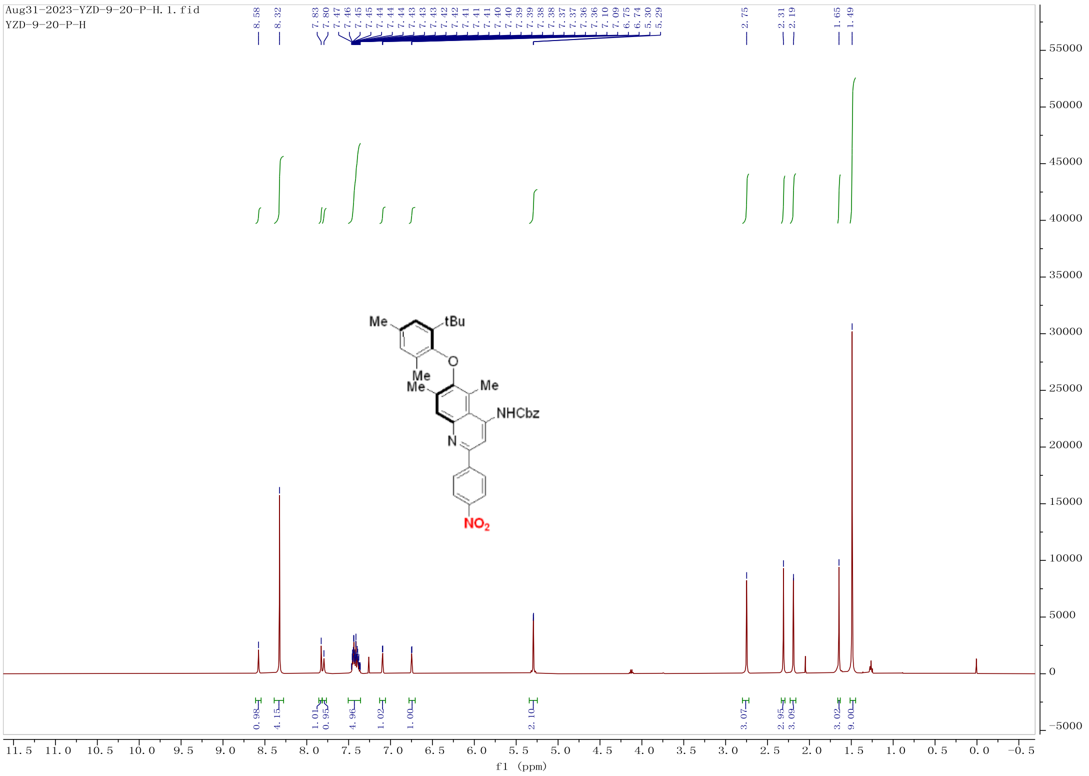

4b: ^{13}C NMR (126 MHz, Chloroform-*d*)

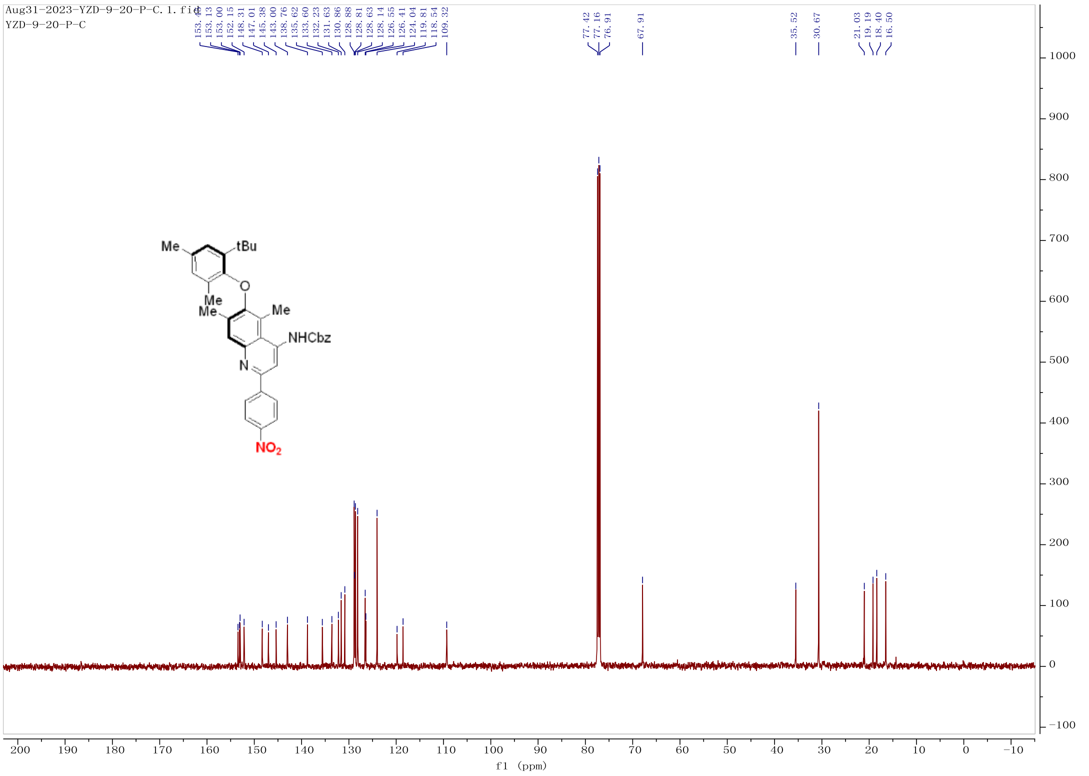

benzyl (6-(2-(tert-butyl)-4,6-dimethylphenoxy)-2-(4-cyanophenyl)-5,7-dimethylquinolin-4-yl)carbamate (**4c**)

4c: ^{1}H NMR (500 MHz, Chloroform-*d*)

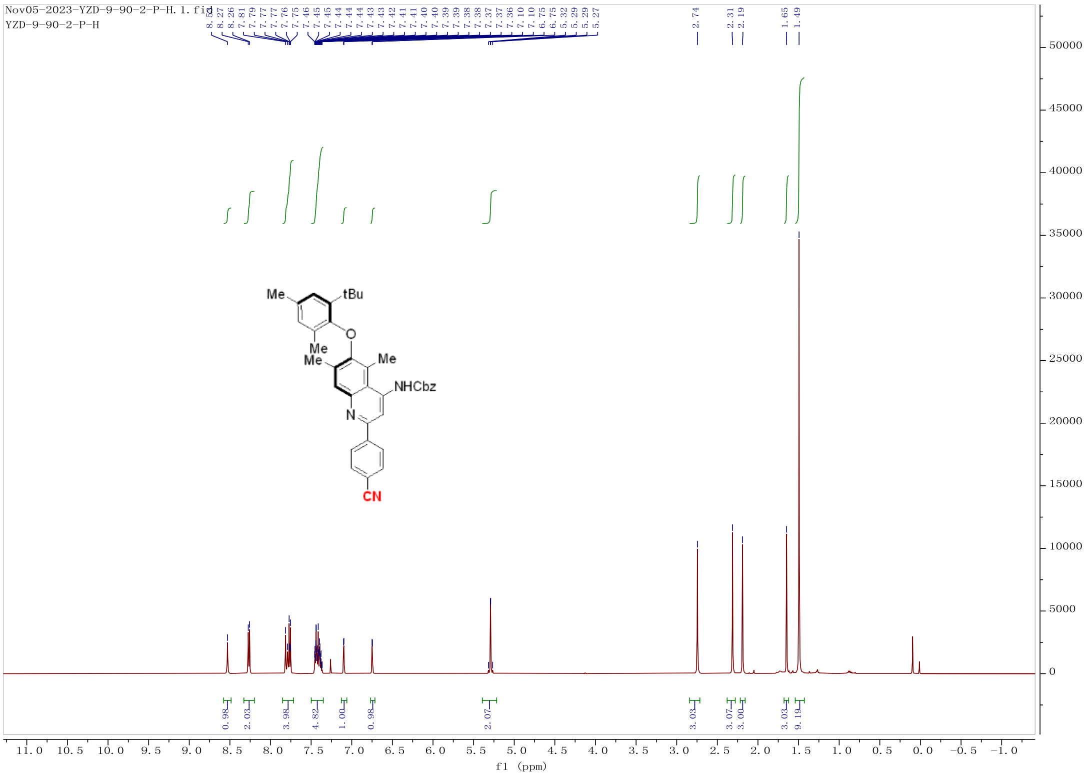

4c: ^{13}C NMR (126 MHz, Chloroform-*d*)

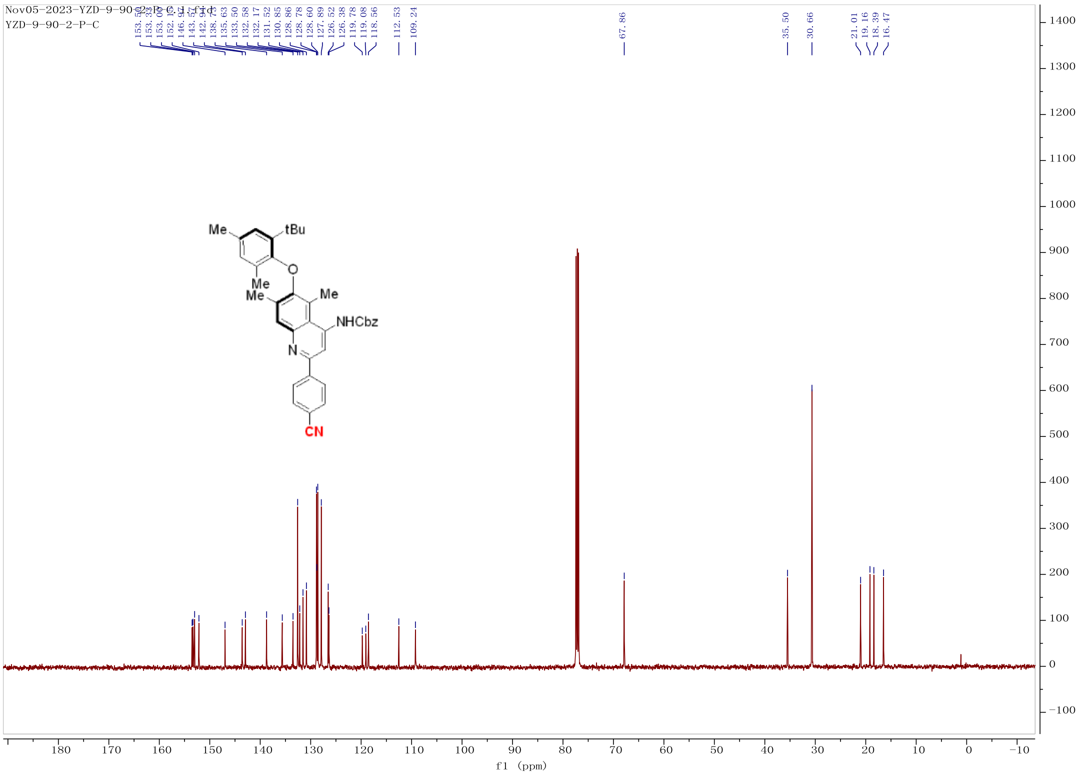

benzyl (6-(2-(tert-butyl)-4,6-dimethylphenoxy)-2-(4-methoxyphenyl)-5,7-dimethylquinolin-4-yl)carbamate (**4d**)

4d: ^{1}H NMR (500 MHz, Chloroform-*d*)

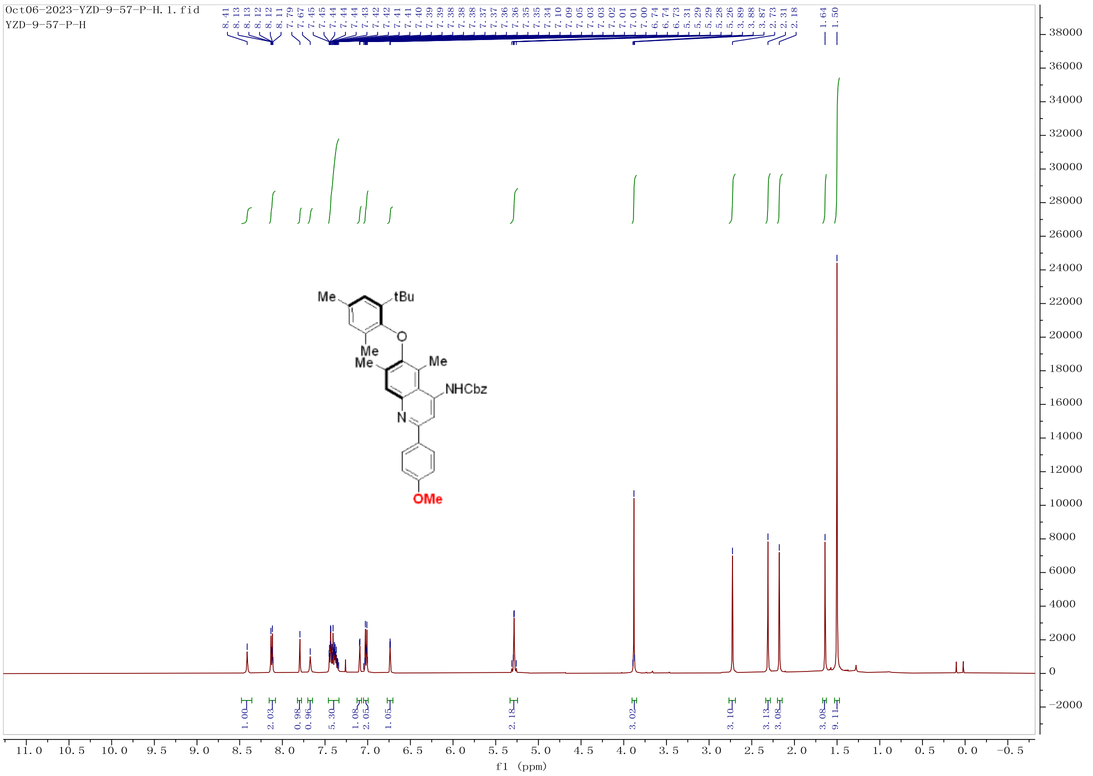

4d: ^{13}C NMR (126 MHz, Chloroform-*d*)

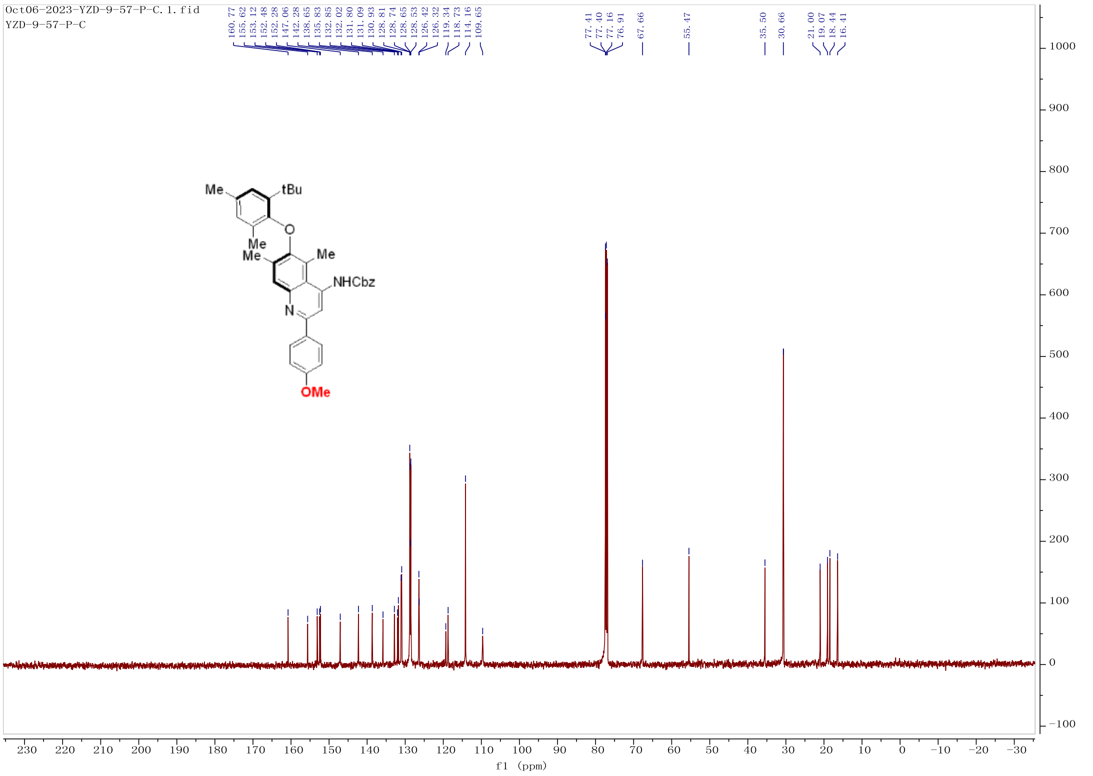

benzyl (6-(2-(tert-butyl)-4,6-dimethylphenoxy)-5,7-dimethyl-2-(3-(trifluoromethyl)phenyl)quinolin-4-yl)carbamate (**4e**)

4e: ^{1}H NMR (500 MHz, Chloroform-*d*)

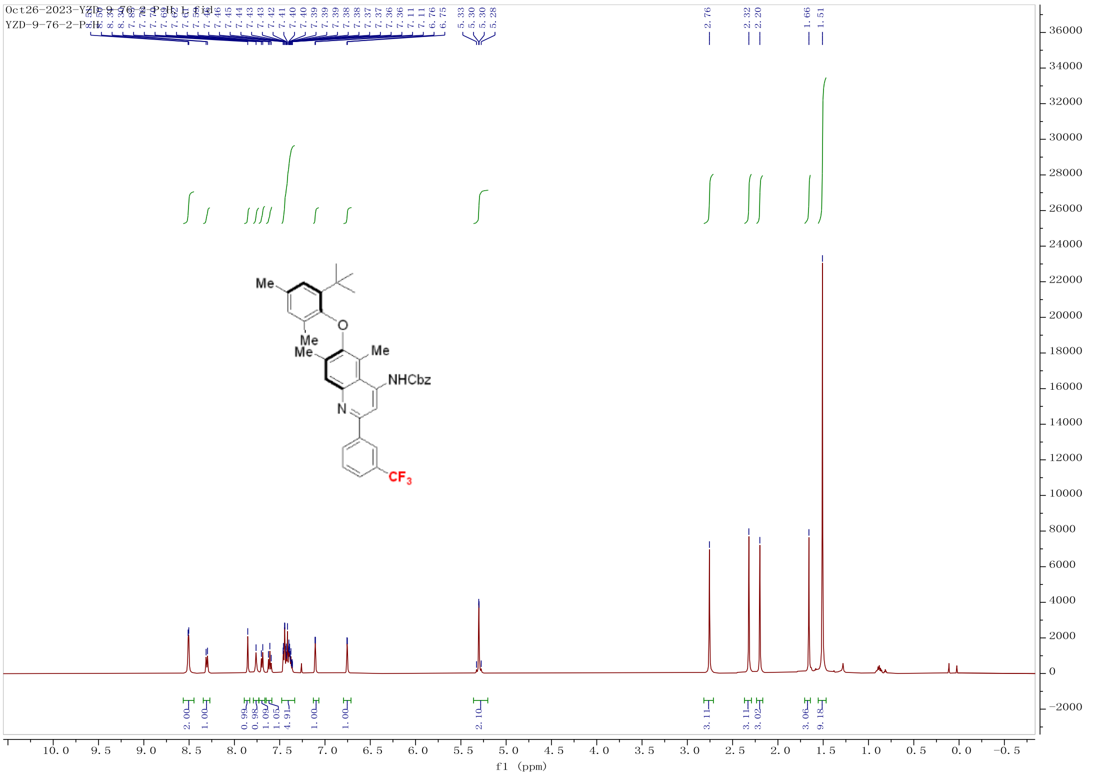

4e: ^{13}C NMR (126 MHz, Chloroform-*d*)

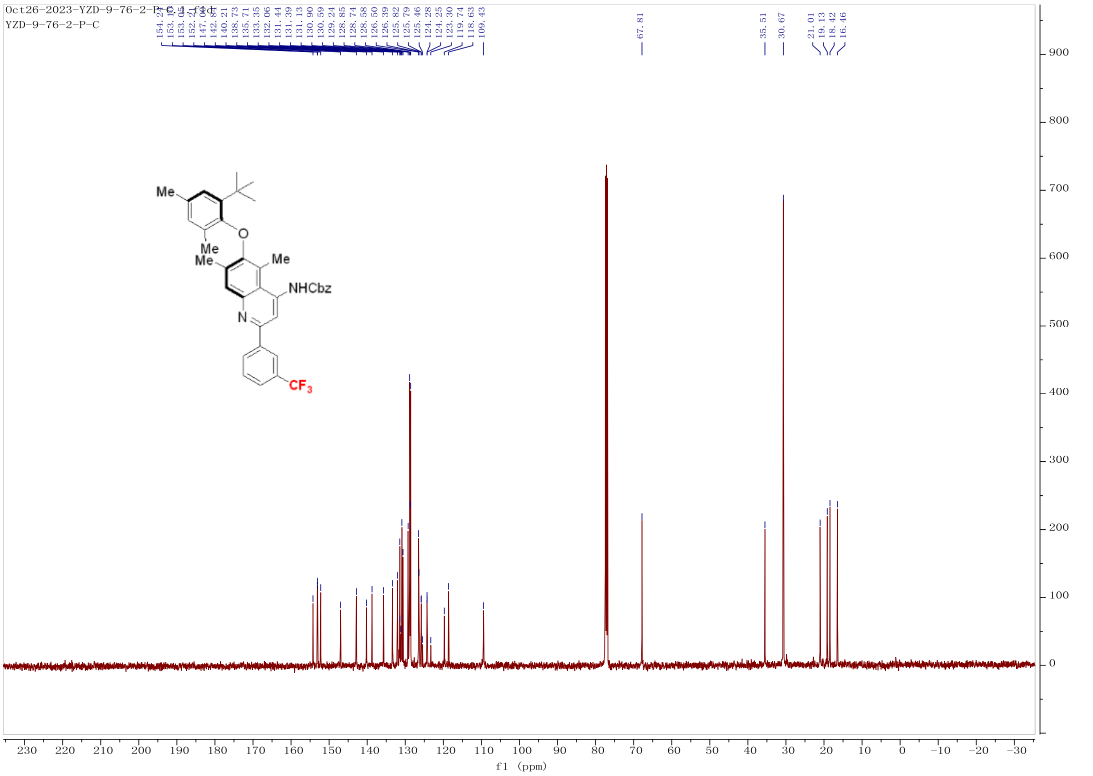

4e: ^{19}F NMR (471 MHz, Chloroform-*d*)

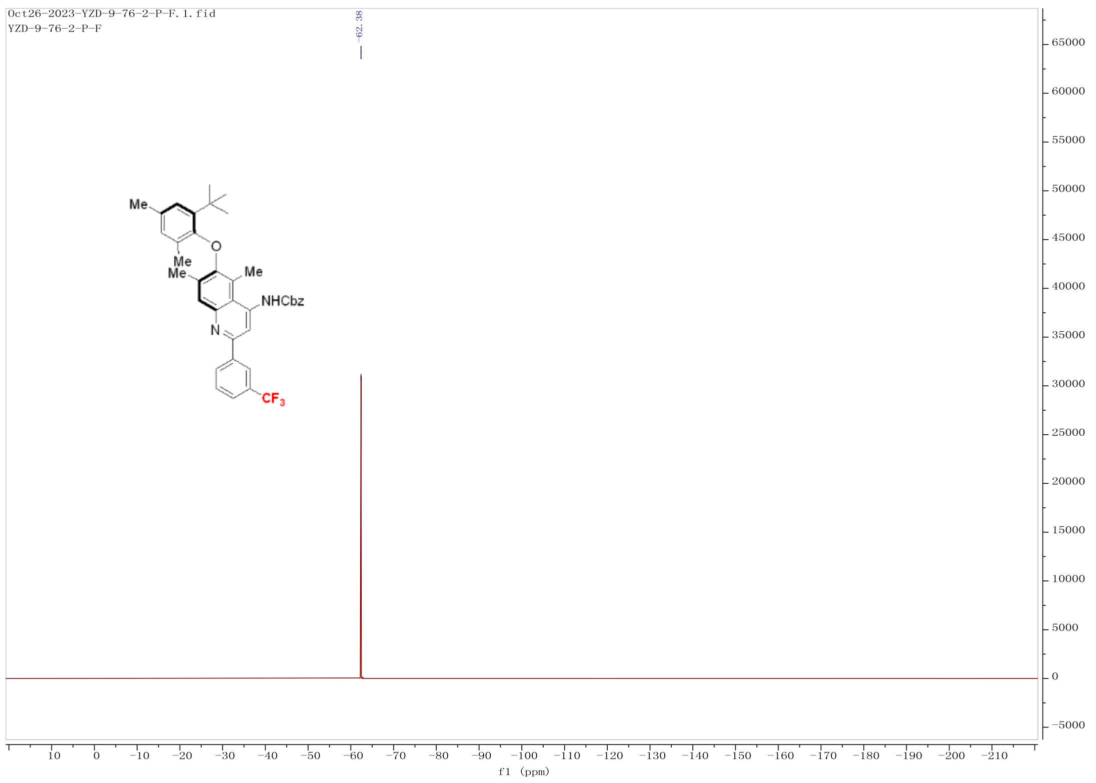

benzyl (6-(2-(tert-butyl)-4,6-dimethylphenoxy)-5,7-dimethyl-2-(o-tolyl)quinolin-4-yl) carbamate (**4f**)

4f: ^{1}H NMR (500 MHz, Chloroform-*d*)

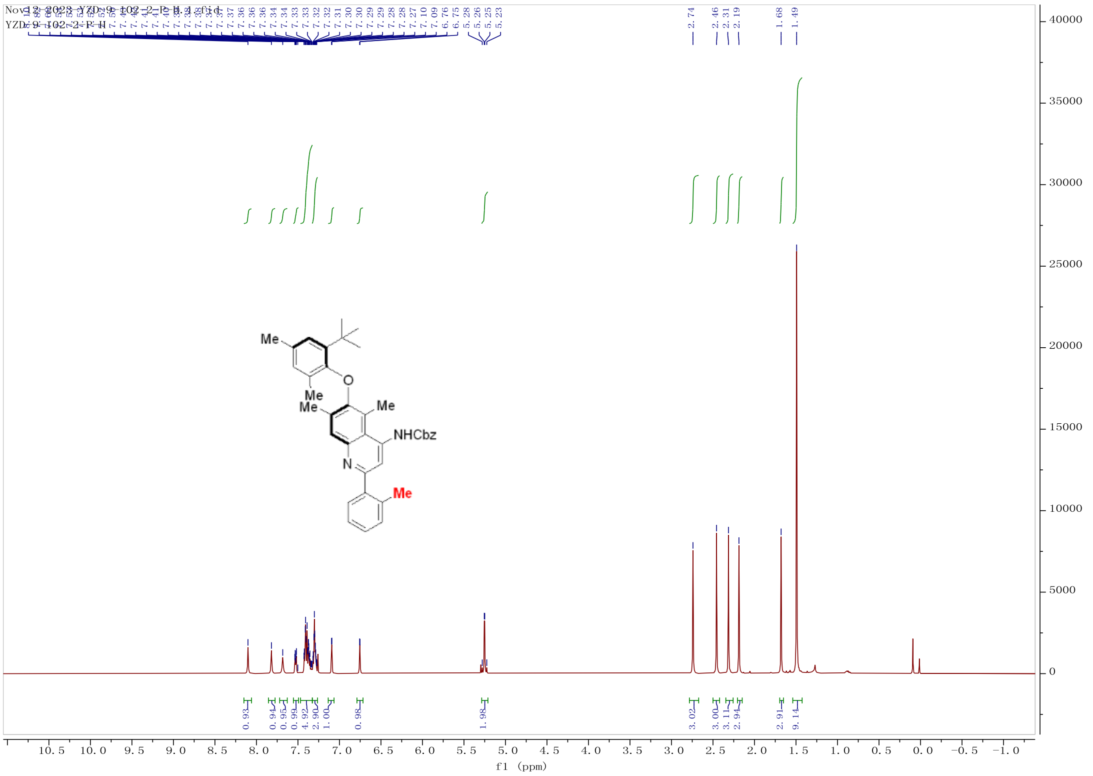

4f: ^{13}C NMR (126 MHz, Chloroform-*d*)

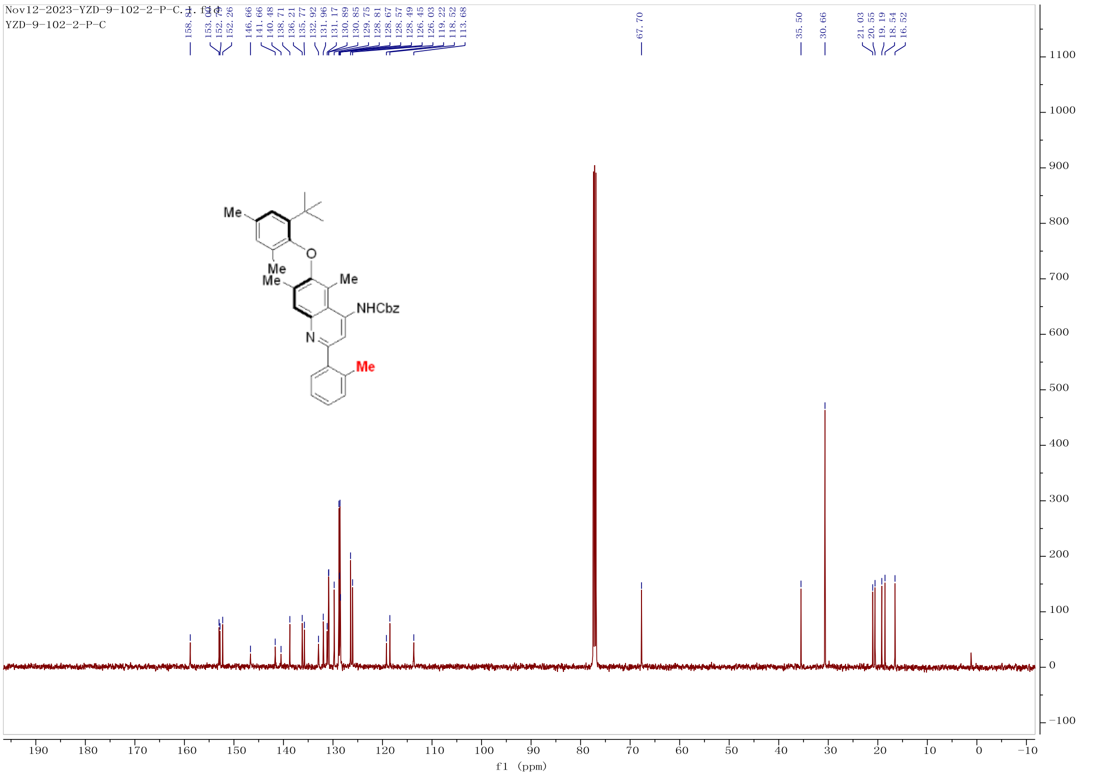

benzyl (2-(2-bromophenyl)-6-(2-(tert-butyl)-4,6-dimethylphenoxy)-5,7-dimethylquinolin-4-yl)carbamate (**4g**)

4g: ^{1}H NMR (400 MHz, Chloroform-*d*)

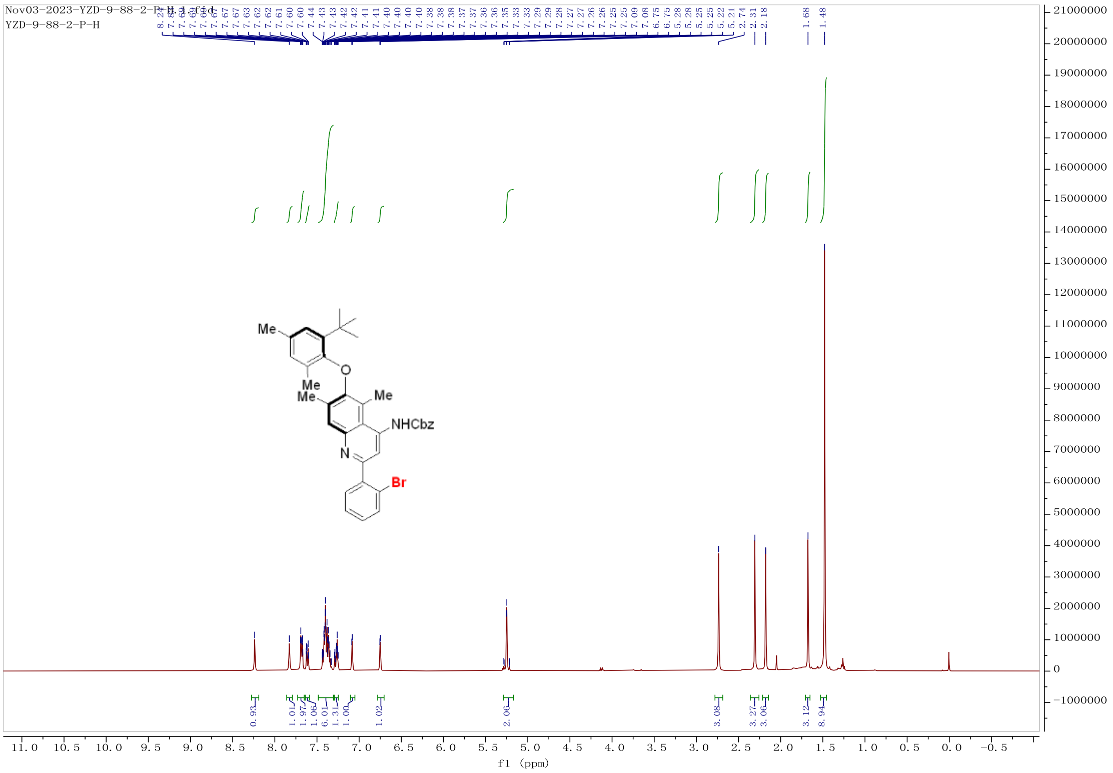

4g: ^{13}C NMR (101 MHz, Chloroform-*d*)

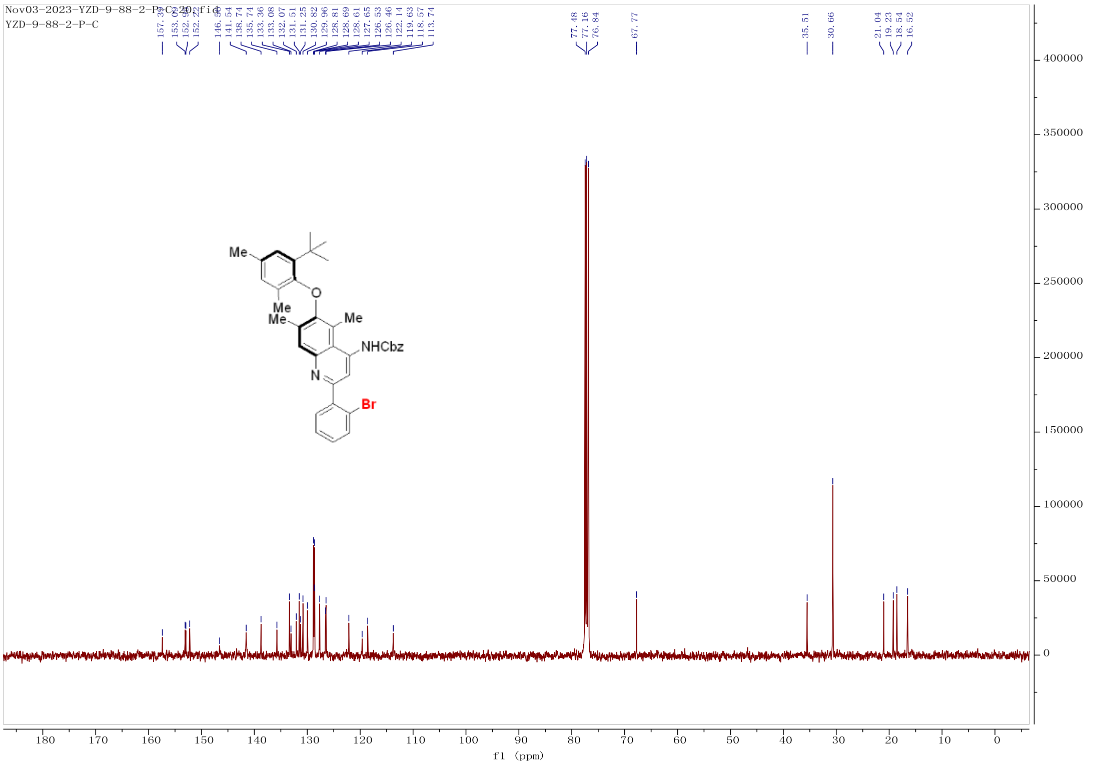

benzyl (6-(2-(tert-butyl)-4,6-dimethylphenoxy)-2-(3,4-dichlorophenyl)-5,7-dimethylquinolin-4-yl)carbamate (**4h**)

4h: ^{1}H NMR (500 MHz, Chloroform-*d*)

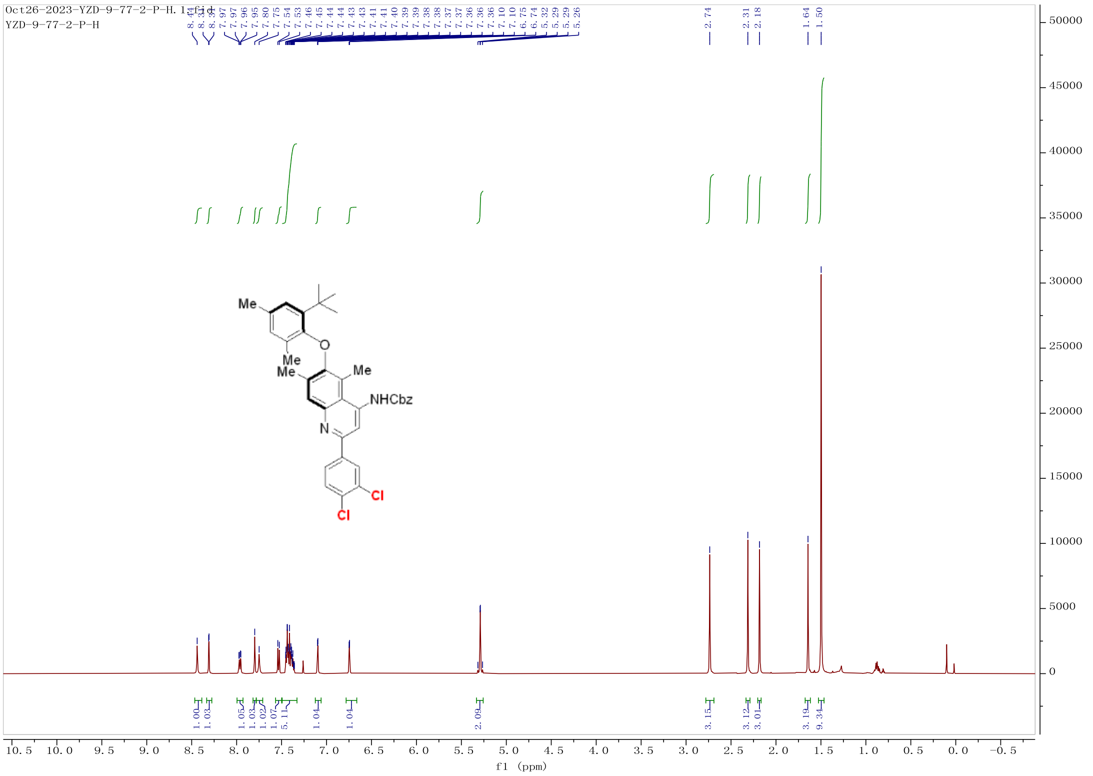

4h: ^{13}C NMR (126 MHz, Chloroform-*d*)

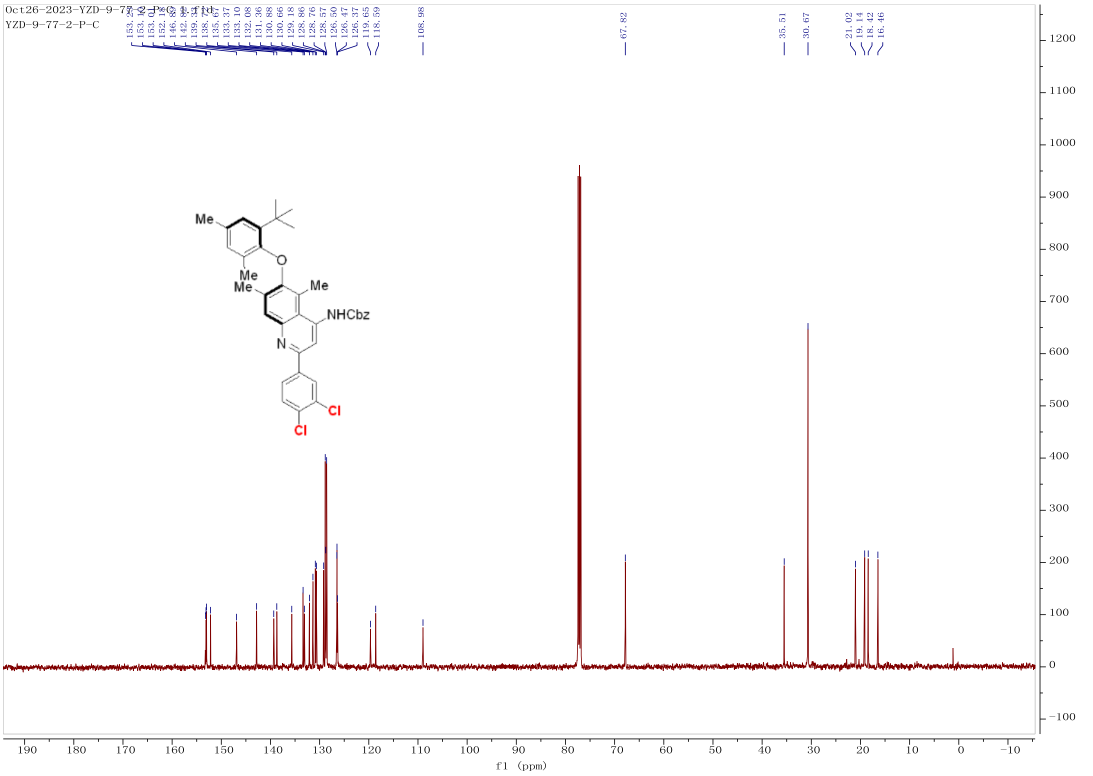

benzyl (6-(2-(tert-butyl)-4,6-dimethylphenoxy)-5,7-dimethyl-2-(naphthalen-2-yl)quinolin-4-yl)carbamate (**4i**)

4i: ^{1}H NMR (500 MHz, Chloroform-*d*)

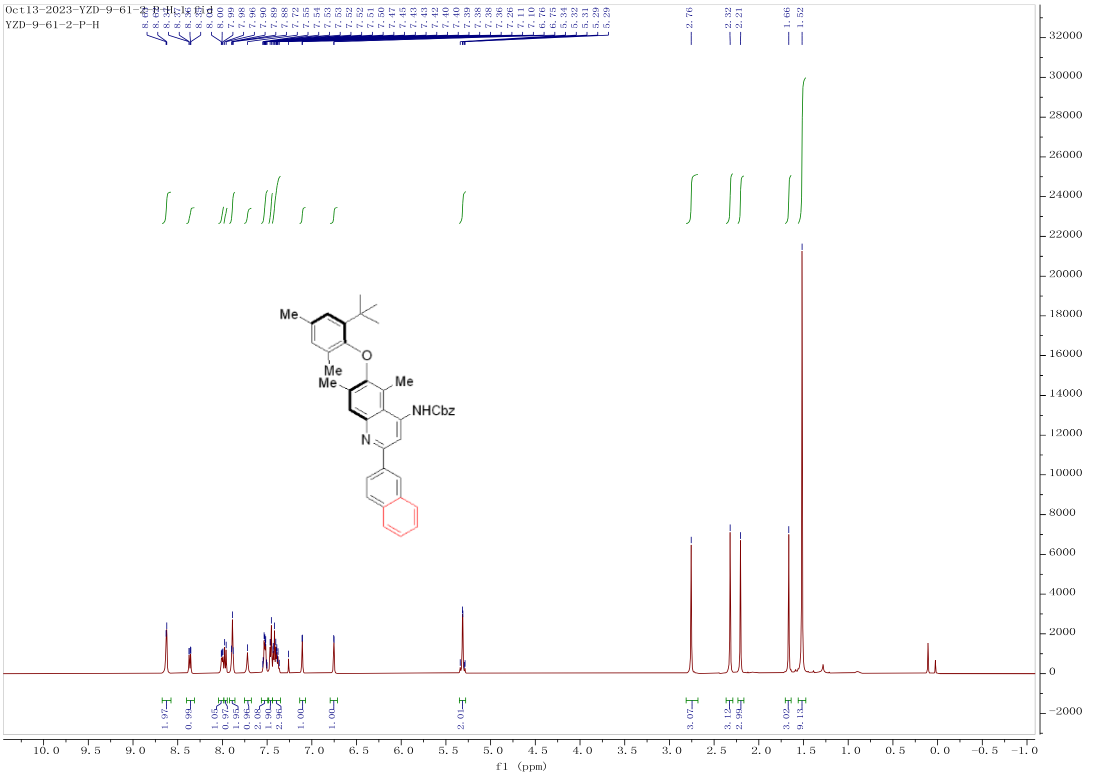

4i: ^{13}C NMR (126 MHz, Chloroform-*d*)

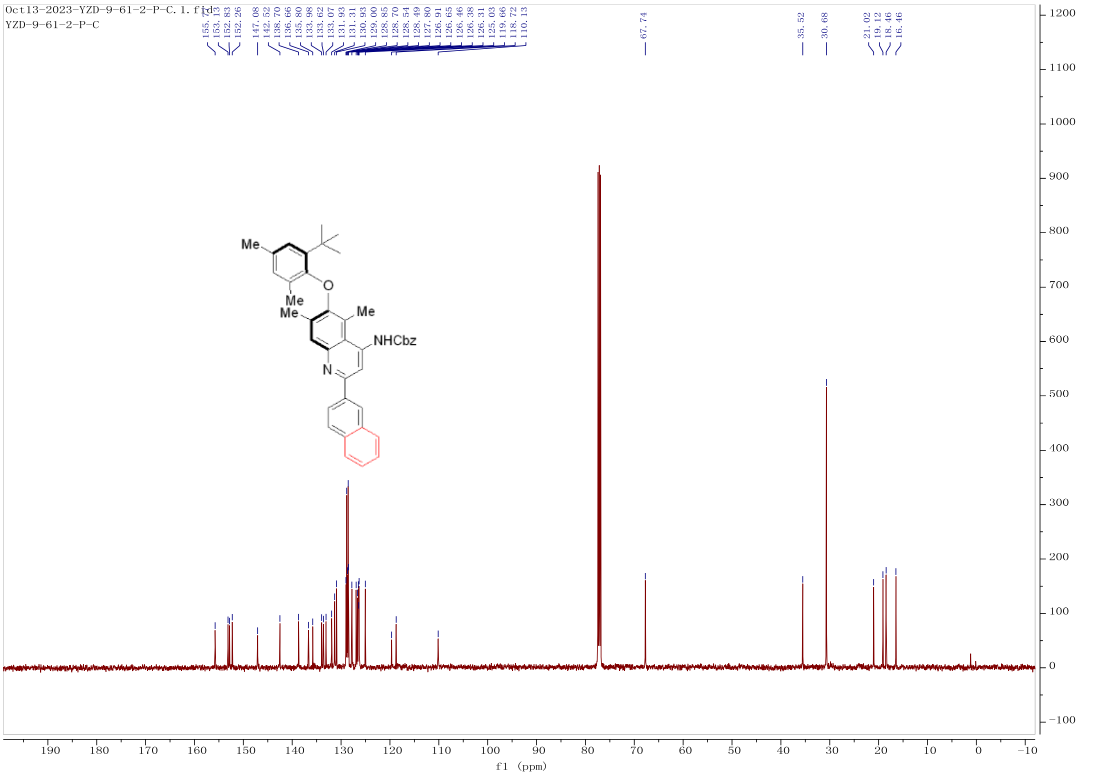

benzyl (6-(2-(tert-butyl)-4,6-dimethylphenoxy)-5,7-dimethyl-2-(naphthalen-1-yl)quinolin-4-yl)carbamate (**4j**)

4j: ^{1}H NMR (500 MHz, Chloroform-*d*)

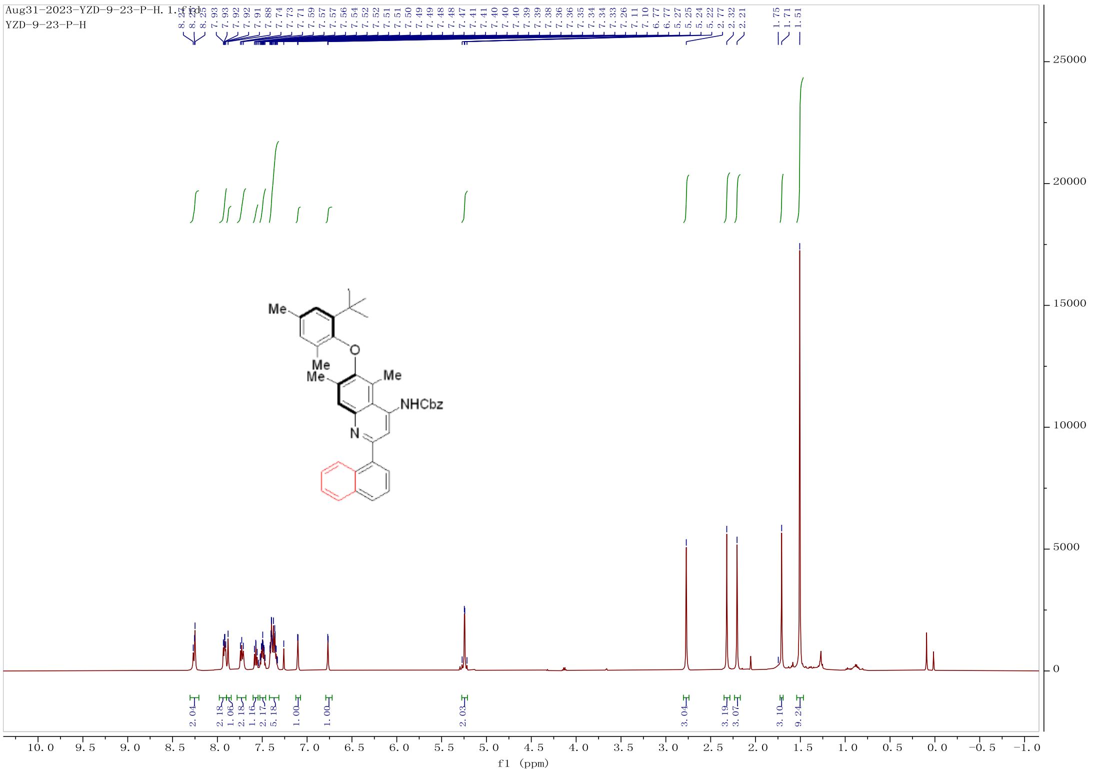

4j: ^{13}C NMR (126 MHz, Chloroform-*d*)

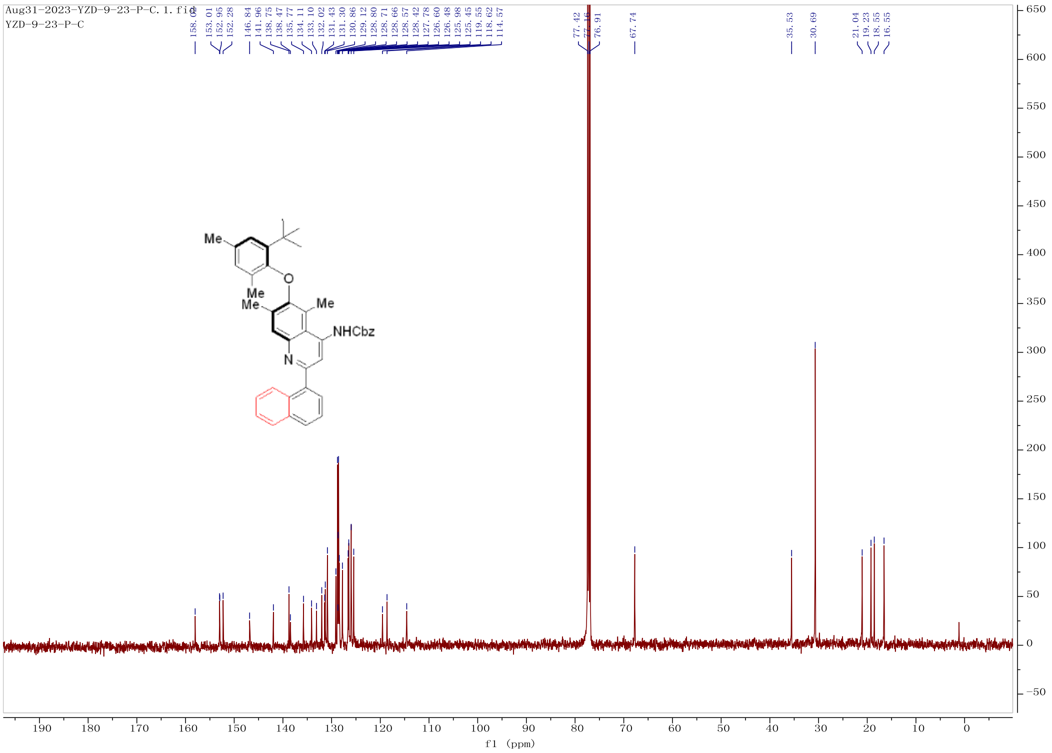

benzyl (6-(2-(tert-butyl)-4,6-dimethylphenoxy)-5,7-dimethyl-2-propylquinolin-4-yl)carbamate (**4k**)

4k: ^{1}H NMR (400 MHz, Chloroform-*d*)

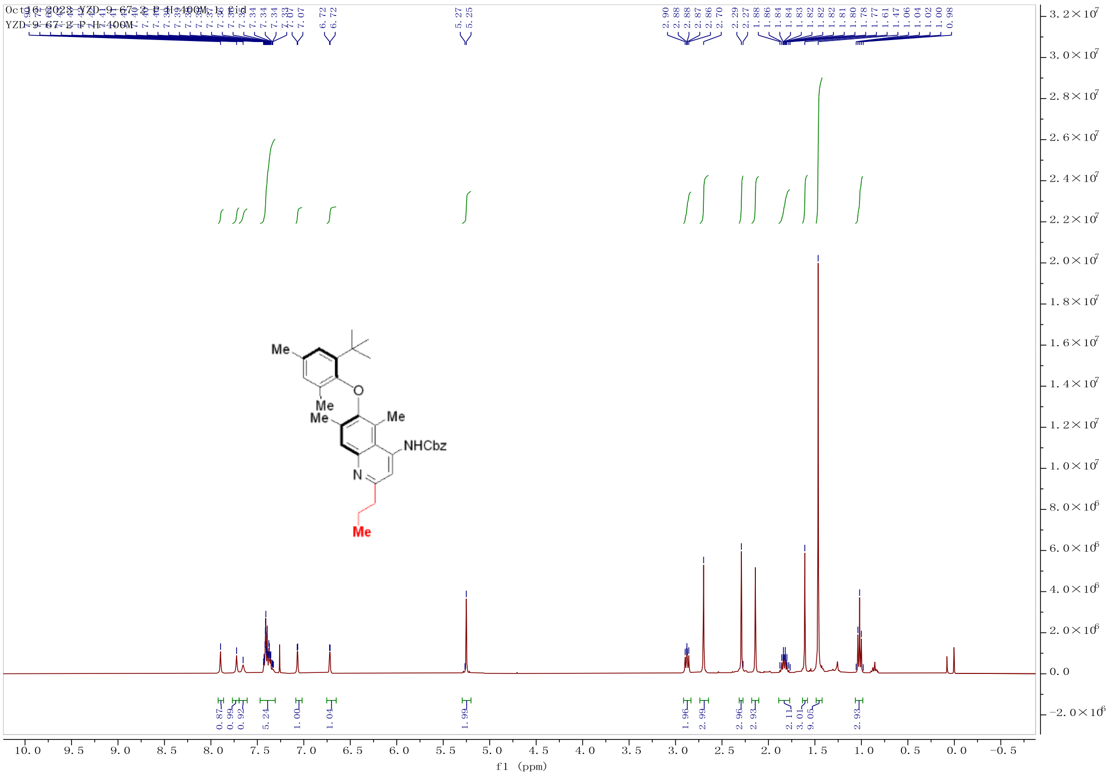

4k: ^{13}C NMR (101 MHz, Chloroform-*d*)

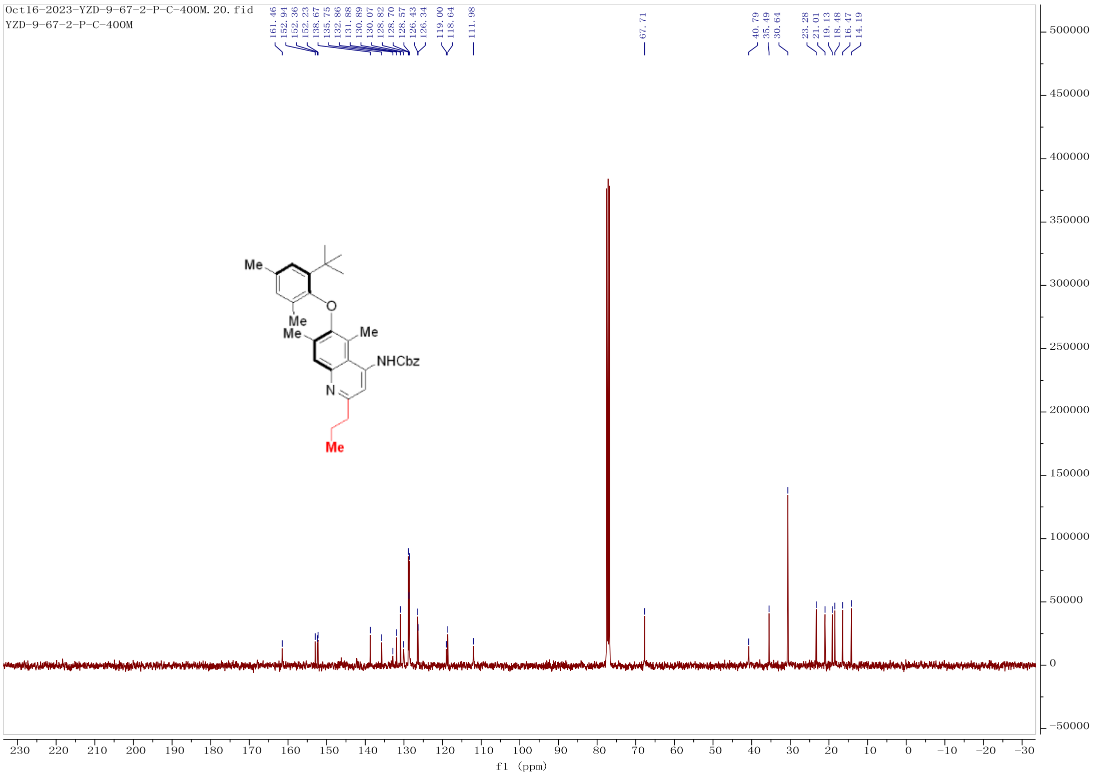

benzyl (6-(2-(tert-butyl)-4,6-dimethylphenoxy)-2-isopropyl-5,7-dimethylquinolin-4-yl)carbamate (**4l**)

4l: ^{1}H NMR (500 MHz, Chloroform-*d*)

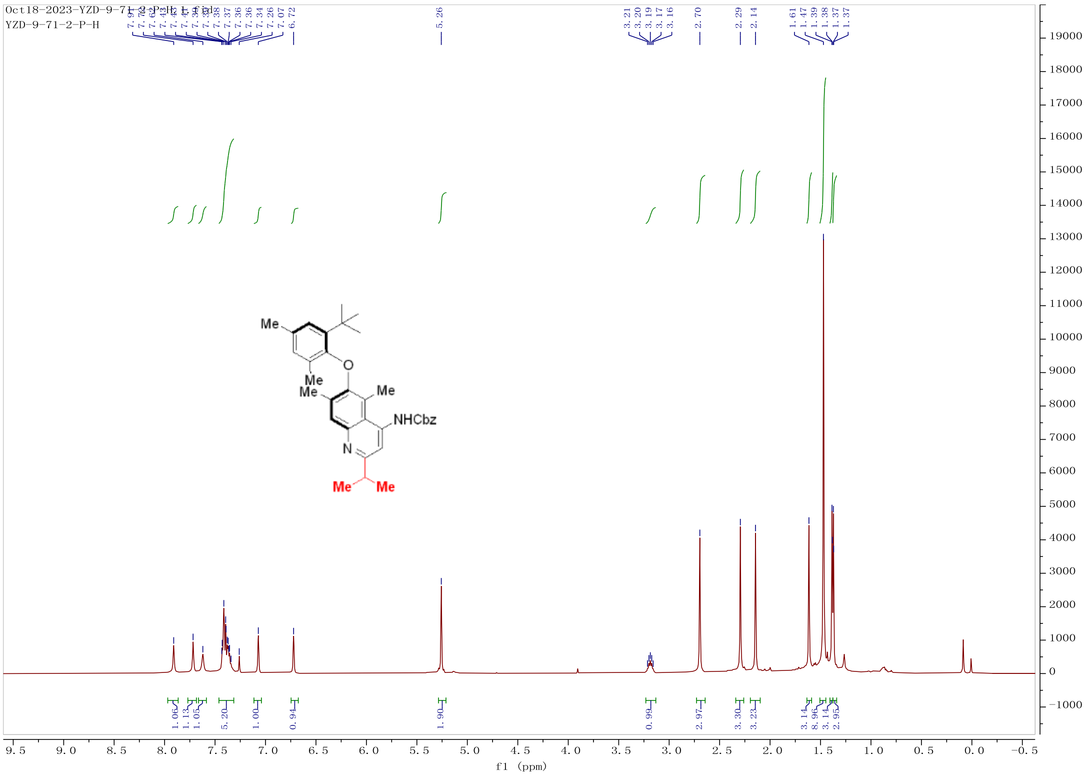

4l: ^{13}C NMR (126 MHz, Chloroform-*d*)

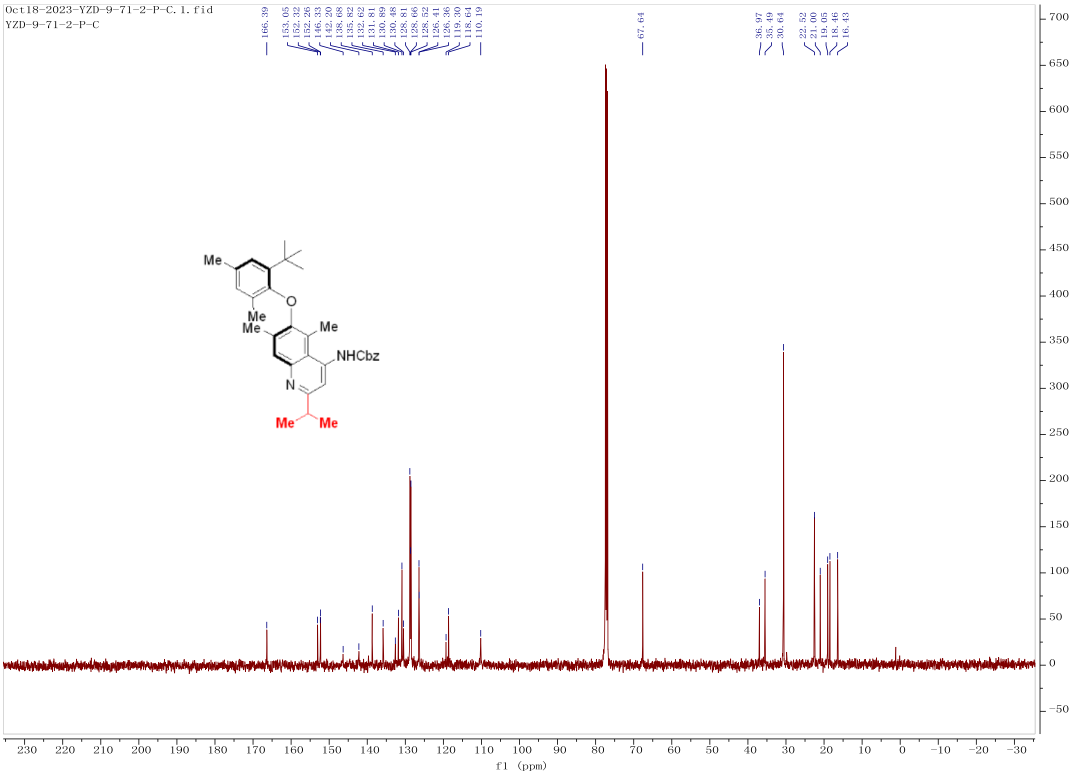

benzyl (6-(2-(tert-butyl)-4,6-dimethylphenoxy)-2-cyclohexyl-5,7-dimethylquinolin-4-yl)carbamate (**4m**)

4m: ^{1}H NMR (500 MHz, Chloroform-*d*)

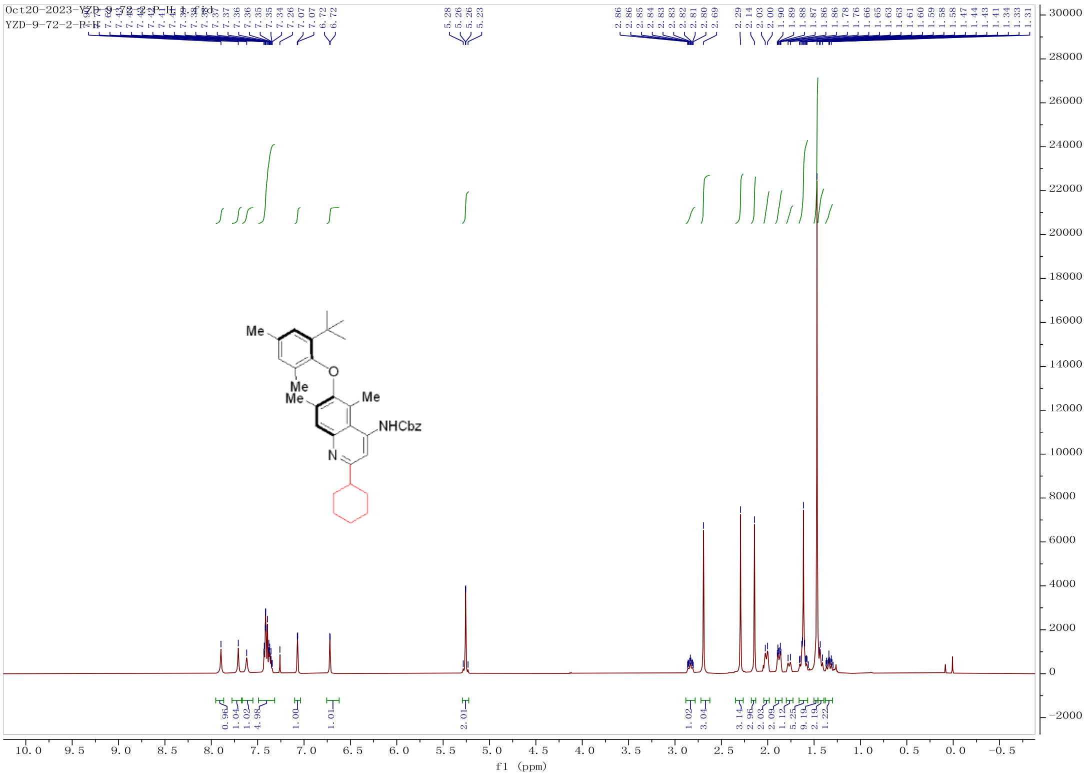

4m: ^{13}C NMR (126 MHz, Chloroform-*d*)

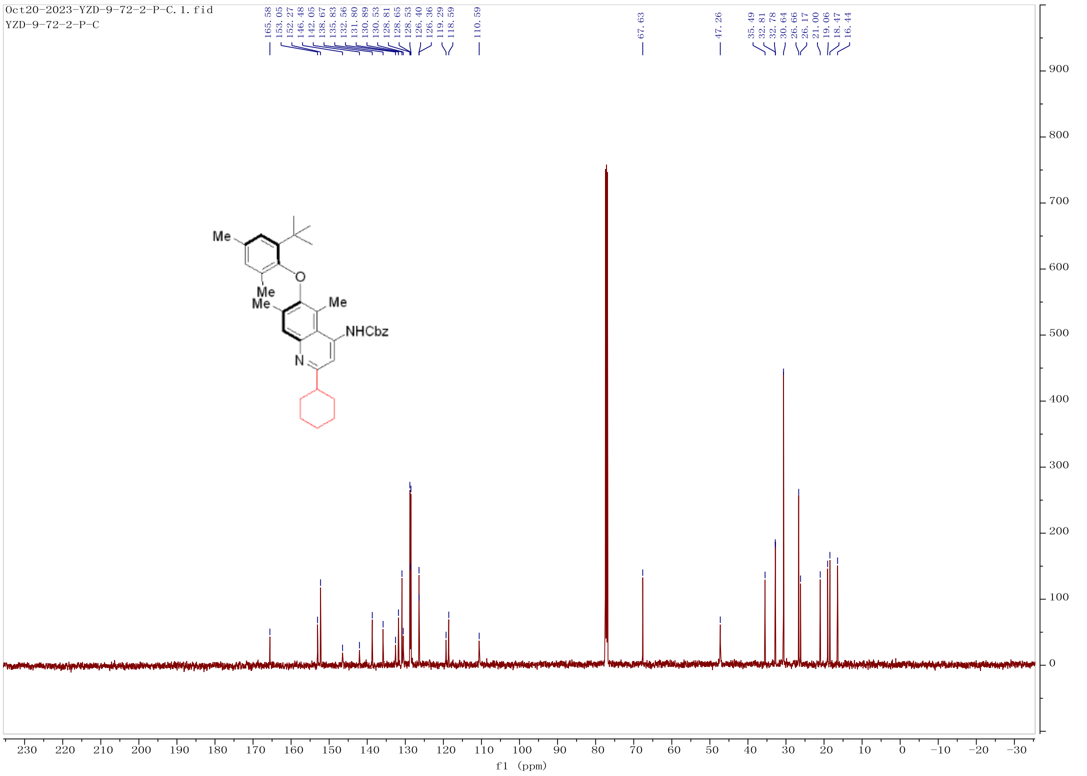

benzyl (6-((3-(tert-butyl)-5-methyl-[1,1'-biphenyl]-2-yl)oxy)-5,7-dimethyl-2-phenylquinolin-4-yl)carbamate (**4o**)

4o: ^{1}H NMR (500 MHz, Chloroform-*d*)

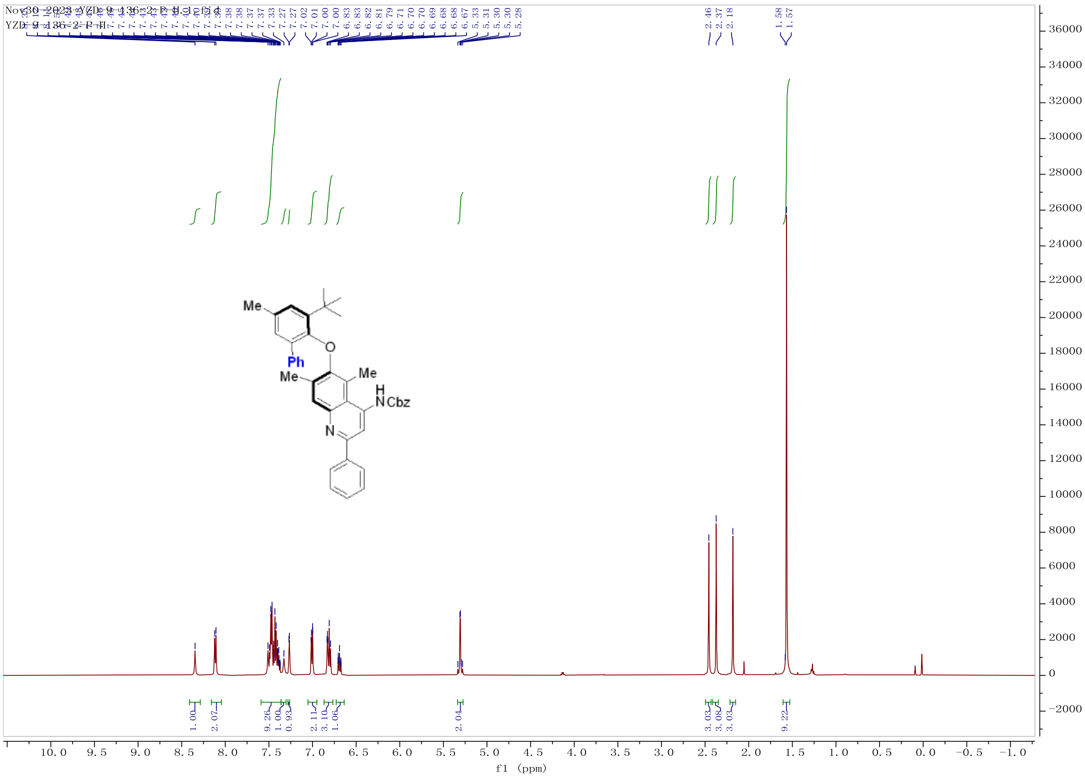

4o: ^{13}C NMR (126 MHz, Chloroform-*d*)

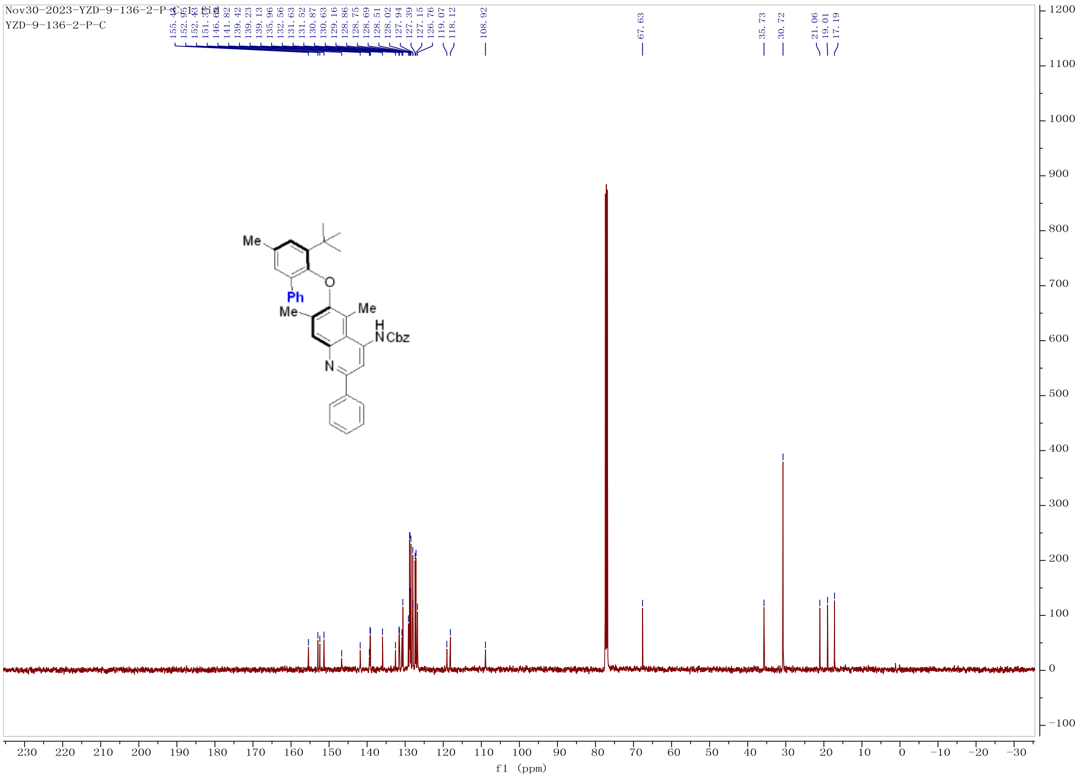

benzyl (6-((3-(tert-butyl)-5-methyl-3'-(trifluoromethyl)-[1,1'-biphenyl]-2-yl)oxy)-5,7-dimethyl-2-phenylquinolin-4-yl)carbamate (**4p**)

4p: ^{1}H NMR (500 MHz, Chloroform-*d*)

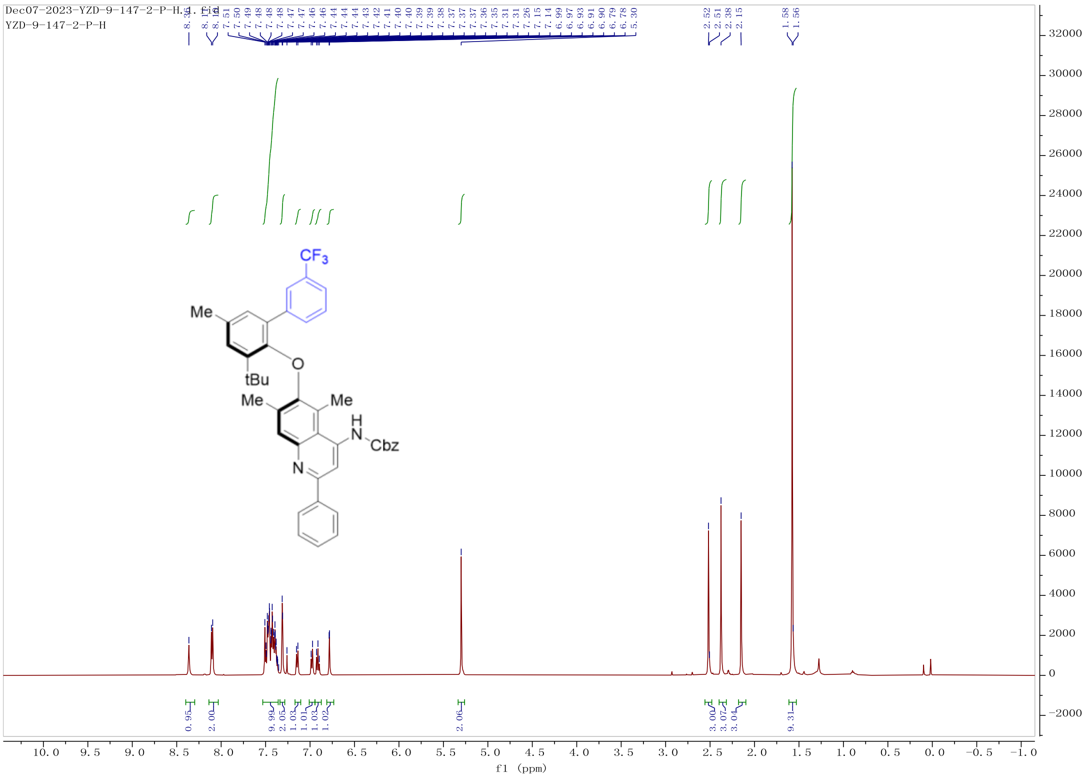

4p: ^{13}C NMR (126 MHz, Chloroform-*d*)

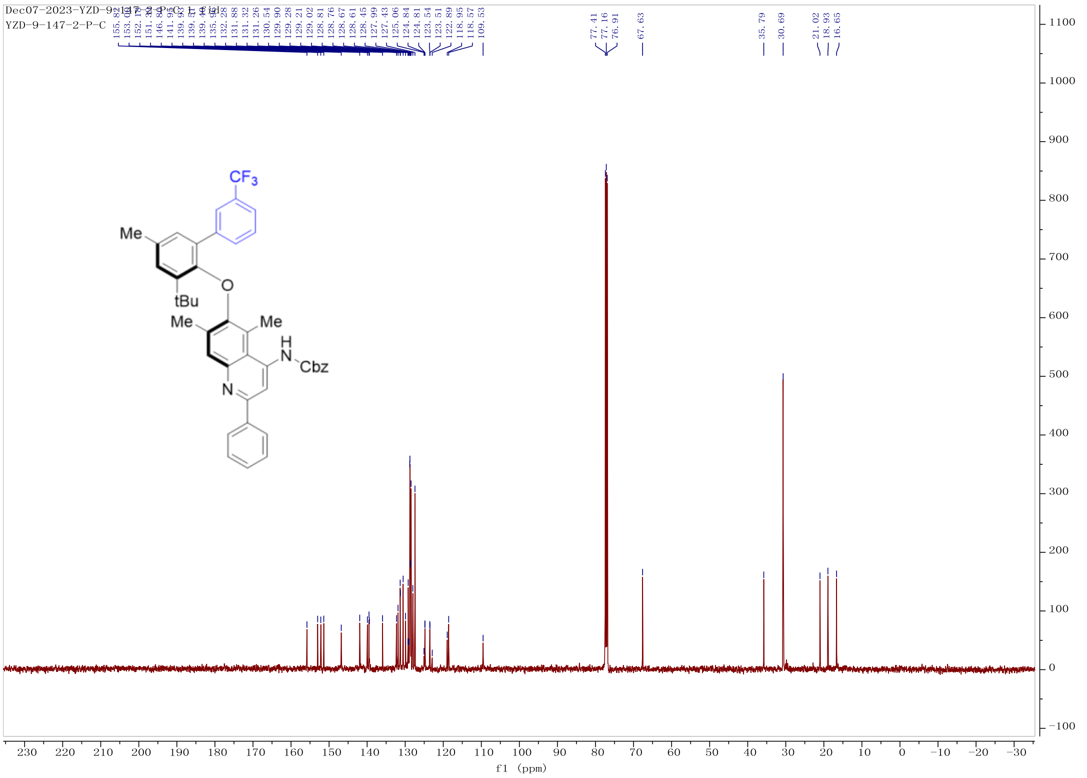

4p: ^{19}F NMR (471 MHz, Chloroform-*d*)

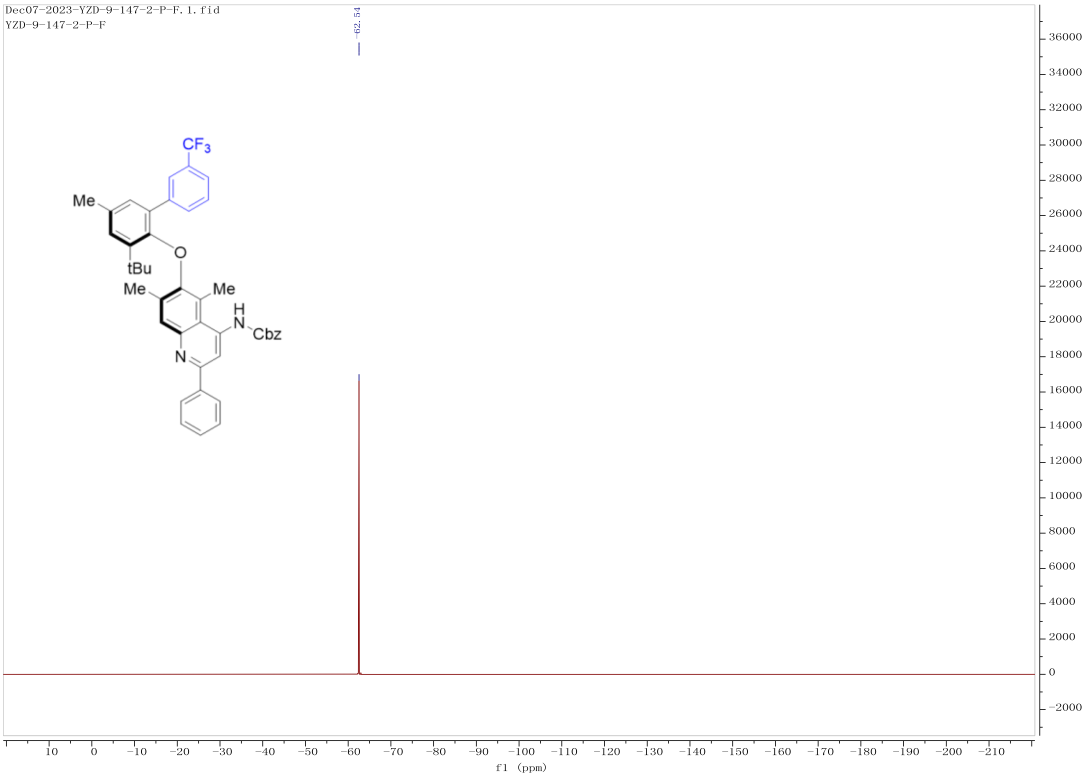

benzyl (6-(2-(tert-butyl)-4-methyl-6-(naphthalen-1-yl)phenoxy)-5,7-dimethyl-2-phenylquinolin-4-yl)carbamate (**4q**)

4q: ^{1}H NMR (500 MHz, Chloroform-*d*)

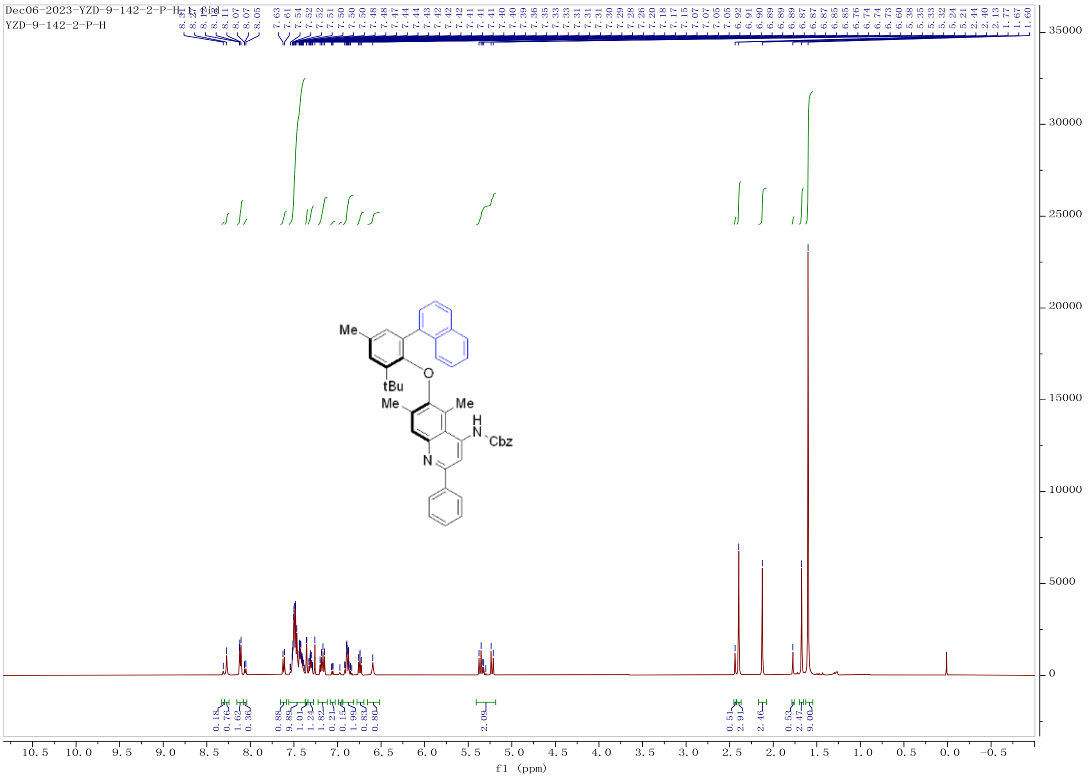

4q: ^{13}C NMR (126 MHz, Chloroform-*d*)

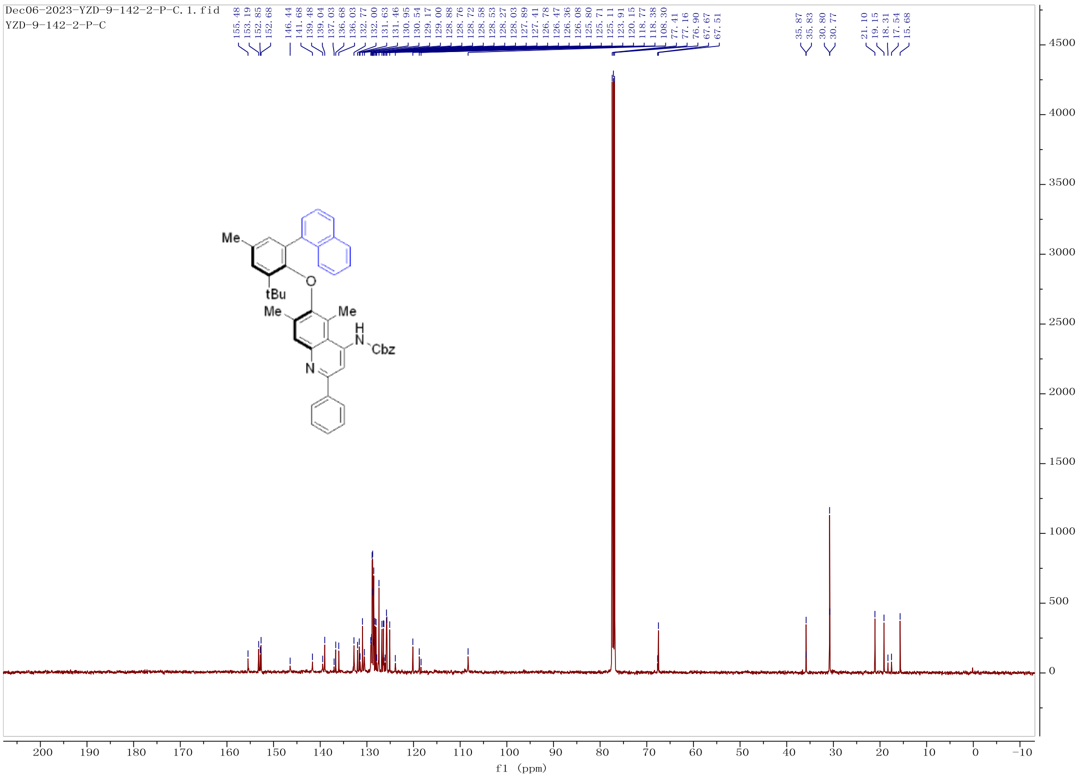

benzyl (6-((3-(tert-butyl)-5-methyl-2',3',4',5'-tetrahydro-[1,1'-biphenyl]-2-yl)oxy)-5,7-dimethyl-2-phenylquinolin-4-yl)carbamate (**4r**)

4r: ^{1}H NMR (500 MHz, Chloroform-*d*)

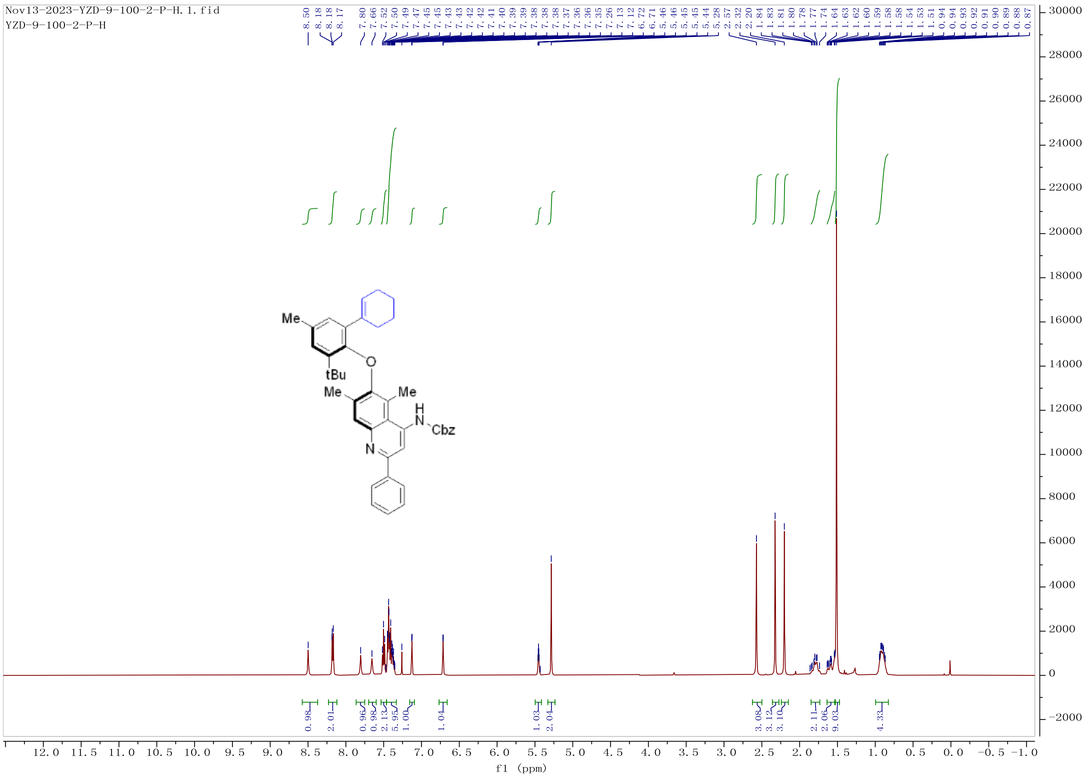

4r: ^{13}C NMR (126 MHz, Chloroform-*d*)

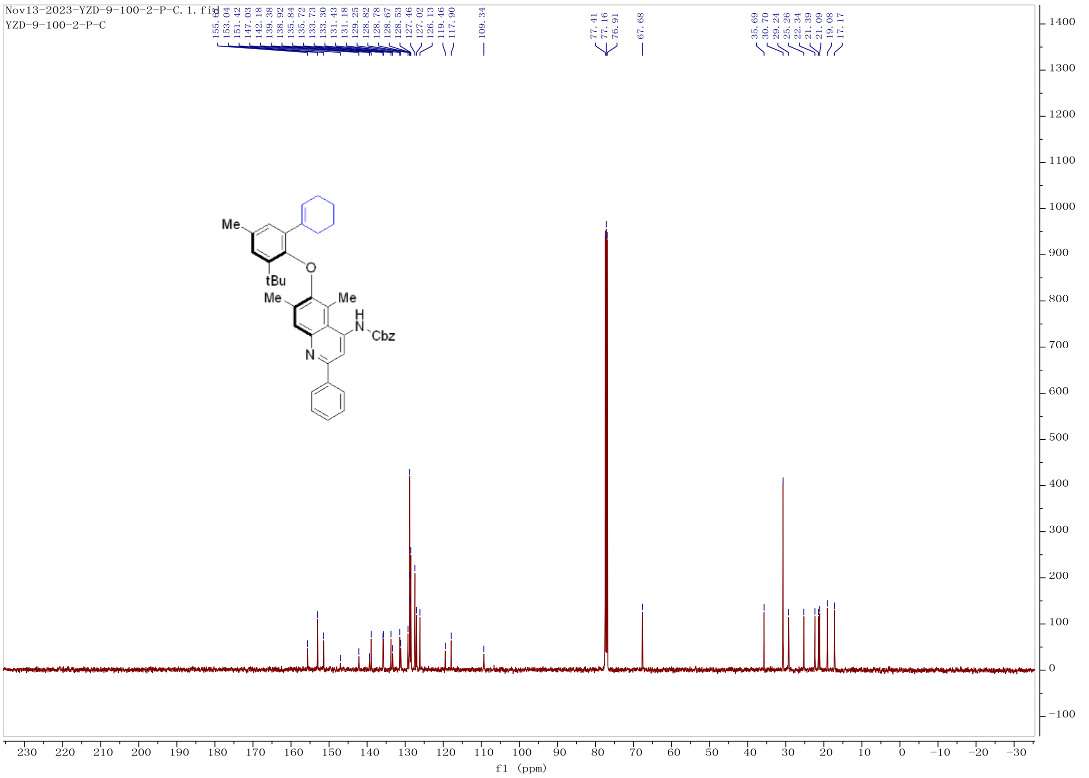

benzyl (6-(2-(tert-butyl)-6-methylphenoxy)-5,7-dimethyl-2-phenylquinolin-4-yl)carbamate (**4s**)

4s: ^{1}H NMR (500 MHz, Chloroform-*d*)

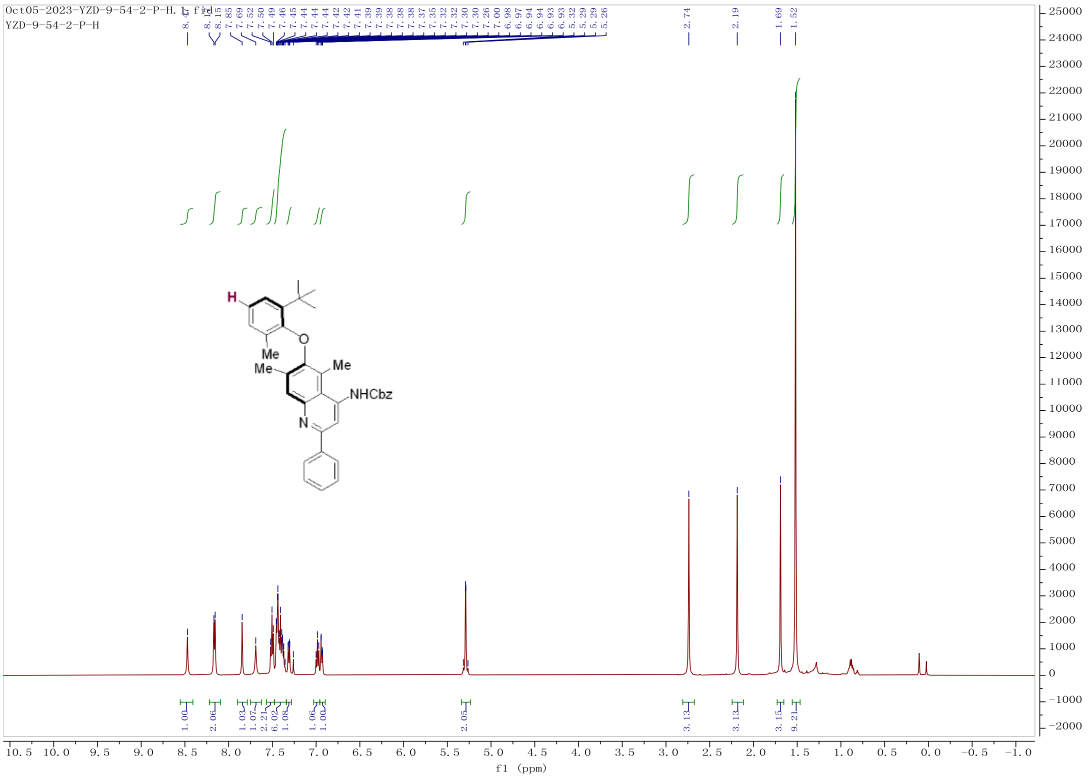

4s: ^{13}C NMR (126 MHz, Chloroform-*d*)

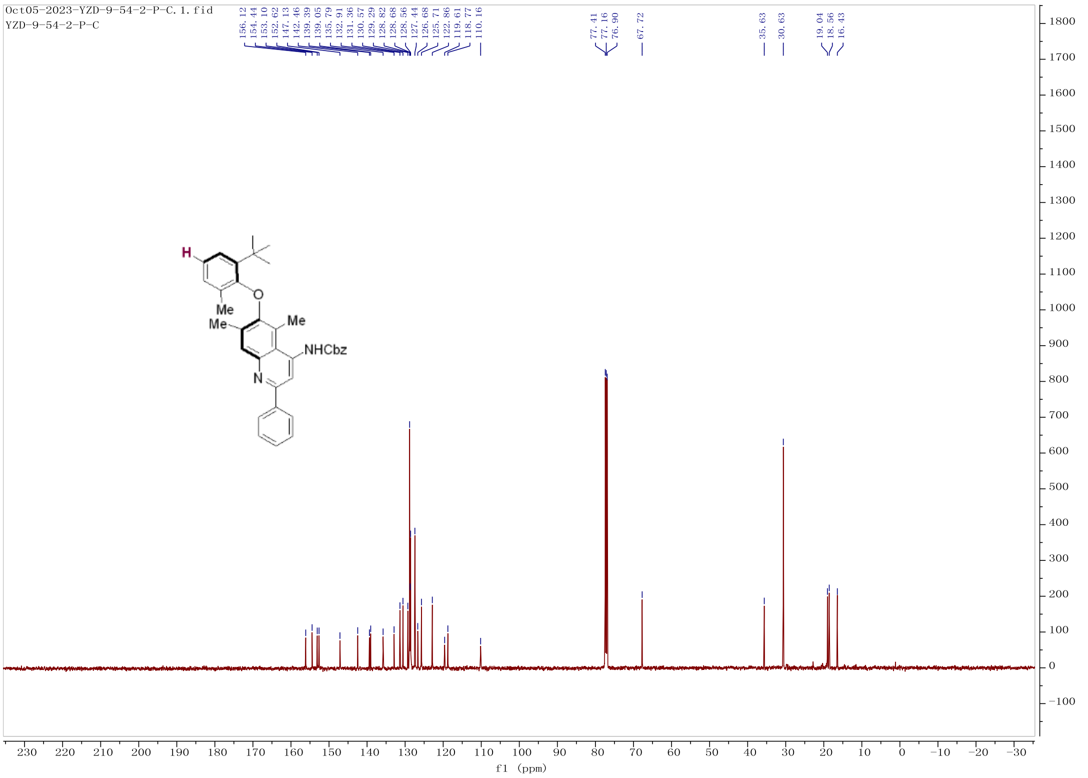

benzyl (6-((5'-(tert-butyl)-[1,1':3',1''-terphenyl]-4'-yl)oxy)-5,7-dimethyl-2-phenylquinolin-4-yl)carbamate (**4t**)

4t: ^{1}H NMR (500 MHz, Chloroform-*d*)

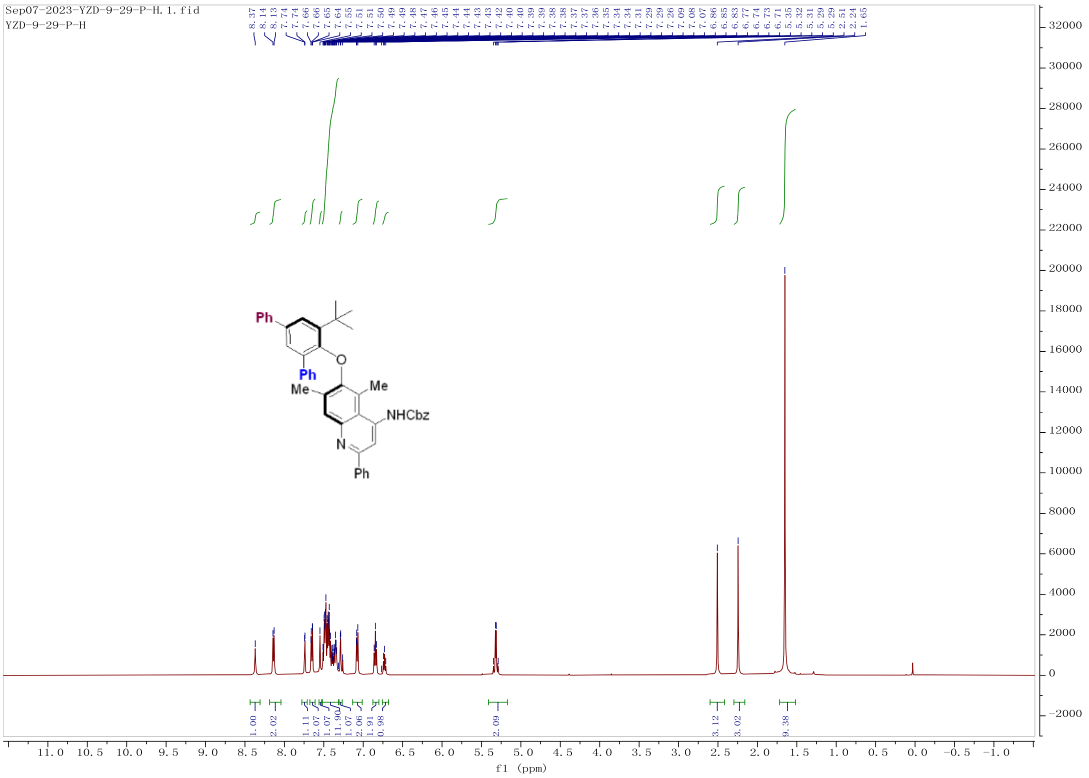

4t: ^{13}C NMR (126 MHz, Chloroform-*d*)

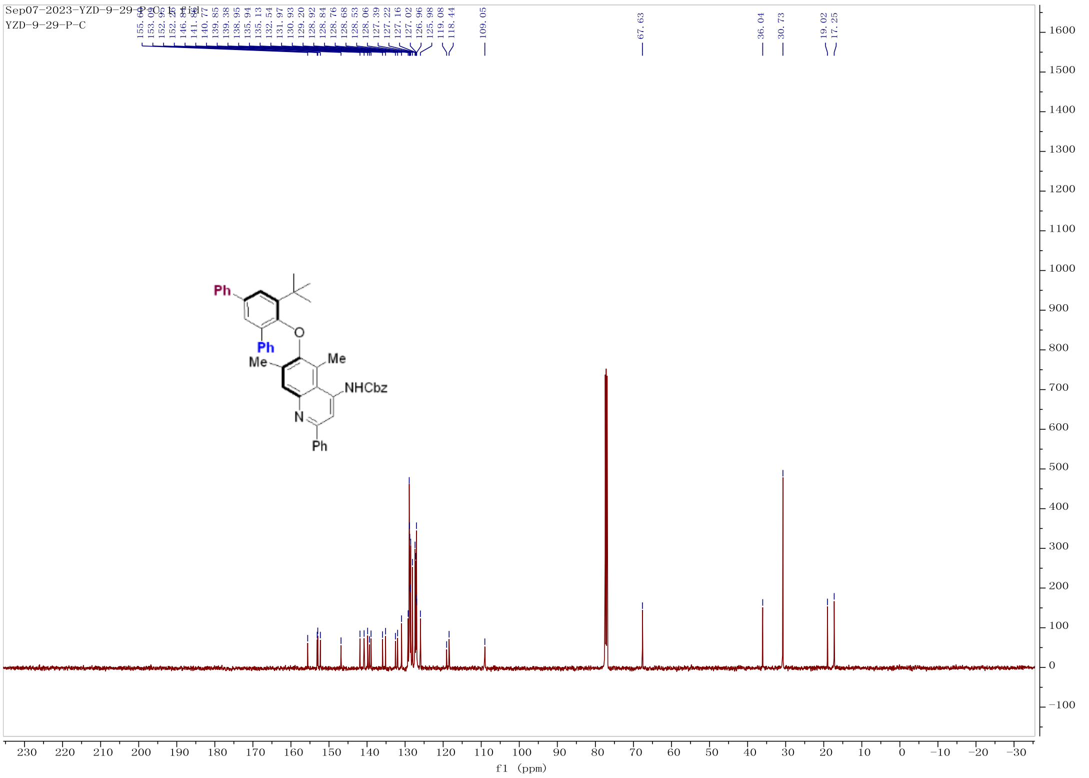

benzyl (6-((5'-(tert-butyl)-4,4"-dimethoxy-[1,1':3',1"-terphenyl]-4'-yl)oxy)-5,7-dimethyl-2-phenylquinolin-4-yl)carbamate (**4u**)

4u: ^{1}H NMR (500 MHz, Chloroform-*d*)

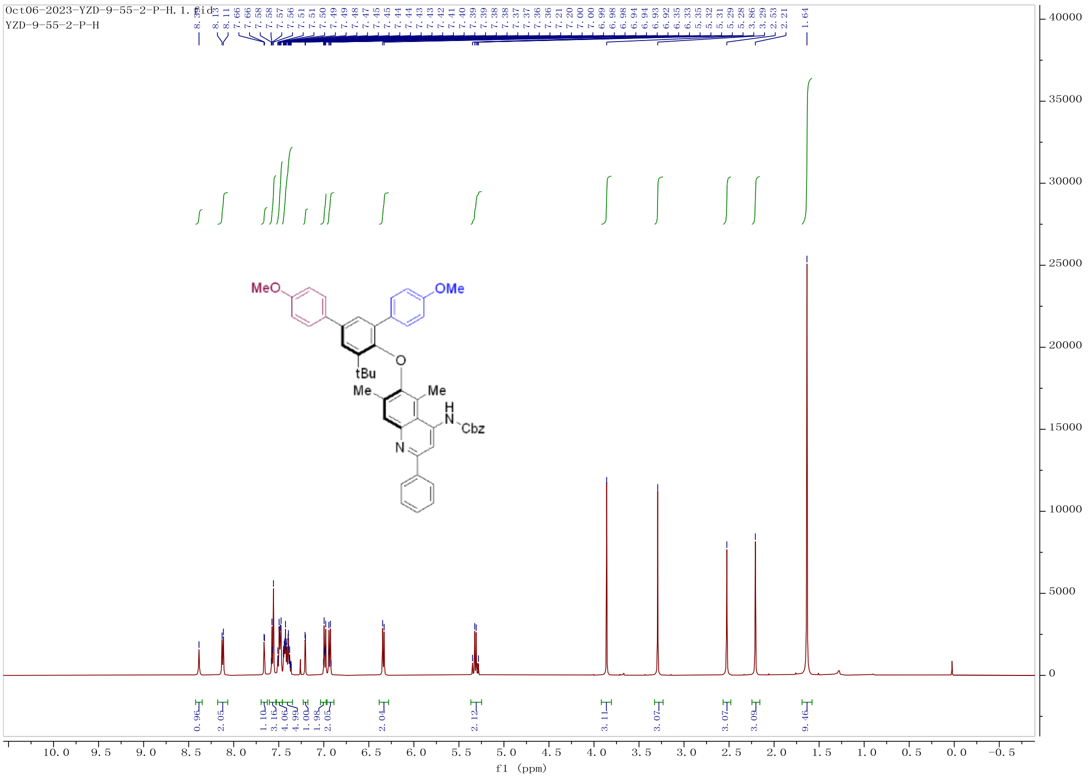

4u: ^{13}C NMR (126 MHz, Chloroform-*d*)

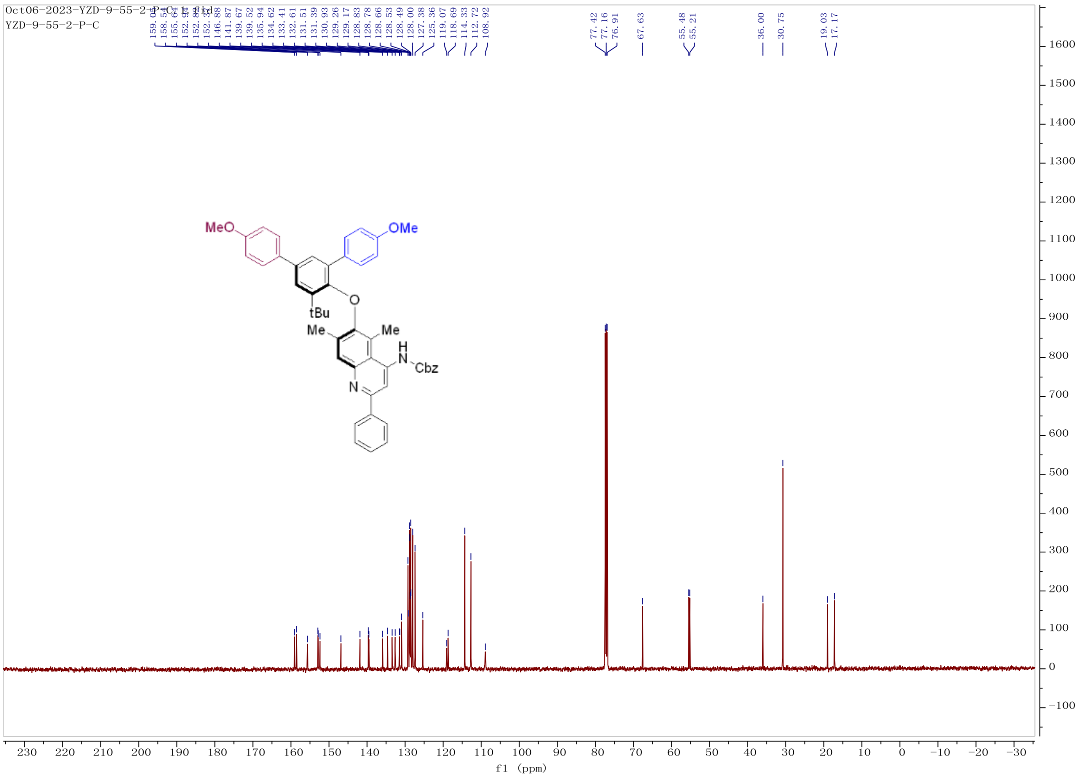

benzyl (6-(2-(tert-butyl)-4,6-dimethylphenoxy)-5,7-diethyl-2-phenylquinolin-4-yl)carbamate (**4v**)

4v: ^{1}H NMR (500 MHz, Chloroform-*d*)

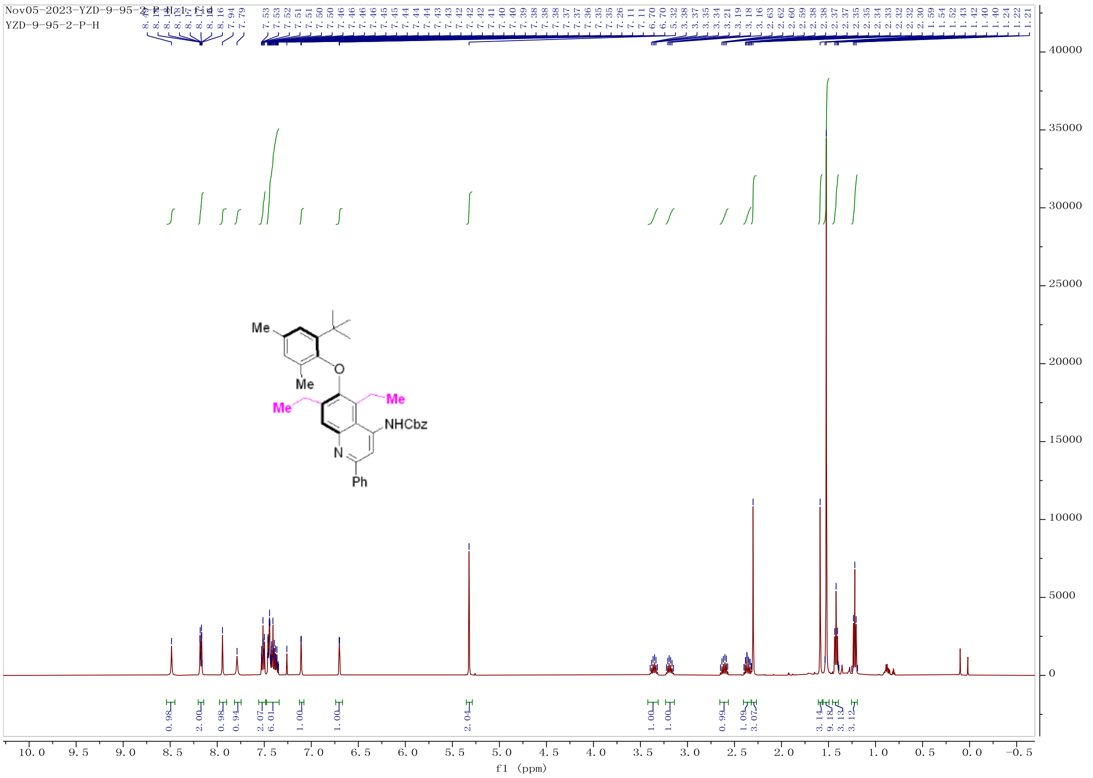

4v: ^{13}C NMR (126 MHz, Chloroform-*d*)

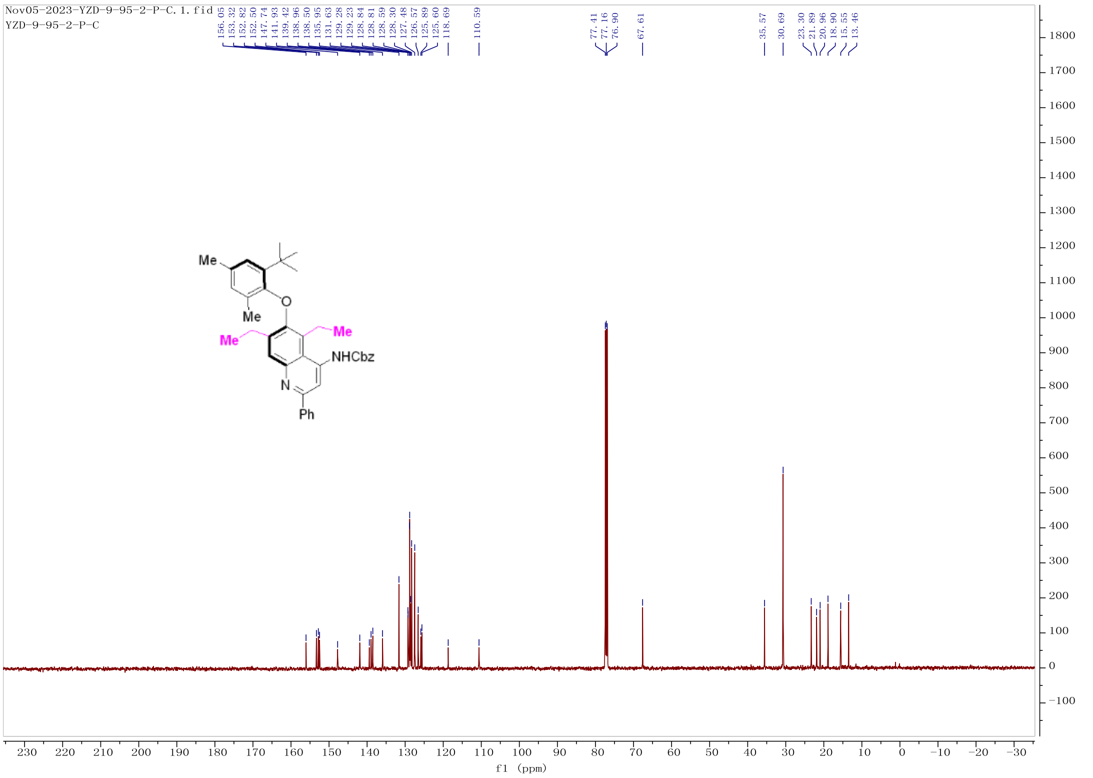

benzyl-(6-(2-(tert-butyl)-4,6-dimethylphenoxy)-2,5,7-triphenylquinolin-4-yl)carbamate (**4w**)

4w: ^{1}H NMR (500 MHz, Chloroform-*d*)

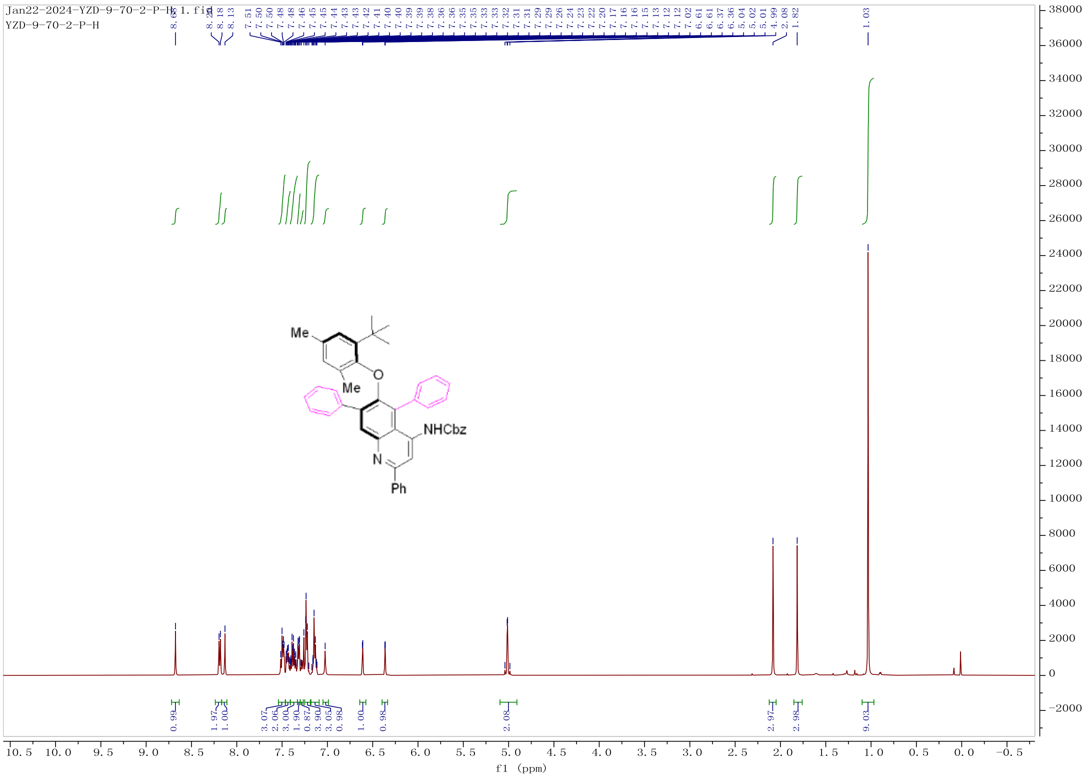

4w: ^{13}C NMR (126 MHz, Chloroform-*d*)

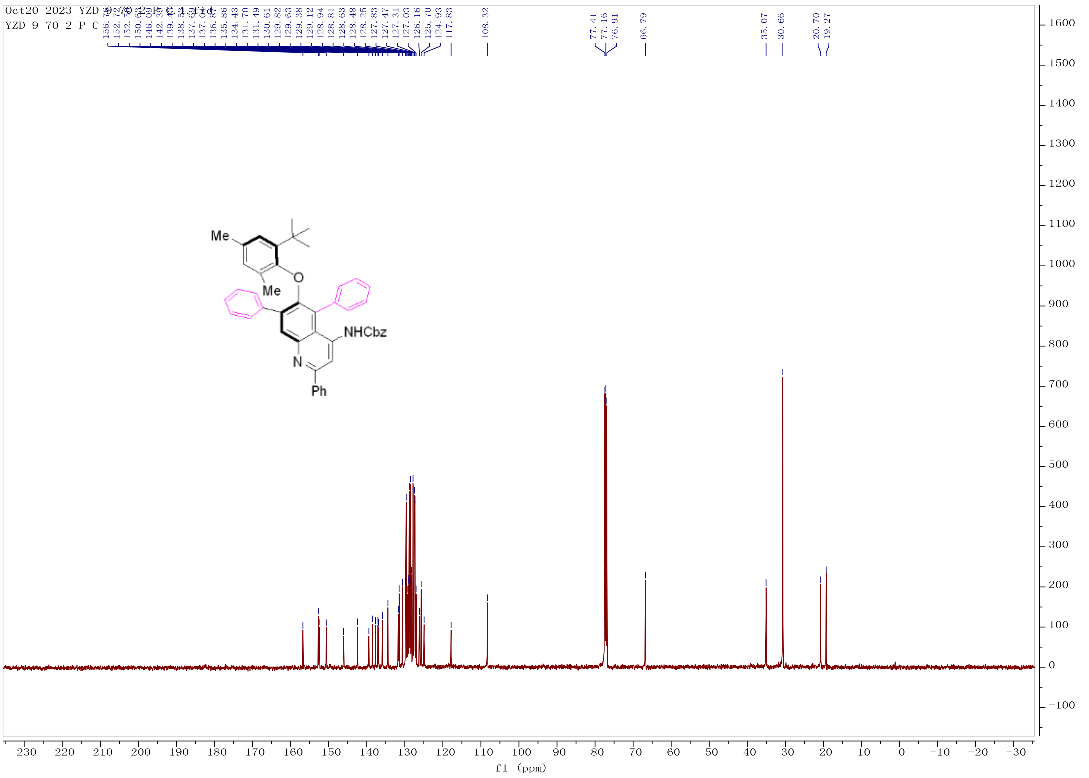

benzyl-(6-(2-(tert-butyl)-4,6-dimethylphenoxy)-5,7-bis(4-methoxyphenyl)-2-phenylquinolin-4-yl)carbamate (**4x**)

4x: ^{1}H NMR (500 MHz, Chloroform-*d*)

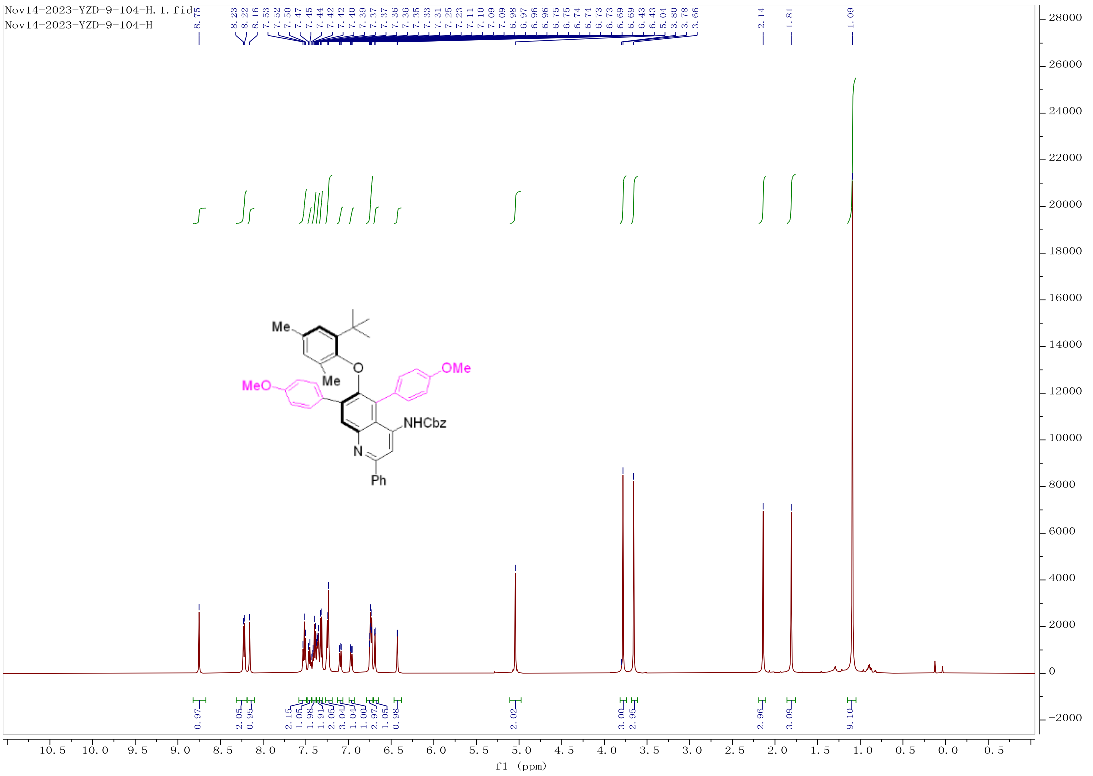

4x: ^{13}C NMR (126 MHz, Chloroform-*d*)

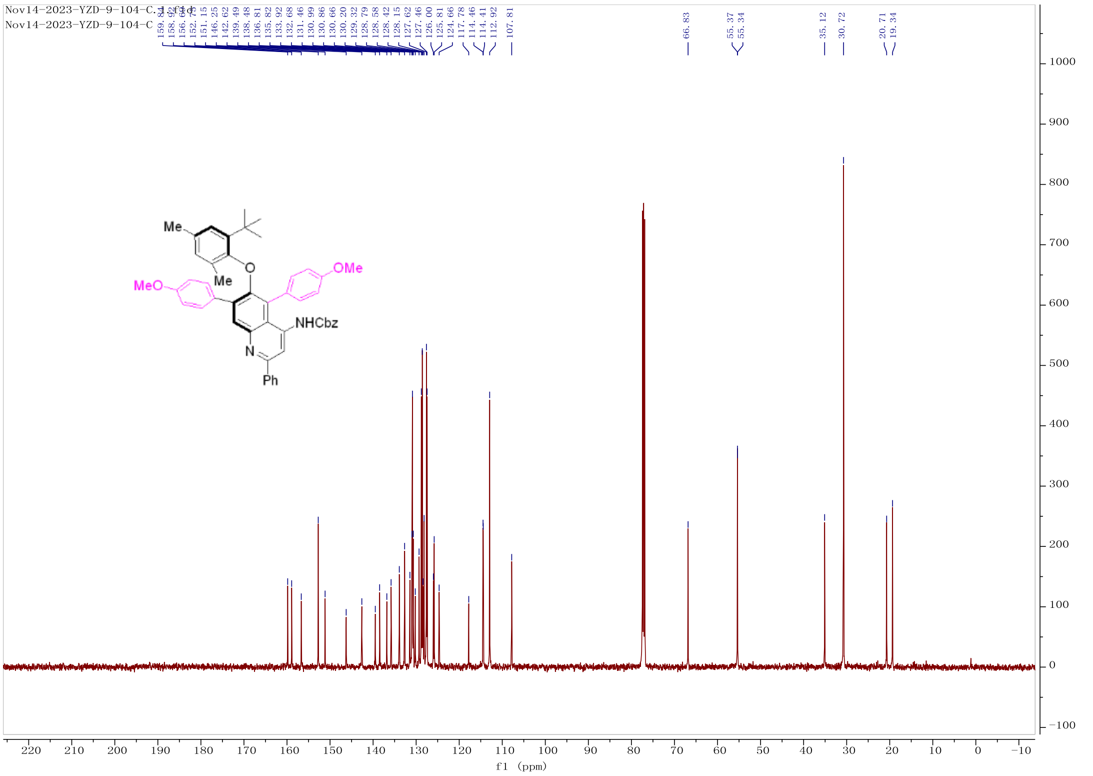

(*R*)-6-(2-(tert-butyl)-4,6-dimethylphenoxy)-5,7-bis(2-methoxyphenyl)-2-phenylquinoline (**4y**)

4y: ^{1}H NMR (500 MHz, Chloroform-*d*)

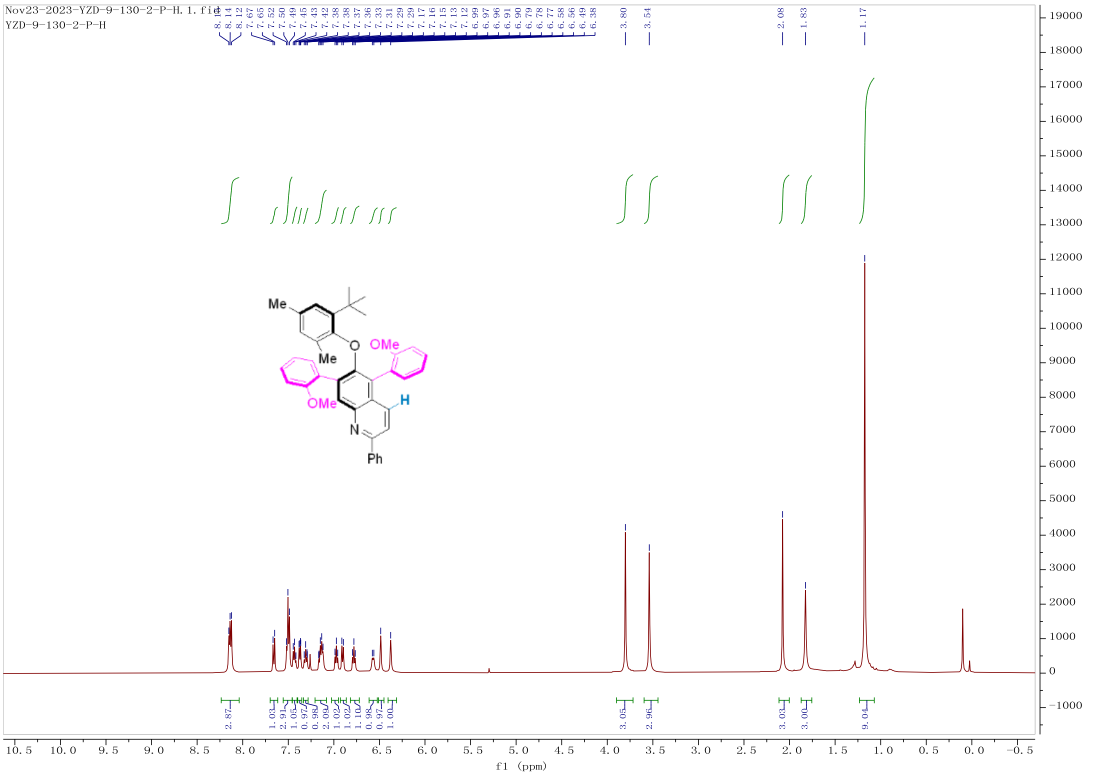

4y: ^{13}C NMR (126 MHz, Chloroform-*d*)

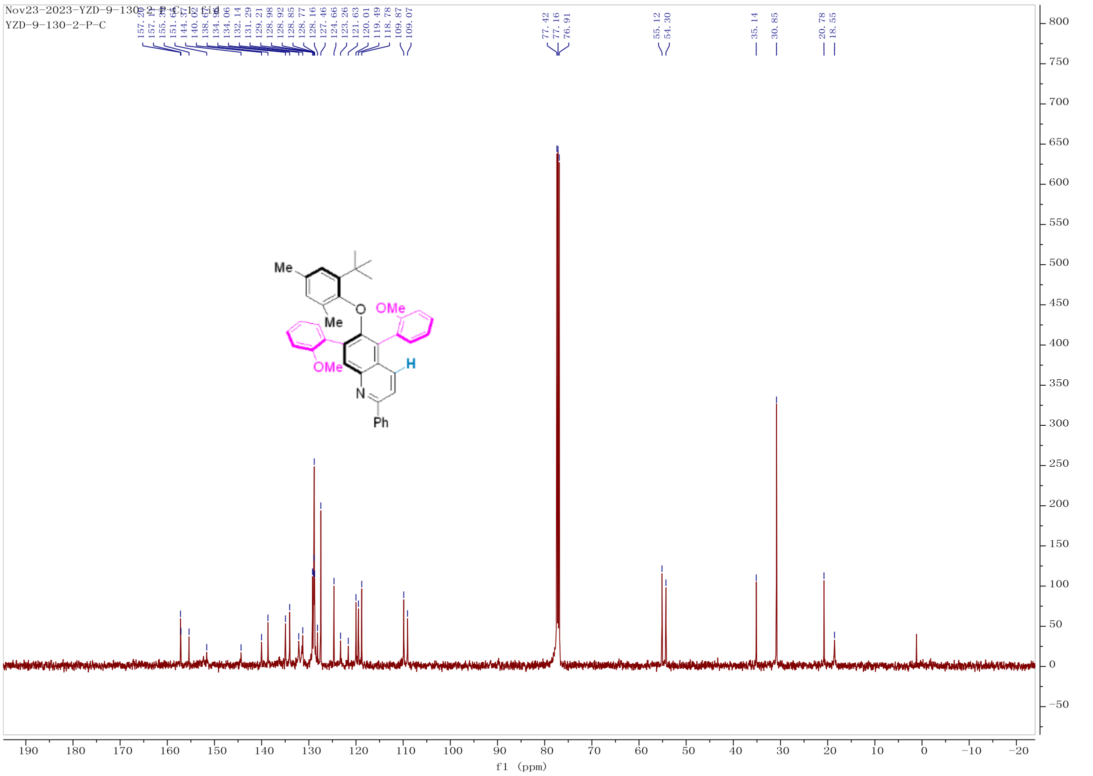

benzyl-(*R*)-(6-(2-(tert-butyl)-4,6-dimethylphenoxy)-5,7-bis(2-fluorophenyl)-2-phenyl quinolin-4-yl)carbamate (**4z**)

4z: ^{1}H NMR (500 MHz, Chloroform-*d*)

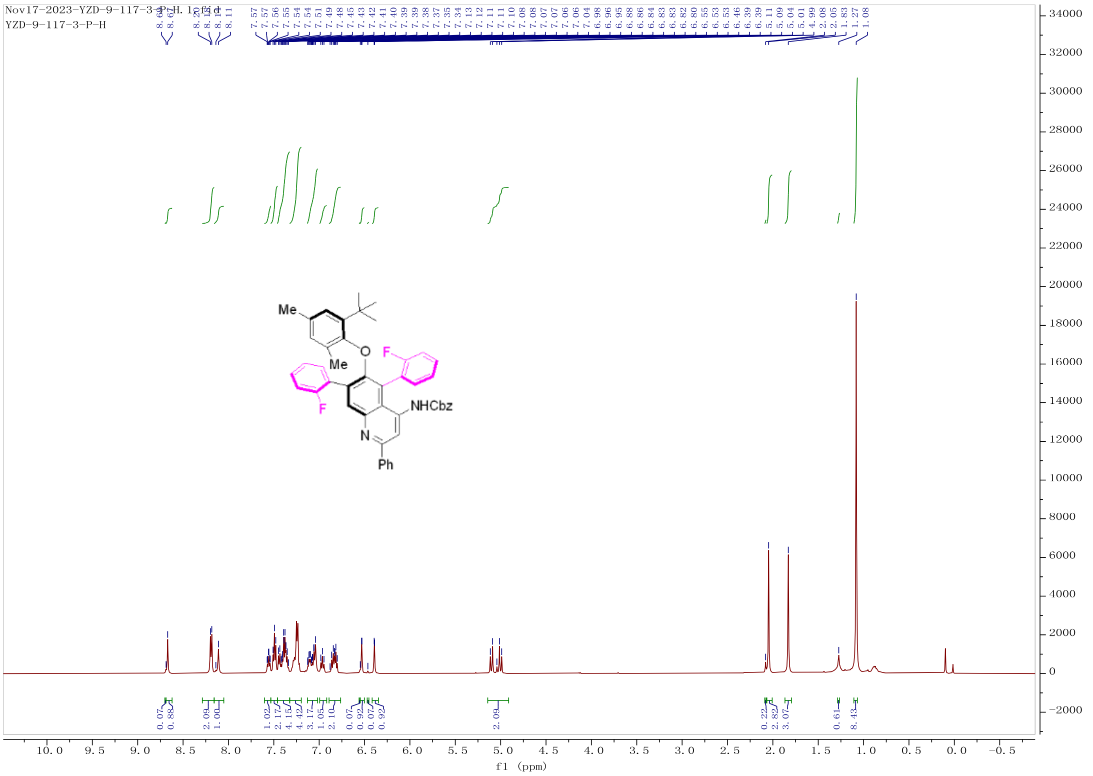

4z: ^{13}C NMR (126 MHz, Chloroform-*d*)

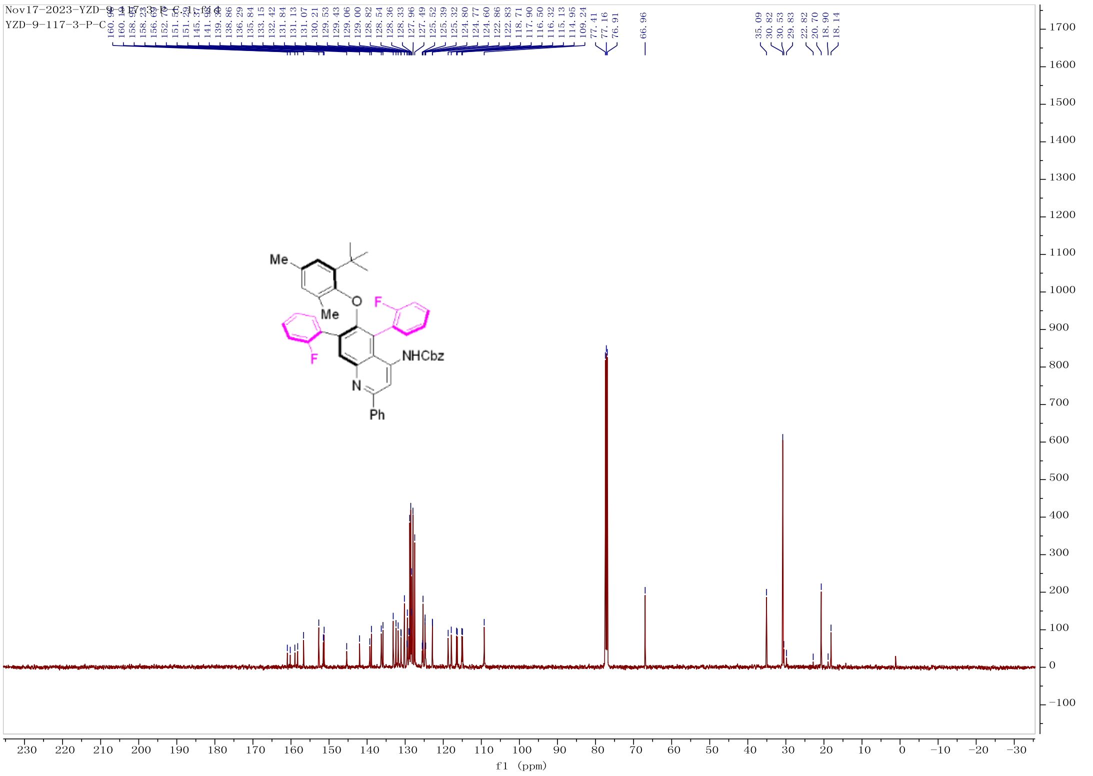

1z: ^{19}F NMR (471 MHz, Chloroform-*d*)

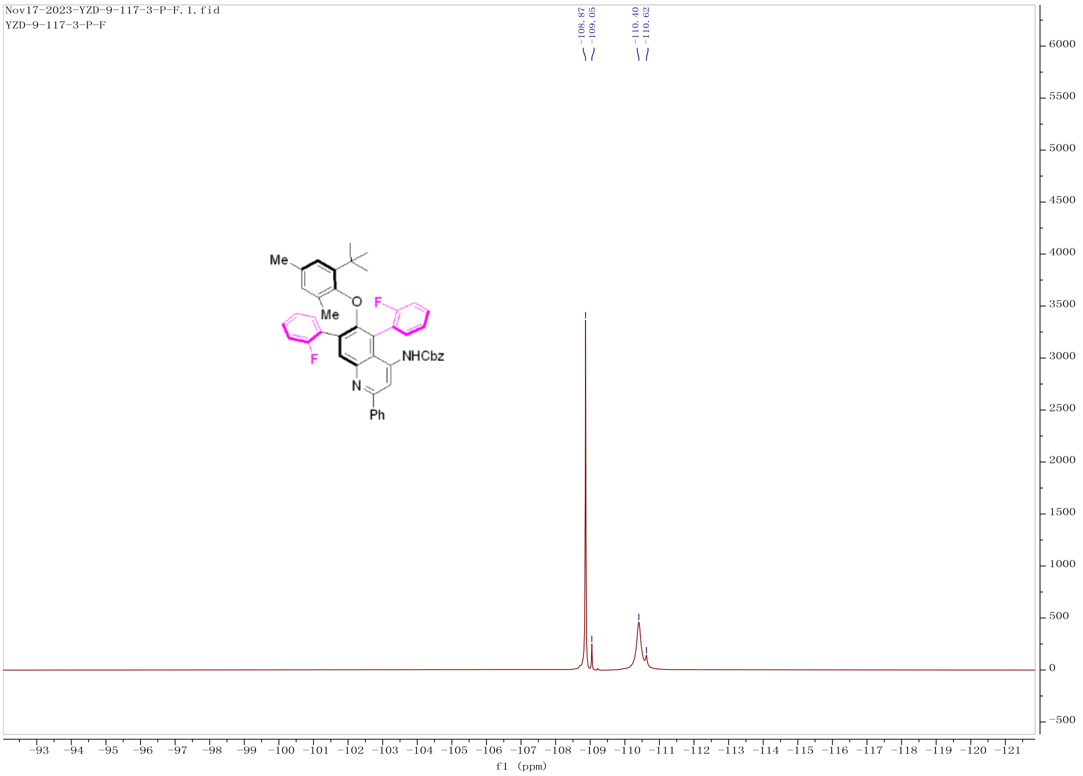

benzyl (6-(2-(tert-butyl)-4,6-dimethylphenoxy)-2-phenyl-5,7-di((E)-styryl)quinolin-4-yl)carbamate (**4aa**)

4aa: ^{1}H NMR (500 MHz, Chloroform-*d*)

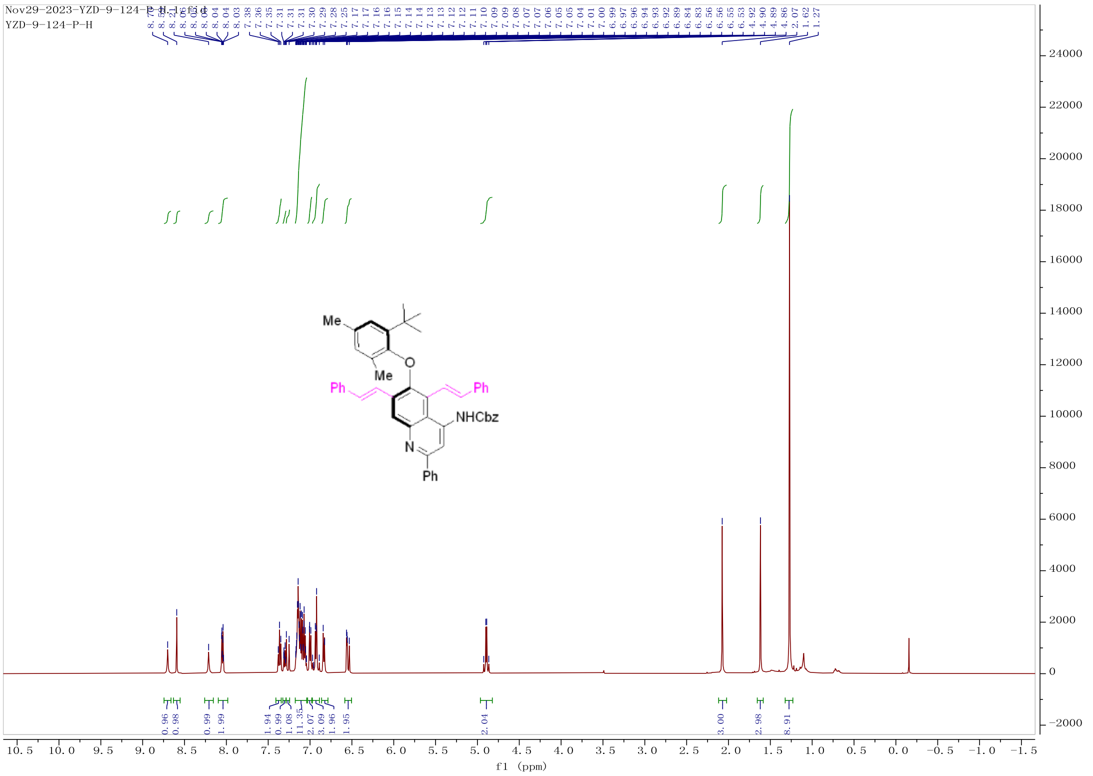

4aa: ^{13}C NMR (126 MHz, Chloroform-*d*)

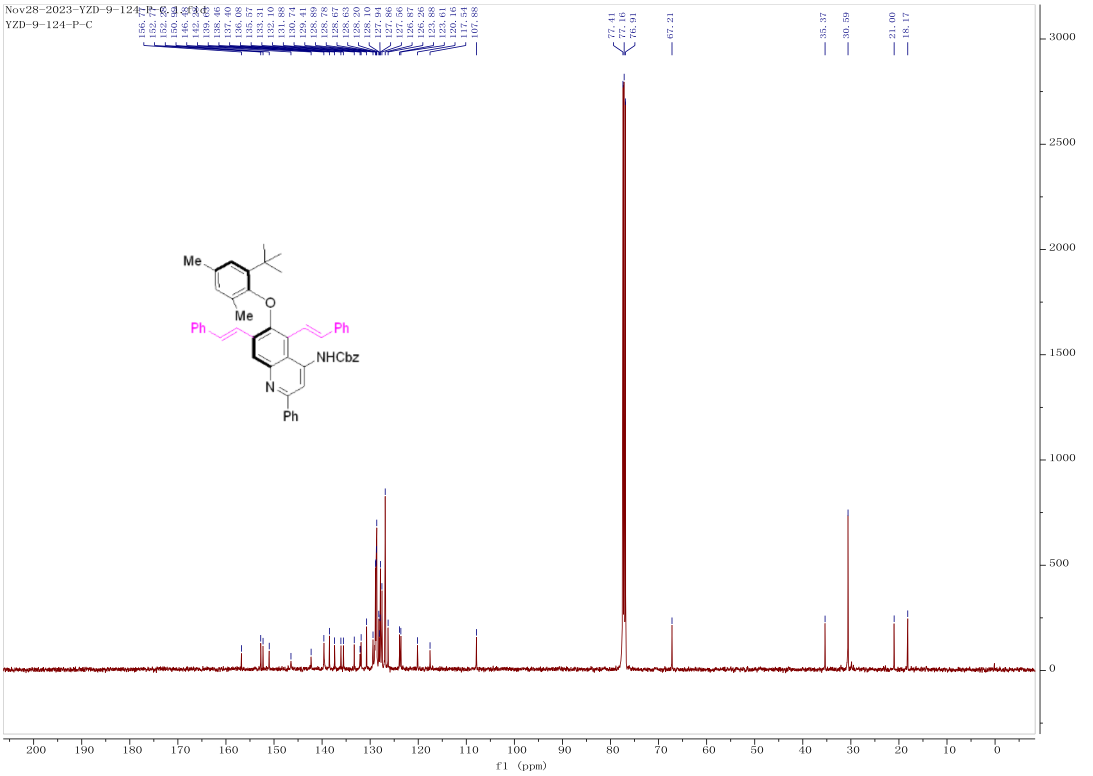

benzyl (5,7-dibromo-6-(2-(tert-butyl)-4,6-dimethylphenoxy)-2-phenylquinolin-4-yl)carbamate (**4ab**)

4ab: ^{1}H NMR (500 MHz, Chloroform-*d*)

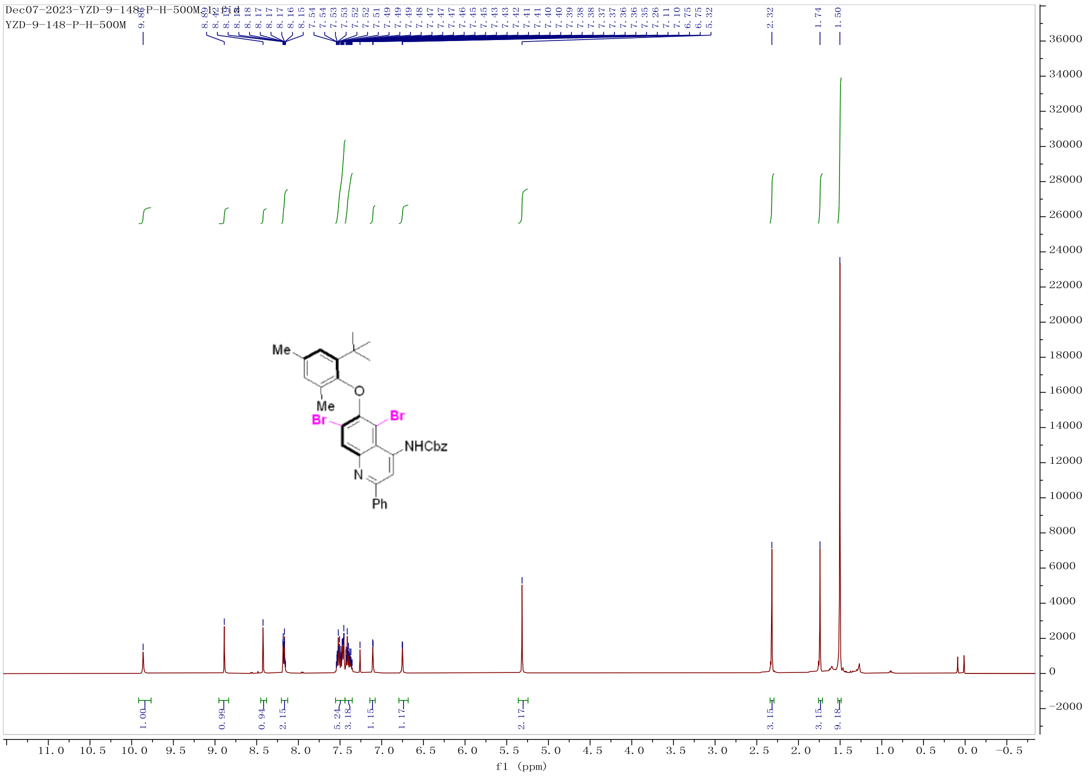

4ab: ^{13}C NMR (126 MHz, Chloroform-*d*)

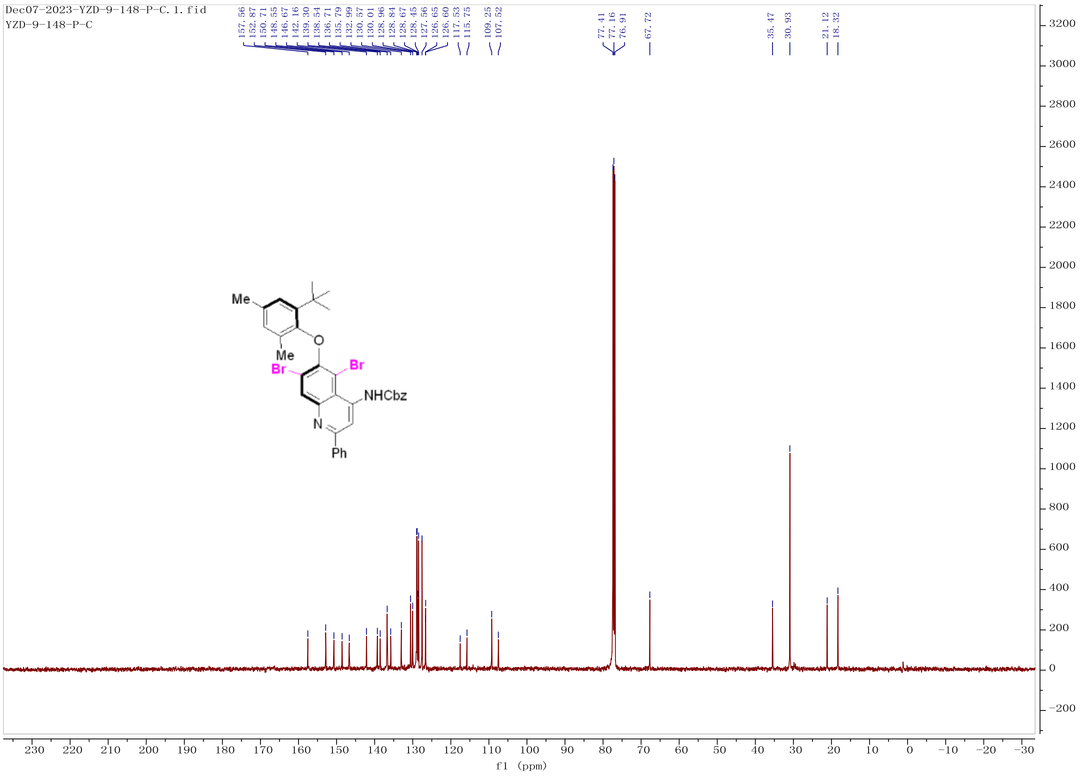

benzyl (6-(2-cyclohexyl-4,6-dimethylphenoxy)-2,5,7-triphenylquinolin-4-yl)carbamate (**4ac**)

4ac: ^{1}H NMR (500 MHz, Chloroform-*d*)

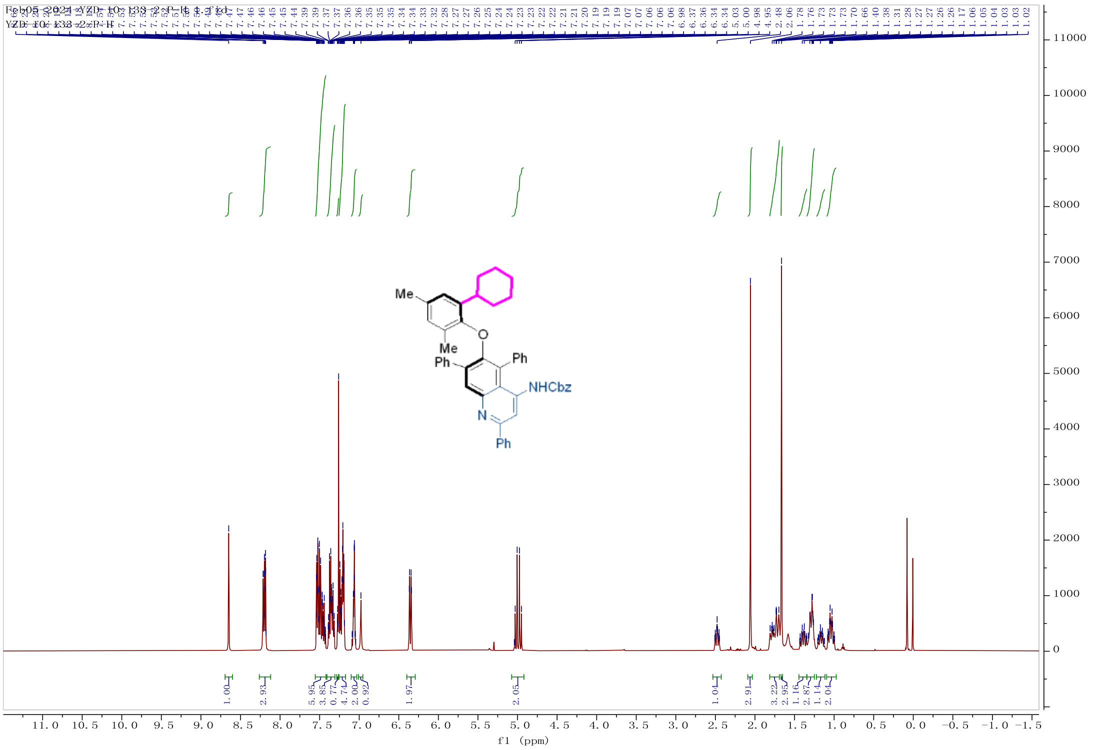

4ac: ^{13}C NMR (126 MHz, Chloroform-*d*)

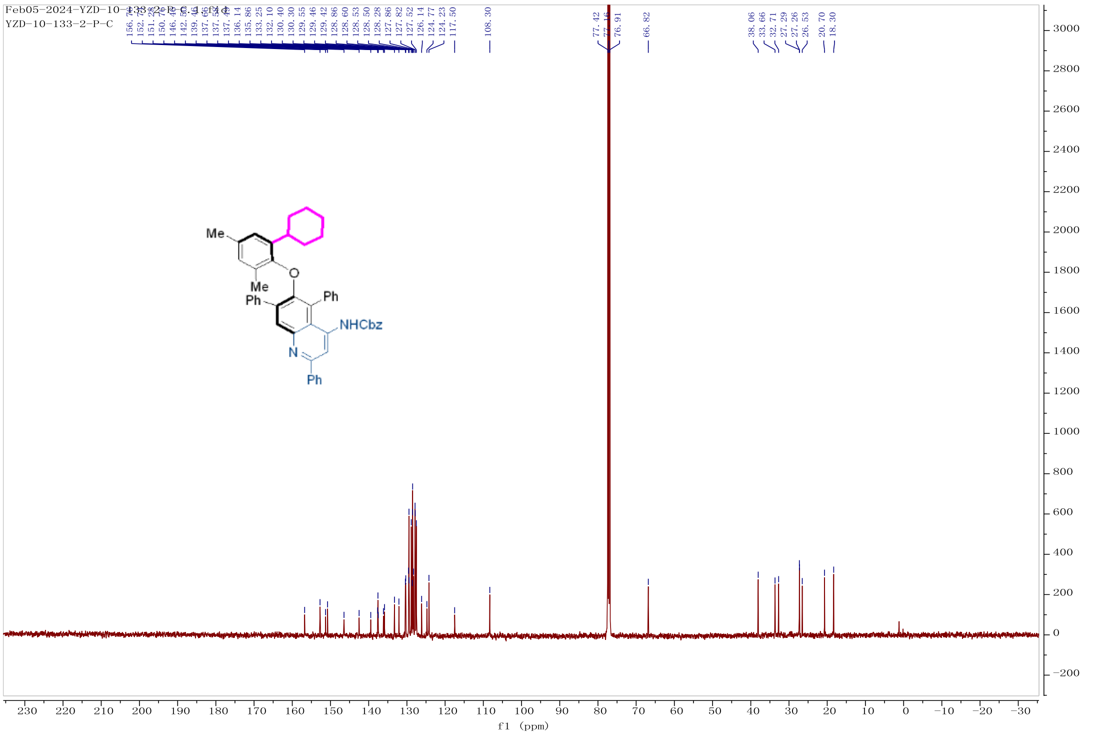

benzyl ((3*R*,4*R*)-6-(2-(tert-butyl)-4,6-dimethylphenoxy)-3-iodo-5,7-dimethyl-2-phenyl-3,4-dihydroquinolin-4-yl)carbamate (**5a’**)

5a’: ^{1}H NMR (500 MHz, Chloroform-*d*)

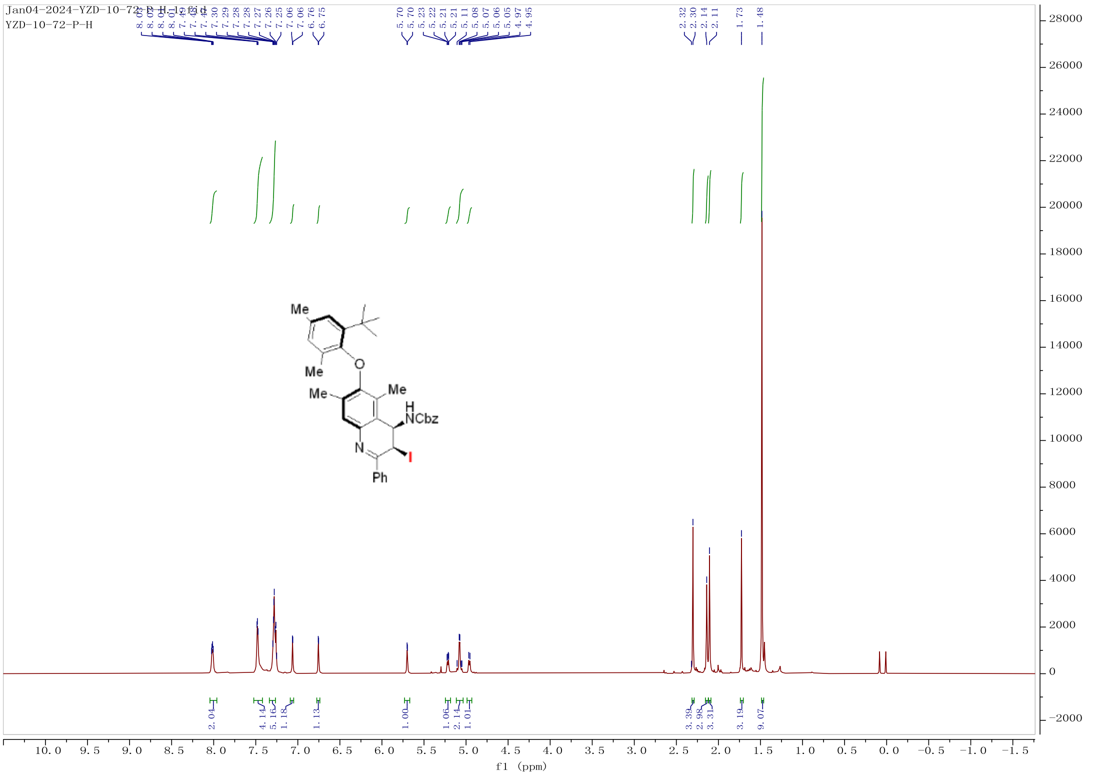

5a’: ^{13}C NMR (126 MHz, Chloroform-*d*)

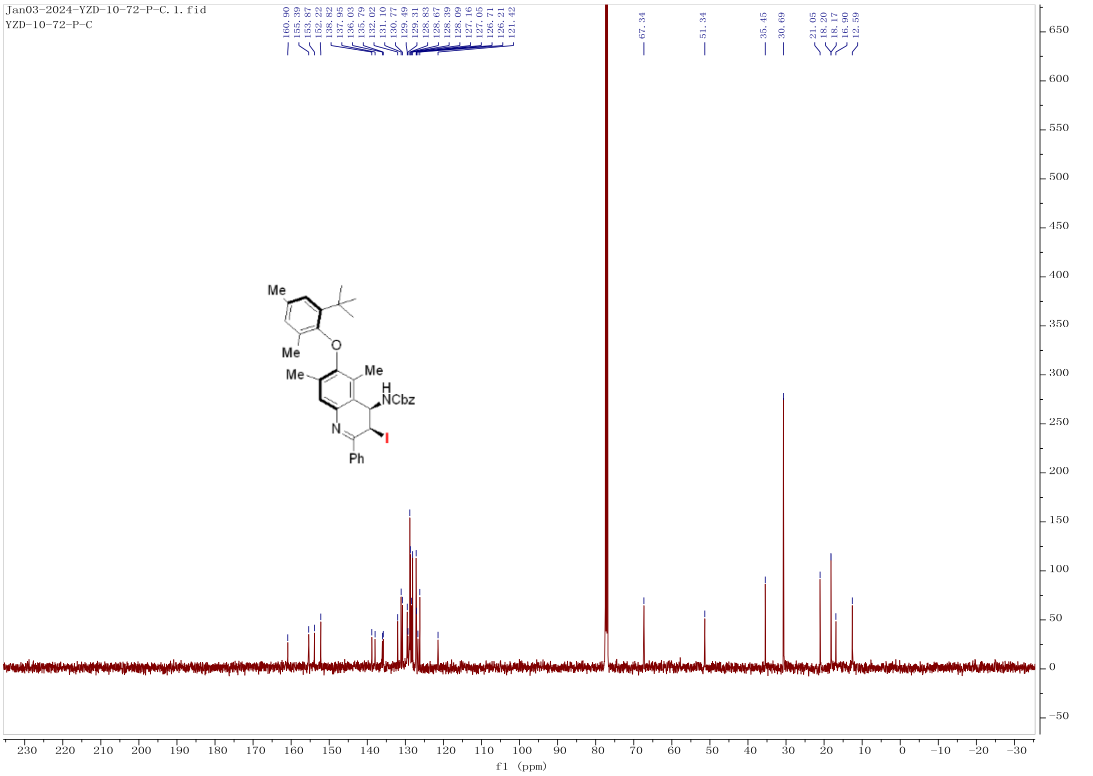

6-(2-(tert-butyl)-4,6-dimethylphenoxy)-5,7-dimethyl-2-phenylquinoline (**5a**)

5a: ^{1}H NMR (500 MHz, Chloroform-*d*)

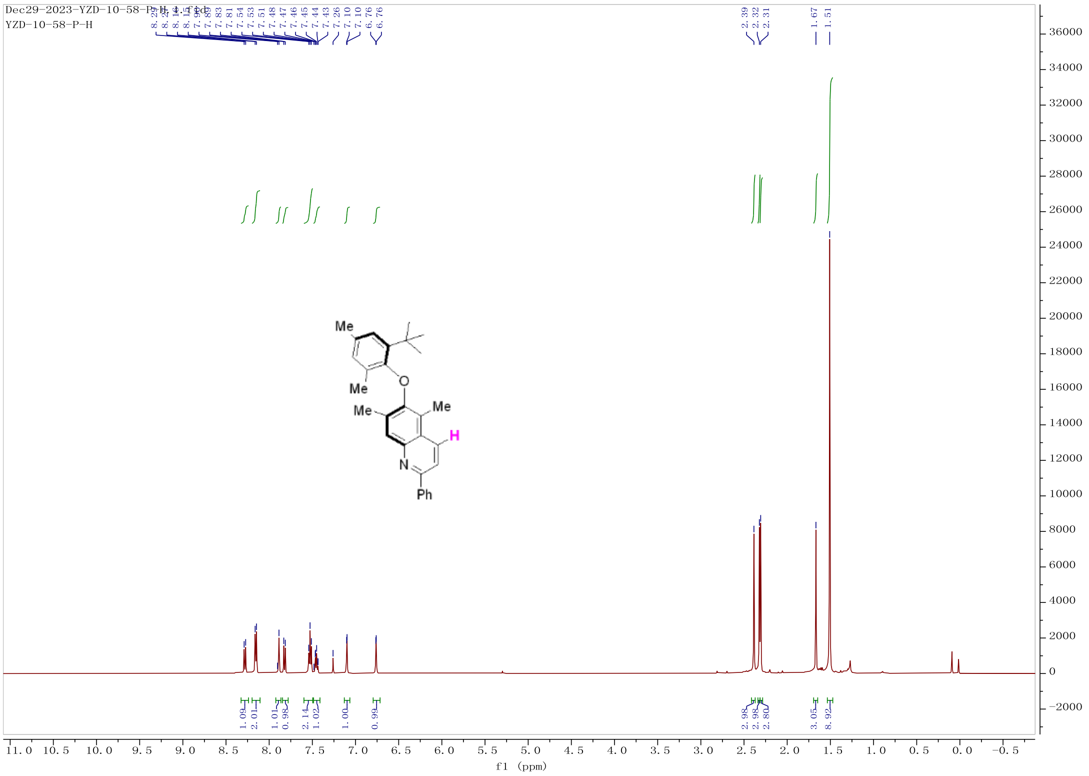

5a: ^{13}C NMR (126 MHz, Chloroform-*d*)

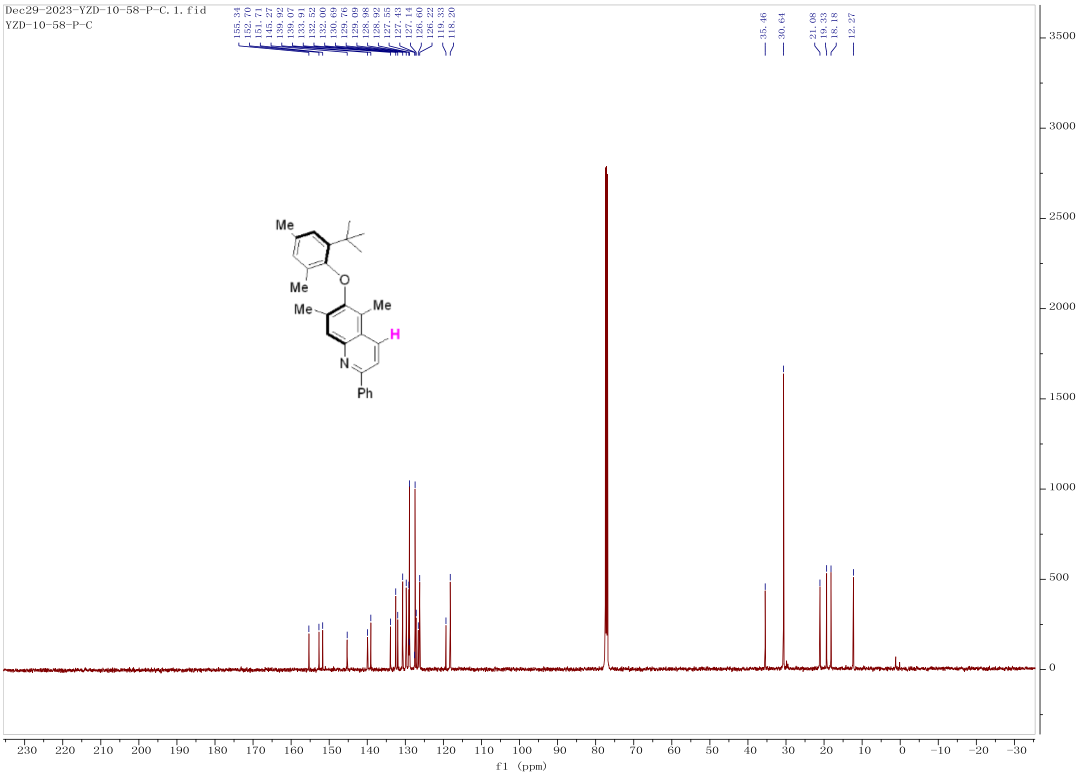

benzyl ((2*S*,4*S*)-8-bromo-6-(2-(tert-butyl)-4,6-dimethylphenoxy)-5,7-dimethyl-2-phenyl-1,2,3,4-tetrahydroquinolin-4-yl)carbamate (**6a**)

6a: ^{1}H NMR (500 MHz, Chloroform-*d*)

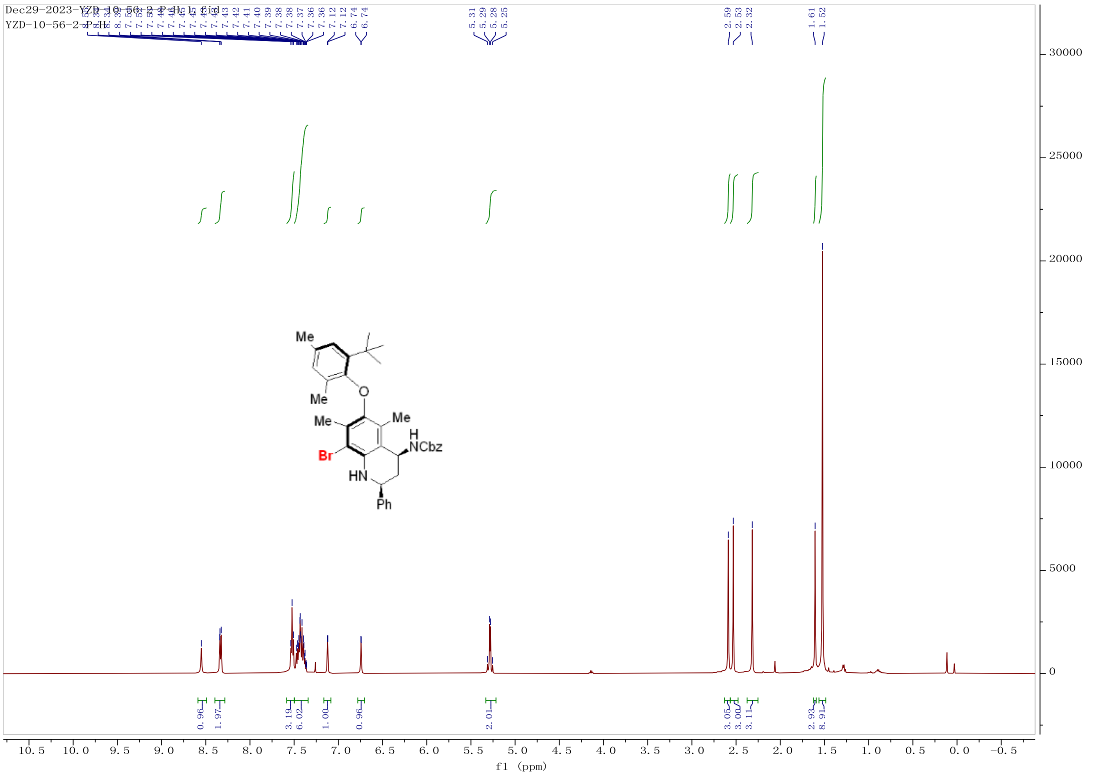

6a: ^{13}C NMR (126 MHz, Chloroform-*d*)

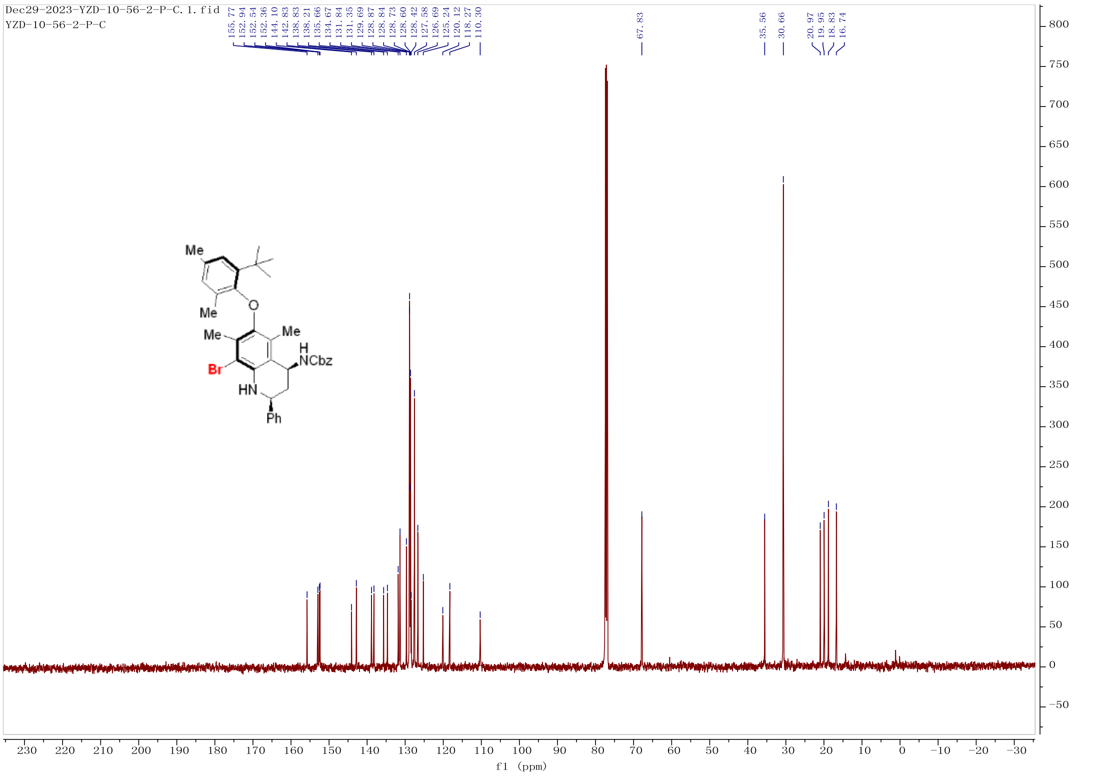

benzyl (8-bromo-6-(2-(tert-butyl)-4,6-dimethylphenoxy)-5,7-dimethyl-2-phenylquinolin-4-yl)carbamate (**7a**)

7a: ^{1}H NMR (500 MHz, Chloroform-*d*)

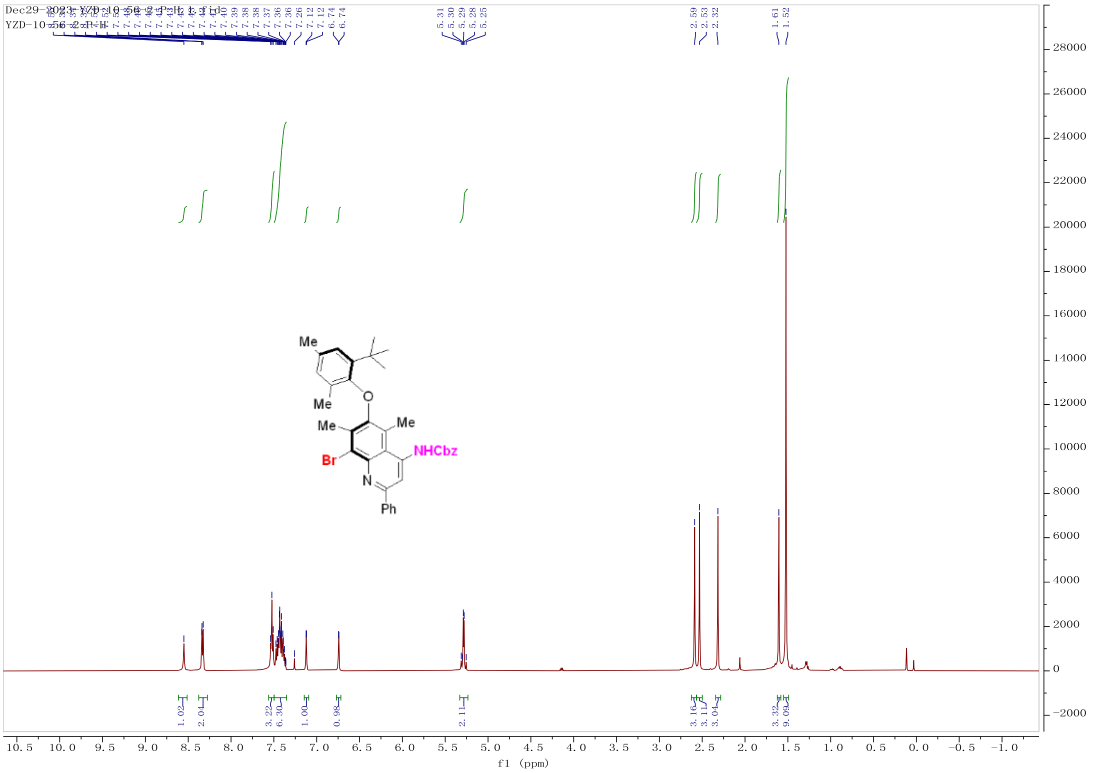

7a: ^{13}C NMR (126 MHz, Chloroform-*d*)

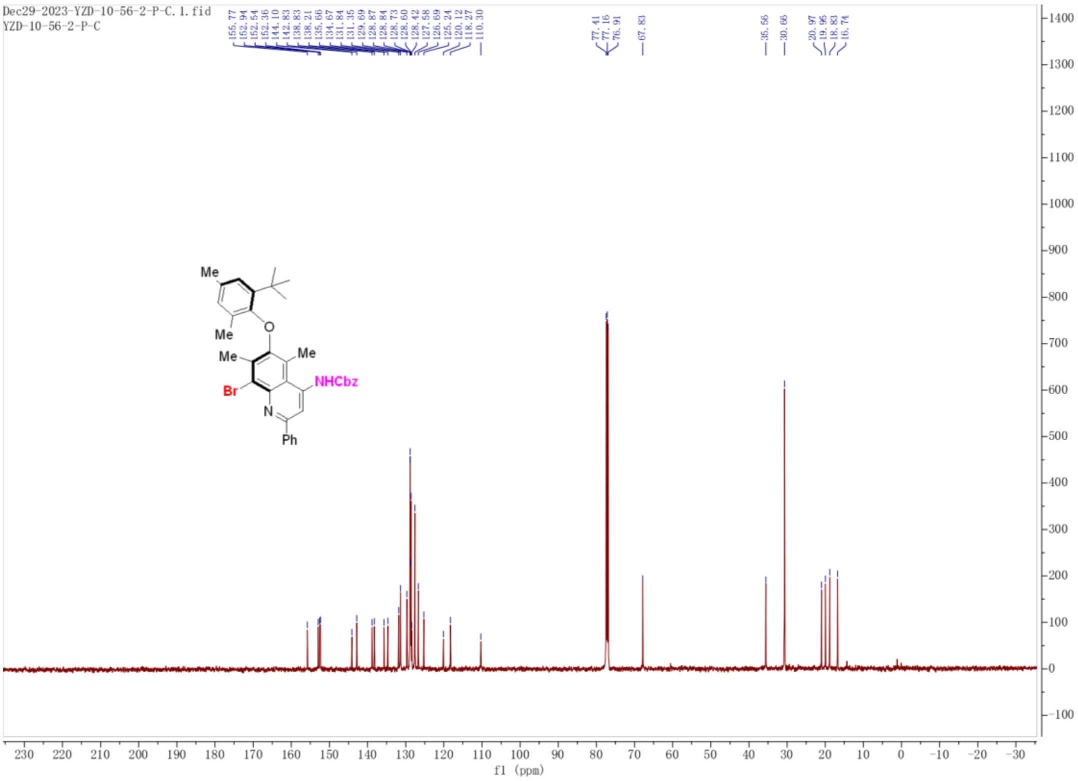

8-bromo-6-(2-(tert-butyl)-4,6-dimethylphenoxy)-5,7-dimethyl-2-phenylquinoline (**8a**)

8a: ^{1}H NMR (400 MHz, Chloroform-*d*)

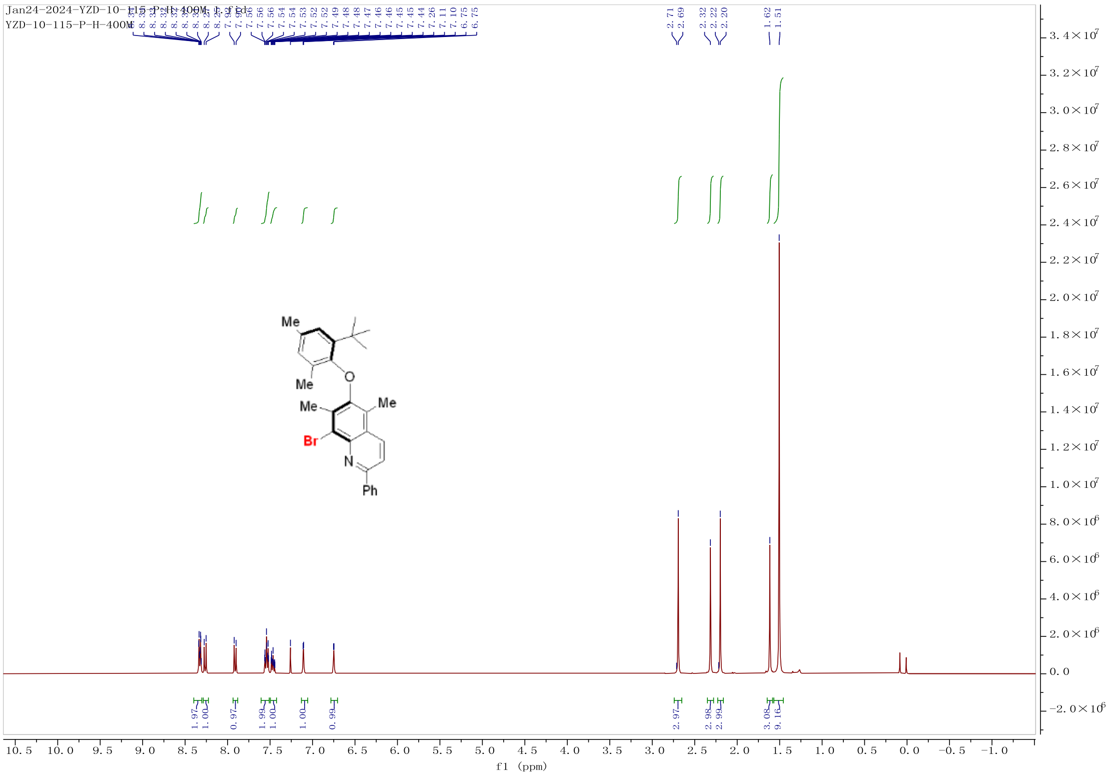

8a: ^{13}C NMR (101 MHz, Chloroform-*d*)

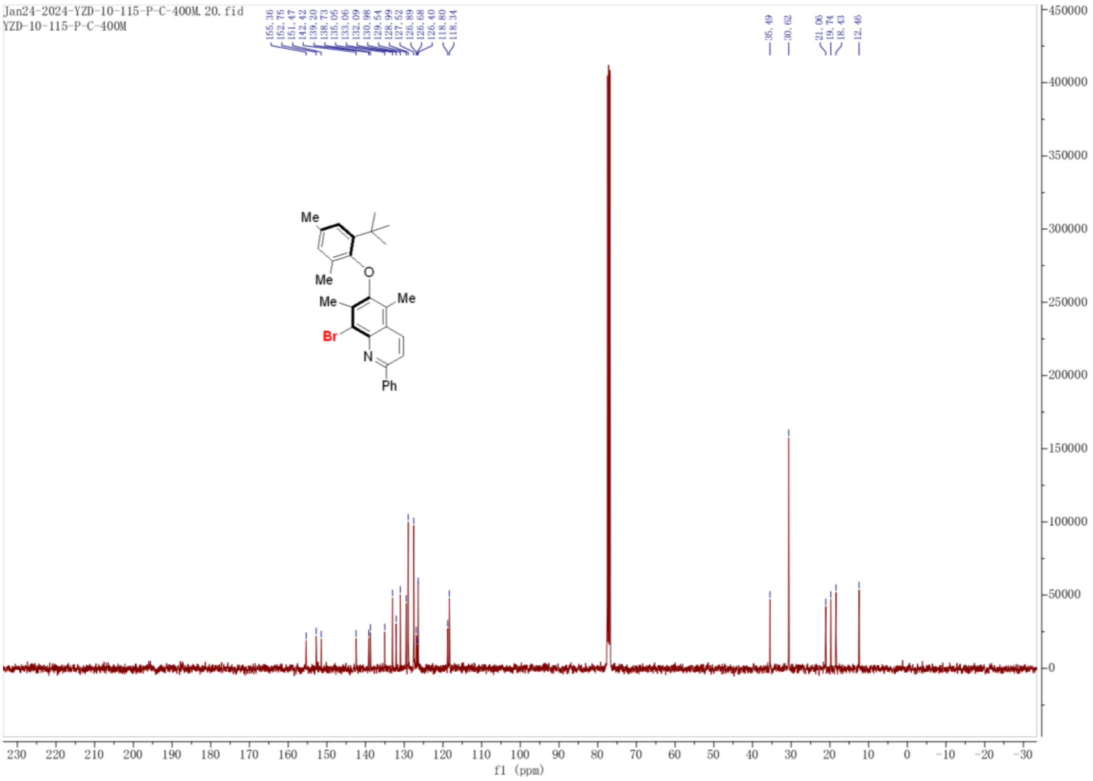

3,8-dibromo-6-(2-(tert-butyl)-4,6-dimethylphenoxy)-5,7-dimethyl-2-phenylquinoline (**9a**)

9a: ^{1}H NMR (500 MHz, Chloroform-*d*)

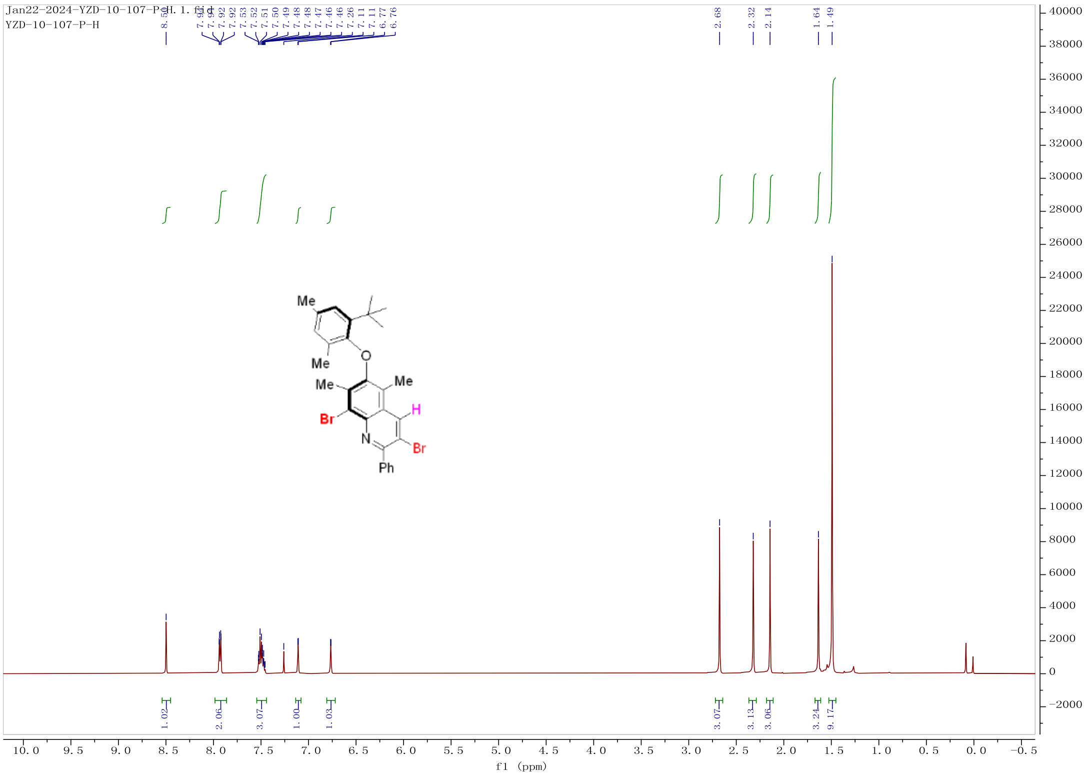

9a: ^{13}C NMR (126 MHz, Chloroform-*d*)

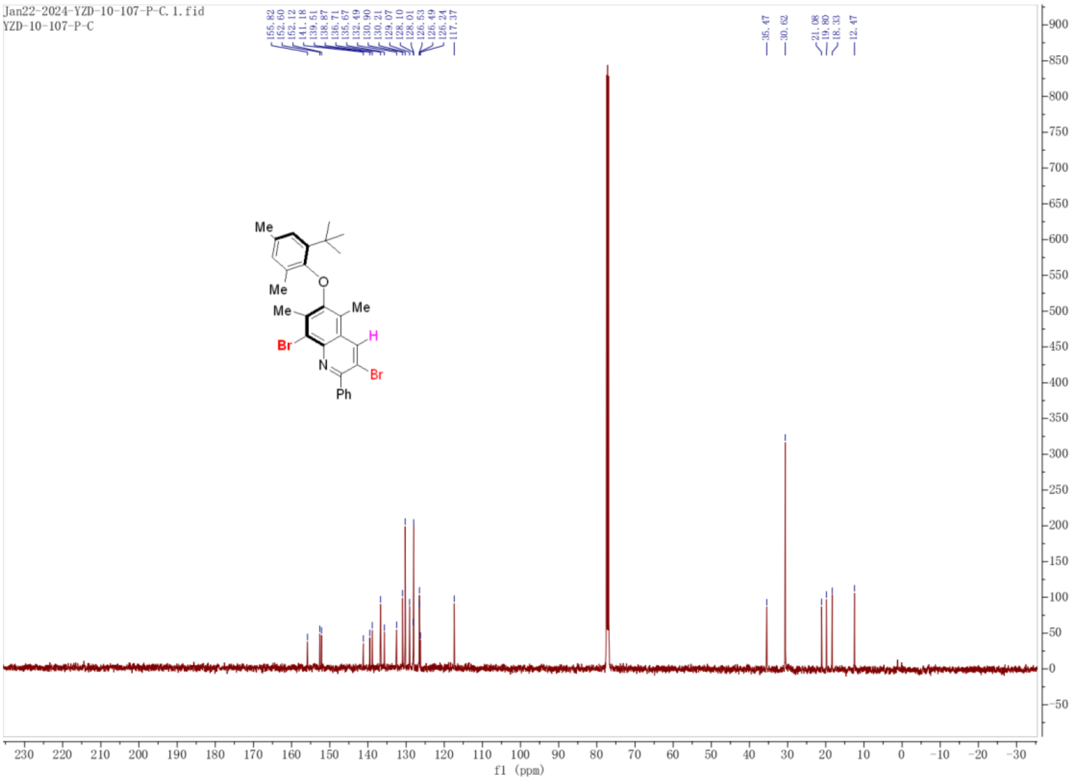

6-(2-(tert-butyl)-4,6-dimethylphenoxy)-5,7-dimethyl-2-phenylquinolin-4-amine (**10a**)

10a: ^{1}H NMR (500 MHz, Chloroform-*d*)

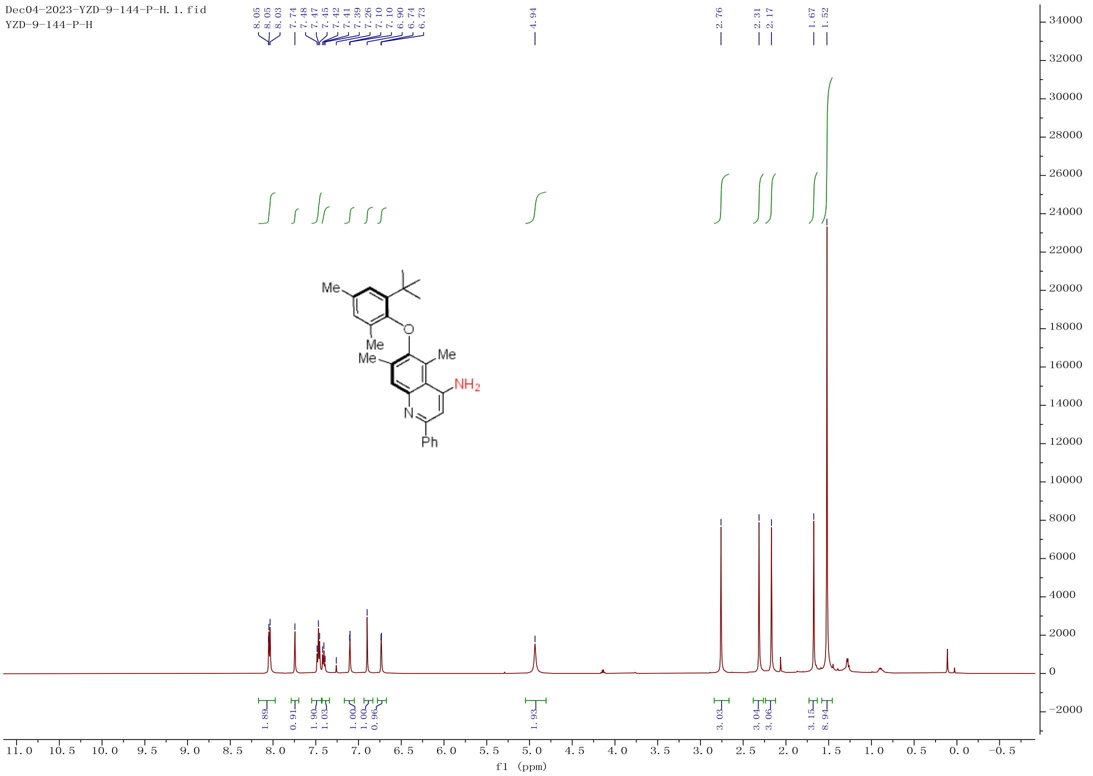

10a: ^{13}C NMR (126 MHz, Chloroform-*d*)

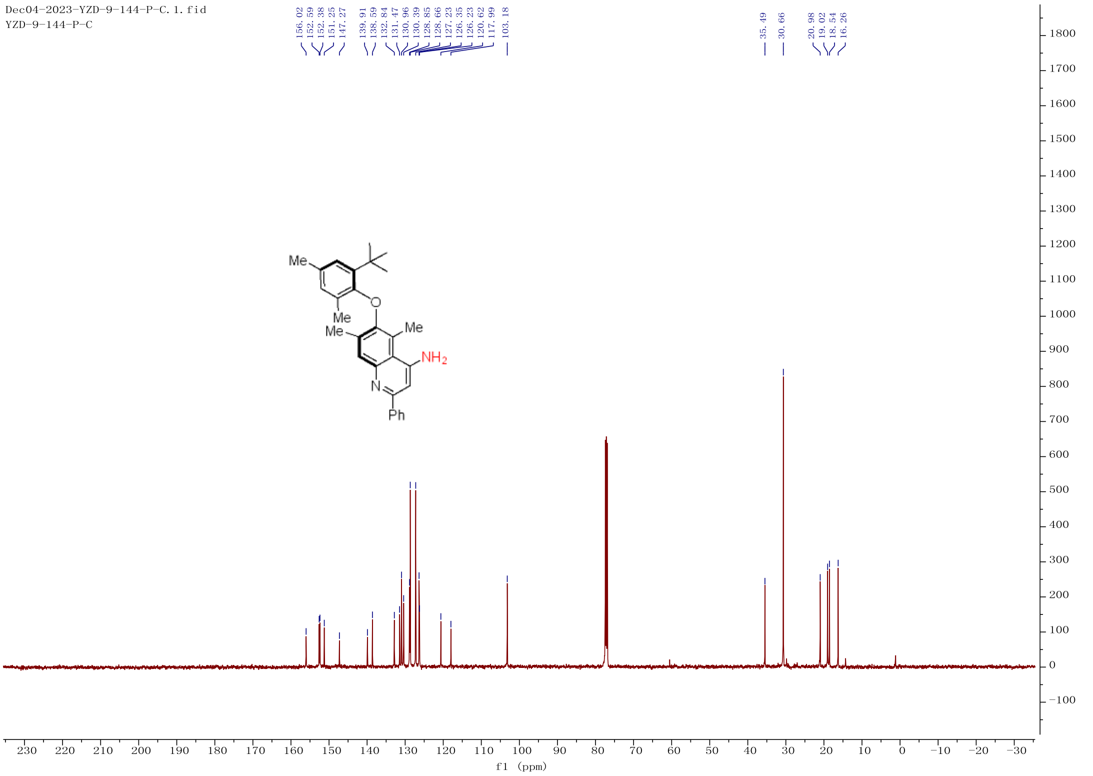

4-bromo-6-(2-(tert-butyl)-4,6-dimethylphenoxy)-5,7-dimethyl-2-phenylquinoline (**11 a**)

11a: ^{1}H NMR (500 MHz, Chloroform-*d*)

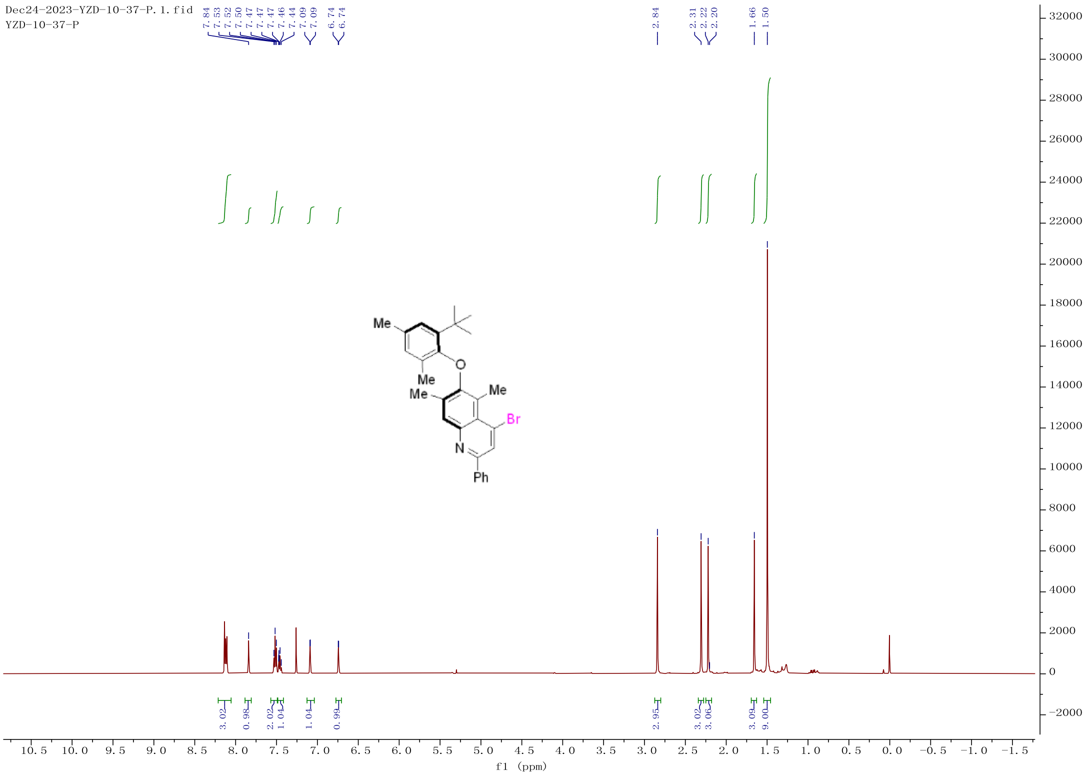

11a: ^{13}C NMR (126 MHz, Chloroform-*d*)

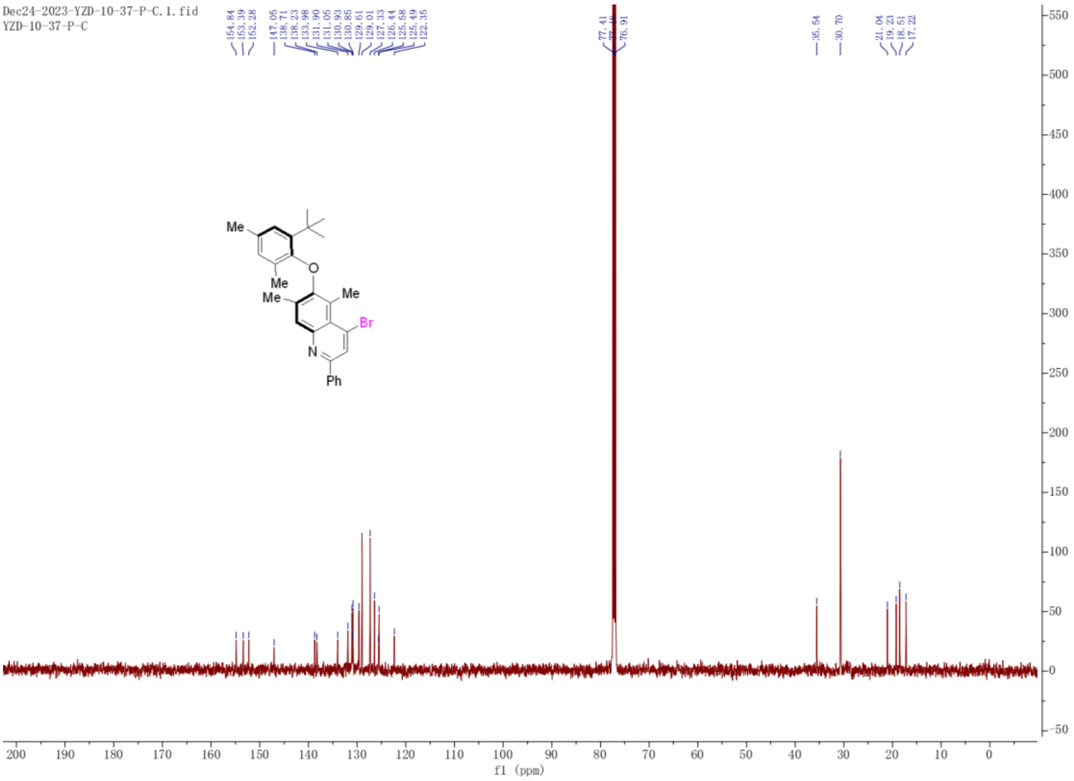

6-(2-(tert-butyl)-4,6-dimethylphenoxy)-4-iodo-5,7-dimethyl-2-phenylquinoline (**12a**)

12a: ^{1}H NMR (500 MHz, Chloroform-*d*)

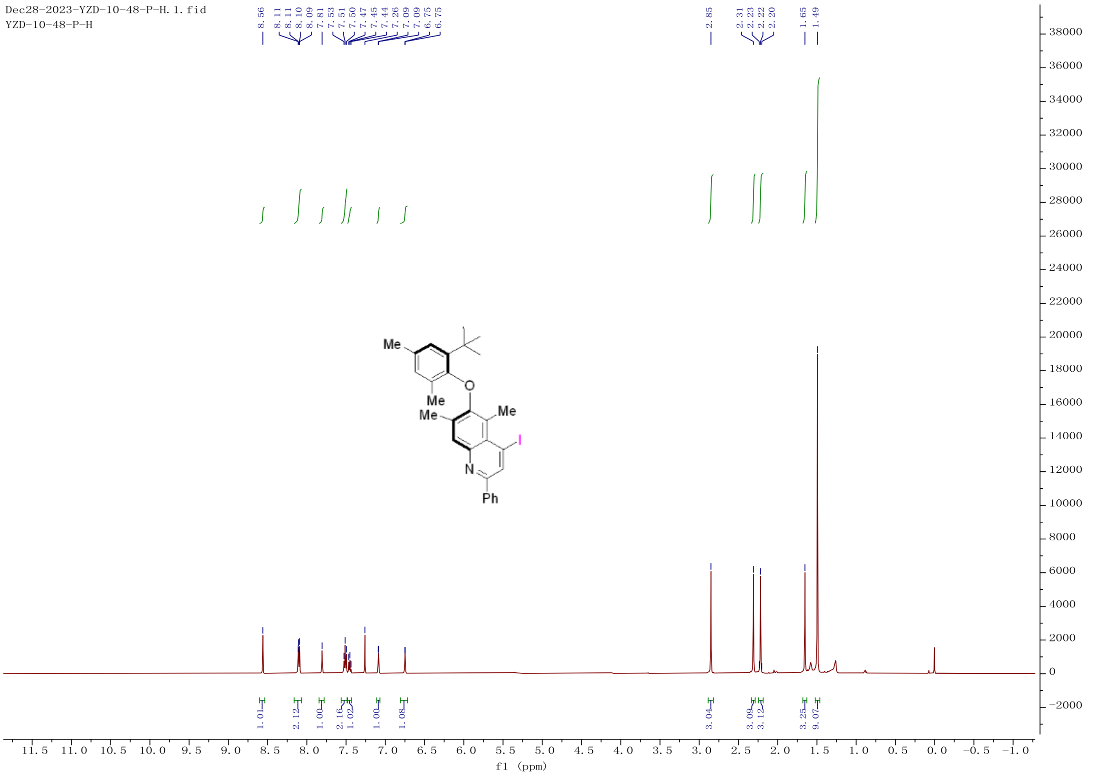

12a: ^{13}C NMR (126 MHz, Chloroform-*d*)

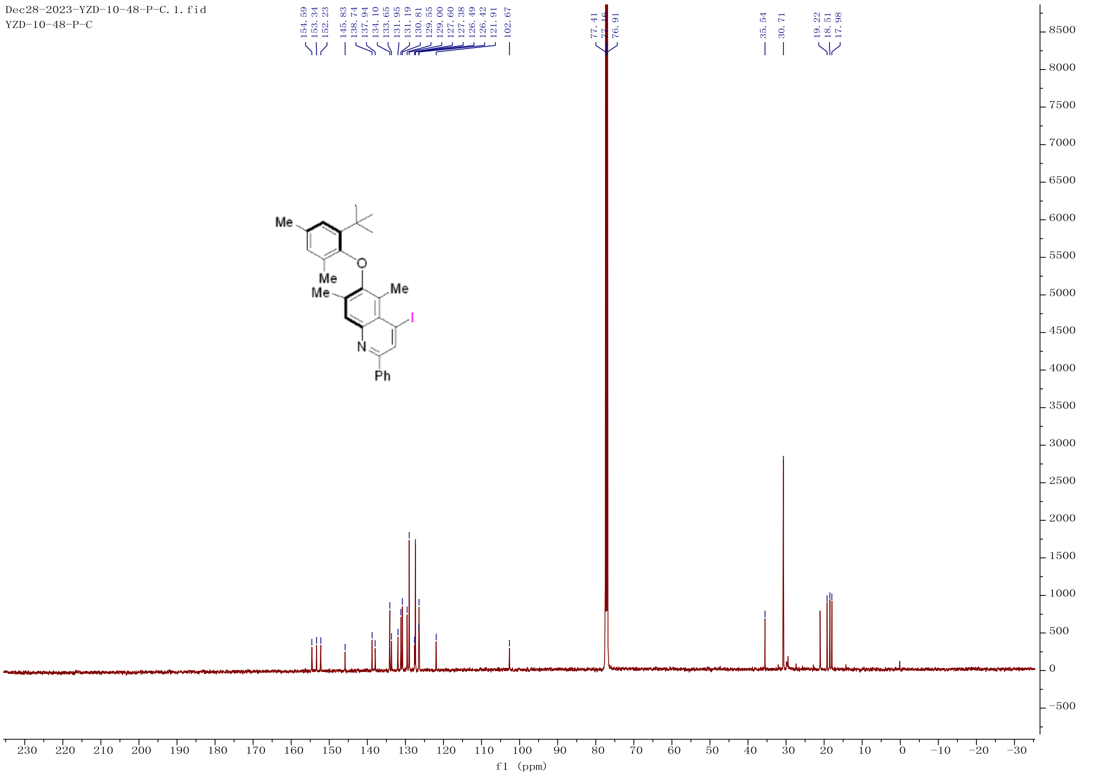

3-bromo-6-(2-(tert-butyl)-4,6-dimethylphenoxy)-5,7-dimethyl-2-phenylquinolin-4-amine (**13a**)

13a: ^{1}H NMR (500 MHz, Chloroform-*d*)

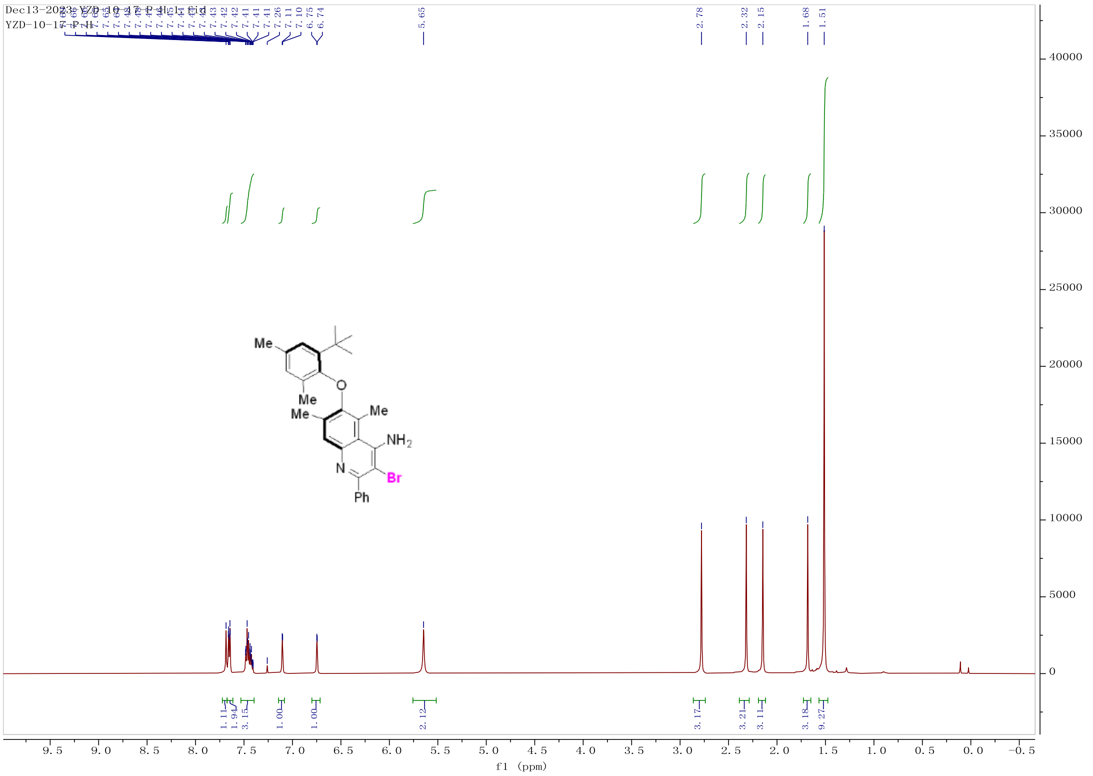

13a: ^{13}C NMR (126 MHz, Chloroform-*d*)

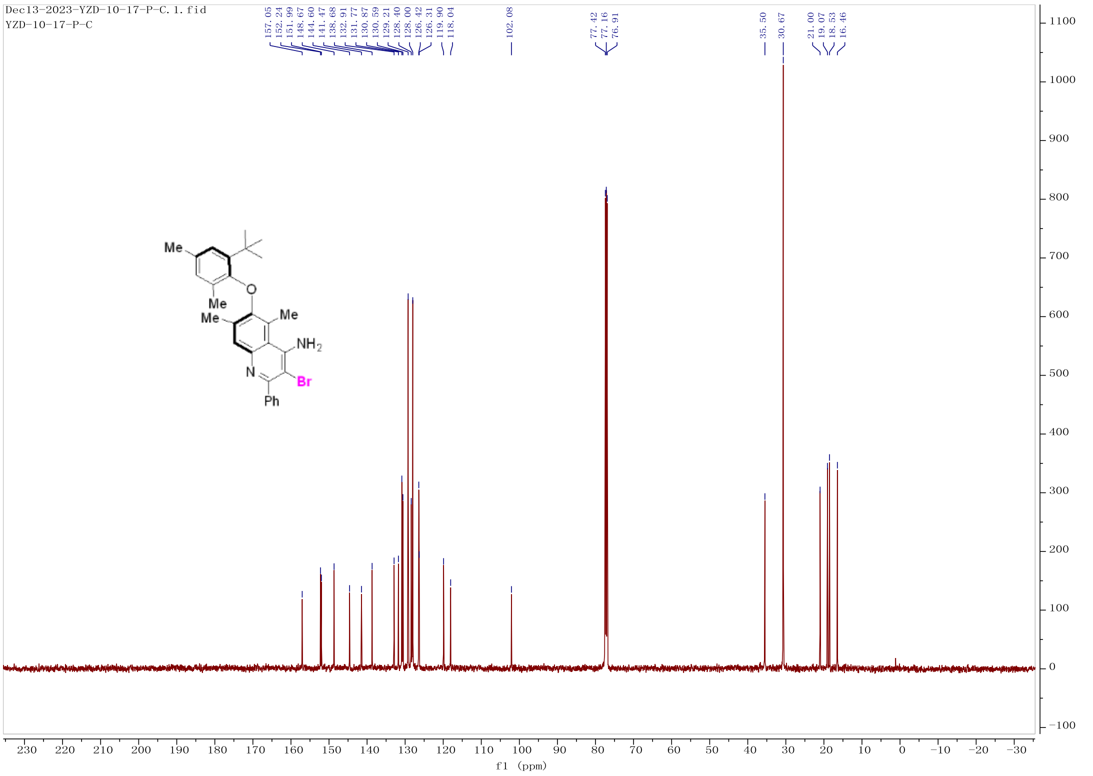

6-(2-(tert-butyl)-4,6-dimethylphenoxy)-3-iodo-5,7-dimethyl-2-phenylquinolin-4-amine (**14a**)

14a: ^{1}H NMR (500 MHz, Chloroform-*d*)

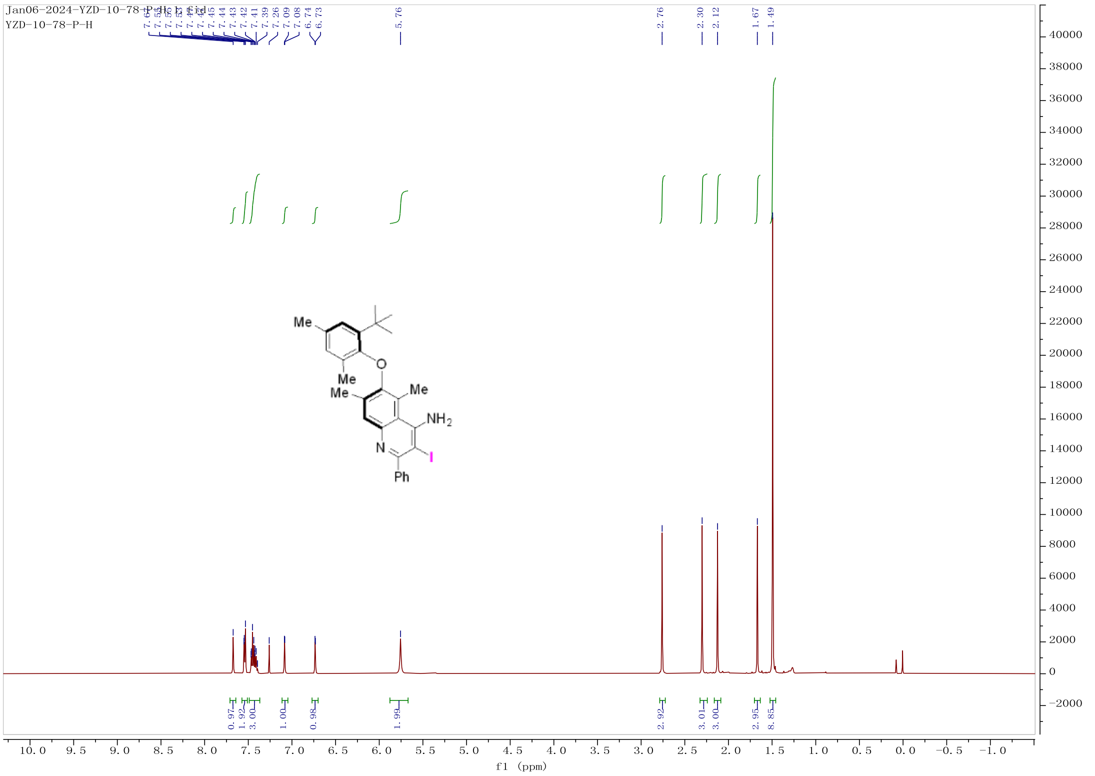

14a: ^{13}C NMR (126 MHz, Chloroform-*d*)

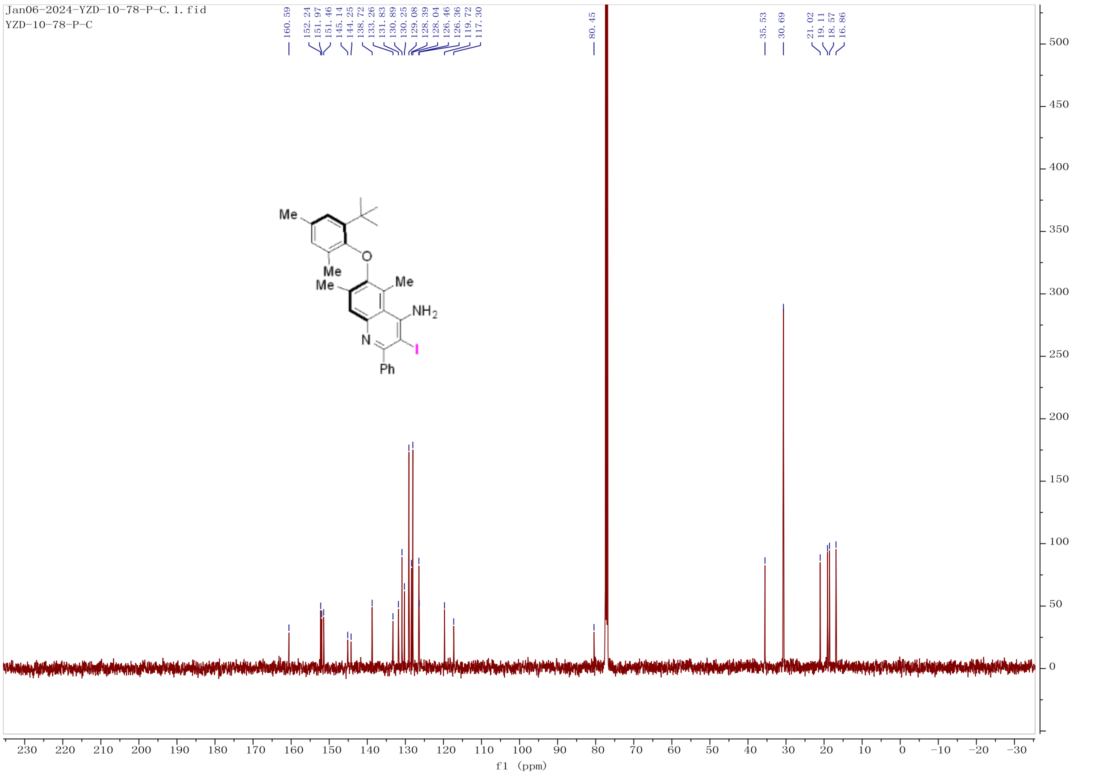

6-(2-(tert-butyl)-4,6-dimethylphenoxy)-5,7-dimethyl-2,3-diphenylquinolin-4-amine (**15a**)

15a: ^{1}H NMR (500 MHz, Chloroform-*d*)

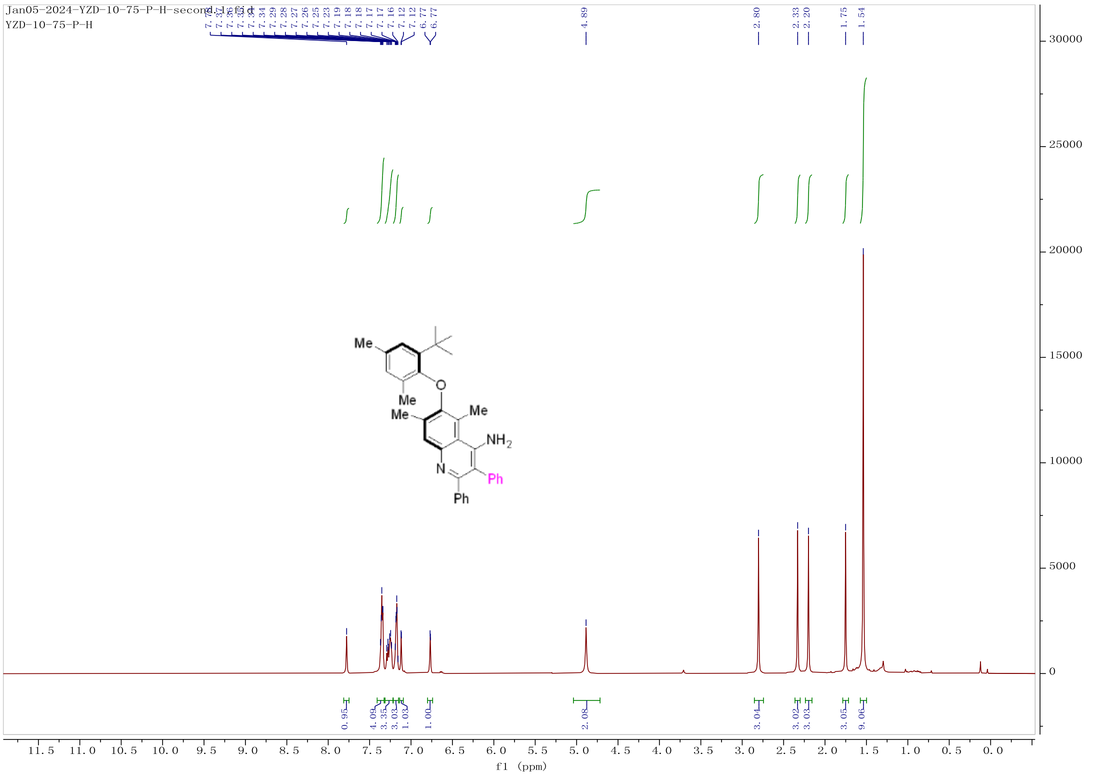

15a: ^{13}C NMR (126 MHz, Chloroform-*d*)

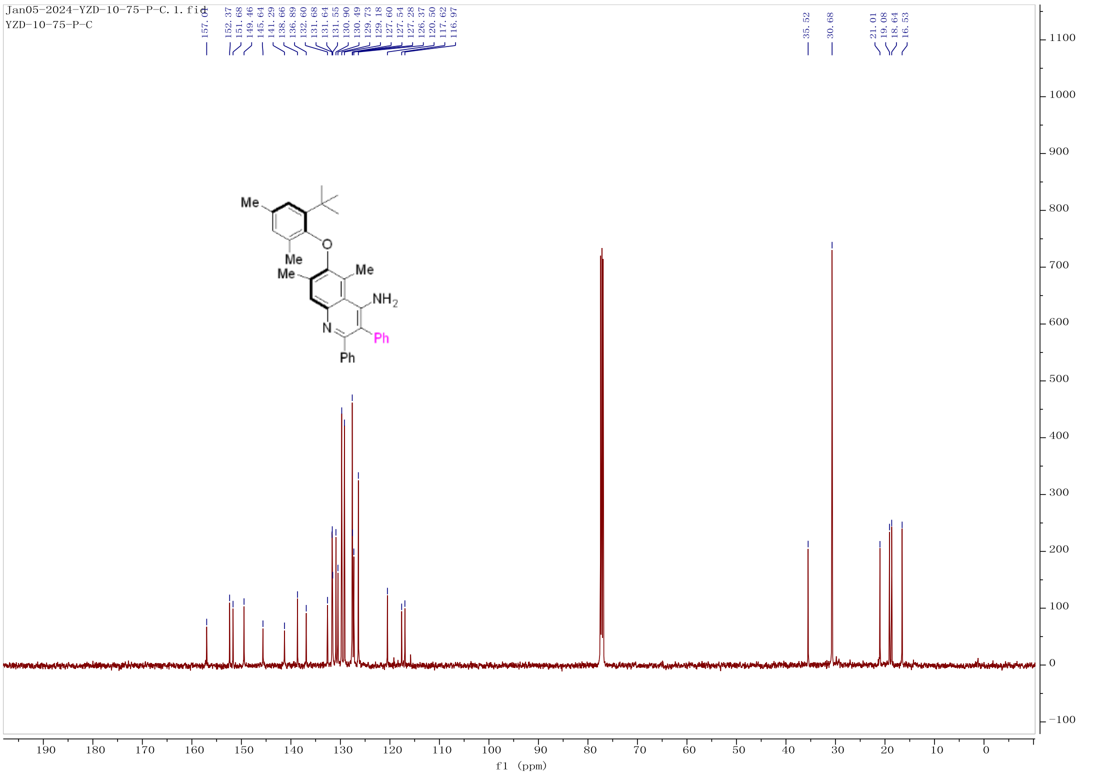

8-(2-(tert-butyl)-4,6-dimethylphenoxy)-7,9-dimethyl-4-phenyl-1H-pyrrolo[3,2-c]quinoline (**16a**)

16a: ^{1}H NMR (500 MHz, Chloroform-*d*)

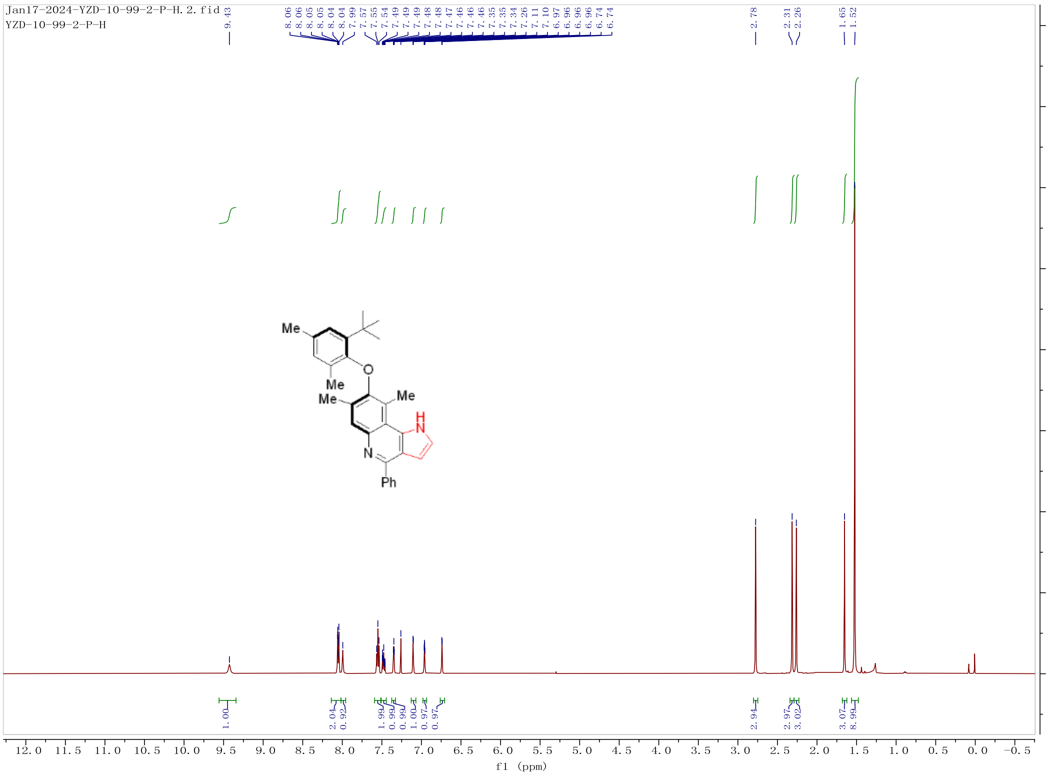

16a: ^{13}C NMR (126 MHz, Chloroform-*d*)

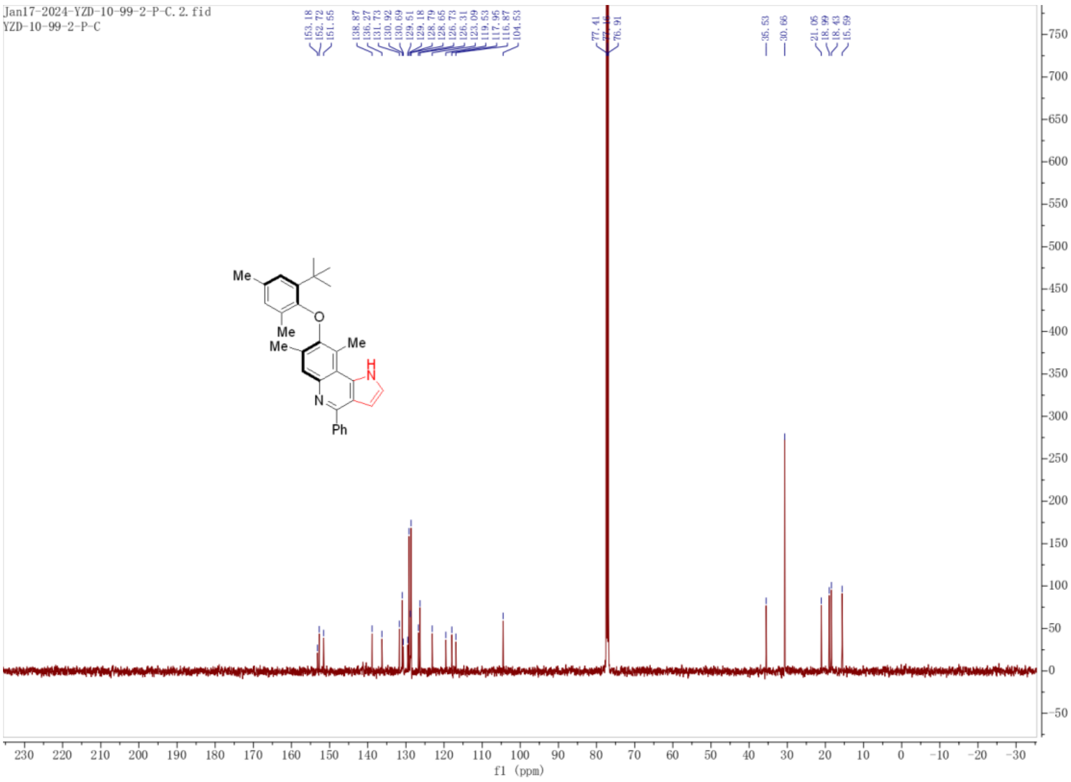

6-(2-(tert-butyl)-4,6-dimethylphenoxy)-5,7-dimethyl-2-phenyl-3-(phenylethynyl)quinolin-4-amine (**17a**)

17a: ^{1}H NMR (500 MHz, Chloroform-*d*)

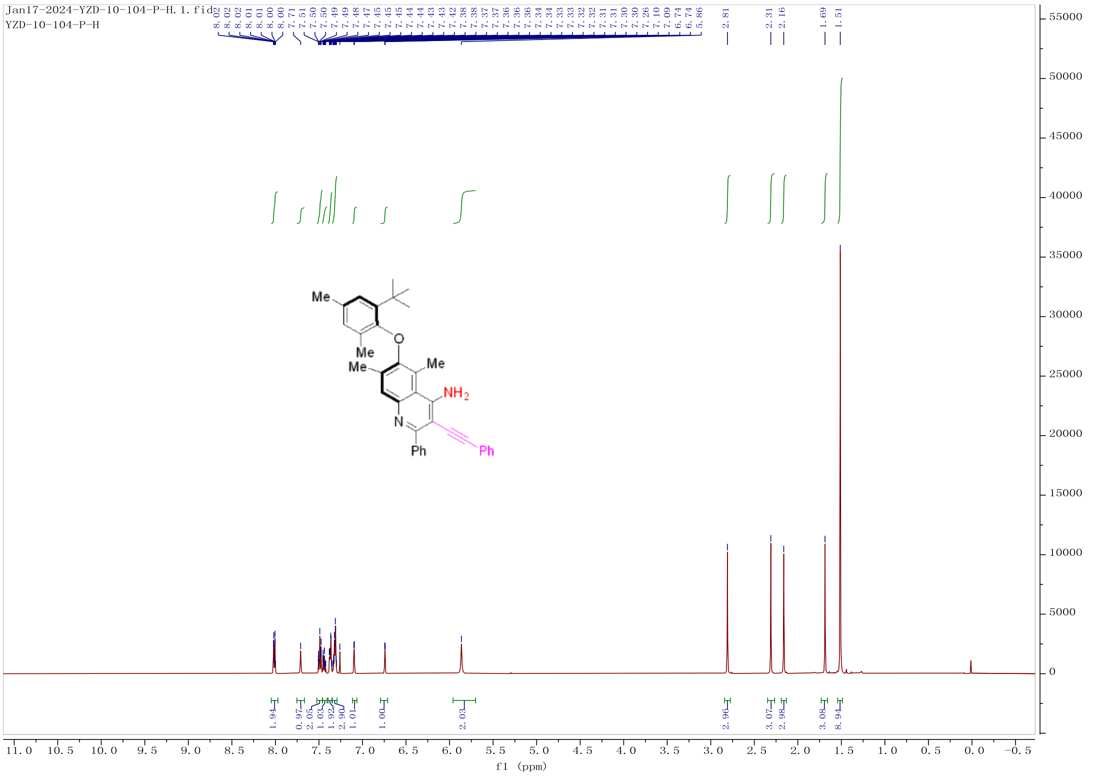

17a: ^{13}C NMR (126 MHz, Chloroform-*d*)

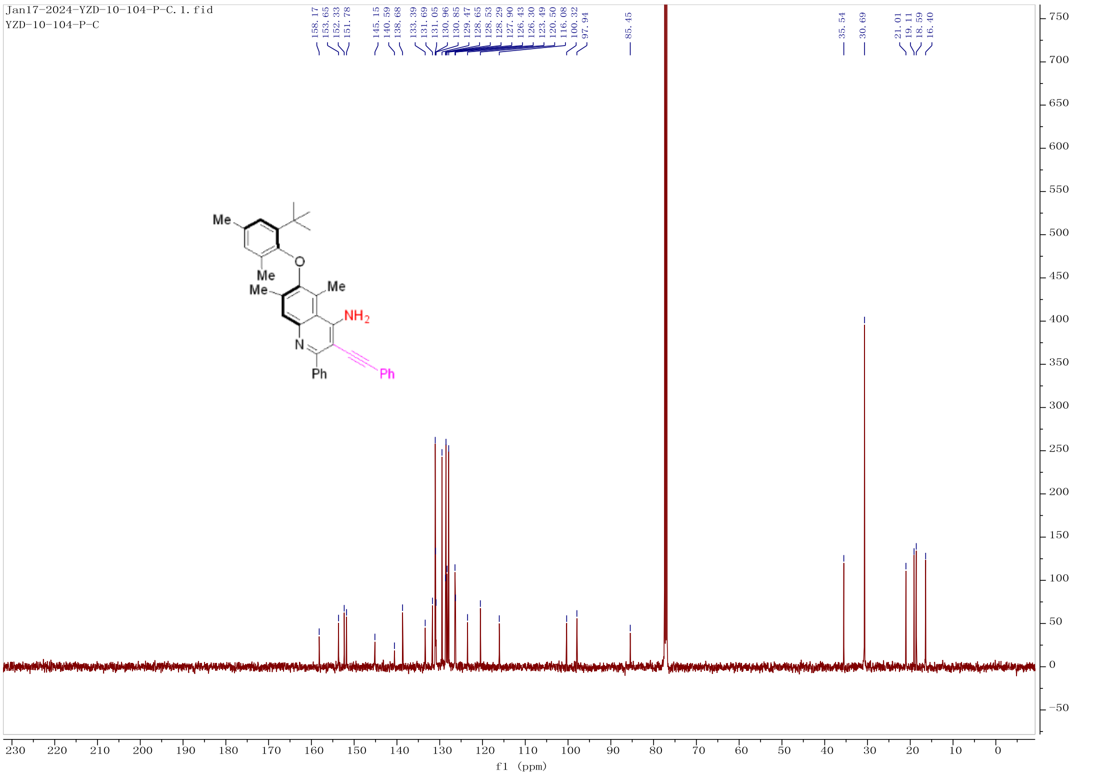

8-(2-(tert-butyl)-4,6-dimethylphenoxy)-7,9-dimethyl-2,4-diphenyl-1H-pyrrolo[3,2-c]quinoline (**18a**)

18a: ^{1}H NMR (500 MHz, Chloroform-*d*)

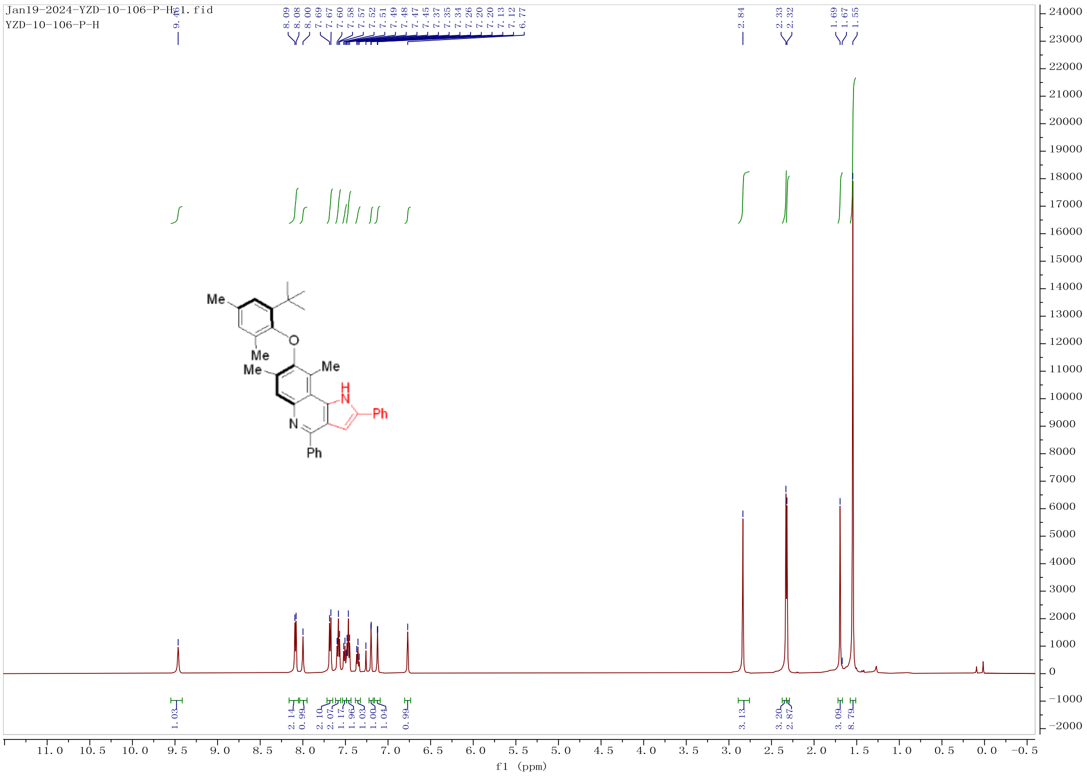

18a: ^{13}C NMR (126 MHz, Chloroform-*d*)

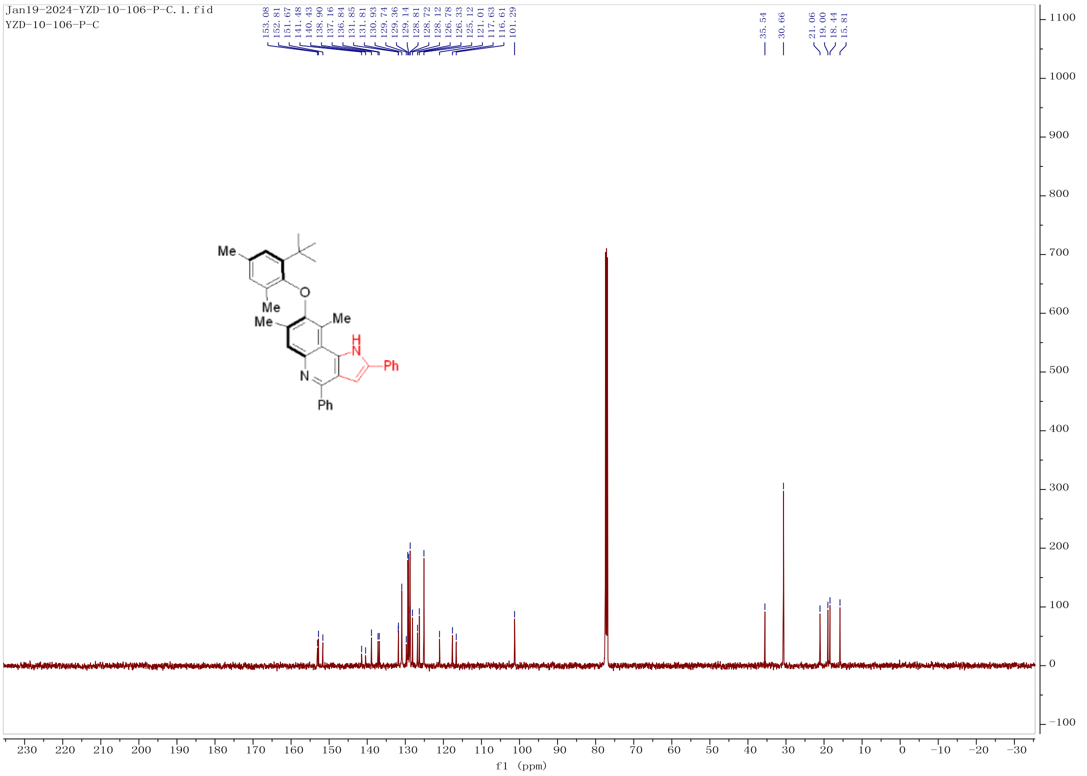

Supplement: Supplementary file 1 — Supporting Information [file ADVS-11-2403125-s001.pdf]
